# Whole-genome sequencing of cryopreserved resources from French Large White pigs at two distinct sampling times reveals strong signatures of convergent and divergent selection between the dam and sire lines

**DOI:** 10.1186/s12711-023-00789-z

**Published:** 2023-03-02

**Authors:** Simon Boitard, Laurence Liaubet, Cyriel Paris, Katia Fève, Patrice Dehais, Alban Bouquet, Juliette Riquet, Marie-José Mercat

**Affiliations:** 1 CBGP, CIRAD, INRAE, Institut Agro, IRD, Université de Montpellier, Montferrier-sur-Lez, France; 2 GenPhySE, INRAE, INP, Université de Toulouse, Castanet-Tolosan, France; 3 IFIP Institut du porc/Alliance R & D, Le Rheu, France

## Abstract

**Background:**

Numerous genomic scans for positive selection have been performed in livestock species within the last decade, but often a detailed characterization of the detected regions (gene or trait under selection, timing of selection events) is lacking. Cryopreserved resources stored in reproductive or DNA gene banks offer a great opportunity to improve this characterization by providing direct access to recent allele frequency dynamics, thereby differentiating between signatures from recent breeding objectives and those related to more ancient selection constraints. Improved characterization can also be achieved by using next-generation sequencing data, which helps narrowing the size of the detected regions while reducing the number of associated candidate genes.

**Methods:**

We estimated genetic diversity and detected signatures of recent selection in French Large White pigs by sequencing the genomes of 36 animals from three distinct cryopreserved samples: two recent samples from dam (LWD) and sire (LWS) lines, which had diverged from 1995 and were selected under partly different objectives, and an older sample from 1977 prior to the divergence.

**Results:**

French LWD and LWS lines have lost approximately 5% of the SNPs that segregated in the 1977 ancestral population. Thirty-eight genomic regions under recent selection were detected in these lines and the corresponding selection events were further classified as convergent between lines (18 regions), divergent between lines (10 regions), specific to the dam line (6 regions) or specific to the sire line (4 regions). Several biological functions were found to be significantly enriched among the genes included in these regions: body size, body weight and growth regardless of the category, early life survival and calcium metabolism more specifically in the signatures in the dam line and lipid and glycogen metabolism more specifically in the signatures in the sire line. Recent selection on *IGF2* was confirmed and several other regions were linked to a single candidate gene (*ARHGAP10*, *BMPR1B*, *GNA14*, *KATNA1*, *LPIN1*, *PKP1*, *PTH*, *SEMA3E* or *ZC3HAV1*, among others).

**Conclusions:**

These results illustrate that sequencing the genome of animals at several recent time points generates considerable insight into the traits, genes and variants under recent selection in a population. This approach could be applied to other livestock populations, e.g. by exploiting the rich biological resources stored in cryobanks.

**Supplementary Information:**

The online version contains supplementary material available at 10.1186/s12711-023-00789-z.

## Background

Numerous genomic scans for positive selection have been performed in livestock species within the last decade (see [1] for a recent review), which have benefited from the development of standardized single-nucleotide polymorphism (SNP) chips and the genotyping of millions of animals using these chips for genetic improvement. This has led to the deciphering of the molecular basis of a particularly rich selection history, starting with initial domestication and leading to a large variety of local or commercial breeds adapted to diverse environmental conditions and characterized by distinct morphology, coat color or production performance. Genomic scans for selection provide an interesting alternative to genome-wide association studies (GWAS), enabling the detection of loci that influence key traits without any *a priori* regarding the traits involved.

Identifying the trait underlying a given signature is a prime goal, yet it is also the most difficult aspect of a genomic scan for selection. In some cases, this identification can be deduced from the function of genes or transcripts found in the region, but in many other cases, it remains unknown or very hypothetical. Achieving this objective is substantially hampered by the large size of the detected regions, which often span several megabases and thus include up to dozens of genes. The use of next-generation sequencing (NGS) data narrows the size of detected regions, e.g. below 100 kb (median size) in [2]. Several other genomic scans for selection based on NGS data have been published with regard to livestock species, see e.g. [3, 4] in pigs.

Almost all genomic scans for selection published so far on livestock species are also limited by their reliance on contemporary animals that are considered to belong to the same generation. These present-day data can be used to detect past selection events because the rapid increase in frequency of favorable (i.e. positively selected) alleles generates specific patterns of genetic diversity at neighbouring loci. However, these signatures do not allow precise estimation of the timing or intensity of the underlying selection event [5]. Hence the selection process at detected loci is usually poorly understood. In contrast, sampling genomic data at different times in the past sheds light on temporal variations in allele frequencies over the sampling period. Alleles that increased in frequency can be directly observed rather than indirectly deduced from local genetic diversity patterns, thereby boosting the detection power. Furthermore, the trajectory of allele frequencies at a detected locus is informative about the time period when selection occurred and the intensity of this selection [6]. This refined characterization of the selection process at candidate regions would enable us to take advantage of external knowledge concerning the selection constraints applied on populations during a given period of their history, which are often well documented in livestock species [7], such that it would be possible to more precisely infer the trait associated with a given candidate region.

In this context, cryopreserved resources stored in gene banks offer a wonderful opportunity to detect and characterize recent selection processes in livestock species. In contrast to studies on ancient DNA time series, which document evolution over a very long time scale starting from the initial domestication [8], gene bank collections capture the evolution of genetic diversity over the last few decades via recurrent re-sampling of populations over this period [9]. The resulting short-term genomic time series allow detection of loci that have been selected as a result of recent selection objectives [10], thereby distinguishing them from those that had been selected before this modern intensive breeding period [11]. These data also inform us about the evolution of the effective population size or inbreeding over this recent period [10, 12, 13].

**Fig. 1:**
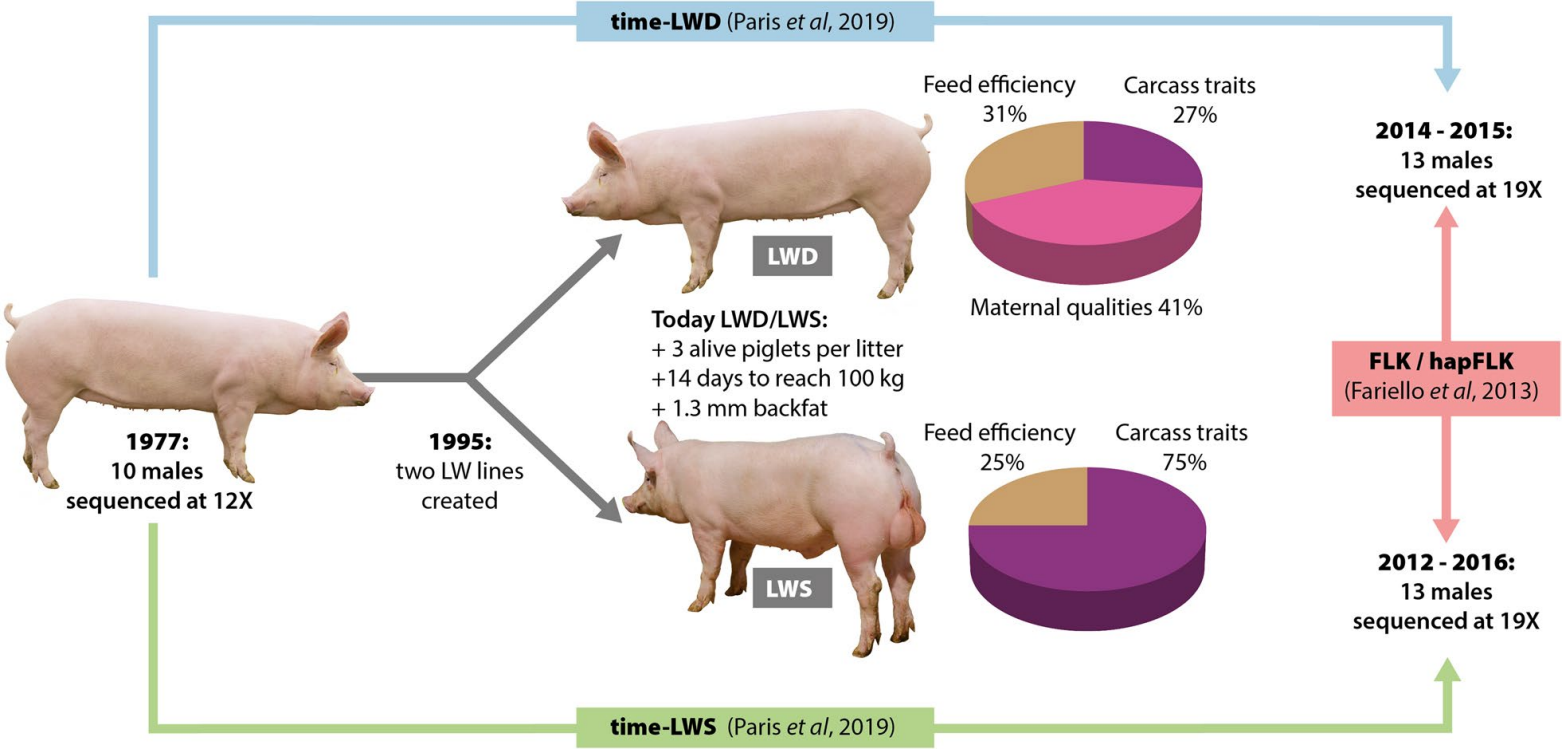
Experimental design used to detect genomic regions under recent positive selection in French LW pigs. Indicative breeding goals used in the two lines in 2017 are provided

Based on these rationales, in this study we analyze NGS data from two distinct sampling times for the French Large White (LW) pig breed. This breed was first selected for feed efficiency and carcass traits, and later for litter size. From 1995, two distinct selection schemes (Fig. 1) started based on best linear unbiased predictor (BLUP) animal model breeding values, leading to the recognition of two subpopulations in 1999: a sire line (LWS) selected for feed efficiency and carcass traits and a dam line (LWD) with greater emphasis on maternal traits [14]. Maternal traits have evolved over the years and included, for instance, the number of piglets (i.e. total born, born alive or weaned) per litter, the number of functional teats or, more recently, maternal abilities [14]. As a result of these distinct selection schemes, in 2015, LWD and LWS exhibited major phenotypic differences, with more than three additional piglets per litter in the dam line compared to the sire line, but also almost 14 more days to reach 100 kg live weight and ultimately 1.5 mm more back fat. The recent splitting of these two lines and the fact that partly distinct selection objectives have been applied since this split make French LW pigs a great model for detecting and characterizing molecular signatures of recent selection in the genome by comparing allele/haplotype frequencies between lines. Moreover, this model is even more relevant thanks to the availability of biological material collected before the split (1977 samples).

Here we provide NGS data from cryopreserved material (semen and blood biobanks) sampled from three distinct populations: LW pigs born in 1977, LWD pigs in 2014–2015 and LWS pigs in 2012–2016. We compared genetic diversity in these three samples and detected several significant signatures of recent selection in the LWD and LWS lines. By characterizing these signatures, we show that they can be classified into distinct categories of selection scenarios: convergent selection among lines, divergent selection between lines or selection specific to a single line. These different categories of regions were found to be associated with different types of traits and molecular functions. Finally, we identified a short list of possible causal variants in several of the detected regions.

## Methods

### Sampling

Thirty-six animals were studied: ten LW boars born in 1977 and 13 recent boars from each selected line that were born in 2014 and 2015 for the LWD line and between 2012 and 2016 for the LWS line. Blood or semen was available for only 11 boars born in 1977, one of which was excluded because it had no known offspring in the herd book database. The 26 recent boars were chosen based on several criteria including relatedness, external admixture from other breeds and DNA quality (see Additional file 1: Text S1). In order to preserve the reproductive potential of the cryobank, DNA was extracted from blood rather than from semen.

### Whole-genome sequencing

Library preparation and sequencing were performed by GENEWIZ [15]. Briefly, Illumina libraries were prepared with the NEBNext Ultra DNA Library Prep Kit and sequenced on an Illumina Hiseq X Ten system with the 2 × 150 bp paired-end configuration. The expected sequencing depth was 10× and 15× for boars born in 1977 and the recent ones, respectively.

### SNP calling

The different processing steps for calling SNPs and genotypes from sequenced reads (see Additional file 1: Text S2) were based on the best practices for variant discovery in DNAseq provided by the Broad Institute for GATK3 [16]. The reference genome used for this study was *Sus scrofa* version 11.1, retrieved from Ensembl. Genetic variants (SNPs and indels) were called using three callers: samtools v1.3.1 Mpileup, Freebayes v1.1.0 and GATK v3.7 HaplotypeCaller. Two sets of variants were then defined. The first set (denoted HQSNP for high quality SNPs) included only autosomal bi-allelic SNPs found by all three callers and passing through a number of GATK quality filters. The second set (denoted AV for autosomal variants) included all autosomal SNPs or indels called by at least one caller, without further filtering. HQSNP was used for all general genome-wide analyses, while AV was used for the detailed investigation of candidate regions.

### Genetic diversity

Genetic diversity was projected onto two principal components using multidimensional scaling (MDS) analysis of the identity-by-state (IBS) distance matrix between all individual pairs. This matrix was computed using PLINK 1.9 (version 1.90b5.3) and the MDS analysis was performed in R (version 3.5.1) using the cmdscale() function. The number of total and private polymorphisms in each population was computed using custom R scripts based on allele frequencies computed with PLINK 1.9. The effective population size in each modern line was estimated by comparing allele frequencies in this line with those in the 1977 population. This analysis was performed using the method of [17] as implemented in the R package NB [18], based on SNPs with a minor allele frequency (MAF) above 10% in the 1977 population.

### Detection of signatures of selection 

Genomic regions under positive selection were detected using two approaches, one based on genetic differentiation between the two modern lines and the other on the variation in allele frequencies of these two lines compared to the 1977 population (Fig. 1).

Genomic regions showing an excess of genetic differentiation between populations, compared to what would be expected under neutral evolution, were detected using the FLK [19] and hapFLK [20] statistics implemented in the hapFLK software package, version 1.4 [21]. The FLK statistic is an extension of the classical ${F_{ST}}$ statistic that accounts for differences in effective population size among populations and for the hierarchical structure of populations. In this approach, neutral evolution of allele frequencies is modeled by a population tree, with branch lengths corresponding to drift units. This neutral tree is first estimated using genome-wide data, and the deviation from this tree is then tested at each SNP by the FLK statistic. The hapFLK statistic extends FLK by measuring haplotype (rather than allele) frequency differentiation at every SNP position based on local haplotype cluster frequencies that were estimated using the approach of [22].

The FLK and hapFLK tests were applied at two different steps in our study. First, to detect candidate regions under selection in a genome-wide scan, we considered HQSNPs with MAF above 10% in the 1977 population and relied on the hapFLK statistic, which is considered to provide higher detection power than FLK [20]. HapFLK values were computed based on the two modern populations, while the 1977 population was only used as an outgroup when estimating the population tree. HapFLK values were computed using 10 haplotype clusters (parameter -K) and 10 iterations of the expectation maximization (EM) algorithm (parameter -nfit). HapFLK p values were computed according to the scaling_chi2_hapflk.py script, available on the hapFLK webpage. Significant hapFLK values were called using the qvalue R package [23] with the aim of controlling the false discovery rate (FDR) at the 20% level, i.e. q values lower than 0.2 were considered significant. FLK and hapFLK tests were also used in a second step of the study to characterize candidate regions under selection, as described in the next section.

SNPs showing an excess of allele frequency variation since 1977, as compared to what would be expected under neutral evolution, were detected in each line using the method of [24]. This method exploits the evolution of allele frequencies in a population along different sampling times (only two are available here). It is based on a hidden Markov model (HMM) approach which allows modelling both the stochastic evolution of population allele frequencies over time, as a result of genetic drift and selection (if any), and the additional noise arising as a result of the finite sample size at each time point. This method was applied to HQSNPs with MAF above 10% among the two analyzed populations (1977 and LWD or 1977 and LWS), with the effective population sizes estimated as described in the previous section. For each SNP, it provided a p value quantifying the selection evidence and an estimation of the selective advantage of the reference allele (positive if this allele is favoured, negative if the other allele is favoured). This temporal analysis of allele frequencies from 1977 to LWD (resp. LWS) is denoted ‘time-LWD’ (resp. ‘time-LWS’) throughout the manuscript.

In order to exploit linkage disequilibrium information in this temporal analysis, genomic regions with a local excess of low p values (i.e. candidate regions under selection) were detected using the local score (LS) approach proposed in [25] (see Additional file 1: Text S3). In contrast to standard windowing approaches, the LS approach can evaluate the statistical significance of outlier regions; here the LS detection threshold for each chromosome was set to obtain a chromosome-wide false positive rate (FPR) of 1%.

Regions detected by one of the three analyses described above (hapFLK, time-LWD+LS, time-LWS+LS) were combined to obtain a final set of candidate regions under selection. Regions detected by the same method and with a physical distance between regions shorter than 1 Mb between them, and overlapping regions between two or three tests, were merged into a single candidate region. To further simplify the discussion, each region was given an ID of the form SSCA:B, where A indicates the chromosome and B the integer value of the first position of this region in Mb.

### Characterisation of the signatures of selection 

Additional analyses were performed for each candidate region in order to characterize the selection scenario and identify candidate causal variants under selection. As causal variants may have been removed from the first genome scan due to insufficient quality or MAF, the FLK, hapFLK, time-LWD and time-LWS analyses described above were repeated using all available variants (AV set) within each candidate region. In addition, one hapFLK analysis including the 1977 population was conducted in order to produce visual representations of haplotype frequencies in all three populations using the -annot option of hapFLK and the hapflk-clusterplot.R R script available on the hapFLK webpage.

These more detailed results were used to classify candidate regions into four main categories. Regions showing high FLK/hapFLK values and contrasted haplotype frequencies between the two modern lines were considered under divergent selection (named ‘div’). Regions showing high time-LWD values but with little haplotype frequency differentiation between 1977 and LWS were considered to be under selection only in LWD (‘LWD’). The ‘LWS’ category was defined similarly. Finally, regions showing high time-LWD and time-LWS values but with similar haplotype frequencies between the two modern lines were considered to be under convergent selection (named ‘conv’). Two optional sub-categories were also added to this category (‘conv(LWD)’ and ‘conv(LWS)’), in cases where differentiation from the 1977 population was clearly more marked in one of the two modern lines.

Genes included in each candidate region were extracted from a gtf file reporting all genes and their positions for genome assembly Sscrofa11.1, which was downloaded from Ensembl v93 [26]. All genome-wide autosomal variants (AV set) were annotated using SnpEff, version 4.3t [27] and were classified by this software into four categories of decreasing functional impact: ‘HIGH’, ‘LOW’, ‘MODERATE’ or ‘MODIFIER’.

Candidate causal variants within each region were sought using two strategies (see Additional file 1: Text S4). The first one, which we refer to as the ‘statistical’ approach, focused on the most relevant test statistic (FLK, time-LWD or time-LWS depending on the region category) and considered the few variants with the highest value of this statistic as candidates. The second one, which we refer to as the ‘functional’ approach, considered a larger set of variants with high values of one of the test statistics, but only selected the functional variants (in the sense defined above) of this initial list.

### Overlap with quantitative trait loci

Quantitative trait loci (QTL) detected by previous association studies and located less than 2 Mb away from each candidate region were extracted from QTLdb [28]. These QTL were then classified into three categories: ‘Both’ for traits selected in the two lines with a similar weight (feed efficiency traits, see Fig. 1), ‘Both.Sire’ for traits selected in the two lines with a greater weight in the LWS line (carcass traits) and ‘Dam’ for traits selected only in the LWD line (maternal abilities). Regardless of the category, a score of 5 (resp. 1) was attributed to QTL corresponding to traits directly included in the selection index (resp. indirectly affected by a trait in the selection index). Other QTL (no direct or known indirect selection in French LW) were removed from the analysis. For each candidate region and trait category, a cumulated score was computed by summing the scores of all relevant QTL. Cumulated scores were then summed over all candidate regions from the same category, leading to a contingency table between trait and region categories. Associations between trait and region categories were quantified by the residuals of this contingency table, which were computed in R using the wtable() function. These residuals account for marginal weights of trait or region categories (i.e. for the fact that some trait or region categories are more frequent than others).

### Functional enrichment analysis

Lists of genes included in candidate regions under selection were explored with the Genecodis4 [29, 30] web tool to identify associated biological functions. For regions without genes, we assumed that selection affected a cis-regulatory variant and thus included a maximum of four genes flanking the region in the enrichment analysis using the following procedure: on each side of the candidate region, the closest gene was included if its distance to the region was less than 1 Mb, and the second closest gene was included if its distance to the first gene was less than 50 kb.

First, genes (or flanking genes) from all candidate regions were considered jointly and the functions enriched among these genes were investigated independently based on three databases: the Biological Process from Gene Ontology database (GOBP, [31]), the Kyoto Encyclopedia of Genes and Genomes database (KEGG, [32]) and the Mouse Genome Informatics database (MGI, [33]). KEGG provided access to curated signaling and metabolic pathways and MGI allowed us to extract phenotypic information from spontaneous or genetically-induced mutations in the mouse model. *Homo sapiens* was used as reference organism to overcome the less complete annotation of the pig genome. Each enrichment was associated with a p value, which was corrected for multiple tests using the false discovery rate (FDR) based on the method of [34].

Significant functions returned by these three analyses were further explored to investigate whether they were associated with specific categories of signatures. For each function and region category, the number of genes related to this function and located in a candidate region from this category was computed. The association between functions and region categories was then evaluated using a contingency table approach similar to that described in the previous section, while focusing on the 20 most significant terms. A similar analysis based on the number of candidate regions (rather than the number of genes) related to each function was also performed.

Then two distinct lists of genes corresponding to the region categories LWD, conv(LWD) and div on one hand, and LWS, conv(LWS) and conv on the other, were analyzed independently using Genecodis4. For each of the two lists, Genecodis4 queried the three databases simultaneously and identified subsets of genes with significant co-annotations for different functions.

Information on gene function and regulation (genetic and transcriptional) was retrieved from the Monarch [35] and Genecards [36] databases.

## Results

### Genetic diversity

In total 13,408,342 high quality SNPs (HQSNP set) were called in our sample of 36 LW pigs. An MDS analysis of allele frequencies at these SNPs showed that the three categories of sampled individuals (1977, LWD and LWS) were clearly differentiated and could thus be considered as three distinct genetic groups or populations (see Additional file 2: Fig. S1). The 1977 population had the highest genetic diversity: 83.1% of the HQSNPs were polymorphic in this breed, and for 6.9% of the HQSNPs, the minor (i.e. less frequent) allele was private to this population. These private alleles might either have been lost in the modern lines or not detected due to the limited sample size. Among the two modern lines, the highest diversity was observed in LWD, with 79.2% of polymorphic SNPs and 5.3% of private alleles, versus 76% of polymorphic SNPs and 4.3% of private alleles for LWS. Note that due to the short evolution time from 1977 to each modern line, private alleles observed in LWD or LWS are unlikely to come from new mutations that occurred during this period (see Additional file 1: Text S5). Thus, those private alleles most likely correspond to alleles that existed in 1977 but were not observed in our sample due to an insufficient sample size or coverage.

The highest genetic diversity in LWD was also confirmed when estimating the effective population size of each line by comparing its allele frequencies with those in the 1977 population: an effective size of 80 diploid individuals was estimated in LWD, versus 74 in LWS.

### Overview of signatures of selection 

The number of genomic regions detected under selection was 12 for the hapFLK test, 12 for the time-LWD+LS test and 16 for the time-LWS+LS test. This led to a total of 38 candidate regions under selection, after merging those that overlapped between the different tests. The size of these regions ranged from less than 1 kb to 3.2 Mb, with a median of 27 kb. One hundred and fifty-one genes (Ensembl ID and RNA gene) were located in these regions, with the number of genes per region ranging from 0 to 26 with a median of 1. A summary of these regions is provided in Table 1 and the full list of genes in each region is given in Additional file 3. A Manhattan plot summarizing the results of the three genome scans is shown in Figs. S2 and S3 (see Additional file 2: Figs. S2 and S3).

**Fig. 2:**
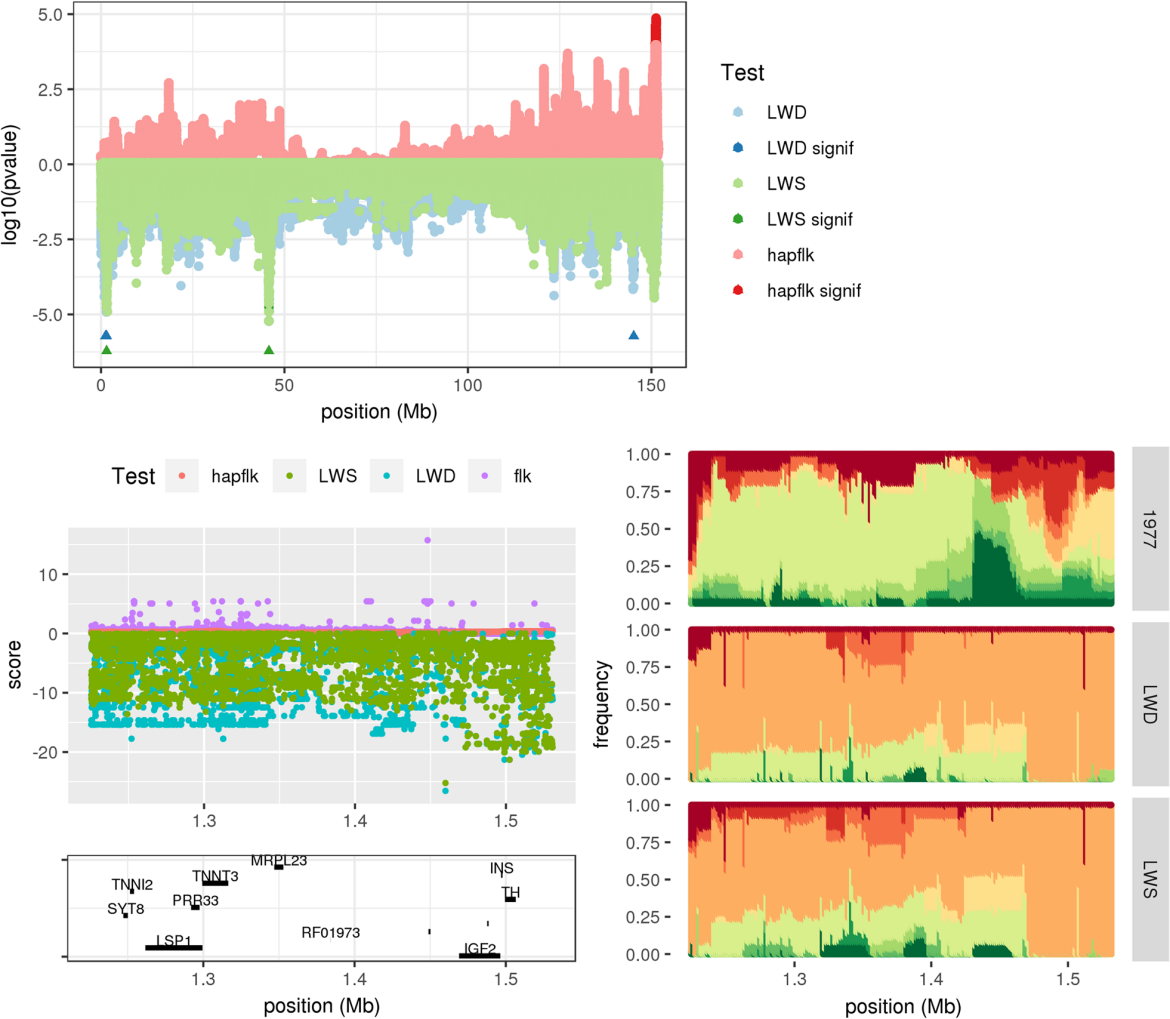
Signature of divergent selection around the *IGF2* gene. Top: Summary of the genome scan for selection on chromosome 2. P values of the time-LWD (blue), time-LWS (green) and hapFLK (red) tests are plotted in log10 scale (y axis) for HQSNPs as a function of the genomic position (x axis). Opposite signs are used for time-LWD and time-LWS on the one hand and hapFLK on the other hand, i.e. low p values correspond to highly negative points for time-LWD and time-LWS and to highly positive points for hapFLK. Significant p values are shown in darker color for the three tests. For time-LWD and time-LWS, SNPs ranked as significant by the local score approach (see “Methods” for further details) may be hidden by non-significant SNPs with higher individual p values, so significant regions for the local score were also highlighted by small triangle marks below the plot. Bottom: focus on the signature of selection detected in both LWD and LWS from 1.22 to 1.53 Mb (SSC2:1 region). The left panel shows the values of the three statistics plus FLK (in purple) for all available variants (AV set) in the region, together with genes included in the region (genes without gene name are reported with their Ensembl ID). The right panel shows the haplotype diversity in this region, estimated using hapFLK with 10 haplotype clusters (for a single EM run). For a given genomic position, each color corresponds to a different haplotype cluster and the height of the color band in a given population (represented by one of the panels) gives the frequency of the cluster in this population

A detailed study of the test statistics and haplotype frequency patterns in each candidate region, based on all variants (AV set) rather than just high-quality SNPs, revealed that these regions could be grouped into four main categories: convergent selection in the two lines (18 ‘conv’ regions), divergent selection between the two lines (10 ‘div’ regions), selection only in the LWD line (6 ‘LWD’ regions) and selection only in the LWS line (4 ‘LWS’ regions). Regions were considered to be under convergent selection when the same haplotype(s) increased in frequency in the two lines compared to 1977. An extreme example of this situation concerned the candidate region SSC2:1 (Fig. 2). This region included the *IGF2* (*insulin-like growth factor 2*) gene, which encodes a fetal growth hormone and includes a known quantitative trait nucleotide (QTN) affecting muscle growth [37] and segregating in European LW (among other breeds). This region was found to be significant with both time-LWD+LS and time-LWS+LS (Fig. 2, top) but the hapFLK and FLK values were very low (Fig. 2, bottom left), indicating low genetic differentiation between the two lines. As can be seen in Fig. 2 (bottom right), a haplotype (represented by the orange color) segregating at low frequency in the 1977 population increased to high frequency in both LWD and LWS. This increase was particularly strong at the end of the region, around 1.5 Mb, where the orange haplotype was almost fixed in the two lines. Consistent with this pattern, higher time-LWD and time-LWS values were observed in this part of the region (Fig. 2, bottom left). Interestingly, this sub-region corresponded to the location of the *IGF2* gene. Thus, the signature observed in this region was most likely due to the selection of the QTN allele that increases muscle growth in pigs. In all other regions classified as conv, only one of the two time-LWD+LS or time-LWS+LS tests was significant but both showed elevated values and the haplotype frequency pattern was similar to that in Fig. 2, although generally less pronounced. For some of these regions, the evidence for selection (value of the temporal test and observed haplotype difference with 1977) was clearly more marked in one of the two lines; we highlighted these cases by adding the ‘conv(LWD)’ or ‘conv(LWS)’ sub-categories in Table 1.

**Fig. 3:**
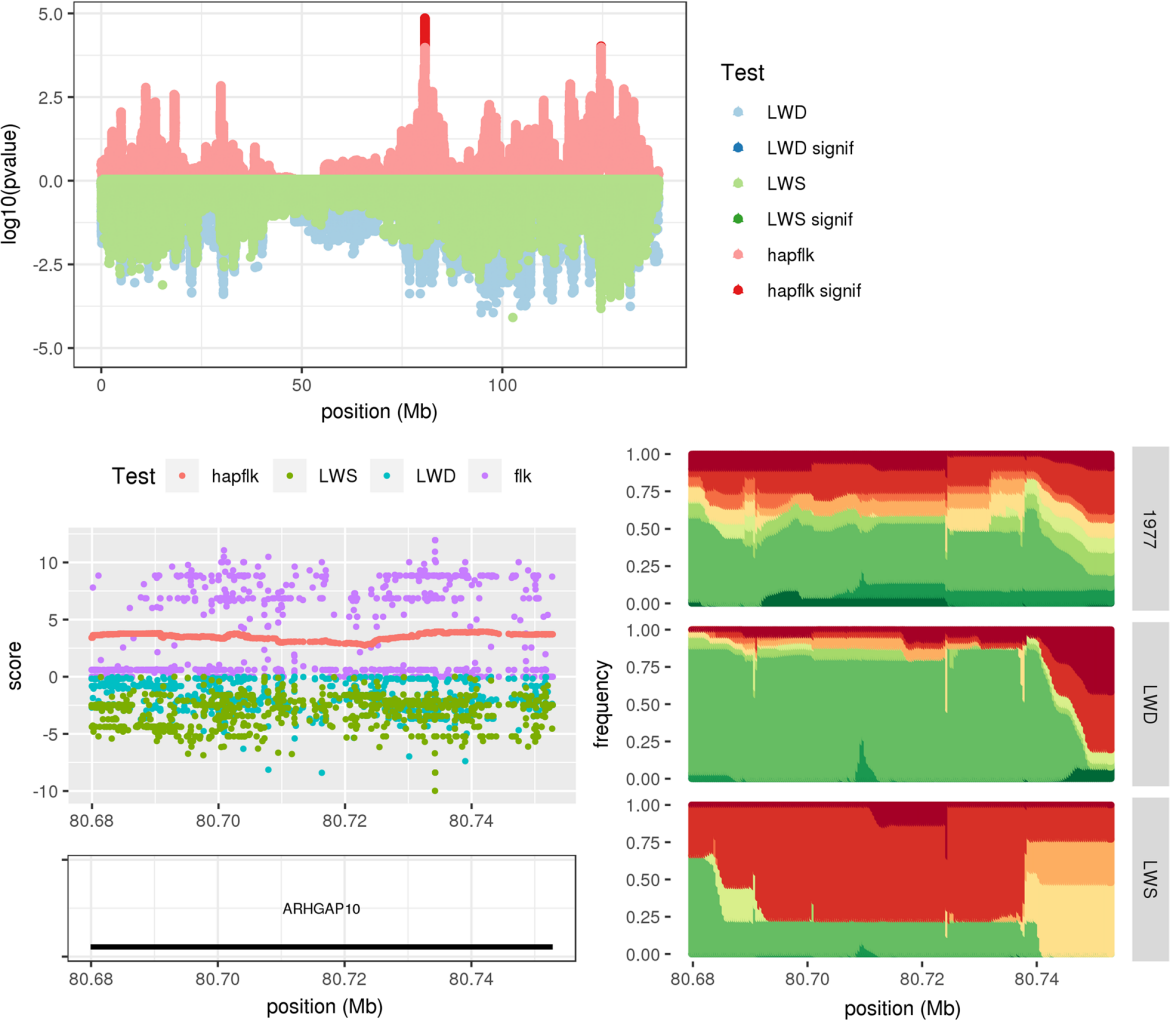
Signature of divergent selection around the *ARHGAP10* gene. This is similar to Fig. 2 but focuses on the signature of selection detected by hapFLK on chromosome 8 from 80.67 to 80.75 Mb (SSC8:80 region)

**Table 1:** Genomic regions under recent selection in French Large White pigs

ID	Chr	Start (Mb)	End (Mb)	L (kb)	WG tests	Category	Genes
SSC1:16	1	16.33	16.36	34	hapFLK	div	KATNA1 GINM1
SSC1:95	1	95.44	95.63	186	LWS	conv	3
SSC1:110	1	110.37	111.69	1328	hapFLK	div	6
SSC1:230	1	230.43	230.44	6	LWD	LWD	GNA14
SSC1:238	1	238.38	239.9	1518	LWD	conv(LWD)	20
SSC1:253	1	253.31	253.45	134	LWD	LWD	3
SSC2:1	2	1.22	1.53	305	LWD,LWS	conv	12
SSC2:45	2	45.74	45.76	20	LWS	conv(LWS)	PTH (+1)
SSC2:145	2	145.18	145.18	0	LWD	conv(LWD)	0
SSC2:151	2	151.01	151.38	371	hapFLK	div	10
SSC3:3	3	3.85	3.86	6	LWD	conv(LWD)	SLC29A4
SSC3:124	3	124.83	124.84	1	LWS	LWS	0
SSC4:18	4	18.01	18.03	17	hapFLK	div	0
SSC4:54	4	54.79	54.8	5	LWS	conv(LWS)	SNX16
SSC5:94	5	94.16	94.17	18	hapFLK	div	0
SSC6:13	6	13.87	13.87	1	LWS	LWS	(+1)
SSC6:72	6	72.3	72.31	15	LWS	conv(LWS)	VPS13D RF00156
SSC7:18	7	18.6	18.62	11	hapFLK	div	0
SSC7:85	7	85.68	85.69	1	LWS	LWS	(+1)
SSC8:80	8	80.67	80.75	72	hapFLK	div	ARHGAP10
SSC8:124	8	124.62	124.62	0	hapFLK	LWS	BMPR1B
SSC9:19	9	19.37	19.9	538	LWS	conv(LWS)	13
SSC9:96	9	96.7	98.42	1721	LWD	conv(LWD)	5
SSC9:101	9	101.3	104.51	3206	LWD	conv	26
SSC9:107	9	107.83	108.62	784	LWS	conv(LWS)	8
SSC10:23	10	23.7	23.7	2	LWS	conv(LWS)	PKP1
SSC10:62	10	62.92	62.93	12	LWS	conv(LWS)	0
SSC11:7	11	7.66	7.79	130	LWD,hapFLK	LWD	HSPH1 B3GLCT (+1)
SSC11:51	11	51.15	51.16	5	LWD	LWD	0
SSC13:99	13	99.09	99.09	3	LWS	conv	0
SSC13:109	13	109.1	110.15	1046	LWS	conv	9
SSC13:113	13	113.97	114.2	233	LWS	conv(LWS)	NAALADL2
SSC13:191	13	191.52	191.54	14	hapFLK	div	0
SSC16:69	16	69.96	70.9	941	LWD	LWD	6
SSC17:1	17	1.86	4.02	2153	LWD	LWD	8
SSC17:43	17	43.48	43.91	434	LWS	conv(LWS)	3
SSC17:60	17	60.33	60.79	466	hapFLK	div	0
SSC18:10	18	10.54	10.56	20	hapFLK	div	ZC3HAV1

Columns ‘ID’, ’Chr’, ’Start (Mb)’ and ’End (Mb)’ indicate the name and the genomic position of each region and column ‘L (kb)’ gives its length in kb. The ’WG tests’ column provides the whole genome test(s) that detected this region as significant; LWD (resp. LWS) stands for time-LWD+LS (resp. time-LWS-LS) to simplify the notations. The ’Type’ column indicates the selection scenario proposed for this region (as described in “Methods” section). The ’Genes’ column reports the number of genes found in the region. Gene names are reported for regions when there is a maximum of two genes with gene names, in this case the number of genes without gene name (only an Ensembl ID) is given in parenthesis. The full list of genes for all regions is provided in Additional file 3

In contrast to this first category, regions were considered to be under divergent selection when distinct haplotypes increased in frequency in LWD and LWS compared to the 1977 population. This resulted in high hapFLK and FLK values and all the regions in this category were initially detected from hapFLK (see Table 1). Elevated time-LWD and time-LWS values were generally observed in these regions as a result of the haplotype frequency change in the two lines, but this did not lead to a significant LS value. A typical example of this situation concerned the candidate region SSC8:80 (Fig. 3), where one first haplotype (in green) increased from a frequency of about 40% in the 1977 population to a frequency of about 80% in the LWD population, while a second haplotype of the same region (in red) increased from a frequency of about 20% in the 1977 population to a frequency of about 75% in the LWS population. This region included a single gene, i.e. *ARHGAP10* (*Rho GTPase activating protein 10*), which is known to be involved in sexual interaction, coordination and other motor capabilities in mice.

Finally, some signatures of selection concerned only one of the two lines, with the other line showing allele and haplotype frequencies similar to those in the 1977 population. Such regions were generally detected by time-LWD+LS or time-LWS+LS and showed small values for the other test. They often showed elevated values of hapFLK and two of them (SSC8:124 and SSC11:7) were actually detected with this test (see Table 1).

The behavior of the three test statistics in each category is summarized in Table 2 (upper part). Local genome scan plots and haplotype frequency plots similar to those in Figs. 2 and 3 are provided for all regions in Additional files 4 and 5, respectively.

**Table 2:** Typology of the categories of signatures of selection

		Region category					
		LWD	LWS	conv(LWD)	conv(LWS)	conv	div
Test statistic	Time-LWD	XX		XX	X	X/XX	X
	Time-LWS		XX	X	XX	X/XX	X
	hapFLK	X	X				XX
Trait category	Both			X	X	XX	
	Both.Sire		X		XX	X	
	Dam	XX		X			X

The upper part of the table summarizes the behavior of the four test statistics for each category of signatures of selection: symbol XX indicates a significant value (note that for time-LWS and time-LWD, significance is assessed by the local score approach), while symbol X indicates a suggestive but non-significant value. The lower part of the table indicates the categories of signatures of selection that are expected for a given trait category: symbol XX corresponds to the most likely signature and symbol X to other possible signatures. These expectations rely on the assumption that each signature of selection arises from selection at a single locus, i.e. they overlook the fact that signatures of selection may arise from the selection of multiple QTL segregating in the same genomic region

**Table 3:** Focus on a few biological functions under selection in LW

Biological term	Database	Region category	Nb. genes	Nb. regions	Candidate genes
Body weight	MGI	Dam	8	6	LPIN1, CAMK2A, KITLG, NR3C1
Body weight	MGI	Both.Sire	16	8	RELN, EPG5, GATA3, NRCAM, IGF2, TNIK, PNPLA8, PLCG1
Body size	MGI	Dam	9	7	B3GLCT, PDGFRB, LPIN1, KITLG, NR3C1
Body size	MGI	Both.Sire	11	6	RELN, IGF2, SFRP4, PLCG1
PI3K signaling ptw.	KEGG	Dam	3	2	PDGFRB, KITLG
PI3K signaling ptw.	KEGG	Both.Sire	5	3	MAGI2, RELN, IGF2
Preweaning lethality	MGI	Dam	9	8	KATNA1, ARHGAP10, TMTC3, RBM26, SLC6A7
Perinatal lethality	MGI	Dam	4	4	KITLG, NR3C1, PDGFRB
Calcium signaling ptw.	KEGG	Dam	3	2	PDGFRB, CAMK2A, GNA14
Osteoporosis	MGI	Dam	4	3	NR3C1
Lipid metabolic process	GOBP	Both.Sire	5	5	LPIN1, PNPLA8, PLCG1
Fatty acid metabolic process	GOBP	Both.Sire	3	3	LPIN1, PNPLA8
Insulin secretion	KEGG	Both.Sire	3	3	LPIN1
Glycogen biosynthetic process	GOBP	Both.Sire	3	2	IGF2, PTH

Biological functions listed in this table are highlighted because they are among the most significantly enriched in signatures of selection (body weight, body size and PI3K signaling pathway) or because they are specifically associated with one category of signatures of selection (other terms). The ‘Nb. genes’ column gives the number of genes associated with a given biological function and located within (or flanking) a region from a specific region category. The ‘Nb. regions’ column gives the number of distinct regions where these genes are located. The ‘Candidate genes’ column reports the genes that can be considered among the best candidate genes within their candidate region (among those counted in the ‘Nb. genes’ column). The determination of candidate genes within a candidate region is based on the local values of the statistics used to detect selection in this region, and on expression data when available. It does not account for functional information concerning the genes. Details regarding this determination procedure can be found in (see Additional file 2: Table S1). The full list of biological functions showing significant enrichment in signatures of selection is in Additional file 6

### Region categories show specific QTL contents

The observation of different categories of recent selection signatures in French LW pigs might be related to differences in selection objectives in the two lines. More precisely, traits under selection in French LW pigs could be classified into three main categories: feed efficiency traits, which have a similar weight in the selection index of the two lines, carcass traits, which are selected in the two lines but with a greater weight in the LWS line, and maternal quality traits, which are selected only in the LWD line (Fig. 1). These three categories of traits, which we respectively denote as ‘Both’, ‘Both.Sire’ and ‘Dam’, are expected to lead to different patterns of signatures, as summarized in Table 2 (lower part). For example, selection of an allele affecting a trait from the Both category is typically expected to produce a signature from the conv category; stochastic effects introduced by genetic drift or non-exhaustive population sampling might also lead to variations around this typical signature, leading to a conv(LWD) or conv(LWS) signature.

**Fig. 4:**
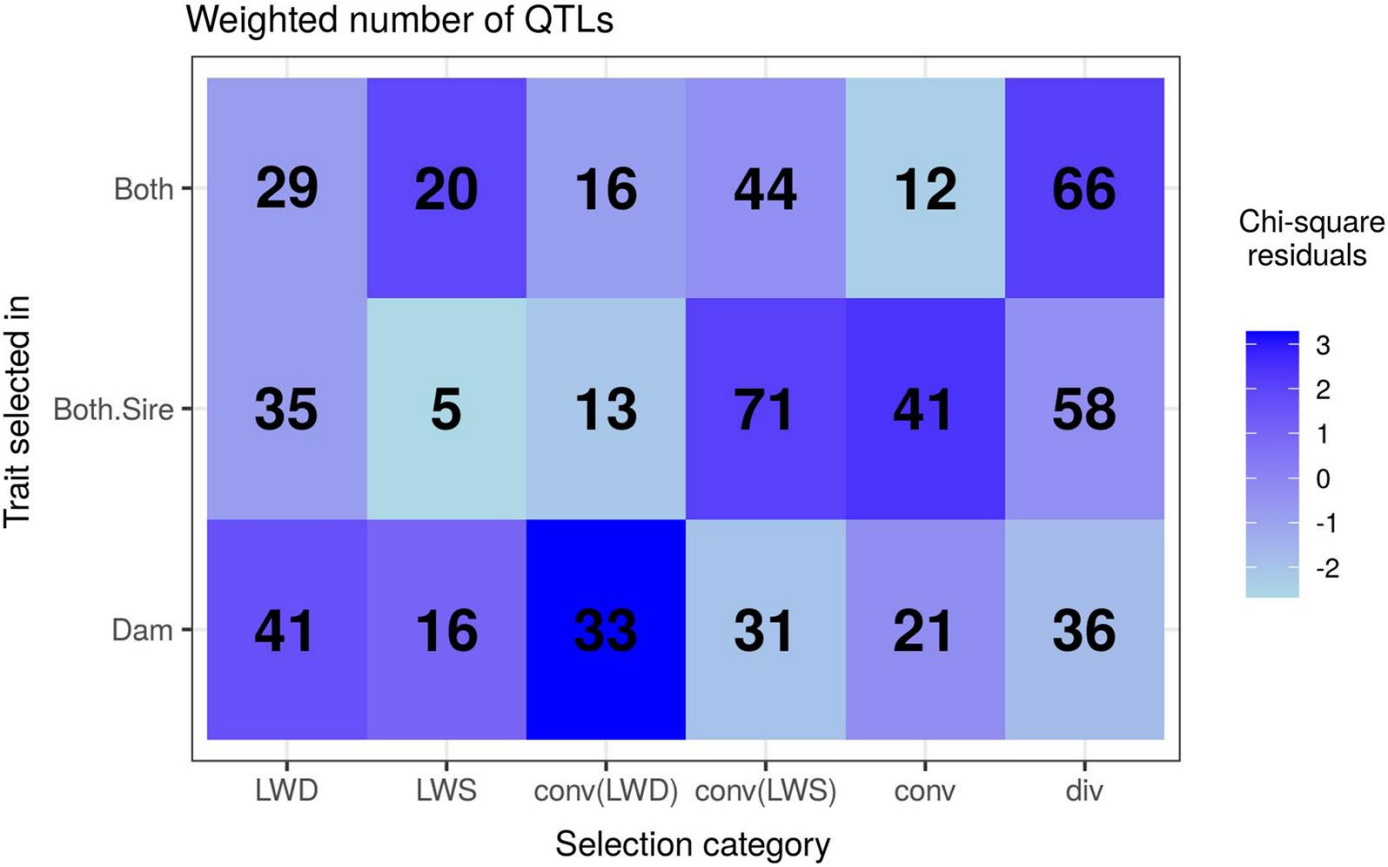
Association between trait categories and selection signature categories. For each trait category i, the weight (or cumulated score) of all QTL overlapping candidate regions from category j is reported in cell (i,j). The color of cells (i,j) represents the association between trait i and region category j, which is quantified by the residual (see the legend) of this contingency table: positive (resp. negative) residuals indicate that the weight is larger (resp. smaller) than what would be expected if trait and region categories were independent

To explore this hypothesis, we collected known QTL that overlapped with each candidate region under selection using QTLdb and studied the associations between trait categories and selection signature categories using the contingency table approach described in “Methods”. The observed association patterns (Fig. 4) were generally consistent with the expectations described above. In particular, QTL related to Dam traits showed the highest enrichment among signatures from the conv(LWD) and LWD categories, and QTL related to Both.Sire traits showed the highest enrichment among signatures from the conv and conv(LWS) categories. These results confirmed that the candidate regions detected in this study, and the selection scenarios associated with these regions, were biologically relevant.

### Biological functions under selection

**Fig. 5:**
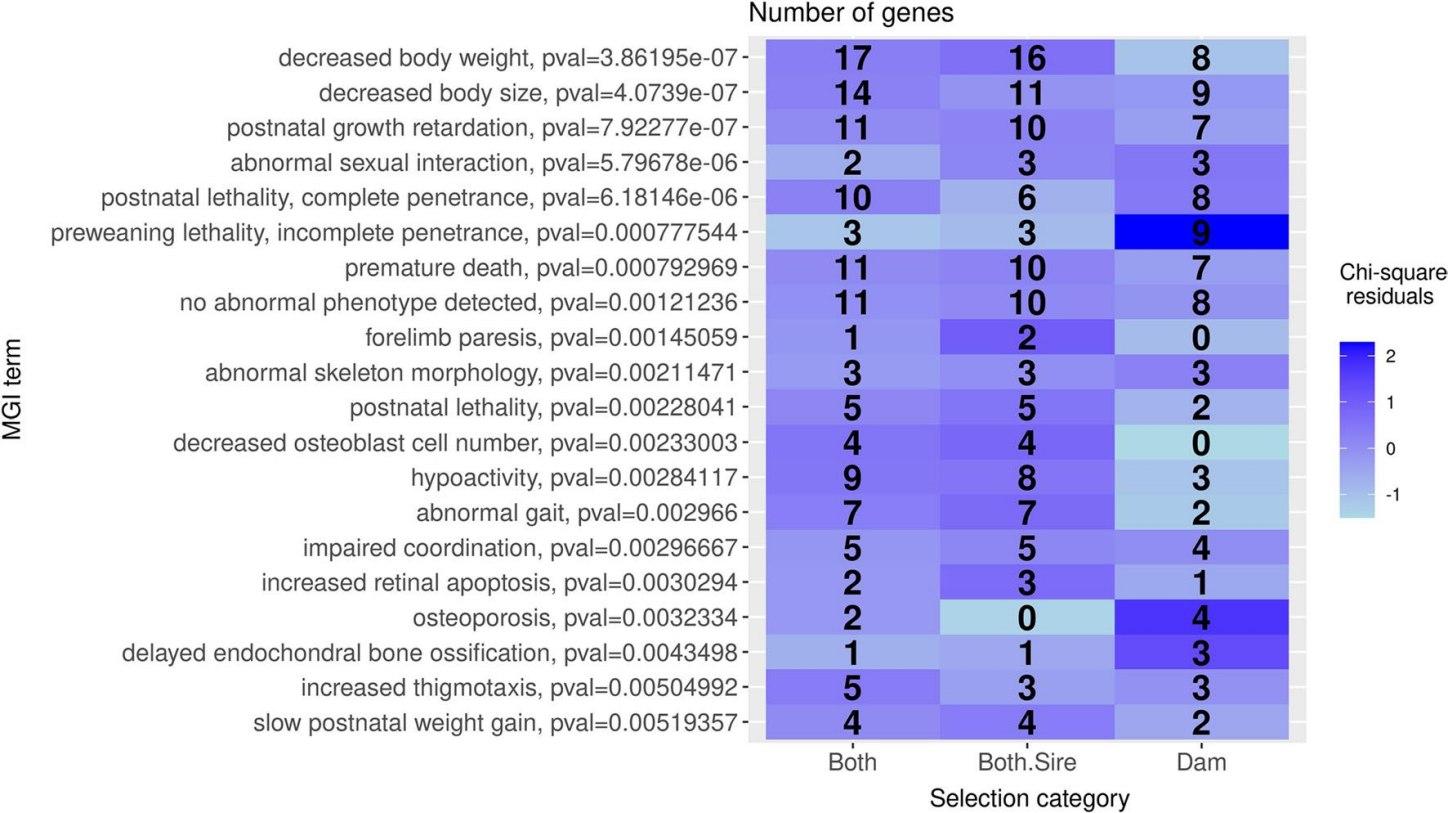
Top 20 significant MGI terms and association between these terms and groups of selection signature categories. For this analysis, signatures of selection were merged into three (overlapping) groups corresponding to the three categories of traits defined in Table 2: Both, Both.Sire and Dam. For each MGI term i, the number of genes within (or flanking) candidate regions from group j is reported in cell (i,j). The color of cells (i,j) represents the association between term i and group j, which is quantified by the residual (see legend) of this contingency table: positive (resp. negative) residuals indicate that the number of genes is larger (resp. smaller) than would be expected if MGI terms and groups of regions were independent. The p value indicated for each term quantifies the significance of this term among genes from all regions (independently of their group)

We next explored which biological functions were the most represented among candidate regions under selection, and whether these functions differed between categories of signatures of selection. Following the rationale proposed in Table 2 and supported by the QTL enrichment results shown in Fig. 4, categories of signatures of selection were merged into three groups corresponding to the Both, Both.Sire and Dam traits. We first focused on high-order phenotypes defined in the MGI database and found that three categories of these phenotypes were particularly enriched among the signatures of selection (Fig. 5): (i) body weight, size and growth, represented by four terms, including the three most significant ones, (ii) lethality and premature death (four terms) and (iii) bone physiology (four terms). Body size and growth terms were found in similar proportions in all region categories. In contrast, several terms related to lethality (‘preweaning lethality, incomplete penetrance’) or bone physiology (‘osteoporosis’, ‘delayed endochondral bone ossification’) were clearly associated with the signatures expected for Dam traits.

Two similar enrichment analyses were conducted based on biological processes from the GO database (see Additional file 2: Fig. S4) and biological pathways from the KEGG database (see Additional file 2: Fig. S5) in order to obtain a more detailed characterization of biological functions. These analyses confirmed the evidence for selection on bone physiology, as two out of the top 20 GOBP terms (‘regulation of bone resorption’, ‘osteoblast differentiation’) and one of the top 20 KEGG terms (‘calcium signaling pathway’) were related to this biological function. The KEGG ‘calcium signaling pathway’ term was clearly associated with the Dam category, while the GOBP ‘osteoblast differentiation’ term was clearly associated with the Both.Sire category. The GOBP and KEGG analyses also showed strong enrichment for a number of metabolic and signaling pathways. Among these, two GOBP terms related to the regulation of glycogen (‘positive regulation of glycogen biosynthetic process’) and fructose (‘fructose transmembrane transport’), and one related to lipid metabolism (‘lipid catabolic process’), were found to be associated with the Both.sire category.

One limit of the analyses described above is that the Both category partly overlapped the Both.Sire and Dam categories (see Table 2), which may affect the association signal between biological terms and region categories. To confirm the observed differences between the biological functions under selection in the LWD and LWS lines, we thus performed two additional analyses based on two non-overlapping sets of signatures: those expected for Dam traits (region categories LWD, conv(LWD) or div) on one hand, and those expected for Both.Sire traits (region categories LWS, conv(LWS) or conv) on the other hand. For each of these two analyses, the three MGI, KEGG and GOBP databases were queried simultaneously and significant co-annotations were detected (Additional file 6: sheets 5 and 6). These analyses confirmed the main conclusions outlined above. First, the two sets of regions provided a large number of significant co-annotation terms related to body size and growth (but these biological functions were represented by different genes in the two sets of regions, as these were non-overlapping). Second, biological functions related to early life survival or bone physiology were more significant in the analysis based on signatures expected for Dam traits, while biological functions related to glycogen or lipid metabolism were only significant in the analysis based on signatures expected for Both.Sire traits (see Additional file 1: Text S6).

### Towards causal variants

The use of whole-genome sequencing data provides access to a very high proportion of the common variants segregating in the sample, which is promising for the identification of causal variant(s) in each candidate region under selection. Indeed, previous genome scans for selection based on NGS data provided examples of regions where the highest FLK value corresponded to the causal variant [2, 38]. Based on this principle, we looked for potential causal variants in all candidate regions under selection identified in this study using two strategies: one ’statistical’ approach based only on the values of the different test statistics within the region, and one ’functional’ approach combining this information with functional information obtained from SnpEff for each variant (see Additional file 1: Text S4). Candidate variants obtained by these approaches are provided in Additional file 7.

The signature detected around the *IGF2* gene provides an interesting case study to evaluate the potential of this strategy because the causal variant at this gene has been previously identified [37]: it is an SNP at position 1,483,817 bp on the *Sscrofa11.1* assembly used in this study. However, the region is hard to sequence due to its high GC content [37] and only nine animals (out of 36) were called at this position in the AV set. Consequently, this true causal variant was not included in the list of candidate variants selected for this region, although mapped reads were consistent with the selection signature (all individuals from LWD and LWS carried the T allele with a positive effect on muscle growth). This example illustrates that the quest for causal variants within each region may be affected by the local call rate; we thus accounted for this effect when investigating other regions under selection.

Outside the *IGF2* region, five convincing candidate variants with a putative functional effect were identified: a synonymous variant (LOW impact) in the *EPG5* gene (*ectopic P-granules autophagy protein 5 homolog*, SSC1:95 region), a missense (MODERATE impact) and a synonymous (LOW impact) variants in the *PTH* gene (*parathyroid hormone*, SSC2:45 region), a synonymous variant (LOW impact) in the *VPS13D* gene(*vacuolar protein sorting 13 homolog D*, SSC6:72 region) and a start codon gain variant (LOW impact) in the 5′ UTR region of the *RPL22L1* gene (*ribosomal protein L22 like 1*, SSC13:109 region) (see Additional file 1: Text S4).

Many candidate variants with no functional effect (according to SnpEff) were also detected with the statistical approach. In particular, several regions with a high call rate at all positions included a couple of candidate variants that clearly stood out compared to the others, e.g. two intronic variants in the *GNA14* gene (*G protein subunit alpha 14*, SSC1:230 region) and two intronic variants in the *PKP1* gene (*plakophilin 1*, SSC10:23 region). Several intergenic regions, e.g. SSC2:145, SSC3:124 or SSC13:191, also included such promising candidate variants. Although such variants are likely regulatory and would be difficult to biologically validate, it was interesting to note that in many regions under recent selection in LW, the causal variant under selection could be identified in a list of no more than two or three candidates (Additional file 7). In the case of the SSC3:124 region, the hypothesis of a regulatory variant under selection is supported by the fact that *LPIN1* (*lipin 1*), one of the genes flanking this intergenic region, is differentially expressed between LWS and LWD (see Additional file 1: Text S7 and Additional file 8).

## Discussion

### A powerful design to detect and characterize recent signatures of selection

In this study, we described molecular signatures of recent selection in French LW pigs using an original design (Fig. 1), including (i) a comparison of two lines which split about 20 generations ago and were subsequently derived based on partly distinct selection objectives, (ii) a comparison of these two modern lines with an ancestral population more ancient than the divergence of the two lines, and (iii) an analysis of all of these samples with whole-genome NGS data. The combination of these three features not only allowed us to detect signatures with high power but also to characterize these signatures (selection scenario, candidate genes or variants, putative functions under selection) with much greater precision than usual genomic scans for selection based on SNP data from modern samples. A comparison of haplotype frequencies in the two modern lines using the hapFLK test [20] led to the detection of ten genomic regions that were divergently selected in the two lines. A comparison of allele frequencies in these lines with those in the ancestral population using a dedicated method that takes advantage of genomic time series data [24] further led to the detection of six regions selected only in the LWD line, four selected only in the LWS line and 18 convergently selected in the two lines. This high proportion of convergent signatures may seem surprising for lines that were selected independently under different objectives. However, the selection objectives were only partly distinct, with feed efficiency and carcass traits being important for both lines. Besides, there were almost 20 years between the 1977 population and the creation of the two lines, so any genomic change occurring during this period would necessarily be shared between them. Finally, a closer look at the 18 regions under convergent selection revealed that only five of them showed very similar genetic diversity patterns in the two lines (conv regions), while four showed a more marked shift from 1977 in LWD (the conv(LWD) regions) and nine showed a more marked shift from 1977 in LWS (the conv(LWS) regions). These regions may have resulted from selective pressure that was first applied in the ancestral population from 1977 to 1995 and then maintained in only one of the two lines. Alternatively, they may include variants contributing to a polygenic trait that is still under selection in the two lines, but with effects that are relatively weak compared to variants included in pure conv regions, and thereby their effective selection in a given line would be more stochastic.

One important point to outline is that these 18 regions under convergent selection could not have been detected by comparing the two modern lines, as convergent selection does not increase population differentiation. Besides, these signatures are likely too recent to be detected with other standard approaches based on local changes in genetic diversity or LD [11, 39]. Regions that were selected only in LWS or LWD left a small signature of excessive differentiation between lines, but this signal was too small to be detected with high confidence. Besides, studying such regions using modern data only would likely not be sufficient to determine which population was selected (i.e. which changed in the recent past). For all of these reasons, the added value of the ancient population from 1977 for the detection and characterization of signatures of selection was considerable. Sampling this population also allowed us to assess the evolution of genetic diversity between 1977 and 2015 and to estimate the average effective population size of each line during this period.

The use of NGS data was also decisive: it increased the detection power, reduced the size of the detected regions and consequently the number of genes included in these regions, while providing access to virtually all SNPs and short indels within each region. This highlighted candidate genes and variants in many of the detected regions, which in turn improved our understanding of the functions under selection (see below) and of the molecular mechanisms (structural, regulatory, etc.) mediating this selection (see Additional file 1: Text S8).

Two potential limitations of our experimental design are the small sample sizes used in each population (10 to 13 individuals) and the relatively low sequencing depth of the 1977 sample ($\approx$ 10X per individual). The stochasticity arising from sampling a finite number of individuals is explicity modelled in the temporal method of [24], so the small sample sizes used here cannot have led to an excess of false positive selection signals, but may have reduced the detection power. Sample size is not curently accounted for by hapFLK, so we cannot entirely exclude that some of the regions detected by this specific approach were to some extent biased by the random sampling of haplotypes within the modern populations. Besides, while hapFLK regions were detected at a FDR threshold of 20% (implying that two or three of these 12 regions are expected to be false positives), none of them would be significant at a FDR threshold of less than 10%. This moderate statistical support may be related to sample size, but also to less manageable factors such as the small population sizes or the quite large overlap between selection objectives in the two lines (discussed above).

To account for the limited sequencing coverage, we detected signatures using only high-quality SNPs, for which low-quality genotype calls were treated as missing data (HQ set). As the two methods used in this study can deal with missing data and combine evidence from multiple SNPs to detect selection in a region, we believe that the sequencing depth of the 1977 sample had a very minor effect on the regions detected. However, it may affect the identification of the causal variant in a region, as revealed when studying the *IGF2* region. For this reason, our procedure to identify potential causal variants accounted for the call rate throughout the region. In spite of all of these precautions, we acknowledge that larger samples would probably lead to even better results than those presented here and we recommend increasing the sample size in future genomic scans for selection. The influence of the number and size of samples used to detect selection from genomic time series has for instance been discussed in [6].

### Biological functions under selection

The annotation of the genes within or flanking candidate regions in Table 1 provided interesting insight on the biological functions and traits that were most likely impacted by recent selection in LW. Here we discuss several key conclusions of this functional enrichment analysis, which are also summarized in Table 3.

#### Traits under selection in the two lines

When characterizing biological functions through phenotypes from the MGI database, the strongest enrichment was found to be related to body weight, body size and growth and concerned both the Dam and Both.Sire regions (Fig. 5). Genes related to these functions were found in many distinct candidate regions (Table 3). This provides strong evidence that these functions were under selection because several distinct variants were mobilized rather than a single variant affecting several genes due to hitchhiking. Besides, several of these genes represent potential candidate genes in their region, based on the local values of the statistics used to detect selection or on the expression data available from [40] (see Additional file 2: Table S1). These results are consistent with the strong selection for carcass traits that has been applied in the two LW lines in recent decades (Fig. 1).

More specifically, the selection for body weight, body size and growth could have been partly driven by modifications in the PI3K signaling pathway, which was the second most significant KEGG term (see Additional file 2: Fig. S5). Indeed, genes from several distinct regions were related to the PI3K signaling pathway in the Both.Sire and Dam categories (Table 3), and several of these genes led to a significant co-annotation term that also included ‘body size’ or ‘postnatal growth’ (Additional file 6: sheets 5 and 6). PI3K genes were identified as being a checkpoint involved in the regulation of muscle mass [41, 42] by mediating many signaling processes. These signaling processes impact both muscle development (modifying the extracellular matrix and affecting the actin cytoskeleton) and metabolism (glycolysis, glycogenesis, and fatty acids) [40]. The PI3K-Akt signaling pathway was one of the most significantly enriched functions among the genes showing differential expression in four European pig breeds, including the Large White breed [40].

#### Traits under selection in the LWD line

Enrichment analyses also identified several biological functions that were more specifically selected in one of the two lines. A first example concerned early life survival, with greater evidence of selection in the LWD line. In particular, nine genes from eight distinct Dam regions were associated with the ‘preweaning lethality’ term, which was found to be much more significantly enriched in this analysis than in that based on Both.Sire regions. Five of these nine genes represent convincing candidate genes within their regions (Table 3 and see Additional file 2: Table S1), thereby further reducing the possibility that this function was called based on hitchhiker genes. One of these genes, i.e. *KATNA1*, has been associated with brain development, epididymis development and behavior in humans and mice, and the findings from the study on the *KATNA1* knockout mice also underlined a role in neurogenesis [43]. Similarly, four genes from four distinct regions were associated with ‘perinatal lethality’ in Dam regions (Additional file 6), i.e. a term that was not significant in Both.Sire regions. Candidate genes in these regions (Table 3 and see Additional file 2: Table S1) include the *PDGFRB,*  which encodes a growth factor involved in the development of the cardiovascular system. Altogether, these observations provide strong molecular evidence that early life survival has been more specifically selected in the LWD line. This could be related to the great weight placed on maternal abilities that is included in the selection index of this line, in contrast with the LWS line (Fig. 1). These results are also consistent with the QTL enrichment analysis of signatures, which showed that QTL associated with maternal ability traits were more often found in LWD and conv(LWD) regions than in other categories (Fig. 4).

Bone physiology and calcium metabolism were also found to be more specifically under selection in the LWD line, as several terms such as ‘endochondral bone ossification’, ‘osteoporosis’, ‘calcium signaling pathway’ or ‘calcium ion transport’ were significantly enriched only in Dam regions. In particular, the ‘calcium signaling pathway’ term was associated with three genes from two distinct regions, which all represent convincing candidate genes. Calcium needs are proportional to milk production, and a calcium deficit during lactation could be temporarily offset by the mobilization of mineral bone resources [44]. Thus, this suggests that the molecular signatures observed on genes related to calcium metabolism and bone formation are a natural consequence of the selection for maternal abilities applied in the LWD line.

#### Traits under selection in the LWS line

In contrast, molecular functions related to lipid and glycogen metabolism were more specifically found to be selected in the LWS line, as several terms such as ‘lipid metabolic process’, ‘fatty acid metabolic process’, ‘positive regulation of glycogen biosynthetic process’ or ‘insulin secretion’ were significantly enriched only in Both.Sire regions (Additional file 6). All of these terms are based on genes from several distinct regions, many of which are also good candidate genes (Table 3 and see Additional file 2: Table S1). One of these, i.e. *PTH* (*parathyroid hormone*), is one of the two genes within the SSC2:45 region and includes missense and synonymous variants with very high statistical evidence for selection. This gene encodes a calcium regulatory hormone involved in bone mineralization, and is also a regulator of glucose and glycogen metabolism. Besides, the SSC2:45 region overlaps a QTL for residual feed intake in the Duroc breed [45]. Another interesting candidate gene is *LPIN1*, which is one of the two genes flanking the intergenic SSC3:124 region and has a significantly lower expression in LWS than in LWD [40]. This lower expression could result from the selection for leaner animals in the LWS line, compared to the common ancestral LW line. Indeed, *LPIN1* includes an SNP that has been associated with the percentage of leaf fat and intramuscular fat in Chinese pigs [46], and it has also been found to be overexpressed in adipose tissue of fatter pigs [47]. Note also that QTL associated with carcass traits were more often found in conv and conv(LWS) regions than in the other categories (Fig. 4), which is consistent with the fact that these traits were selected in both lines but with a greater weight in LWS (Fig. 1).

#### Other candidate genes under selection

Several other candidate genes under selection in LW (*BMPR1B*, *PKP1*, *RORA*, *SEMA3E*, and *ZC3HAV1*) are worth mentioning, although they were not associated with the biological terms discussed above (see Additional file 1: Text S9).

### Perspectives

The list of regions under recent selection in French LW pigs and the detailed characterization of many of these regions provide a solid basis for future studies aimed at detecting QTN associated with production traits in this breed. Putative functions under selection in each region and information about the line(s) and haplotype(s) that were selected may help in designing association or linkage studies by selecting appropriate animals and traits. Insight on candidate genes and variants may also suggest appropriate functional validations that could be performed in some regions. Without going into further validations, the list of candidate variants provided for each region (Additional file 7) may also be directly used to select animals with desired alleles, at least for regions where selection could be related, with high confidence, to a given biological function. The fact that we used animals for which frozen semen was still available may be highly advantageous for these future possible applications (QTN detection or animal selection), as it paves the way for inseminating live sows with semen carrying a specific allele or haplotype found to be beneficial in our study. Semen from 1977 or LWS animals carrying alleles that appear to be lost (or very rare) in LWD could also be used to restore genetic diversity, although caution is needed regarding the potential impact on the genetic value. Independently of our results concerning diversity or selection, the 36 LW sequences produced in this study, ten of which represent ancestral diversity sampled in 1977, provide a rich resource for the imputation and phasing of animals genotyped at lower density in this breed and will thus help enhance the power of future GWAS in pigs.

## Conclusions

Genetic diversity in modern French LWD and LWS lines was found to be eroded compared to the 1977 ancestral population, which included approximately 5% more SNPs. Thirty-eight genomic regions including 151 genes revealed significant evidence of recent selection in these lines. Six of these regions were selected only in the LWD line, four were selected only in the LWS line, 18 were selected for the same haplotype in the two lines (convergent selection) and 10 were selected for distinct haplotypes in the two lines (divergent selection). Several biological functions were found to be significantly enriched among the genes included in these selection signatures: body size, body weight and growth regardless of the signature category, early life survival and calcium metabolism in signatures expected more specifically in the Dam line and lipid and glycogen metabolism in signatures expected more specifically in the Sire line. One of the most significant signatures was located around the *IGF2* gene, consistent with the strong selection on muscularity that resulted in the fixation of a QTN with major effects on muscle growth in this gene [37]. Many other candidate genes under selection could be proposed because they were the only genes in a detected region, they included significantly higher values of the test statistic than all other genes in the region and/or they were differentially expressed between lines, e.g. *ARHGAP10*, *BMPR1B*, *GNA14*, *KATNA1*, *LPIN1*, *PKP1*, *PTH*, *SEMA3E* or *ZC3HAV1*. A short list of candidate variants could also be identified for some of these genes. These results illustrate that sequencing the genome of animals at several recent time points generates considerable insight into the traits, genes and variants under recent selection in a population. This approach could be applied to other livestock populations, e.g. exploiting the rich biological resources stored in cryobanks.

## Supplementary Information

**Additional file 1: Text S1.** Sampling. Details on the sampling procedure. **Text S2.** SNP calling. Details on the SNP calling procedure. **Text S3.** Local score approach. Details on the local score approach. **Text S4.** Identification of causal variants under selection. Details on the approach used to detect causal variants, and on the results obtained with this approach. **Text S5.** Private alleles. Details on the results obtained regarding private alleles. **Text S6.** Biological functions under selection. Details on the results obtained regarding the biological functions under selection. **Text S7.** Insight from a previous gene expression study. Details on the approach used to explore gene expression in candidate regions, and on the results obtained with this approach. **Text S8.** Molecular mechanisms mediating selection. Discussion on the most likely molecular mechanism (regulatory or protein-coding) mediating selection in each the 10 div regions. **Text S9.** Candidate genes under selection. Details on several interesting candidate genes under selection. **Figure S6.** Distribution of p values obtained by the time-LWD and time-LWS tests. High-quality SNPs (HQSNP set) with a MAF greater than 0.1 were considered. **Figure S7.** Correlation of expression levels in the muscle measured in [40] for 76 probes corresponding to 48 genes found under selection in our study. Correlation between two probes was computed based on normalized expression for Large White sire (n = 10) and dam lines (n = 41) at these two probes. The ordering of probes on the graph follows from a hierarchical clustering of the correlation matrix. Only significant correlations (p value < 0.05) are shown. **Figure S8.** Boxplot of muscle expression levels in LWD and LWS for the 12 genes found differentially to be expressed between the two lines. All genes are differentially regulated between the two lines with a p value < 0.05. None of these genes have an adjusted p value < 0.05. Additional File 1 cites the following references: 2, 24, 25, 40, 48–61.

**Additional file 2: Figure S1.** MDS analysis of the genetic diversity observed in French LW pigs. High-quality SNPs (HQSNP set) with a MAF greater than 0.1 were considered. **Figure S2.** Summary of the genome-wide scan for recent selection in French LW pigs, chromosomes 1 to 9. Each panel of the figure shows the results of the three tests for selection for a different autosome. P values of the time-LWD (blue), time-LWS (green) and hapFLK (red) tests are plotted in log10 scale (y axis) according to genomic position (x axis). Opposite signs are used for time-LWD and time-LWS on the one hand and hapFLK on the other hand, i.e. low p values correspond to highly negative points for time-LWD and time-LWS and to highly positive points for hapFLK. Significant p values are shown in darker color for the three tests. For time-LWD and time-LWS, SNPs ranked as significant by the local score approach (see “Methods” for further details) may be hidden by non significant SNPs with higher individual p values, so significant regions for these tests were also highlighted by small triangle marks below the p value plot. **Figure S3.** Summary of the genome-wide scan for recent selection in French LW pigs, chromosomes 10 to 18. See Figure S2 for details. **Figure S4.** Top 20 significant GO terms for Biological Processes and association between these terms and groups of selection signature categories. See Fig. 5 for details. **Figure S5.** Top 20 significant KEGG terms and association between these terms and groups of selection signature categories. See Fig. 5 for details. **Table S1.** Determination of candidate genes in several selection signatures. This table reports all the genes that (i) can be considered as good candidate genes within their region (based on criteria described below) and (ii) are associated to one of the enriched biological functions listed in Table 3 (or more generally whose biological function is consistent with selection objectives in LW). For a given region of interest, genes that represent even better candidates than those selected based on condition (ii) are also included. Column ‘Nb. genes’ gives the number of genes in a region, and column ‘Gene ID’ lists the candidate genes. Column ‘Local peak’ indicates whether the most relevant test statistic for the region shows a local peak within or close to the gene (see Additional file 4 for summary plots of the four test statistics in all candidate regions); this column takes value NA when no or a single gene is observed in a region. Column ‘Diff. expr.’ indicates whether the gene was found differentially expressed between LWS and LWD in [40]; this column takes value ‘no data’ for genes that were not included in this study. Column ‘Causal variant’ indicates whether a causal variant under selection is known for this gene, or if a small number of potential candidate variants have been identified in our study (these candidates may be functional or not, see the text for more details). Based on all these observations, column ‘Overall evidence’ quantifies the evidence for each candidate gene to be the one under selection in a given region.

**Additional file 3.** Full list of genes included in genomic regions under recent selection in French Large White pigs. Similar to Table 1 but with the full list of genes within each candidate region.

**Additional file 4.** Summary plots of the scan for selection for all candidate regions of Table 1. Each file of this archive corresponds to a different candidate region, whose genomic position can be read in the file name. The top panel shows the results of the four tests for selection applied in the region. Values of the time-LWD (in blue), time-LWS (in green), hapFLK (in red) and FLK (in purple) tests are plotted (y axis) according to genomic position (x axis). Opposite signs are used for time-LWD and time-LWS on the one hand and hapFLK and FLK on the other hand, i.e. high test statistic values correspond to highly negative points for time-LWD and time-LWS and to highly positive points for hapFLK and FLK. All variants (AV set) were considered for these analyses. The bottom panel shows the position and name of genes located in the region.

**Additional file 5.** Haplotype frequency plots for all candidate regions of Table 1. Each file of this archive corresponds to a different candidate region, whose genomic position can be read in the file name. It shows the haplotype diversity in this region, estimated using hapFLK with 10 haplotype clusters (for one single EM run). For a given genomic position, each color corresponds to a different haplotype cluster and the height of the color band in a given population (represented by one of the panels) gives the frequency of the cluster in this population. All variants (AV set) were considered for these analyses.

**Additional file 6.** Functional enrichment for lists of genes within (or flanking) candidate regions under selection. Sheet 1. List of genes within or flanking candidate regions; region name and category are reported for each gene. Sheets 2–4. Enriched functions among genes from all selection categories, based on GOBP (sheet 2), KEGG (sheet 3) or MGI (sheet 4) databases. Sheet 5. Co-annotation enrichment among genes from the LWD, conv(LWD) and div categories. Sheet 6. Co-annotation enrichment among genes from the LWS, conv(LWS) and conv categories. P values were adjusted for multiple tests using the Benjamini–Hochberg correction (BH < 0.05). Co-annotation enrichment (sheets 5–6) was performed with Genecodi4 using the human genome as reference, merging annotations from GOBP, KEGG and MGI databases. The minimal number of genes used to find enrichment is given in brackets; this parameter is 2 or 3 according to the total number of genes per condition tested.

**Additional file 7.** Candidate causal variants in each candidate region under selection. This archive contains three types of results. (i) Each file with suffix .annot corresponds to a different candidate region, whose genomic position can be read in the file name. It provides a list of candidate variants detected by the statistical approach in this region, together with their SnpEff annotation. (ii) The all strong.func file provides a list of candidate variants with a functional effect (according to SnpEff) selected by this approach for all regions, together with their annotation. The all weak.func file provides the same, but for variants that could only be detected by the functional approach. (iii) Each file with the suffix .png corresponds to a different candidate region, whose genomic position can be read in the file name. It provides a plot showing the values of the relevant statistic(s) in this region with additional details concerning the status of each variant: ‘invalid’ (i.e. with low call rate or allele frequency patterns inconsistent with the type of selection in the region), ‘valid’ (high call rate and consistent allele frequency patterns) or ‘candidate’ (those ‘valid’ variants with the highest value of the statistic, also listed in the .annot file). When functional candidate variants exist in a region, these are plotted in black, while all other variants are plotted in a color depending on the test represented. Finally, the rate of missing genotypes in sliding windows of 2 kb are represented by the black points on the negative part of the y axis, from 0 (no missing data) to − 1 (only missing data). Horizontal dashed lines at − 0.5 and − 1 help visualize the missing rates achieved at a given position.

**Additional file 8.** Annotation of genes included in candidate regions under selection. Each line of the table (grouped by candidate region) corresponds to a gene and reports several types of information concerning this gene. Gene description and gene name were extracted from the Ensembl Sscrofa11.1 (GCA 000003025.6) annotation. The position of each gene on the genome is specified in bp (gene start and end). Annotations for RNA genes were retrieved from the Rfam database. Corresponding probes from the GSE56011 expression dataset are given for 48 genes together with the Wilcoxon test result (p value and BH adjusted p value) comparing gene expression between LWS (n = 10) and LWD (n = 41), see Additional file 1 for more details.

## Data Availability

DNA samples of the animals used in this study are stored at the @BRIDGE platform which, like the National Cryobank, is part of the CRB-Anim infrastructure (see below). Raw sequencing data produced in this study (fastq files) can be found at https://www.ebi.ac.uk/ena under accession number PRJEB51909. Genotype datasets resulting from these data (HQSNP and AV) can be found at 10.5281/zenodo.6415023. These two resources will be made publically available upon acceptance of the manuscript. Gene expression data supporting the results of this article are available in the Gene Expression Omnibus (GEO) repository, http://www.ncbi.nlm.nih.gov/geo, under accession number GSE56011. The data analyses described in this study can be reproduced using the code available at https://github.com/sboitard/LWseq_analysis.
