# Supplementary material for: Whole-genome sequencing of cryopreserved resources from French Large White pigs at two distinct sampling times reveals strong signatures of convergent and divergent selection between the dam and sire lines
Source: Genet Sel Evol. 2023 Mar 2;55:13. doi: 10.1186/s12711-023-00789-z (PMC9979506; doi:10.1186/s12711-023-00789-z)
Supplement: Supplementary file 1 — Additional file 1: Text S1. Sampling. Details on the sampling procedure. Text S2. SNP calling. Details on the SNP calling procedure. Text S3. Local score approach. Details on the local score approach. Text S4. Identification of causal variants under selection. Details on the approach used to detect causal variants, and on the results obtained with this approach. Text S5. Private alleles. Details on the results obtained regarding private alleles. Text S6. Biological functions under selection. Details on the results obtained regarding the biological functions under selection. Text S7. Insight from a previous gene expression study. Details on the approach used to explore gene expression in candidate regions, and on the results obtained with this approach. Text S8. Molecular mechanisms mediating selection. Discussion on the most likely molecular mechanism (regulatory or protein-coding) mediating selection in each the 10 div regions. Text S9. Candidate genes under selection. Details on several interesting candidate genes under selection. Figure S6. Distribution of p values obtained by the time-LWD and time-LWS tests. High-quality SNPs (HQSNP set) with a MAF greater than 0.1 were considered. Figure S7. Correlation of expression levels in the muscle measured in [40] for 76 probes corresponding to 48 genes found under selection in our study. Correlation between two probes was computed based on normalized expression for Large White sire (n = 10) and dam lines (n = 41) at these two probes. The ordering of probes on the graph follows from a hierarchical clustering of the correlation matrix. Only significant correlations (p value < 0.05) are shown. Figure S8. Boxplot of muscle expression levels in LWD and LWS for the 12 genes found differentially to be expressed between the two lines. All genes are differentially regulated between the two lines with a p value < 0.05. None of these genes have an adjusted p value < 0.05. Additional File 1 cites the following references: 2, 24, 25 [file 12711_2023_789_MOESM1_ESM.pdf]

Whole-genome sequencing of cryopreserved resources from French Large White pigs at two distinct sampling times reveals strong signatures of convergent and divergent selection between the dam and sire lines.
Additional Text

Additional text

Text S1: Sampling

Recent animals were chosen for sequencing using the following criteria. (1) No missing ascendant and no ascendant born outside France in the five previous generations was allowed. (2) The expected genetic contribution of ancestors born outside France was limited as much as possible based on the pedigree in the 20 previous generations. A penalty score $S = \sum_{n=1}^{20} 2^{20-n} N_{imp}(n)$ was computed for each boar, where $N_{imp}(n)$ was the number of imported ancestors at generation n in the pedigree of the boar. Boars were then ranked according to S and animals with the lowest values of this score were chosen for DNA extraction. (3) Poor DNA quality samples were discarded. (4) Some final trade-offs were made to limit the number of common ascendants in the last five generations between the chosen animals. At the end of this selection procedure, most of the recent boars (all LWD boars and six LWS boars) had semen in the National Cryobank, and may thus be used in follow-up studies or applications (see Discussion for further details).

No frozen semen was used specifically for this study so as to preserve the reproductive potential of the cryobank: DNA was extracted from blood, except for eight boars from 1977 whose DNA had been extracted from frozen semen (pellets) many years ago. A standard DNA extraction protocol was used for blood samples [48]. For semen samples, DTT and proteinase K were included in the lysis buffer and phenol chloroform extraction was performed.

Text S2: SNP calling

Read quality was checked using FastQC v0.11.2 and was found to be sufficient for all of them. All reads were mapped on the Sus scrofa genome version 11.1, retrieved from Ensembl, using bwa mem v0.7.15 with default parameters (except for option "-M": mark shorter split hits as secondary) and one read group per animal. Resulting bam files were sorted with samtools v1.3.1 and filtered using Markduplicate from Picard tools v2.1.1. Final bam files were obtained after local read realignment and base recalibration using GATK v3.7.

Genetic variants (both SNPs and indels) were called from these bam files using three different callers: (i) samtools v1.3.1 Mpileup with adjusted mapping quality "-C 50" and a minimal mapping quality "-q 30"; BCF files resulting from this command (one per sample) were combined into a single VCF file using bcftools v1.3.1, (ii) Freebayes v1.1.0 with a minimal mapping quality "-m 30" and a minimal polymorphism probability per site "-P 0.001") and (iii) GATK v3.7 HaplotypeCaller with a minimum mapping quality and a standard minimum confidence threshold for emitting both set at 30. Default values of these three software packages were used for all parameters other than those specified above. Two sets of variants were then defined. The first set (denoted HQSNP for high quality SNPs) included only autosomal bi-allelic SNPs found by all three callers and passing the following GATK quality filters: QD $>$ 2.0, FS $<$ 10.0, MQ $>$ 58.0, -2 $<$ MQRankSum $<$ 2, -2 $<$ ReadPosRankSum $<$ 2. These filters were adjusted according to GATK recommendations [49] by plotting the distribution of the corresponding quality scores (QD, FS, etc.) for SNPs found by all 3 callers and setting thresholds allowing rejection of only outlier values. Next, genotype calls with quality (GQ field) below 20 were considered missing and SNPs with a call rate below 90% were further removed from this set. The second set (denoted AV for autosomal variants) included all autosomal SNPs or indels called by at least one caller. Genotype calls with below 10 quality were considered missing but no variant filtering (quality scores or call rate) was applied. HQSNP was used for all general genome-wide analyses, while AV was used for a detailed investigation of candidate regions.

Text S3: Local score approach

In order to take advantage of linkage disequilibrium information in this temporal analysis, genomic regions with a local excess of low p values (i.e. candidate regions under selection) were detected using the local score (LS) approach proposed in [25], which defines a score function at each SNP and detects regions with a high cumulated score. The score function is equal to $-log10(pvalue) - \epsilon$, which implies that only p values lower than $10^{-\epsilon}$ contribute to increase the cumulated score. The default value $epsilon = 1$, recommended by the authors to optimize the detection power, led to a very high number of candidate regions in our dataset. We therefore used the more stringent epsilon=3 value. As the distribution of p values obtained from the method of [24] was close to uniform (Figure S6), the significance threshold of the local score for each chromosome could be evaluated using the closed-form formula provided in equation (3) of [25] and implemented in the R code available at [50]. These significance thresholds were computed for a chromosome-wide false positive rate (FPR) of 1%.

Text S4: Identification of causal variants under selection

Methods

Candidate causal variants within each candidate region were sought using two different strategies. The first one, which we refer to as the 'statistical approach', was based only on statistical criteria and did not use functional information. First, variants with more than 20% missing data or with allele frequency patterns inconsistent with the region category were removed. More precisely, this second criterion means that for regions considered under convergent (resp. divergent) selection in the two lines, we expect selection to favor the same allele (resp. opposite alleles) in

the two lines at the causal variant. This can be assessed by the sign of the selective advantage estimated in each line by the temporal approach, which can be either positive in the two lines, negative in the lines, or positive in one line and negative in the other. Variants passing these first filters were ranked according to the most relevant statistic for the region category and the top 1% were kept (within the limits of at least one and at most 10 per region). The relevant statistic was time-LWD for LWD or conv(LWD) regions and time-LWS for LWS or conv(LWS) regions. For conv regions, time-LWD and time-LWS were both relevant, so the 0.5% top variants of each statistic were selected. Similarly, for div regions the 0.5% top variants of both FLK and hapFLK were selected.

Very few of the variants selected by this first strategy were considered to be functional (i.e. with an at least MODERATE impact) by SnpEff. Thus, we also implemented a strategy with less stringent statistical criteria - the ten top SNPs of all four statistics were kept for each region - but selected only the functional variants (in the sense defined above) provided by this first filter. Note that the top signals of different statistics in a region may overlap, so this strategy generally selected less than 40 candidates per region (before the functional filter). We refer to this strategy as the 'functional' approach.

Results

When applying the 'statistical' approach to the *IGF2 region*, one intergenic variant at position 1,459,935 bp clearly stood out as the best candidate, while six other variants located from 1,499,012 to 1,521,688bp, including one synonymous variant of the *TH* (tyrosine hydroxylase) gene classified as functional, were also selected.

Only three of the candidate variants detected over all regions with the 'statistical' approach were considered to be potentially functional by SnpEff, including the synonymous variant in *TH* mentioned above (LOW impact). The two others were a synonymous variant (LOW impact) in the *EPG5* (ectopic P-granules autophagy protein 5 homolog) gene, included in the SSC1:95 region and a start codon gain variant (LOW impact) in the 5' UTR region of gene *RPL22L1* (ribosomal protein L22 like 1), included in the SSC13:109 region.

Eight additional functional candidate variants were detected using the 'functional' approach, four of which were located in the *IGF2* region already mentioned above. The four remaining variants were a start codon gain variant (LOW impact) in the *KATNA1* gene (SSC1:16 region), a missense (MODERATE impact) and a synonymous (LOW impact) variant in the *PTH* gene (SSC2:45 region) and a synonymous variant (LOW impact) in *VPS13D* (SSC6:72 region). While the variant in *KATNA1* (katanin catalytic subunit A1) was clearly not among the most promising candidates of its region, while the two variants in *PTH* (parathyroid hormone) and that in *VPS13D* (vacuolar protein sorting 13 homolog D) were potentially very interesting: their position was central in the region and their time-LWS value (the single test of interest in this region) was only slightly lower than that of the best candidate variants. Call rates in the two regions were generally quite high, suggesting an almost exhaustive representation of this region in our data.

Text S5: Private alleles

Due to the short evolution time from 1977 to each modern line, private alleles observed in LWD or LWS are unlikely to have come from new mutations occurring during this period. Indeed, assuming an evolution time of ten generations from the divergence of the lines to our modern samples, a per-base per-generation mutation rate of 1e-8 and a genome size of about 3e9 bp, the expected number of new mutations occurring in a sample of 13 animals is at most 780 (2*13*1e-8*3e9), which represents only 0.006% of the total number of HQSNPs. In comparison, 5.3% (resp. 4.3 %) of private alleles were observed in LWD (resp LWS), suggesting that these alleles existed in 1977 but were not observed in our sample due to an insufficient sample size or coverage. Similarly, among HQSNPs that were found to be polymorphic in both LWD and LWS, 4.1% were found to be monomorphic in 1977 in our data.

Text S6: Biological functions under selection

To confirm the observed differences between the biological functions under selection in the LWD and LWS lines (Fig. 5, Additional file 2, Figures S4 and S5), we performed two additional enrichment analyses based on two non-overlapping sets of selection signatures: those expected for Dam traits (region categories LWD, conv(LWD) or div) on one hand, and those expected for Both.Sire traits (region categories LWS, conv(LWS) or conv) on the other. For each of these two analyses, the three MGI, KEGG and GOBP databases were queried simultaneously and significant co-annotations were detected (Additional File 6, sheets 5 & 6). Bbiological functions related to early life survival or bone physiology were more significant in the analysis based on signatures expected for Dam traits. For instance, the 'perinatal lethality' term was found to be significant in the Dam analysis (sheet 5, lines 3, 4, 12 & 25) but not in the Both.Sire analysis and the lowest p value associated to the 'preweaning lethality' term was 1.9e-5 in the Dam analysis (sheet 5, line 11) versus 1.4e-4 in the Both.Sire analysis (sheet 6, line 60). In contrast, biological functions related to glycogen or lipid metabolism were only significant in the analysis based on signatures expected for Both.Sire traits. The word 'lipid' was found to be significant in the Both.Sire analysis (sheet 6, lines 48, 69, 98, 139, 148 & 170), as well as the words 'fatty acids' (line 51) and 'glycogen' (line 22), while all of these words were absent from the Dam analysis.

Text S7: Insight from a previous gene expression study

Methods

In a previous study, gene expression in muscle was evaluated in several French breeds using the Agilent 44Kv1 microarray [40]. Muscle samples from pigs of both Large White sire (n=10) and dam lines (n=41) were included, along with other samples from the Landrace (n=33), Pietrain (n=39), and Duroc (n=24) breeds. The animals were born between 2007 and 2009, in particular 49 out of the 51 Large White pigs were born in 2007. In our study, these data were used to identify possible differential expression between the LWD and LWS lines for the genes included in candidate regions under selection. Information on the expression of these genes (when available) was updated in November 2018 to account for the pig reference genome assembly Sscrofa11.1. Only 48 genes out of the 172 included in (or flanking) the regions under selection were present

on the 44Kv1 microarray and considered as expressed (Additional file 8). These 48 genes were represented by 76 probes (one to six probes per gene). A Wilcoxon test was applied to compare the expression of each probe between LWS and LWD. When several probes corresponded to the same gene, only the most significant one was reported; note that expression data for these probes were generally very positively correlated (Figure S7). All statistical analyses were performed with R version 4.1.0 (2020-10-10) with the corrplot_0.91 package to calculate Spearman correlations.

Results

Positive selection on a regulatory variant is expected to affect the expression of one or several genes in the associated candidate region. Among the 48 genes putatively under selection and for which expression data were available, twelve (*FGL2*, *PSMC2*, *NANS*, *EPDR1*, *TOP1*, *KATNA1*, *CAMK2A*, *SNTB1*, *FAM217B*, *MSR1*, *LPIN1*, *BMPR1B*) were found to be differentially expressed ($p < 0.05$) between LWD and LWS samples, but none of them were significant after multiple testing correction ($BH < 0.05$). Expression variations in these 12 genes are presented in Figure S8. All categories of selection signatures were represented among these 12 genes: conv (2 genes out of a total of 11 with expression data), conv(LWD) (1 out of 7 genes), conv(LWS) (2 out of 10 genes), LWD (1 out of 7 genes), LWS (2 out of 3 genes) and div (4 out of 10 genes). No enrichment of differentially expressed genes was detected in categories involving genetic differentiation between lines (LWD, LWS and div) when compared to the other categories (conv(LWD), conv(LWS) and conv) involving more genetic convergence (Fisher exact test, $p = 0.2$), or in the div category compared to all other classes ($p = 0.18$).

Text S8: Molecular mechanisms mediating selection

Several previous studies (e.g. [51, 2]) provided evidence that short-term adaptation (i.e. at the species level) is most likely driven by regulatory variants rather than protein-coding variants. Under this prediction, we would expect an enrichment of differentially expressed genes between the two lines in the candidate regions under divergent selection, compared to those from other categories. Indeed, allele frequency divergence at a regulatory variant implies differential expression of the regulated gene, which is located in the same genomic region if the variant is acting in cis. We tested this hypothesis based on the genes located in candidate regions under selection and whose expression levels in the muscle had been assessed for the two lines in a previous study [40], but did not observe such enrichment (see previous section).

However, the power of this enrichment test was limited by the following factors: (i) expression data were available for only 48 of the 172 genes located in or near candidate regions and were obtained through a microarray experiment providing a less exhaustive quantification than modern RNAseq approaches, (ii) the detection of differentially expressed genes was likely underpowered due to the small number of animals analyzed in LWS (n=10), (iii) expression data were measured on animals born in 2007 for most of them, which were thus less differentiated than those sequenced, (iv) expression data were measured in muscle and were thus not informative for genes differentially expressed only in other tissues, (v) genes with expression data may not be those under selection in a region, in particular for candidate regions including many genes, (vi) causal genes under convergent selection may still be differentially expressed between lines

due to trans regulatory or non genetic (epigenetic for instance) effects.

To gain further insight into the respective roles of structural versus regulatory mutations, we conducted a more in-depth analysis of the 10 regions detected under divergent selection between the two lines, particularly the four regions of this category that included at least one gene for which expression data were available from [40]. Two of these candidate regions included a single gene, *ARHGAP10* (SSC8:80) and *ZC3HAV1* (SSC18:10), and these two genes showed no differential expression between lines. This suggests that the causal variant under selection in these regions may affect the structure of the protein rather than its expression. No evidence of such mutation was found in the sequence data, with the most promising candidate variants for the two genes being located in introns, but this could be due to insufficient coverage in some parts of the region, at least for *ARHGAP10* (the call rate was found to be very high throughout the region of *ZC3HAV1*). Another hypothesis would be that these genes (or regions) include a regulatory mutation acting in trans on another gene.

In the two other candidate regions under divergent selection and for which gene expression data was available, the causal variant under selection was more likely regulatory. The first of these regions (SSC1:16) included only two genes, i.e. glycoprotein integral membrane 1 (*GINM1*) and katanin catalytic subunit A1 (*KATNA1*) and expression data was available for both. Significant differential expression between lines was observed for *KATNA1* and this gene also included the most promising candidate variants of the region, which were located in an intron (but other parts of *KATNA1* exhibited a rather low call rate). The second region (SSC2:151) included 10 genes and gene expression data was available for three of them, i.e. *CAMK2A*, *PDGFRB* and *TCOF1*. Significant differential expression between lines was observed for calcium/calmodulin cependent protein kinase II clpha (*CAMK2A*) and very high hapFLK values (the highest in the region) were observed throughout this gene. Several candidate causal variants were also located within this gene or close to it, although the highest FLK value in the region was located in an intron of platelet derived growth factor receptor beta (*PDGFRB*).

Finally, note that five of the six other regions found under divergent selection included no known protein or RNA gene. While this could be partly explained by false positive signals (the FDR of the hapFLK analysis was 15%) or imperfect annotation of the current pig genome assembly, this also suggests that many of the causal variants could be involved in cis or trans regulatory effects. For two of these five regions (SSC4:18 and SSC17:60), one of the genes flanking the region (*SNTB1* and *FAM217B*, respectively) was found to be differentially expressed between LWS and LWD, which would actually suggest cis-regulation.

Overall, this analysis of the 10 div regions suggested that the causal variant under selection was possibly protein-coding in only two of them and was more likely regulatory in the others (cis-regulatory in four regions and trans-regulatory in four regions).

Text S9: Candidate genes under selection

In several of the regions found under recent selection in this study, we identified one or a few candidate genes based on statistical criteria (number of genes in the region, local peak of the relevant test statistic, external information on gene expression), whose function was also consistent with selection objectives in LW (Additional file 2, Table S1). While these genes were often related to the key biological terms listed in Table 3 and discussed in the main manuscript,

a few other interesting candidate genes are discussed below.

PKP1 (plakophilin 1), associated with the 'birth weight' term in our functional enrichment analysis, was the only gene found in the conv (LWS) candidate region SSC10:23 and included two promising candidate variants, located in introns. Four regulatory variants in this gene showed very high association with breast muscle thickness in ducks [52], which might be related to possible selection for body weight in LW pigs.

BMPR1B (bone morphogenetic protein receptor type 1B) is the only gene in the LWS SSC8:124 region and is also differentially expressed between lines. This gene is involved in the development of several tissues [53, 54]. It has also been associated with reproduction [55], i.e. more precisely with prolificacy in sheep [56] and pigs [57]. The signature observed on this gene could thus be related to the selection pressure on prolificacy applied in LW from the mid-1980s.

SEMA3E (semaphorin 3E) is located in a local peak of time-LWD in the conv(LWD) SSC9:96 region. It is involved in several developmental processes (angiogenesis, nervous system development, etc.). Rare variants in *SEMA3E* were associated with human obesity [58] and one SNP was associated with backfat thickness in Italian LW pigs [59]. Selection on this gene in French LW pigs might thus be related to the high value placed on backfat thickness in the breeding objective of both the LWD and LWS lines since 1977.

ZC3HAV1, also known as ZAP, was the only candidate gene in the div SSC18:10 region. This gene is a CCCH-type zinc finger antiviral protein that can inhibit the replication of different types of viruses, for instance its expression might impact the ability of pigs to overcome PRSS infection [60]. Expression of *ZC3HAV1* in muscle was not found to be different between LWD and LWS, but a different result might be obtained in tissues more involved in the antiviral response.

One other region (SSC1:110, div) included six genes with no obvious candidate based on genetic or expression data, but one of these genes (*RORA*, RAR related orphan receptor A) is an interesting functional candidate in the context of early life survival, i.e. a biological function found under selection in this study. Indeed, it is a regulator of embryonic development which has been identified as involved in oocyte maturation in pigs [61].

Additional figures

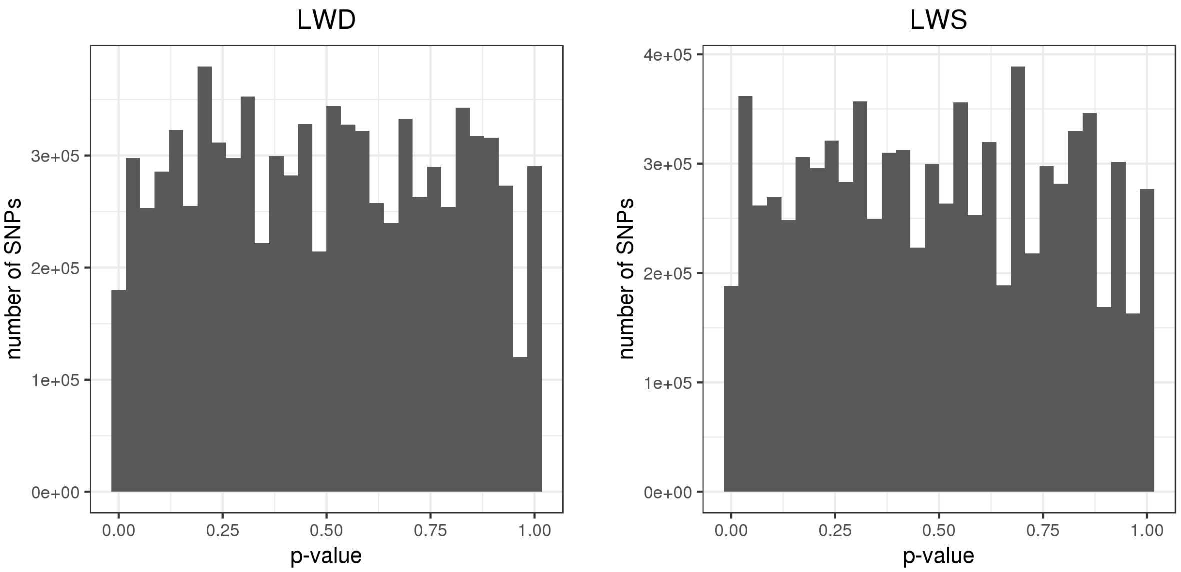

Figure S6: **Distribution of p values obtained by the time-LWD and time-LWS tests.** High quality SNPs (HQSNP set) with MAF greater than 0.1 were considered.

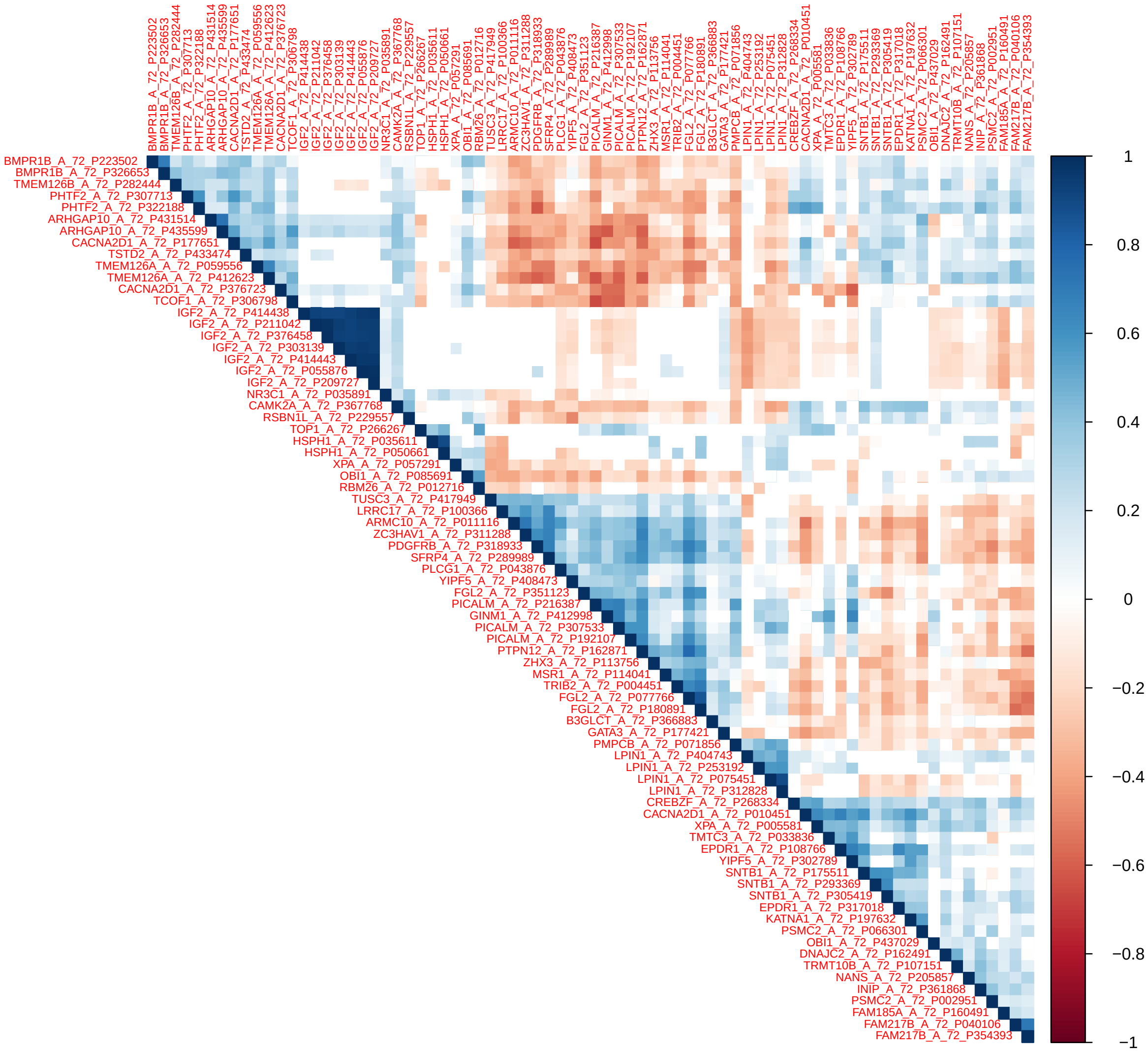

Figure S7: **Correlation of expression levels in the muscle measured in San Cristobal *et al* (2015) for 76 probes corresponding to 48 genes found under selection in our study.** Correlation between two probes was computed based on normalized expression for Large White sire (n=10) and dam lines (n=41) at these two probes. The ordering of probes on the graph follows from a hierarchical clustering of the correlation matrix. Only significant correlation (p value < 0.05) are shown.

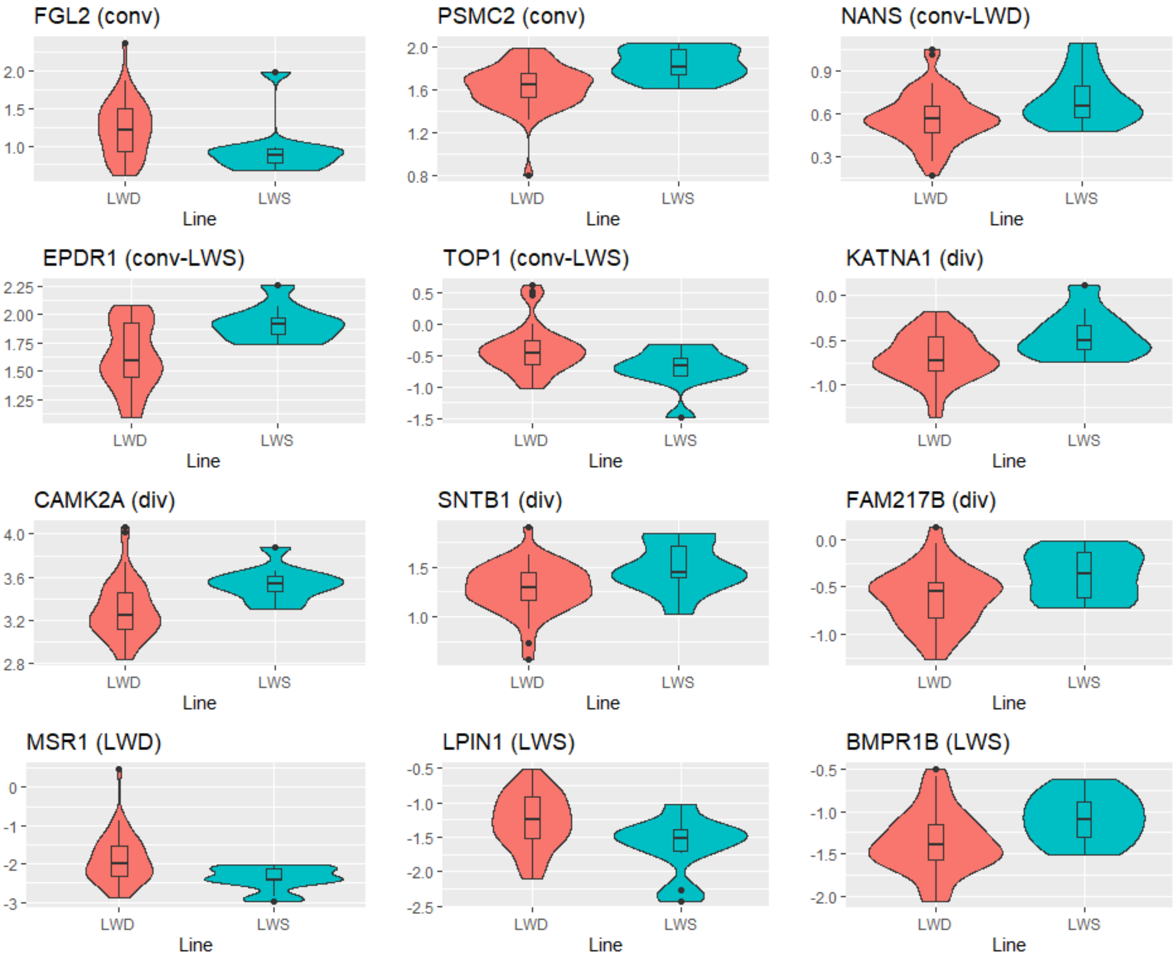

Figure S8: **Boxplot of muscle expression levels in LWD and LWS for the 12 genes found differentially expressed between the two lines.** All genes are differentially regulated between the two lines with a p value < 0.05. None of these genes have an adjusted p value < 0.05.

References

[2] Boitard S, Boussaha M, Capitan A, Rocha D, Servin B. Uncovering adaptation from sequence data: lessons from genome resequencing of four cattle breeds. Genetics. 2016;203:433–450.

[24] Paris C, Servin B, Boitard S. Inference of selection from genetic time series using various parametric approximations to the Wright-Fisher model. G3: Genes, Genomes, Genetics. 2019;9:4073–4086.

[25] Fariello MI, Boitard S, Mercier S, Robelin D, Faraut T, Arnould C, et al. Accounting for linkage disequilibrium in genome scans for selection without individual genotypes: the local score approach. Molecular ecology. 2017;26:3700–3714.

[40] SanCristobal M, Rohart F, Lascor C, Bouffaud M, Trouilh L, Martin PG, et al. Exploring transcriptomic diversity in muscle revealed that cellular signaling pathways mainly differentiate five Western porcine breeds. BMC genomics. 2015;16:1055.

[48] Montgomery G, Sise J. Extraction of DNA from sheep white blood cells. New Zealand Journal of Agricultural Research. 1990;33:437–441.

[49] GATK Hard-filtering germline short variants;. `https://gatk.broadinstitute.org/hc/en-us/articles/360035890471`. Accessed 02 February 2022.

[50] Local score;. `https://forge-dga.jouy.inra.fr/projects/local-score`. Accessed 02 February 2022.

[51] Grossman S, Andersen K, Shlyakhter I, Tabrizi S, Winnicki S, Yen A, et al. Identifying recent adaptations in large-scale genomic data. Cell. 2013;152:703–713.

[52] Deng MT, Zhang F, Zhu F, Yang YZ, Yang FX, Hao JP, et al. Genome-wide association study reveals novel loci associated with fat-deposition and meat-quality traits in Pekin ducks. Animal Genetics. 2020;51:953–957.

[53] Caronia G, Wilcoxon J, Feldman P, Grove EA. Bone morphogenetic protein signaling in the developing telencephalon controls formation of the hippocampal dentate gyrus and modifies fear-related behavior. Journal of Neuroscience. 2010;30:6291–6301.

[54] Katagiri T, Watabe T. Bone morphogenetic proteins. Cold Spring Harbor Perspectives in Biology. 2016;8:a021899.

[55] Fabre S, Pierre A, Mulsant P, Bodin L, Di Pasquale E, Persani L, et al. Regulation of ovulation rate in mammals: contribution of sheep genetic models. Reproductive Biology and Endocrinology. 2006;4:20.

[56] Paradis F, Novak S, Murdoch GK, Dyck MK, Dixon WT, Foxcroft GR, et al. Temporal regulation of BMP2, BMP6, BMP15, GDF9, BMPR1A, BMPR1B, BMPR2 and TGFBR1 mRNA expression in the oocyte, granulosa and theca cells of developing preovulatory follicles in the pig. Reproduction. 2009;138:115.

[57] Li WT, Zhang MM, Li QG, Tang H, Zhang LF, Wang KJ, et al. Whole-genome resequencing reveals candidate mutations for pig prolificacy. Proceedings of the Royal Society B: Biological Sciences. 2017;284:20172437.

[58] Van Der Klaauw AA, Croizier S, De Oliveira EM, Stadler LK, Park S, Kong Y, et al. Human semaphorin 3 variants link melanocortin circuit development and energy balance. Cell. 2019;176:729–742.e18.

[59] Fontanesi L, Schiavo G, Galimberti G, Calò DG, Scotti E, Martelli PL, et al. A genome wide association study for backfat thickness in Italian Large White pigs highlights new regions affecting fat deposition including neuronal genes. BMC genomics. 2012;13:583.

[60] Zhao Y, Song Z, Bai J, Liu X, Nauwynck H, Jiang P. ZAP, a CCCH-type zinc finger protein, inhibits porcine reproductive and respiratory syndrome virus replication and interacts with viral Nsp9. Journal of virology. 2019;93:e00001–19.

[61] Brazert M, Kranc W, Nawrocki MJ, Sujka-Kordowska P, Konwerska A, Jankowski M, et al. New markers for regulation of transcription and macromolecule metabolic process in porcine oocytes during in vitro maturation. Molecular medicine reports. 2020;21:1537–1551.
