# Supplementary material for: Whole-genome sequencing of cryopreserved resources from French Large White pigs at two distinct sampling times reveals strong signatures of convergent and divergent selection between the dam and sire lines
Source: Genet Sel Evol. 2023 Mar 2;55:13. doi: 10.1186/s12711-023-00789-z (PMC9979506; doi:10.1186/s12711-023-00789-z)
Supplement: Supplementary file 2 — Additional file 2: Figure S1. MDS analysis of the genetic diversity observed in French LW pigs. High-quality SNPs (HQSNP set) with a MAF greater than 0.1 were considered. Figure S2. Summary of the genome-wide scan for recent selection in French LW pigs, chromosomes 1 to 9. Each panel of the figure shows the results of the three tests for selection for a different autosome. P values of the time-LWD (blue), time-LWS (green) and hapFLK (red) tests are plotted in log10 scale (y axis) according to genomic position (x axis). Opposite signs are used for time-LWD and time-LWS on the one hand and hapFLK on the other hand, i.e. low p values correspond to highly negative points for time-LWD and time-LWS and to highly positive points for hapFLK. Significant p values are shown in darker color for the three tests. For time-LWD and time-LWS, SNPs ranked as significant by the local score approach (see “Methods” for further details) may be hidden by non significant SNPs with higher individual p values, so significant regions for these tests were also highlighted by small triangle marks below the p value plot. Figure S3. Summary of the genome-wide scan for recent selection in French LW pigs, chromosomes 10 to 18. See Figure S2 for details. Figure S4. Top 20 significant GO terms for Biological Processes and association between these terms and groups of selection signature categories. See Fig. 5 for details. Figure S5. Top 20 significant KEGG terms and association between these terms and groups of selection signature categories. See Fig. 5 for details. Table S1. Determination of candidate genes in several selection signatures. This table reports all the genes that (i) can be considered as good candidate genes within their region (based on criteria described below) and (ii) are associated to one of the enriched biological functions listed in Table 3 (or more generally whose biological function is consistent with selection objectives in LW). For a given region of interest, genes that rep [file 12711_2023_789_MOESM2_ESM.pdf]

Whole-genome sequencing of cryopreserved resources from French Large White pigs at two distinct sampling times reveals strong signatures of convergent and divergent selection between the dam and sire lines.

Additional Figures and Tables

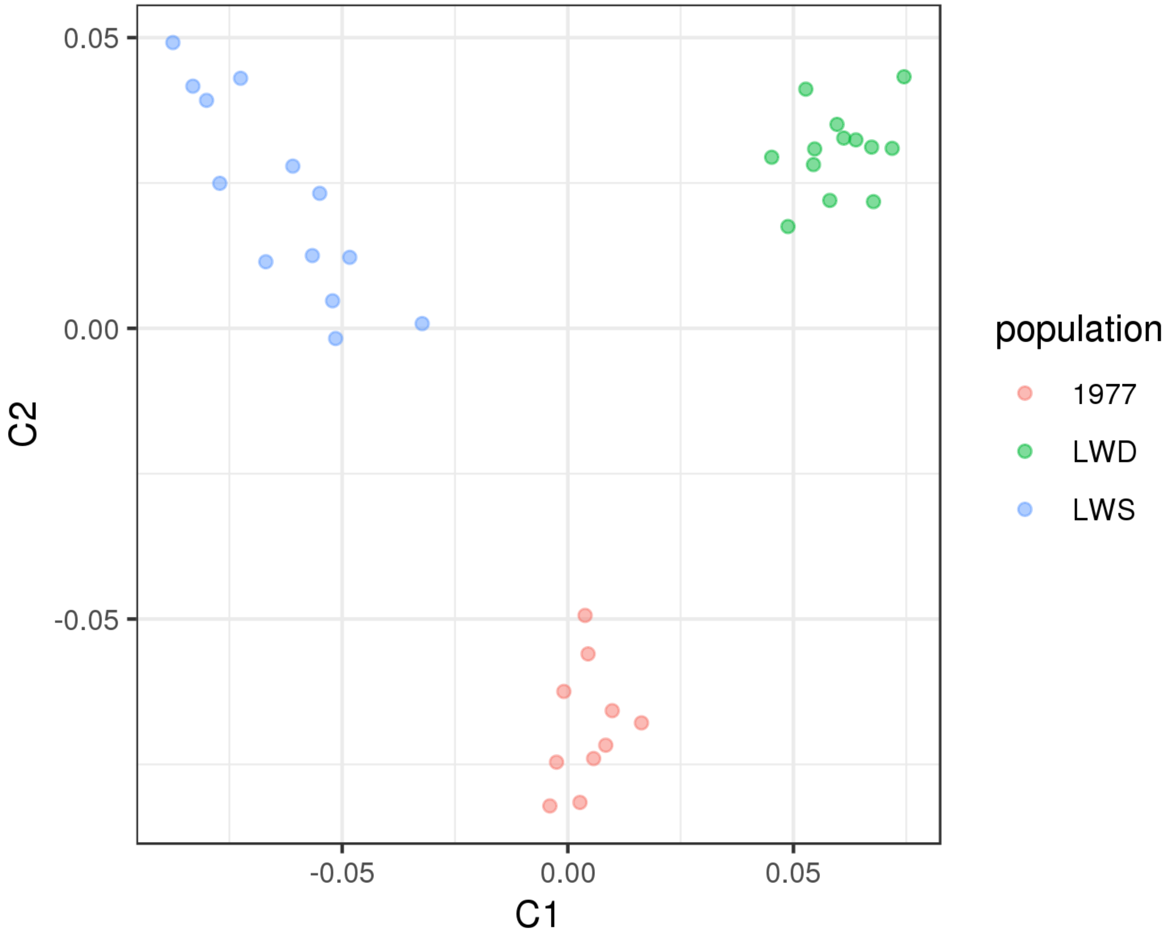

Figure S1: **MDS analysis of the genetic diversity observed in French LW pigs.** High quality SNPs (HQSNP set) with MAF greater than 0.1 were considered.

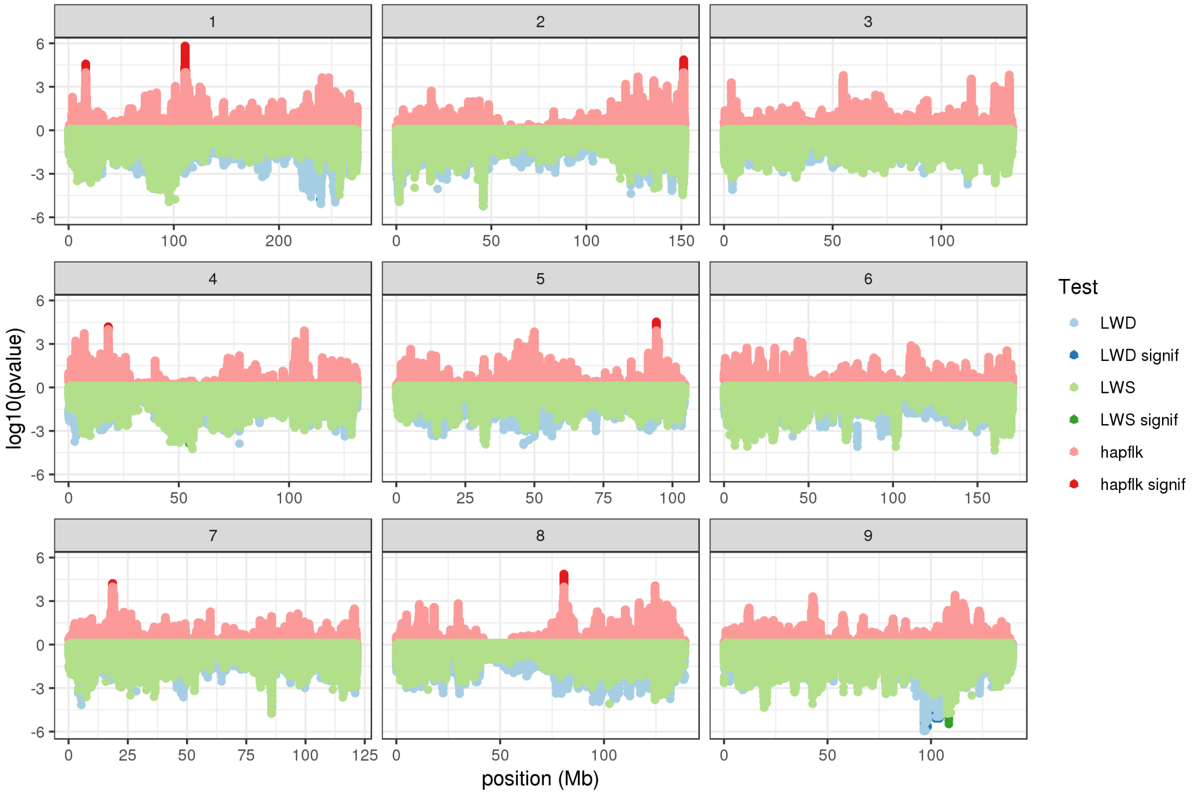

Figure S2: **Summary of the genome-wide scan for recent selection in French LW pigs, chromosomes 1 to 9.** Each panel of the figure shows the results of the three tests for selection for a different autosome. P values of the time-LWD (blue), time-LWS (green) and hapFLK (red) tests are plotted in log10 scale (y axis) as a function of the genomic position (x axis). Opposite signs are used for time-LWD and time-LWS on the one hand and hapFLK on the other, *i.e.* low p values correspond to highly negative points for time-LWD and time-LWS and to highly positive points for hapFLK. Significant p values are shown in darker color for the three tests. For time-LWD and time-LWS, SNPs ranked as significant by the local score approach (see Methods for further details) may be hidden by non significant SNPs with higher individual p values, so significant regions for these tests were also highlighted by small triangle marks below the p value plot.

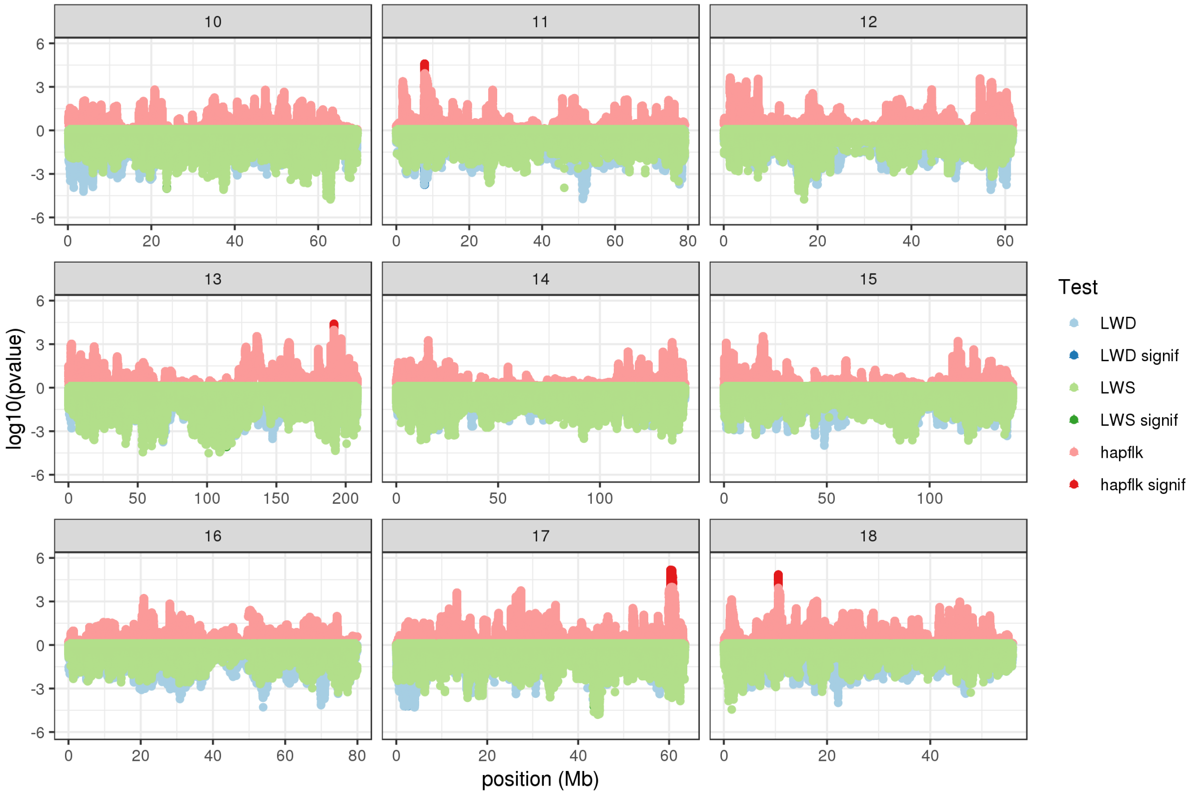

Figure S3: **Summary of the genome-wide scan for recent selection in French LW pigs, chromosomes 10 to 18.** See Figure S2 for details.

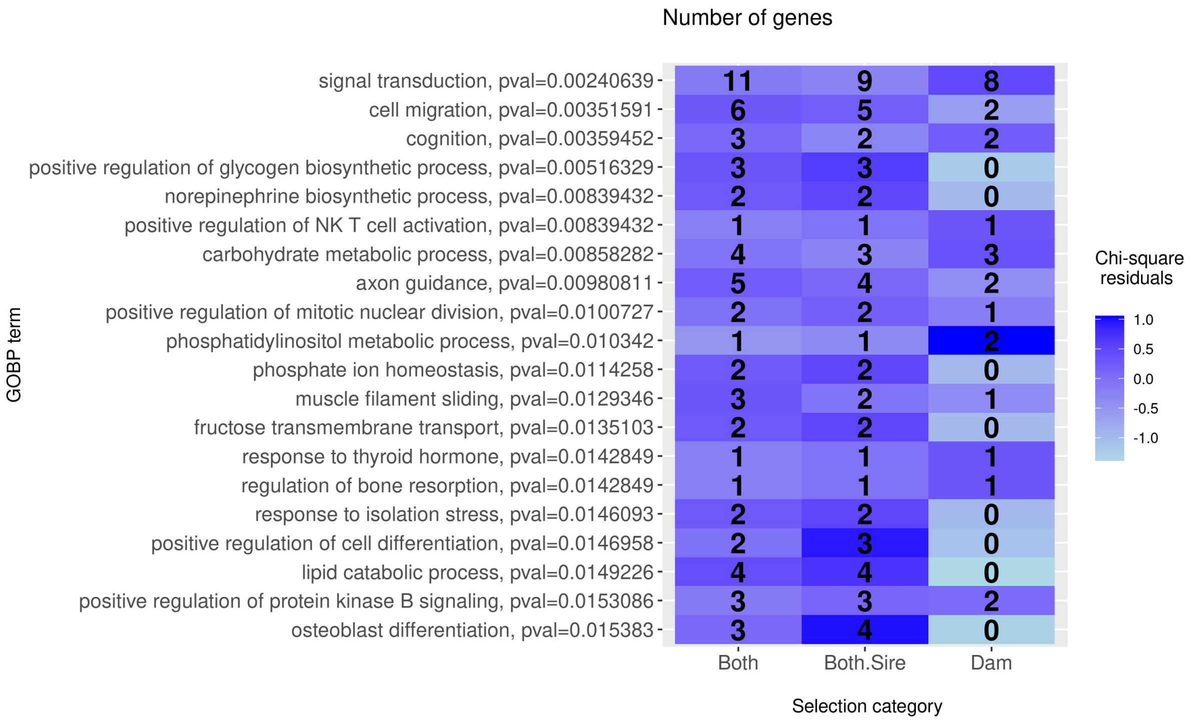

Figure S4: **Top 20 significant GO terms for Biological Processes and association between these terms and groups of selection signature categories.** See Fig. 5 for details.

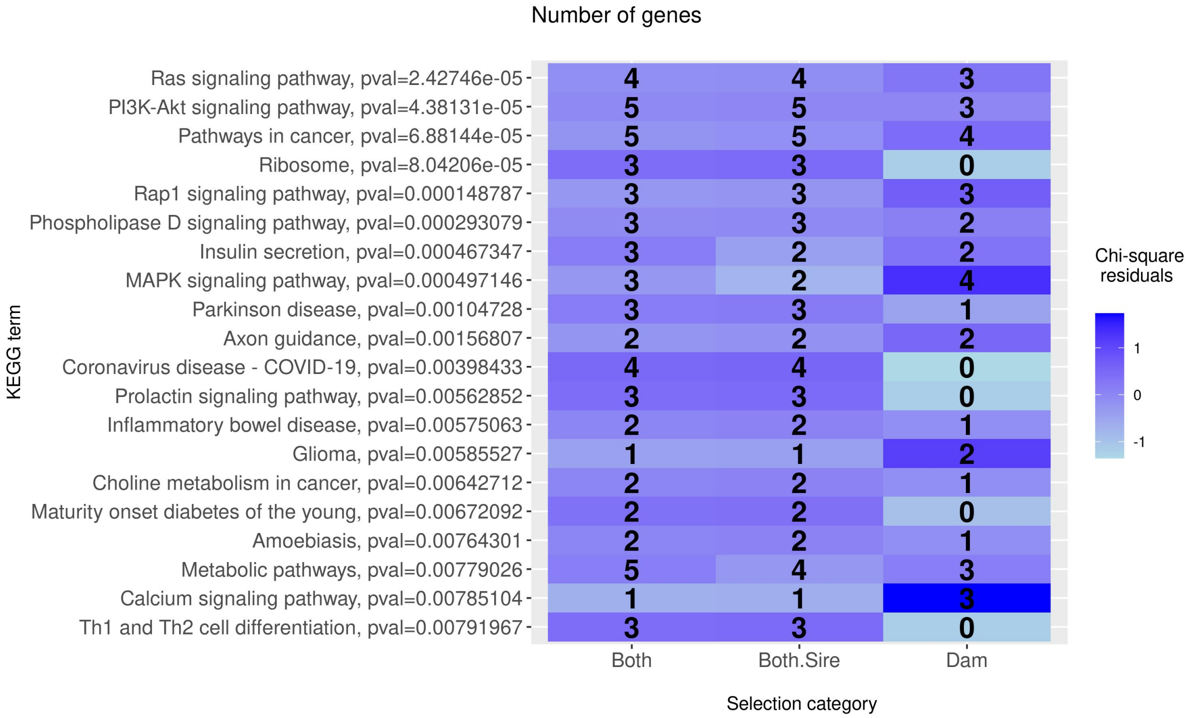

Figure S5: **Top 20 significant KEGG terms and association between these terms and groups of selection signature categories.** See Fig. 5 for details.

Region	Category	Nb. Genes	Gene ID	Local peak	Diff. expr.	Causal variant?	Overall evidence
SSC1:16	div	2	KATNA1	yes	yes		strong
SSC1:95	conv	3	EPG5	yes	no data	candidate	strong
SSC1:230	LWD	1	GNA14	NA	no	candidate	strong
SSC2:1	conv	12	IGF2	yes	no	known	strong
SSC2:45	conv(LWS)	1	PTH	yes	no	candidate	strong
SSC2:145	conv(LWD)	0	NR3C1	NA	no	candidate	medium
SSC2:151	div	10	CAMK2A	yes	yes		strong
SSC2:151	div	10	PDGFRB	yes	no		medium
SSC2:151	div	10	SLC6A7	yes	no data		medium
SSC3:124	LWS	0	LPIN1	NA	yes	candidate	strong
SSC5:94	div	0	KITLG	NA	no data		medium
SSC5:94	div	0	TMTC3	NA	no		medium
SSC8:80	div	1	ARHGAP10	NA	no		strong
SSC8:124	LWS	1	BMPR1B	NA	yes		strong
SSC9:96	conv(LWD)	5	SEMA3E	yes	no data		strong
SSC9:101	conv	26	RELN	yes	no data		medium
SSC9:101	conv	26	MAGI2	yes	no data		medium
SSC9:107	conv(LWS)	8	NRCAM	yes	no data		medium
SSC9:107	conv(LWS)	8	PNPLA8	yes	no data		medium
SSC9:107	conv(LWS)	8	SFRP4	yes	no		medium
SSC10:23	conv(LWS)	1	PKP1	NA	no data	candidate	strong
SSC10:62	conv(LWS)	0	GATA3	NA	no		medium
SSC11:7	LWD	3	B3GLCT	yes	no		medium
SSC11:51	LWD	0	RBM26	NA	no		medium
SSC13:109	conv	9	TNIK	yes	no data		medium
SSC17:43	conv(LWS)	3	TOP1	yes	yes		strong
SSC17:43	conv(LWS)	3	PLCG1	yes	no		medium
SSC18:10	div	1	ZC3HAV1	NA	no		medium

Table S1: **Determination of candidate genes in several selection signatures.** This table reports all the genes that (i) can be considered as good candidate genes within their region (based on criteria described below) and (ii) are associated to one of the enriched biological functions listed in Table 3 (or more generally whose biological function is consistent with selection objectives in LW). For a given region of interest, genes that represent even better candidates than those selected based on condition (ii) are also included. Column 'Nb. genes' gives the number of genes in a region, and column 'Gene ID' lists the candidate genes. Column 'Local peak' indicates whether the most relevant test statistic for the region shows a local peak within or close to the gene (see Additional file 4 for summary plots of the four test statistics in all candidate regions); this column takes value NA when no or a single gene is observed in a region. Column 'Diff. expr.' indicates whether the gene was found differentially expressed between LWS and LWD in the study of San Cristobal *et al* (2015); this column takes value 'no data' for genes that were not included in this study. Column 'Causal variant' indicates whether a causal variant under selection is known for this gene, or if a small number of potential candidate variants have been identified in our study (these candidates may be functional or not, see the text for more details). Based on all these observations, column 'Overall evidence' quantifies the evidence for each candidate gene to be the one under selection in a given region.
