# Supplementary figures and images for: Whole-genome sequencing of cryopreserved resources from French Large White pigs at two distinct sampling times reveals strong signatures of convergent and divergent selection between the dam and sire lines (part 1 of 2)
Source: Genet Sel Evol. 2023 Mar 2;55:13. doi: 10.1186/s12711-023-00789-z (PMC9979506; doi:10.1186/s12711-023-00789-z)

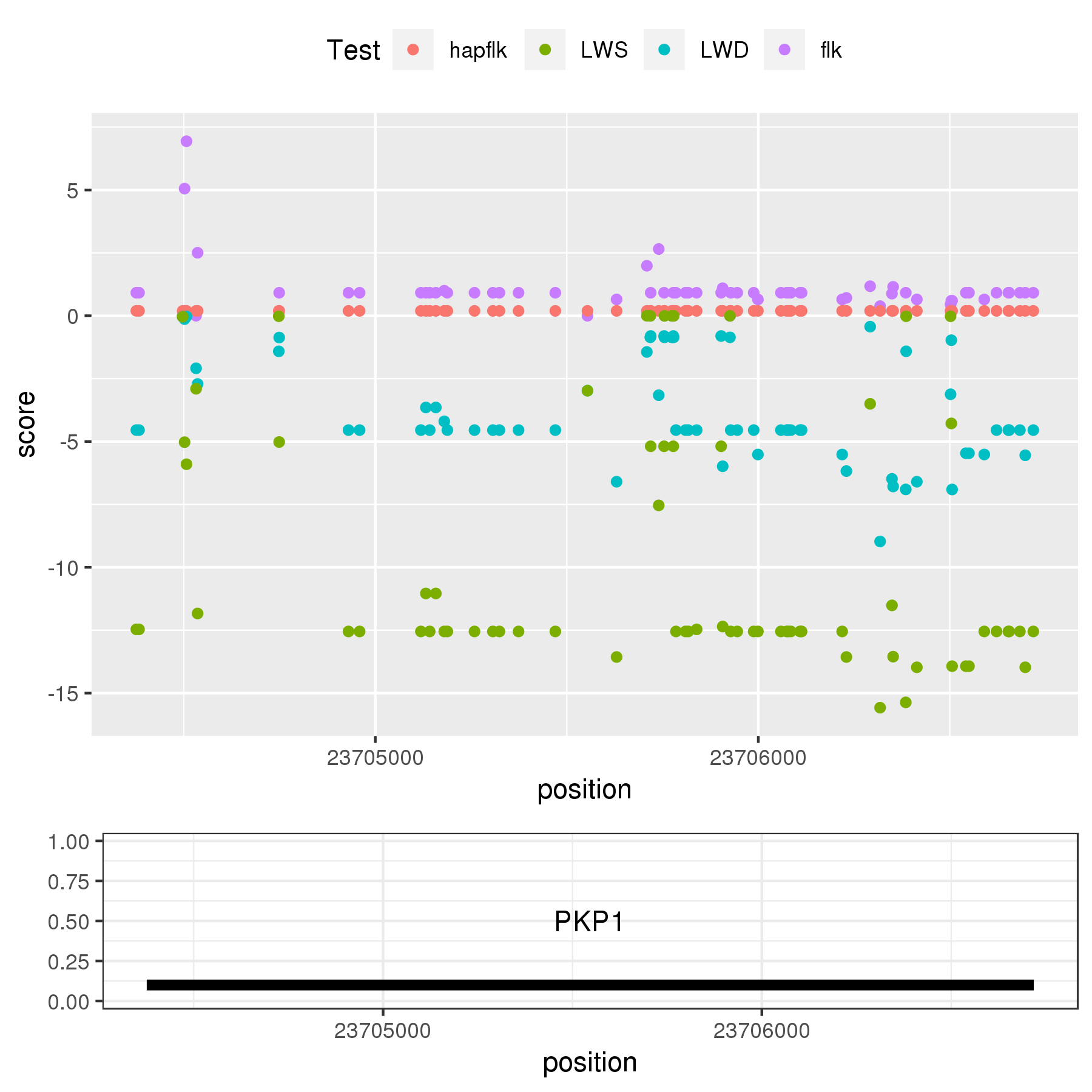

Supplement: Supplementary file 4 — Additional file 4. Summary plots of the scan for selection for all candidate regions of Table 1. Each file of this archive corresponds to a different candidate region, whose genomic position can be read in the file name. The top panel shows the results of the four tests for selection applied in the region. Values of the time-LWD (in blue), time-LWS (in green), hapFLK (in red) and FLK (in purple) tests are plotted (y axis) according to genomic position (x axis). Opposite signs are used for time-LWD and time-LWS on the one hand and hapFLK and FLK on the other hand, i.e. high test statistic values correspond to highly negative points for time-LWD and time-LWS and to highly positive points for hapFLK and FLK. All variants (AV set) were considered for these analyses. The bottom panel shows the position and name of genes located in the region. [file 12711_2023_789_MOESM4_ESM.xz › stat_profiles/all10_auto_chro10_23704376-23706718_scores.png]

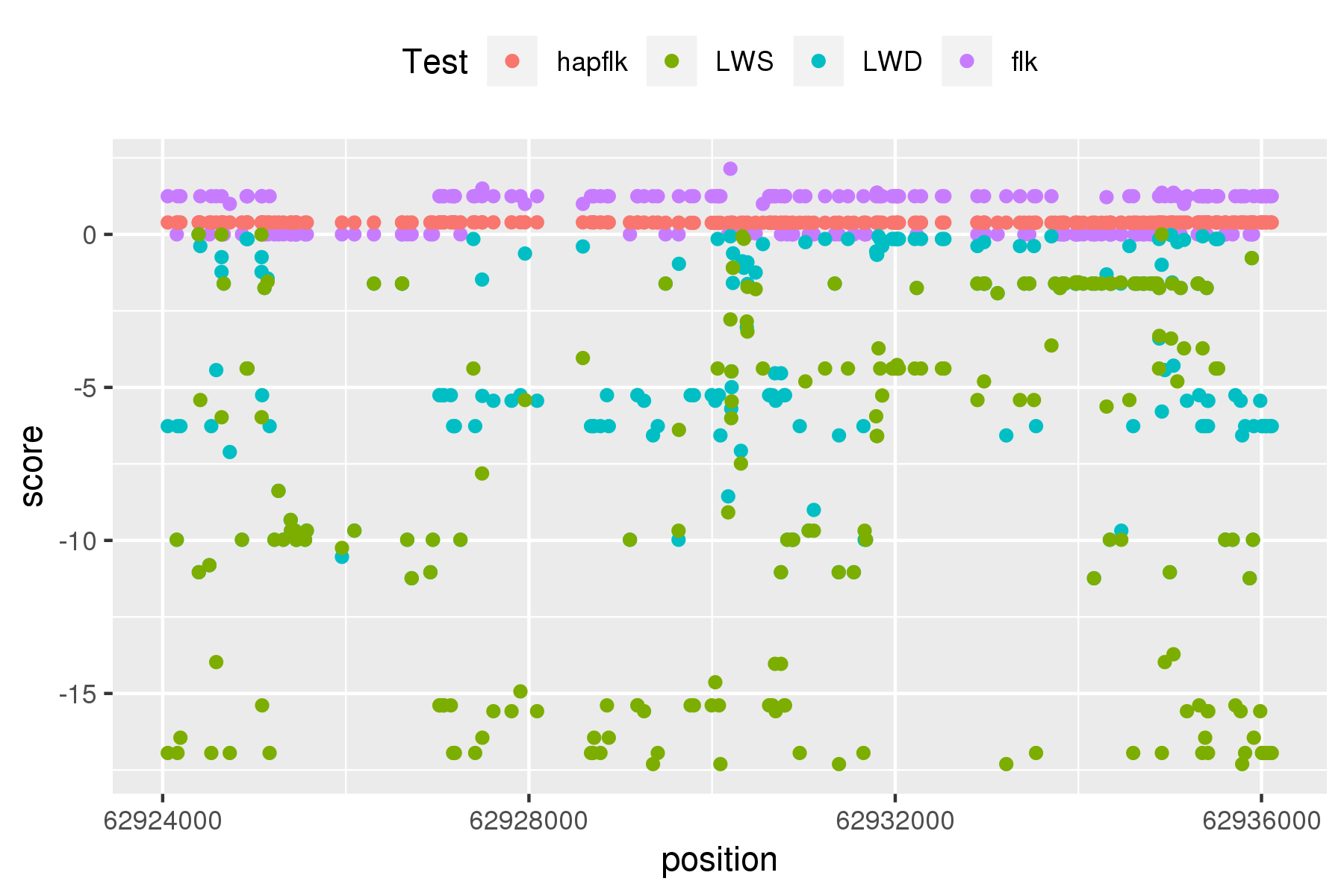

Supplement: Supplementary file 4 — Additional file 4. Summary plots of the scan for selection for all candidate regions of Table 1. Each file of this archive corresponds to a different candidate region, whose genomic position can be read in the file name. The top panel shows the results of the four tests for selection applied in the region. Values of the time-LWD (in blue), time-LWS (in green), hapFLK (in red) and FLK (in purple) tests are plotted (y axis) according to genomic position (x axis). Opposite signs are used for time-LWD and time-LWS on the one hand and hapFLK and FLK on the other hand, i.e. high test statistic values correspond to highly negative points for time-LWD and time-LWS and to highly positive points for hapFLK and FLK. All variants (AV set) were considered for these analyses. The bottom panel shows the position and name of genes located in the region. [file 12711_2023_789_MOESM4_ESM.xz › stat_profiles/all10_auto_chro10_62924056-62936112_scores.png]

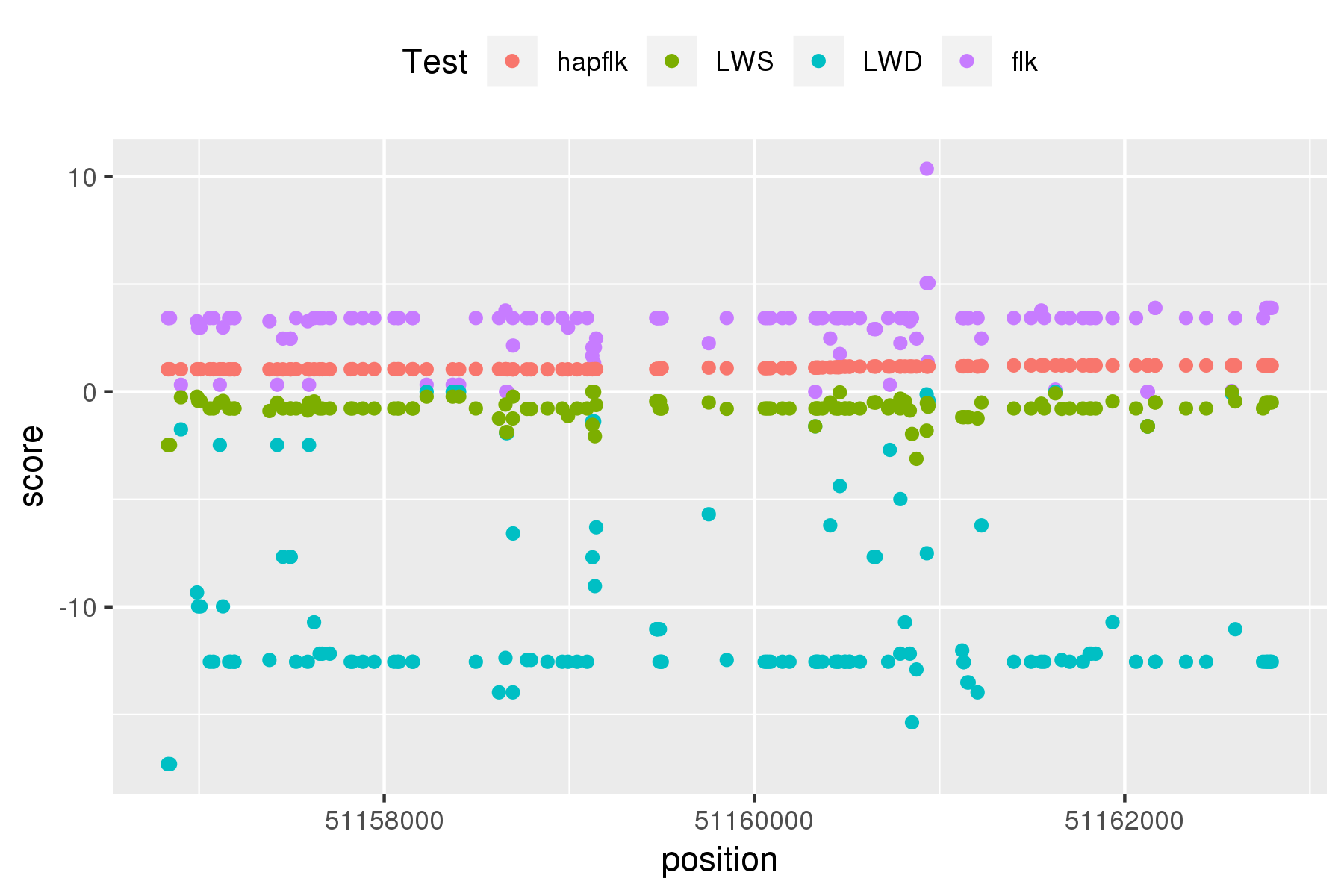

Supplement: Supplementary file 4 — Additional file 4. Summary plots of the scan for selection for all candidate regions of Table 1. Each file of this archive corresponds to a different candidate region, whose genomic position can be read in the file name. The top panel shows the results of the four tests for selection applied in the region. Values of the time-LWD (in blue), time-LWS (in green), hapFLK (in red) and FLK (in purple) tests are plotted (y axis) according to genomic position (x axis). Opposite signs are used for time-LWD and time-LWS on the one hand and hapFLK and FLK on the other hand, i.e. high test statistic values correspond to highly negative points for time-LWD and time-LWS and to highly positive points for hapFLK and FLK. All variants (AV set) were considered for these analyses. The bottom panel shows the position and name of genes located in the region. [file 12711_2023_789_MOESM4_ESM.xz › stat_profiles/all10_auto_chro11_51156831-51162794_scores.png]

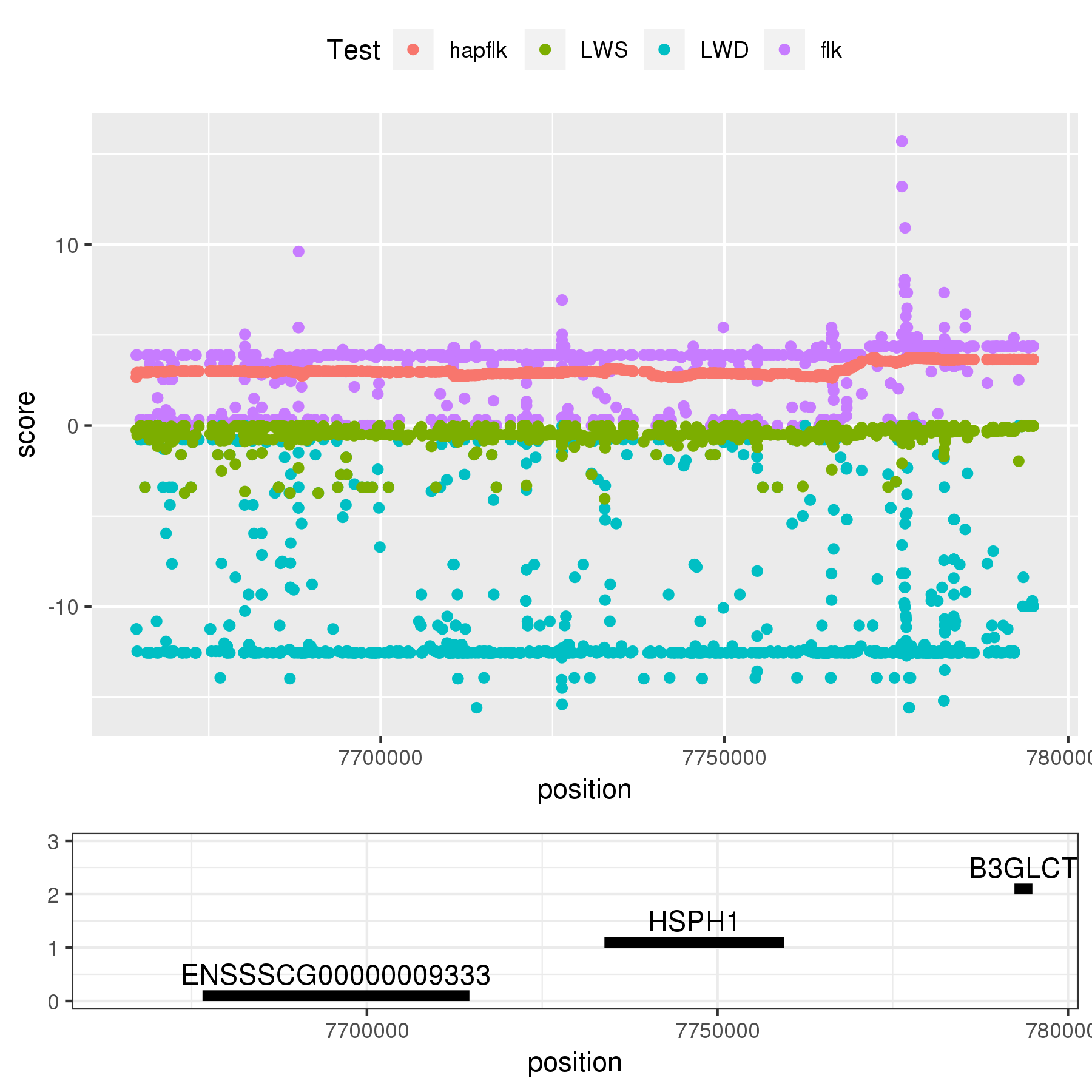

Supplement: Supplementary file 4 — Additional file 4. Summary plots of the scan for selection for all candidate regions of Table 1. Each file of this archive corresponds to a different candidate region, whose genomic position can be read in the file name. The top panel shows the results of the four tests for selection applied in the region. Values of the time-LWD (in blue), time-LWS (in green), hapFLK (in red) and FLK (in purple) tests are plotted (y axis) according to genomic position (x axis). Opposite signs are used for time-LWD and time-LWS on the one hand and hapFLK and FLK on the other hand, i.e. high test statistic values correspond to highly negative points for time-LWD and time-LWS and to highly positive points for hapFLK and FLK. All variants (AV set) were considered for these analyses. The bottom panel shows the position and name of genes located in the region. [file 12711_2023_789_MOESM4_ESM.xz › stat_profiles/all10_auto_chro11_7664458-7794933_scores.png]

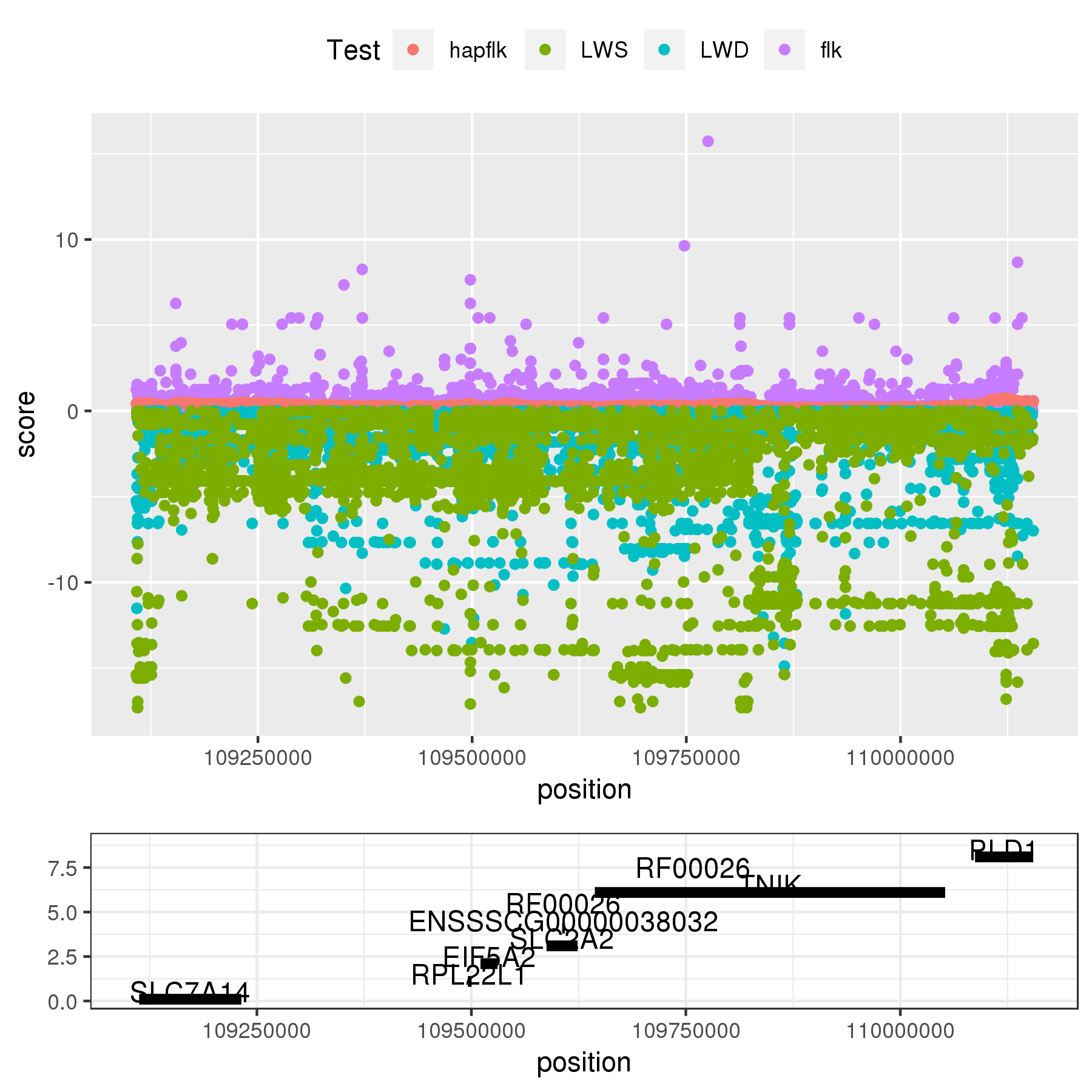

Supplement: Supplementary file 4 — Additional file 4. Summary plots of the scan for selection for all candidate regions of Table 1. Each file of this archive corresponds to a different candidate region, whose genomic position can be read in the file name. The top panel shows the results of the four tests for selection applied in the region. Values of the time-LWD (in blue), time-LWS (in green), hapFLK (in red) and FLK (in purple) tests are plotted (y axis) according to genomic position (x axis). Opposite signs are used for time-LWD and time-LWS on the one hand and hapFLK and FLK on the other hand, i.e. high test statistic values correspond to highly negative points for time-LWD and time-LWS and to highly positive points for hapFLK and FLK. All variants (AV set) were considered for these analyses. The bottom panel shows the position and name of genes located in the region. [file 12711_2023_789_MOESM4_ESM.xz › stat_profiles/all10_auto_chro13_109108079-110154951_scores.png]

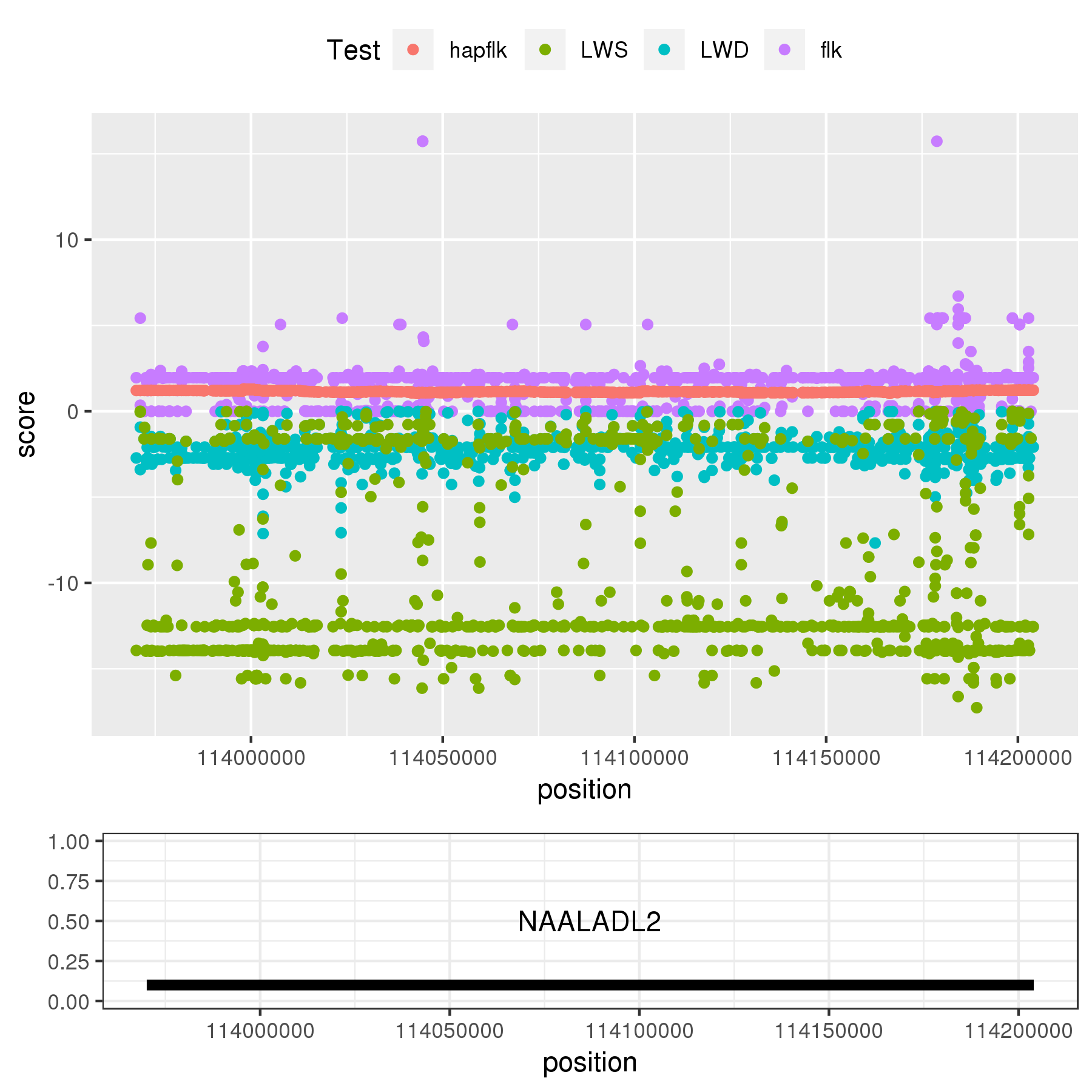

Supplement: Supplementary file 4 — Additional file 4. Summary plots of the scan for selection for all candidate regions of Table 1. Each file of this archive corresponds to a different candidate region, whose genomic position can be read in the file name. The top panel shows the results of the four tests for selection applied in the region. Values of the time-LWD (in blue), time-LWS (in green), hapFLK (in red) and FLK (in purple) tests are plotted (y axis) according to genomic position (x axis). Opposite signs are used for time-LWD and time-LWS on the one hand and hapFLK and FLK on the other hand, i.e. high test statistic values correspond to highly negative points for time-LWD and time-LWS and to highly positive points for hapFLK and FLK. All variants (AV set) were considered for these analyses. The bottom panel shows the position and name of genes located in the region. [file 12711_2023_789_MOESM4_ESM.xz › stat_profiles/all10_auto_chro13_113970091-114204018_scores.png]

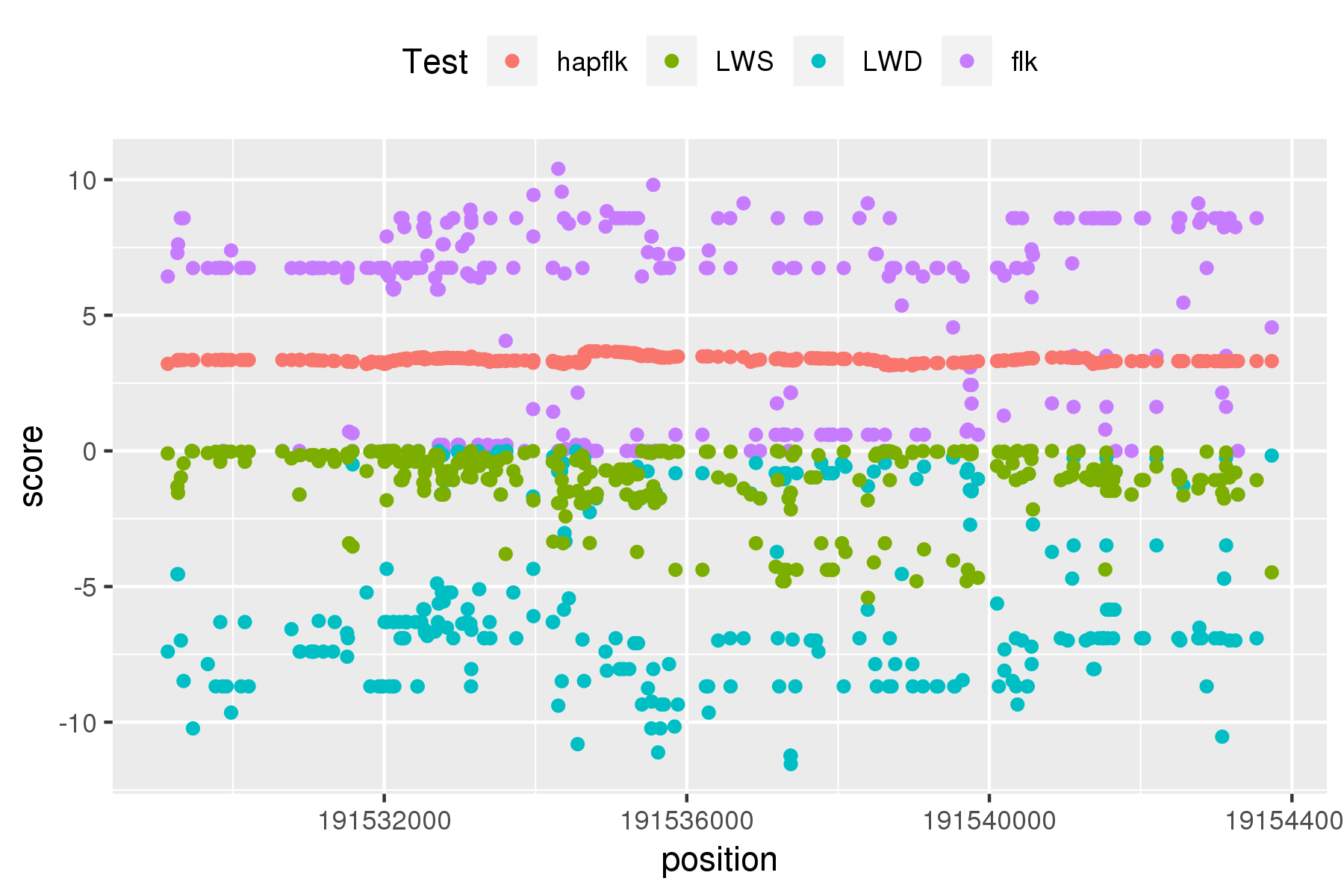

Supplement: Supplementary file 4 — Additional file 4. Summary plots of the scan for selection for all candidate regions of Table 1. Each file of this archive corresponds to a different candidate region, whose genomic position can be read in the file name. The top panel shows the results of the four tests for selection applied in the region. Values of the time-LWD (in blue), time-LWS (in green), hapFLK (in red) and FLK (in purple) tests are plotted (y axis) according to genomic position (x axis). Opposite signs are used for time-LWD and time-LWS on the one hand and hapFLK and FLK on the other hand, i.e. high test statistic values correspond to highly negative points for time-LWD and time-LWS and to highly positive points for hapFLK and FLK. All variants (AV set) were considered for these analyses. The bottom panel shows the position and name of genes located in the region. [file 12711_2023_789_MOESM4_ESM.xz › stat_profiles/all10_auto_chro13_191529139-191543733_scores.png]

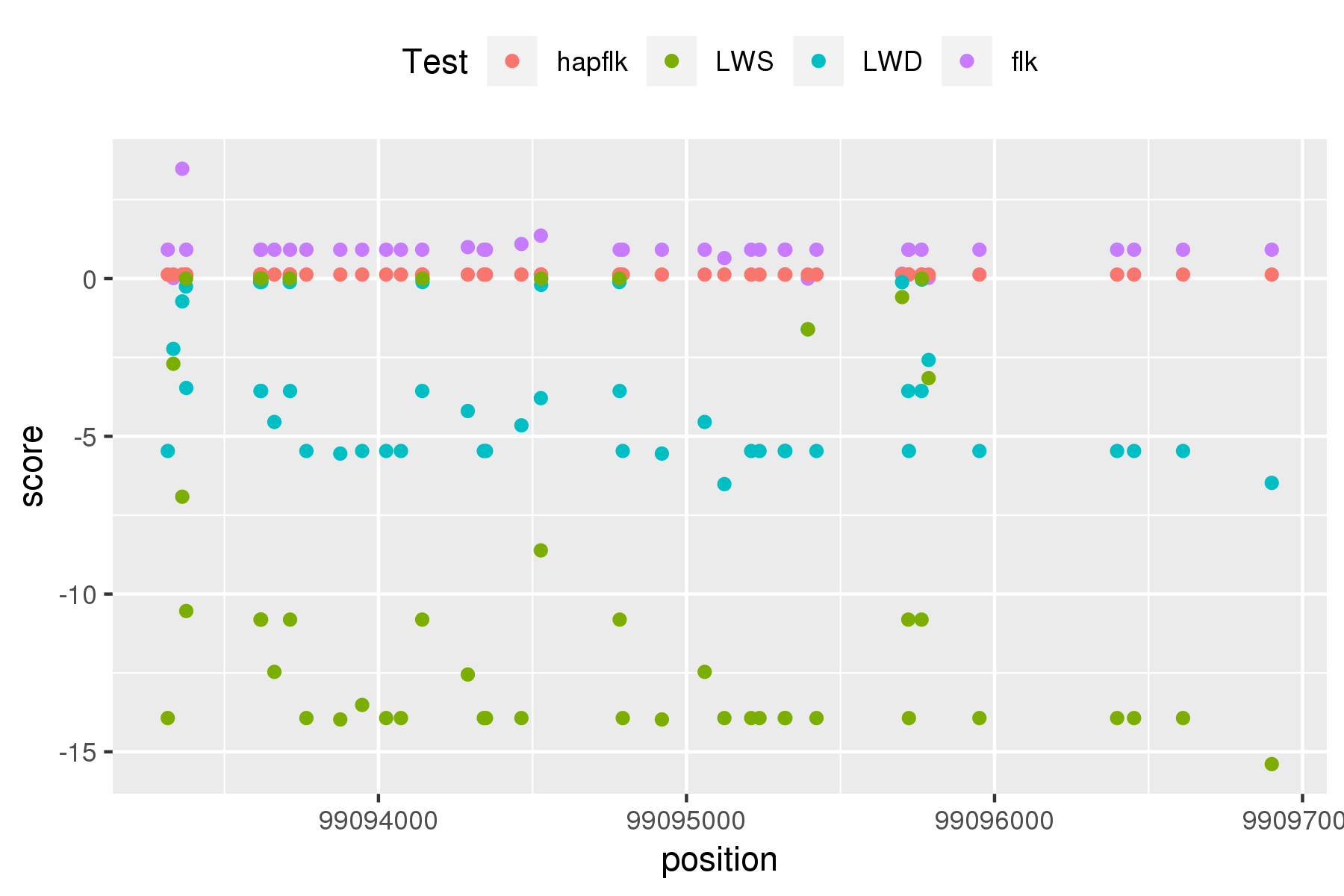

Supplement: Supplementary file 4 — Additional file 4. Summary plots of the scan for selection for all candidate regions of Table 1. Each file of this archive corresponds to a different candidate region, whose genomic position can be read in the file name. The top panel shows the results of the four tests for selection applied in the region. Values of the time-LWD (in blue), time-LWS (in green), hapFLK (in red) and FLK (in purple) tests are plotted (y axis) according to genomic position (x axis). Opposite signs are used for time-LWD and time-LWS on the one hand and hapFLK and FLK on the other hand, i.e. high test statistic values correspond to highly negative points for time-LWD and time-LWS and to highly positive points for hapFLK and FLK. All variants (AV set) were considered for these analyses. The bottom panel shows the position and name of genes located in the region. [file 12711_2023_789_MOESM4_ESM.xz › stat_profiles/all10_auto_chro13_99093316-99096900_scores.png]

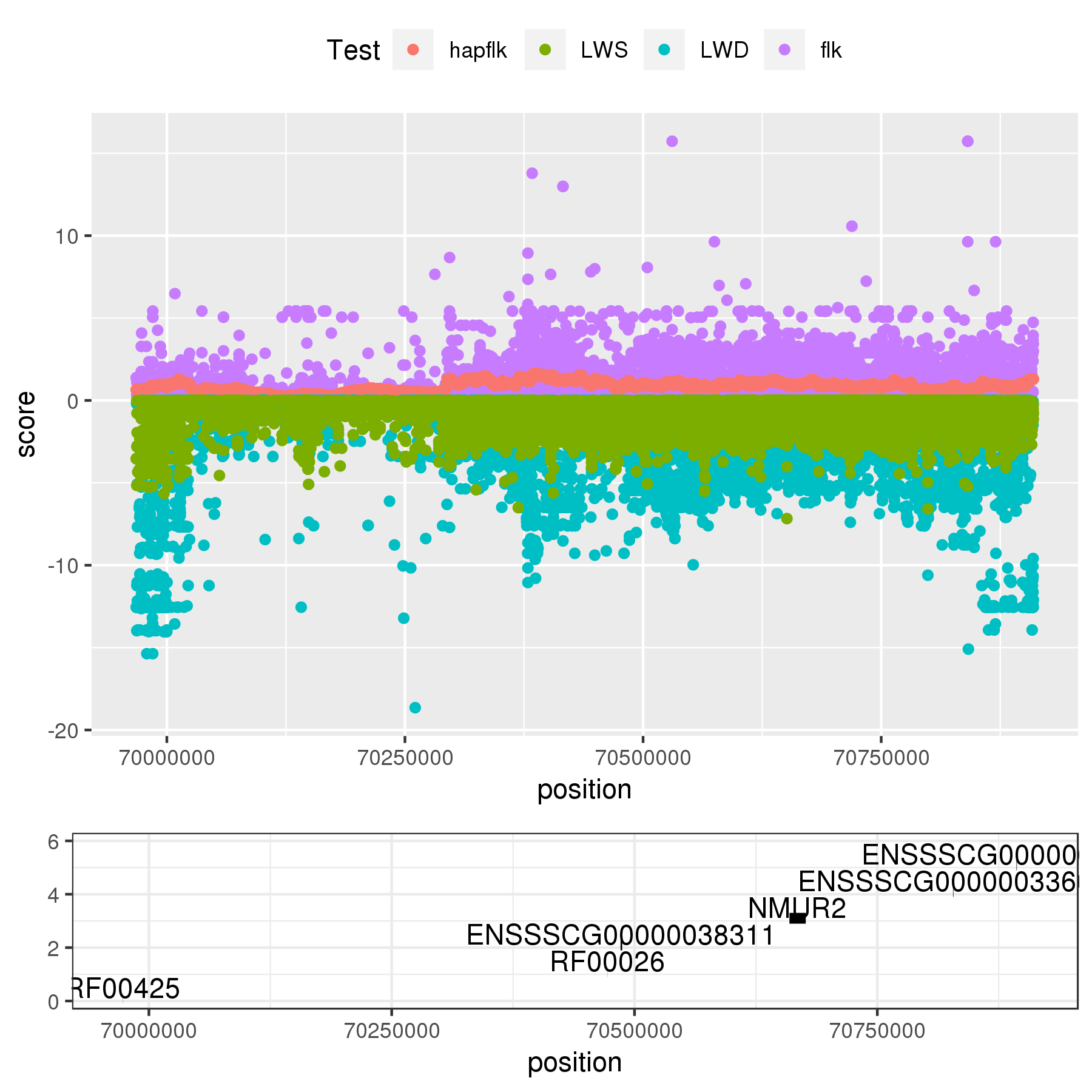

Supplement: Supplementary file 4 — Additional file 4. Summary plots of the scan for selection for all candidate regions of Table 1. Each file of this archive corresponds to a different candidate region, whose genomic position can be read in the file name. The top panel shows the results of the four tests for selection applied in the region. Values of the time-LWD (in blue), time-LWS (in green), hapFLK (in red) and FLK (in purple) tests are plotted (y axis) according to genomic position (x axis). Opposite signs are used for time-LWD and time-LWS on the one hand and hapFLK and FLK on the other hand, i.e. high test statistic values correspond to highly negative points for time-LWD and time-LWS and to highly positive points for hapFLK and FLK. All variants (AV set) were considered for these analyses. The bottom panel shows the position and name of genes located in the region. [file 12711_2023_789_MOESM4_ESM.xz › stat_profiles/all10_auto_chro16_69967970-70909691_scores.png]

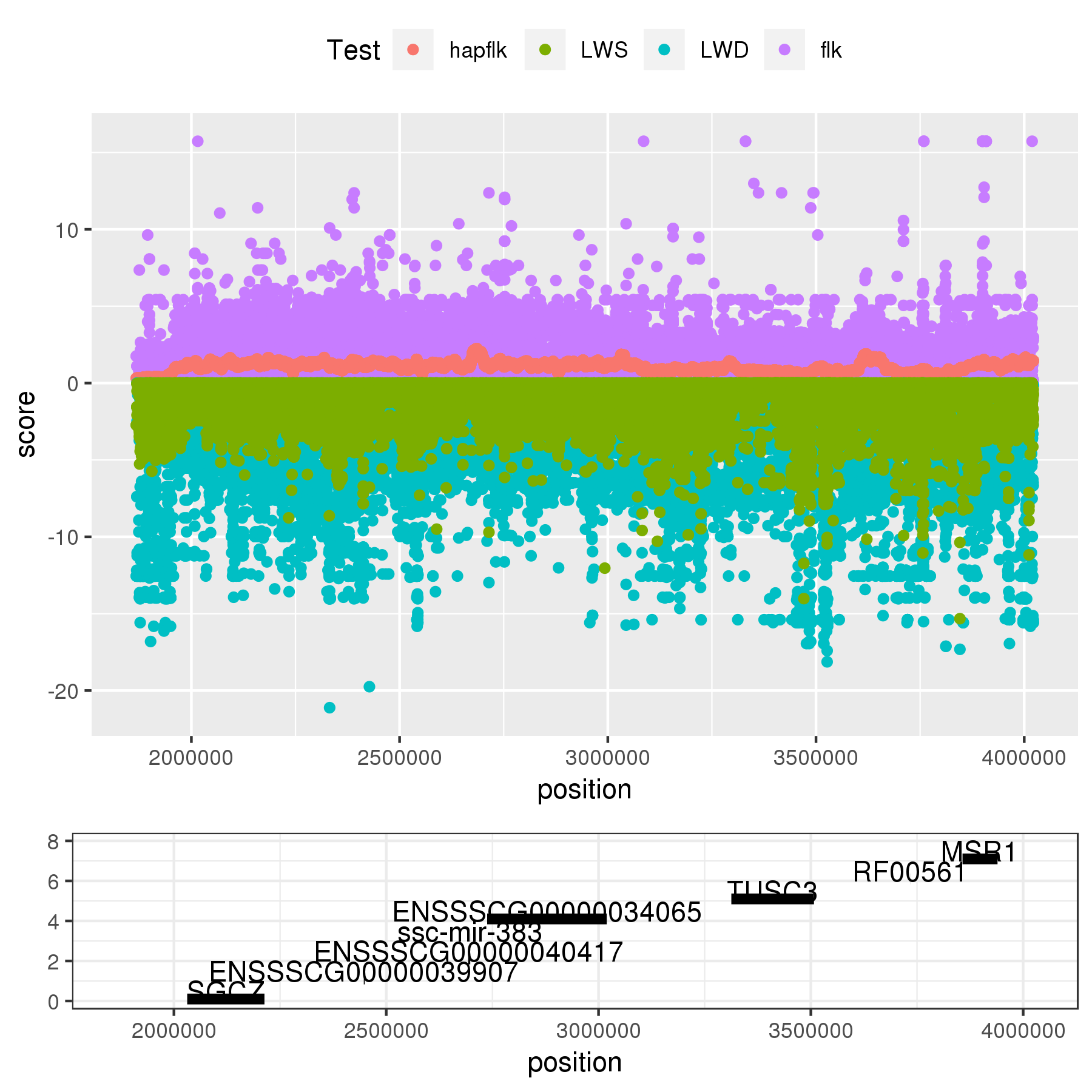

Supplement: Supplementary file 4 — Additional file 4. Summary plots of the scan for selection for all candidate regions of Table 1. Each file of this archive corresponds to a different candidate region, whose genomic position can be read in the file name. The top panel shows the results of the four tests for selection applied in the region. Values of the time-LWD (in blue), time-LWS (in green), hapFLK (in red) and FLK (in purple) tests are plotted (y axis) according to genomic position (x axis). Opposite signs are used for time-LWD and time-LWS on the one hand and hapFLK and FLK on the other hand, i.e. high test statistic values correspond to highly negative points for time-LWD and time-LWS and to highly positive points for hapFLK and FLK. All variants (AV set) were considered for these analyses. The bottom panel shows the position and name of genes located in the region. [file 12711_2023_789_MOESM4_ESM.xz › stat_profiles/all10_auto_chro17_1867811-4021774_scores.png]

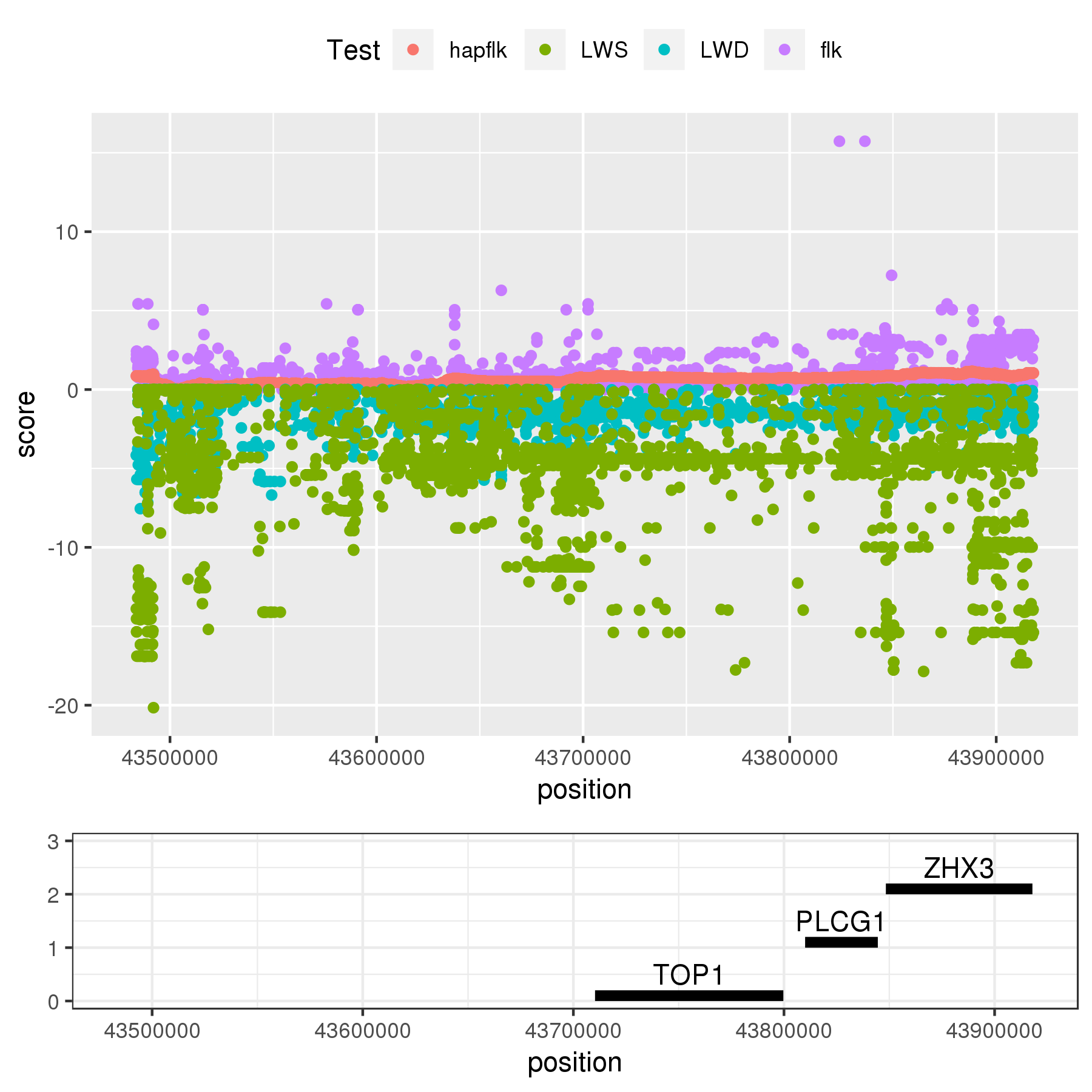

Supplement: Supplementary file 4 — Additional file 4. Summary plots of the scan for selection for all candidate regions of Table 1. Each file of this archive corresponds to a different candidate region, whose genomic position can be read in the file name. The top panel shows the results of the four tests for selection applied in the region. Values of the time-LWD (in blue), time-LWS (in green), hapFLK (in red) and FLK (in purple) tests are plotted (y axis) according to genomic position (x axis). Opposite signs are used for time-LWD and time-LWS on the one hand and hapFLK and FLK on the other hand, i.e. high test statistic values correspond to highly negative points for time-LWD and time-LWS and to highly positive points for hapFLK and FLK. All variants (AV set) were considered for these analyses. The bottom panel shows the position and name of genes located in the region. [file 12711_2023_789_MOESM4_ESM.xz › stat_profiles/all10_auto_chro17_43483704-43917949_scores.png]

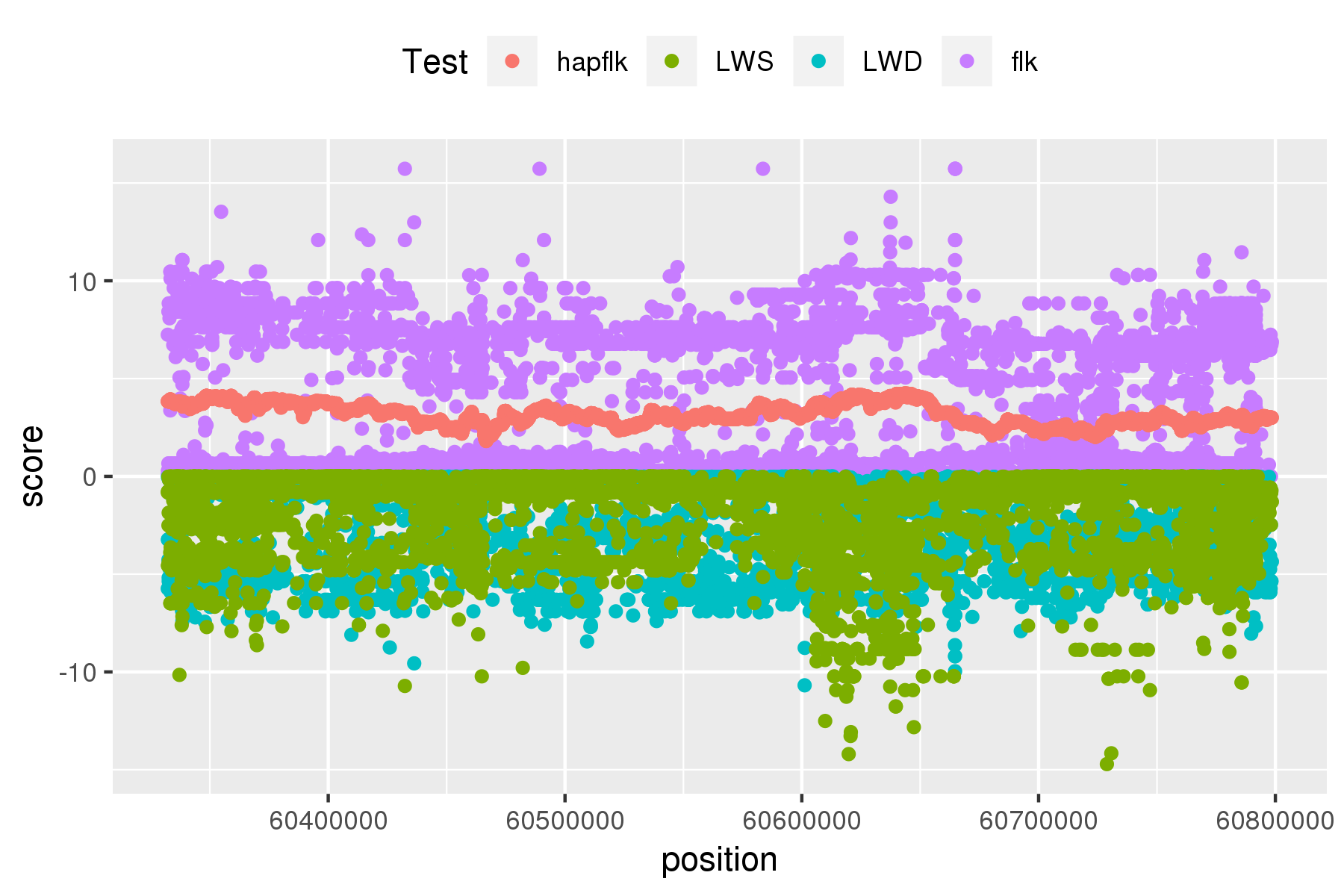

Supplement: Supplementary file 4 — Additional file 4. Summary plots of the scan for selection for all candidate regions of Table 1. Each file of this archive corresponds to a different candidate region, whose genomic position can be read in the file name. The top panel shows the results of the four tests for selection applied in the region. Values of the time-LWD (in blue), time-LWS (in green), hapFLK (in red) and FLK (in purple) tests are plotted (y axis) according to genomic position (x axis). Opposite signs are used for time-LWD and time-LWS on the one hand and hapFLK and FLK on the other hand, i.e. high test statistic values correspond to highly negative points for time-LWD and time-LWS and to highly positive points for hapFLK and FLK. All variants (AV set) were considered for these analyses. The bottom panel shows the position and name of genes located in the region. [file 12711_2023_789_MOESM4_ESM.xz › stat_profiles/all10_auto_chro17_60332242-60798465_scores.png]

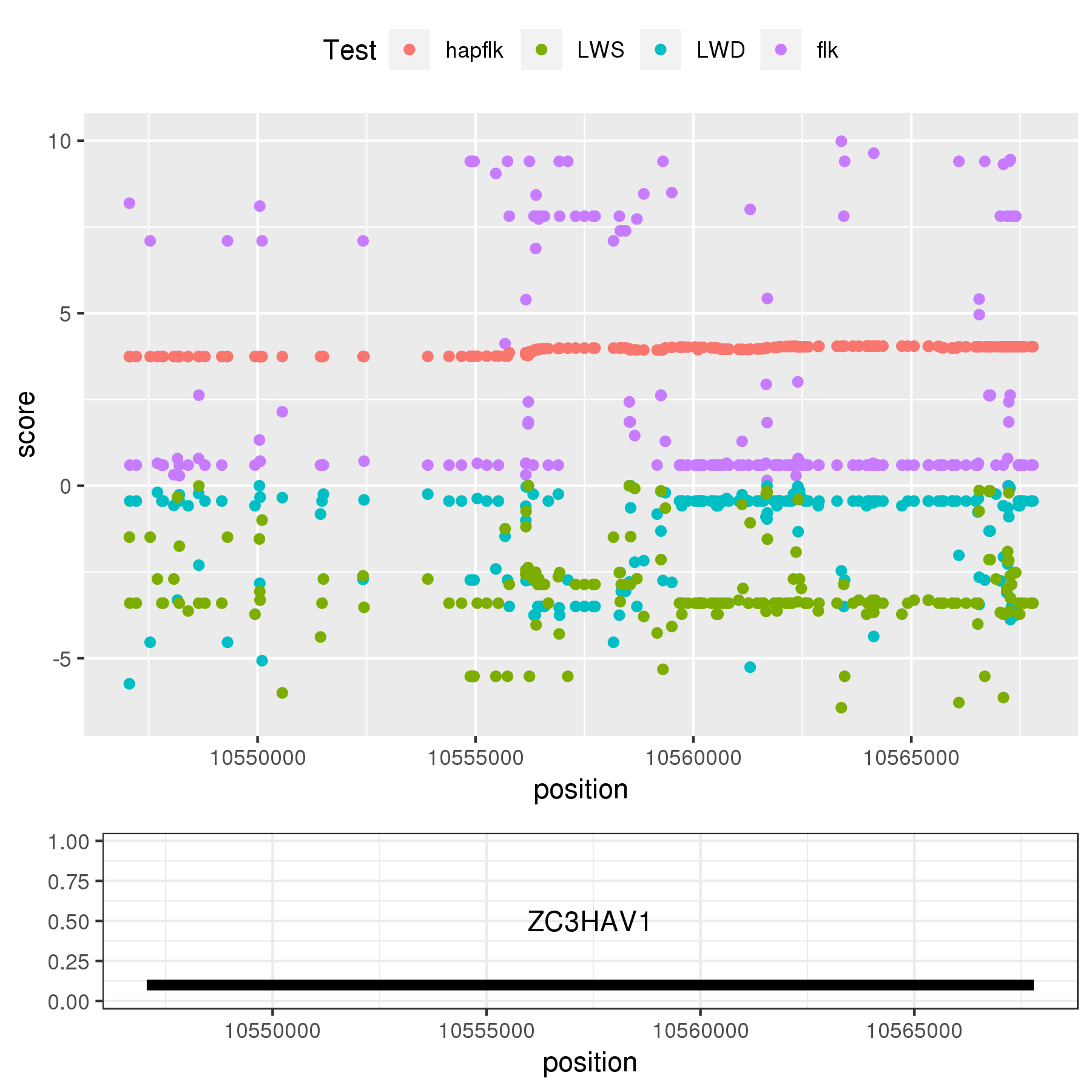

Supplement: Supplementary file 4 — Additional file 4. Summary plots of the scan for selection for all candidate regions of Table 1. Each file of this archive corresponds to a different candidate region, whose genomic position can be read in the file name. The top panel shows the results of the four tests for selection applied in the region. Values of the time-LWD (in blue), time-LWS (in green), hapFLK (in red) and FLK (in purple) tests are plotted (y axis) according to genomic position (x axis). Opposite signs are used for time-LWD and time-LWS on the one hand and hapFLK and FLK on the other hand, i.e. high test statistic values correspond to highly negative points for time-LWD and time-LWS and to highly positive points for hapFLK and FLK. All variants (AV set) were considered for these analyses. The bottom panel shows the position and name of genes located in the region. [file 12711_2023_789_MOESM4_ESM.xz › stat_profiles/all10_auto_chro18_10547058-10567791_scores.png]

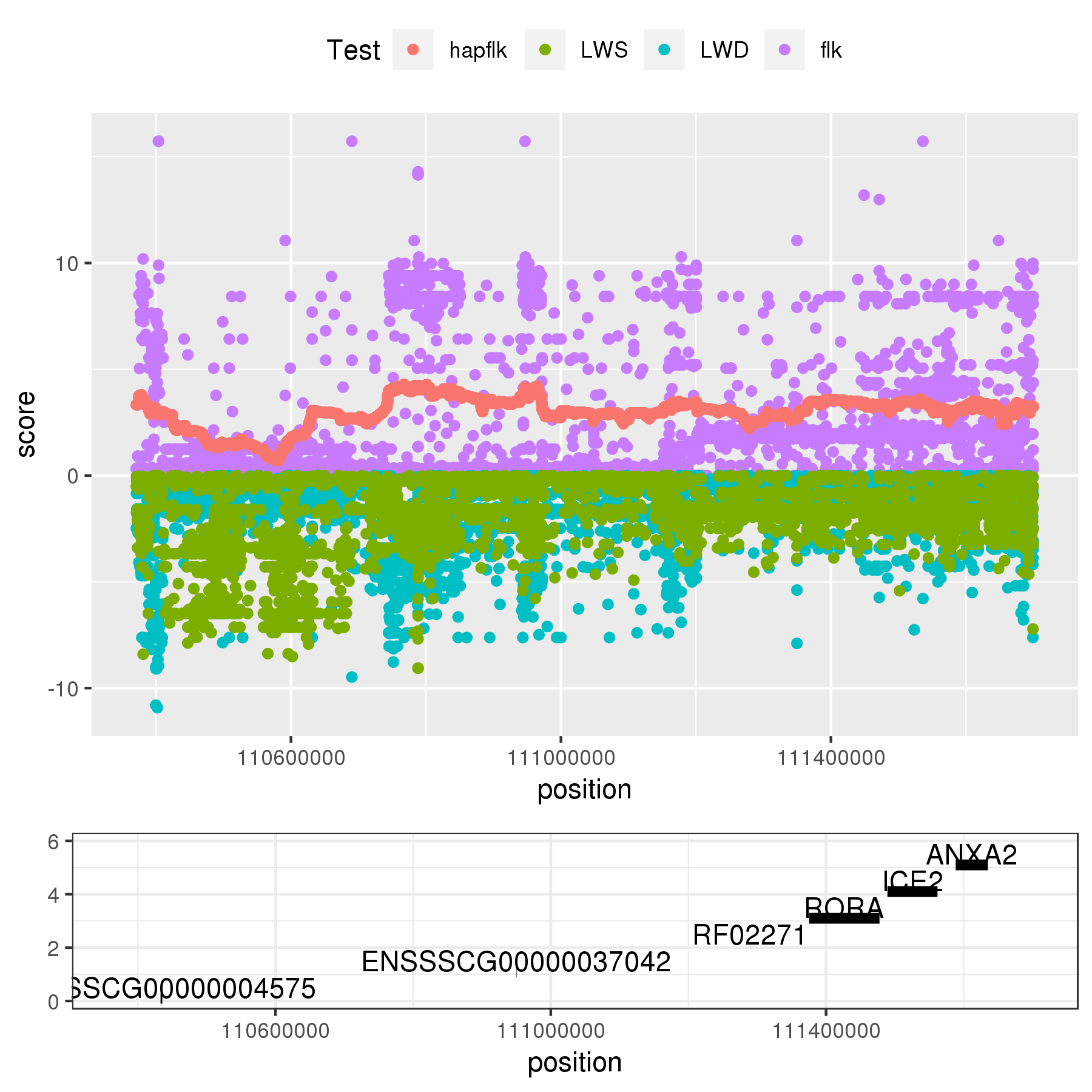

Supplement: Supplementary file 4 — Additional file 4. Summary plots of the scan for selection for all candidate regions of Table 1. Each file of this archive corresponds to a different candidate region, whose genomic position can be read in the file name. The top panel shows the results of the four tests for selection applied in the region. Values of the time-LWD (in blue), time-LWS (in green), hapFLK (in red) and FLK (in purple) tests are plotted (y axis) according to genomic position (x axis). Opposite signs are used for time-LWD and time-LWS on the one hand and hapFLK and FLK on the other hand, i.e. high test statistic values correspond to highly negative points for time-LWD and time-LWS and to highly positive points for hapFLK and FLK. All variants (AV set) were considered for these analyses. The bottom panel shows the position and name of genes located in the region. [file 12711_2023_789_MOESM4_ESM.xz › stat_profiles/all10_auto_chro1_110370901-111699835_scores.png]

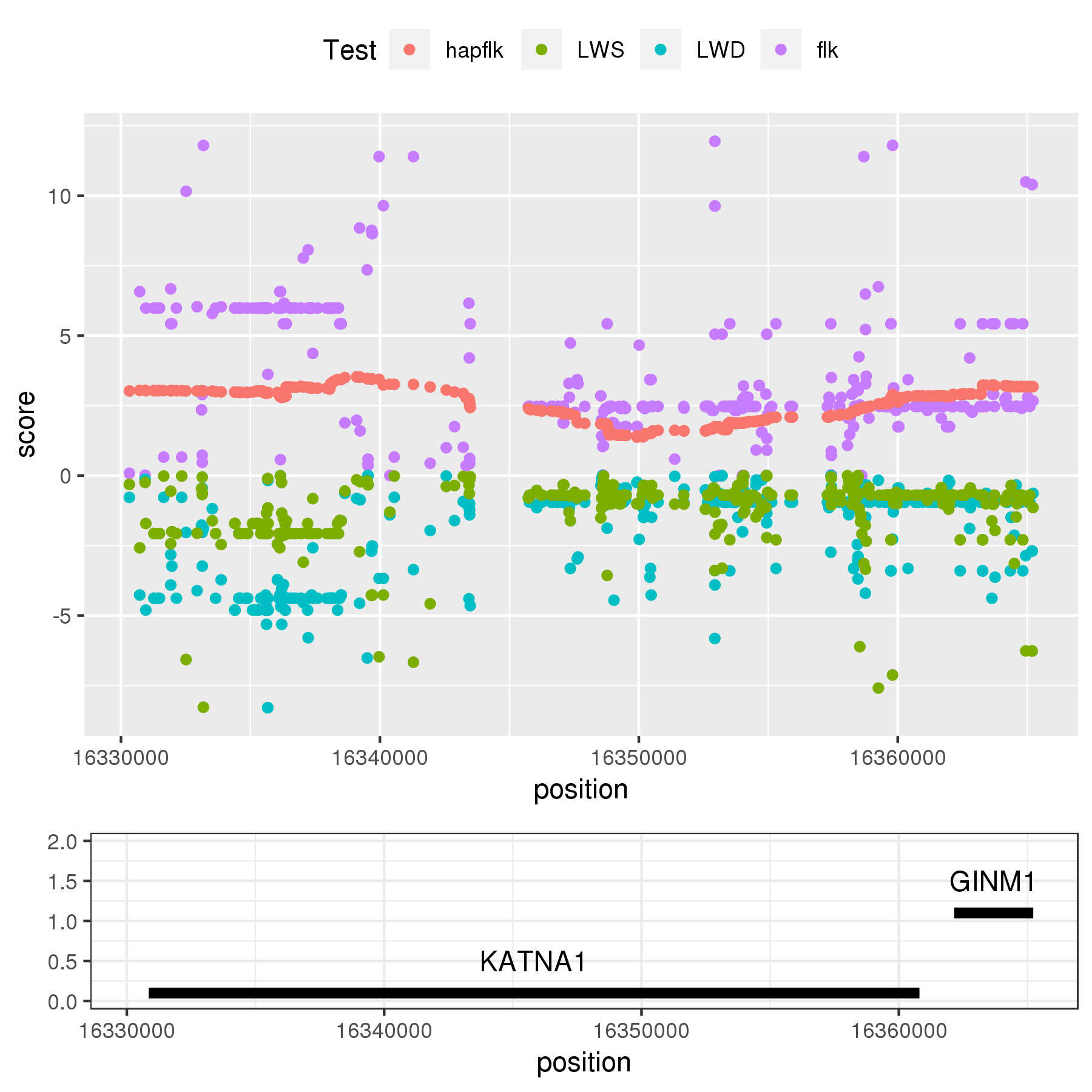

Supplement: Supplementary file 4 — Additional file 4. Summary plots of the scan for selection for all candidate regions of Table 1. Each file of this archive corresponds to a different candidate region, whose genomic position can be read in the file name. The top panel shows the results of the four tests for selection applied in the region. Values of the time-LWD (in blue), time-LWS (in green), hapFLK (in red) and FLK (in purple) tests are plotted (y axis) according to genomic position (x axis). Opposite signs are used for time-LWD and time-LWS on the one hand and hapFLK and FLK on the other hand, i.e. high test statistic values correspond to highly negative points for time-LWD and time-LWS and to highly positive points for hapFLK and FLK. All variants (AV set) were considered for these analyses. The bottom panel shows the position and name of genes located in the region. [file 12711_2023_789_MOESM4_ESM.xz › stat_profiles/all10_auto_chro1_16330324-16365222_scores.png]

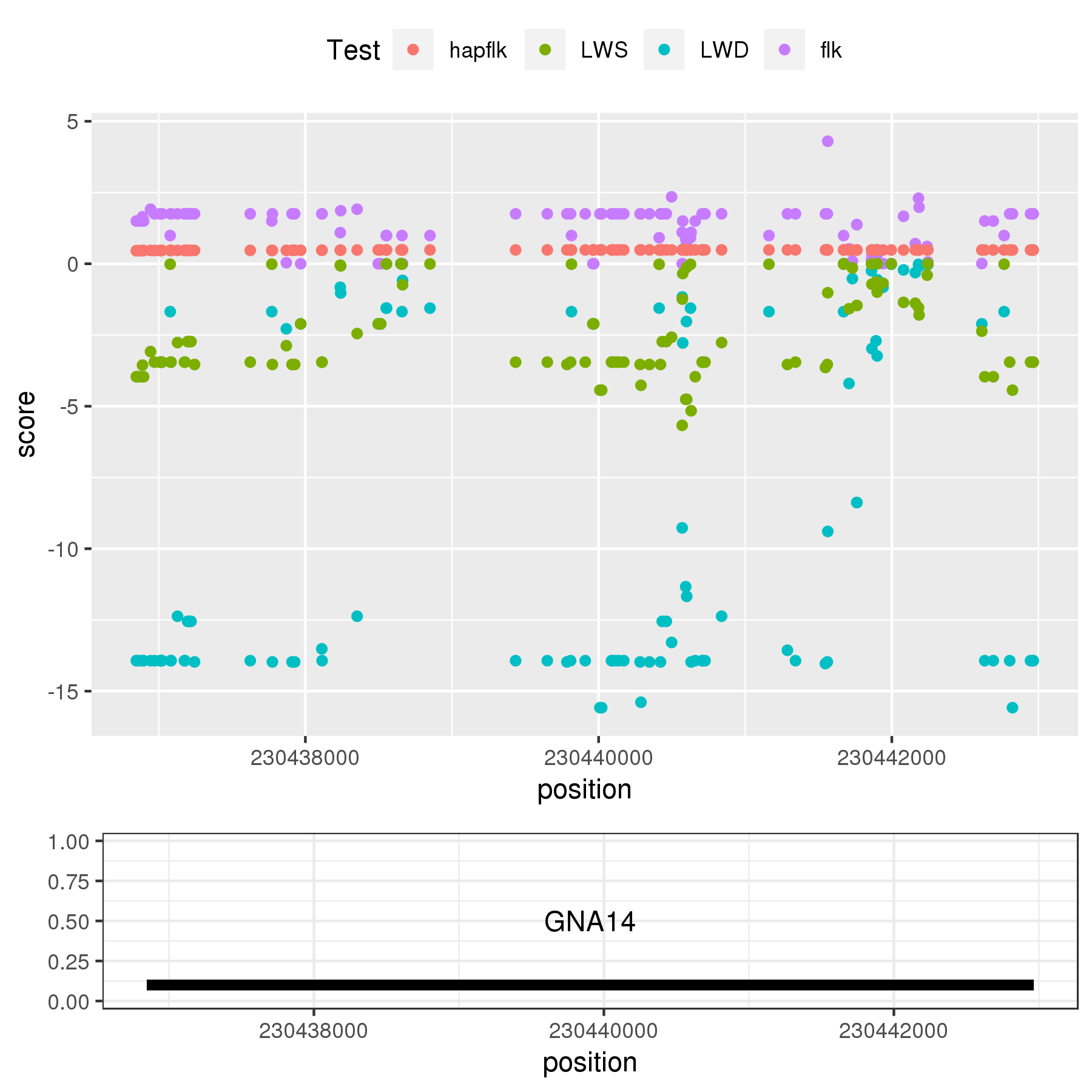

Supplement: Supplementary file 4 — Additional file 4. Summary plots of the scan for selection for all candidate regions of Table 1. Each file of this archive corresponds to a different candidate region, whose genomic position can be read in the file name. The top panel shows the results of the four tests for selection applied in the region. Values of the time-LWD (in blue), time-LWS (in green), hapFLK (in red) and FLK (in purple) tests are plotted (y axis) according to genomic position (x axis). Opposite signs are used for time-LWD and time-LWS on the one hand and hapFLK and FLK on the other hand, i.e. high test statistic values correspond to highly negative points for time-LWD and time-LWS and to highly positive points for hapFLK and FLK. All variants (AV set) were considered for these analyses. The bottom panel shows the position and name of genes located in the region. [file 12711_2023_789_MOESM4_ESM.xz › stat_profiles/all10_auto_chro1_230436847-230442965_scores.png]

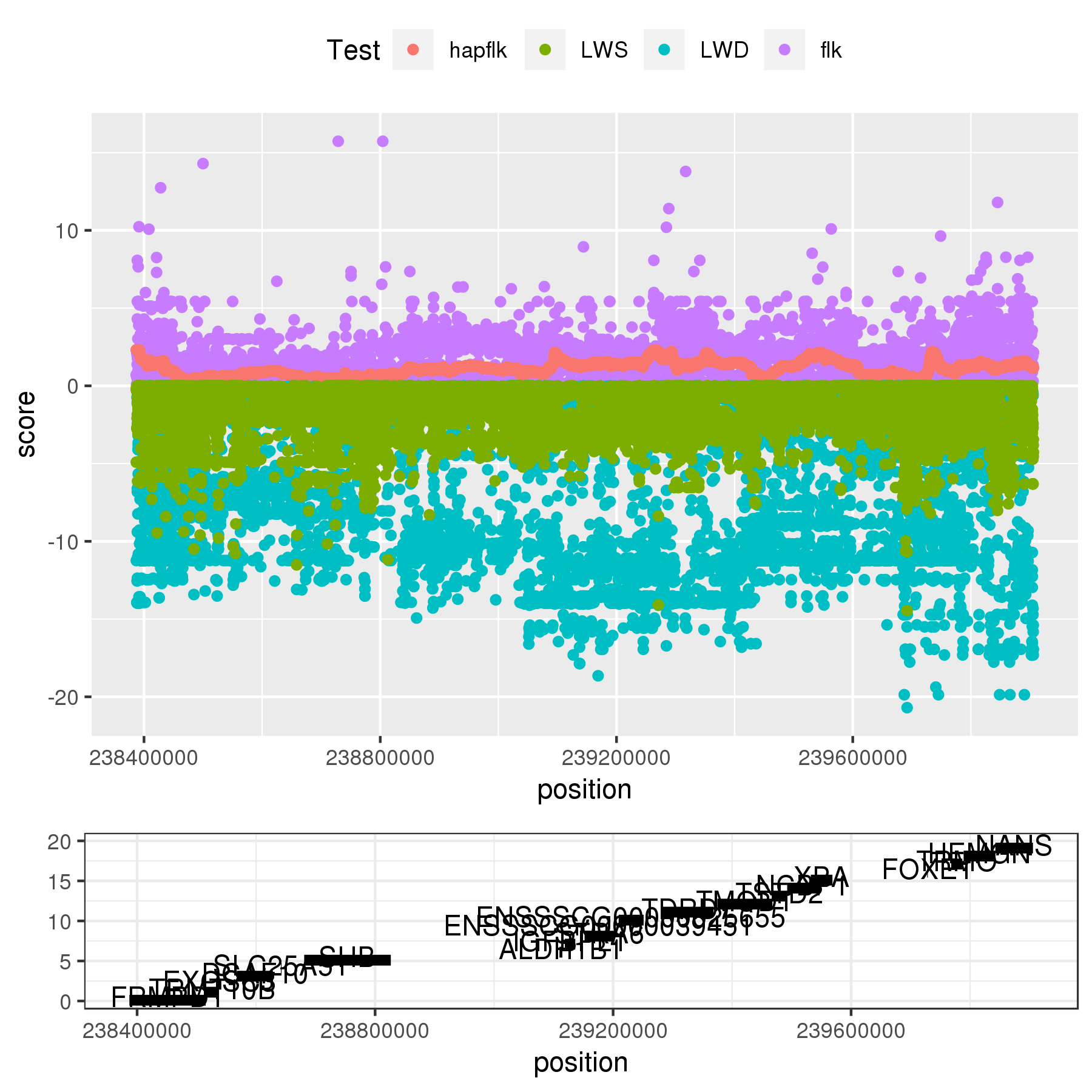

Supplement: Supplementary file 4 — Additional file 4. Summary plots of the scan for selection for all candidate regions of Table 1. Each file of this archive corresponds to a different candidate region, whose genomic position can be read in the file name. The top panel shows the results of the four tests for selection applied in the region. Values of the time-LWD (in blue), time-LWS (in green), hapFLK (in red) and FLK (in purple) tests are plotted (y axis) according to genomic position (x axis). Opposite signs are used for time-LWD and time-LWS on the one hand and hapFLK and FLK on the other hand, i.e. high test statistic values correspond to highly negative points for time-LWD and time-LWS and to highly positive points for hapFLK and FLK. All variants (AV set) were considered for these analyses. The bottom panel shows the position and name of genes located in the region. [file 12711_2023_789_MOESM4_ESM.xz › stat_profiles/all10_auto_chro1_238387016-239905667_scores.png]

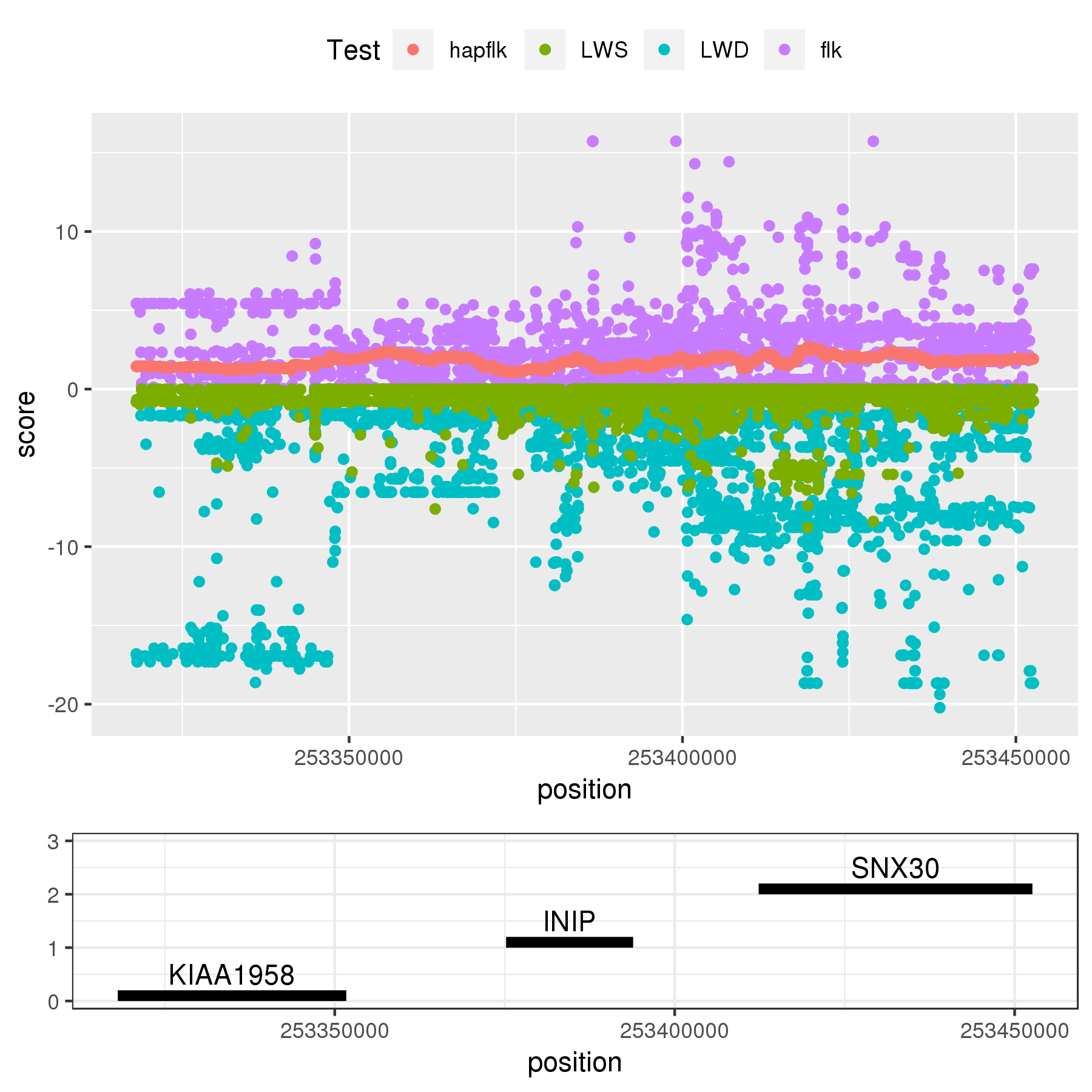

Supplement: Supplementary file 4 — Additional file 4. Summary plots of the scan for selection for all candidate regions of Table 1. Each file of this archive corresponds to a different candidate region, whose genomic position can be read in the file name. The top panel shows the results of the four tests for selection applied in the region. Values of the time-LWD (in blue), time-LWS (in green), hapFLK (in red) and FLK (in purple) tests are plotted (y axis) according to genomic position (x axis). Opposite signs are used for time-LWD and time-LWS on the one hand and hapFLK and FLK on the other hand, i.e. high test statistic values correspond to highly negative points for time-LWD and time-LWS and to highly positive points for hapFLK and FLK. All variants (AV set) were considered for these analyses. The bottom panel shows the position and name of genes located in the region. [file 12711_2023_789_MOESM4_ESM.xz › stat_profiles/all10_auto_chro1_253318090-253452613_scores.png]

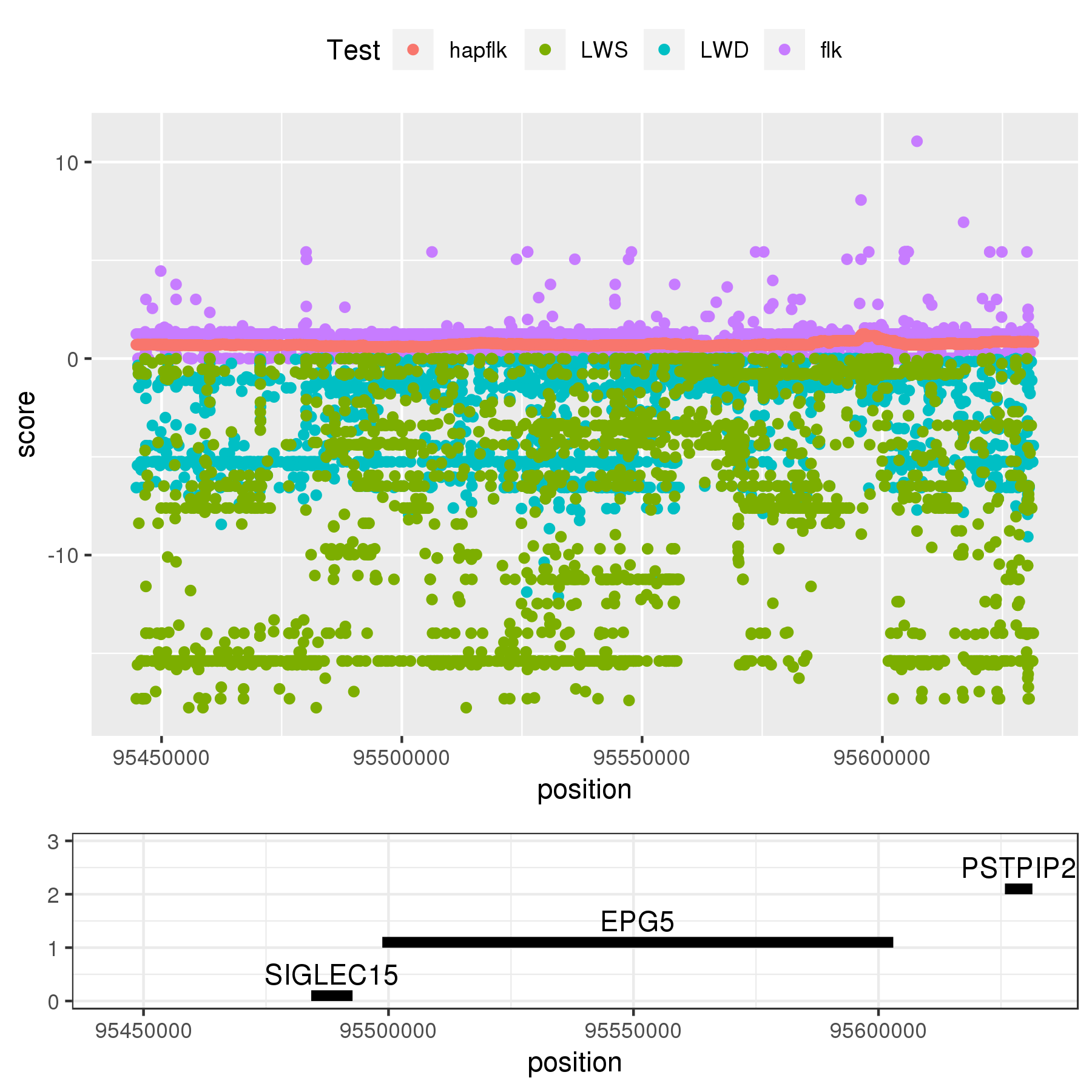

Supplement: Supplementary file 4 — Additional file 4. Summary plots of the scan for selection for all candidate regions of Table 1. Each file of this archive corresponds to a different candidate region, whose genomic position can be read in the file name. The top panel shows the results of the four tests for selection applied in the region. Values of the time-LWD (in blue), time-LWS (in green), hapFLK (in red) and FLK (in purple) tests are plotted (y axis) according to genomic position (x axis). Opposite signs are used for time-LWD and time-LWS on the one hand and hapFLK and FLK on the other hand, i.e. high test statistic values correspond to highly negative points for time-LWD and time-LWS and to highly positive points for hapFLK and FLK. All variants (AV set) were considered for these analyses. The bottom panel shows the position and name of genes located in the region. [file 12711_2023_789_MOESM4_ESM.xz › stat_profiles/all10_auto_chro1_95444749-95631414_scores.png]

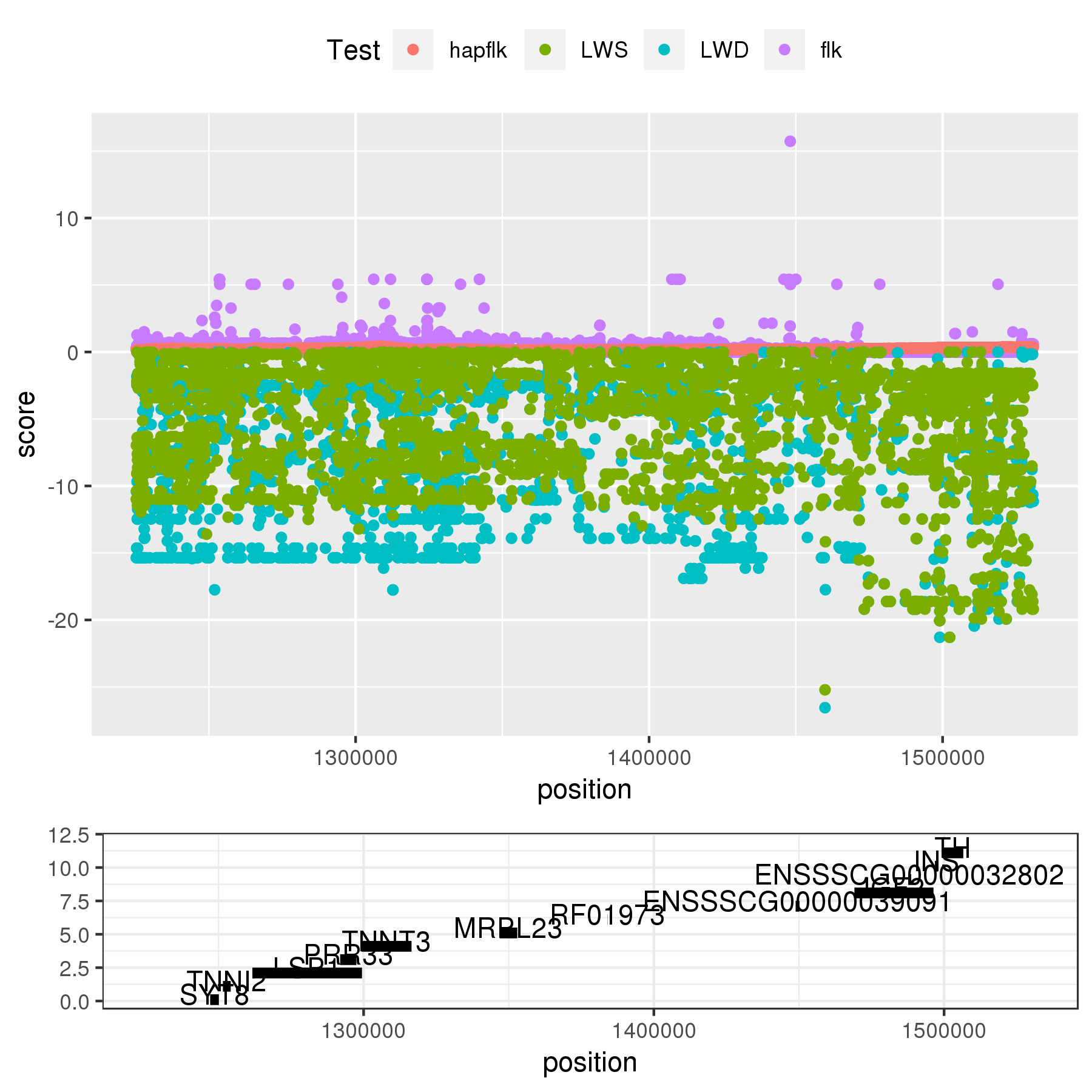

Supplement: Supplementary file 4 — Additional file 4. Summary plots of the scan for selection for all candidate regions of Table 1. Each file of this archive corresponds to a different candidate region, whose genomic position can be read in the file name. The top panel shows the results of the four tests for selection applied in the region. Values of the time-LWD (in blue), time-LWS (in green), hapFLK (in red) and FLK (in purple) tests are plotted (y axis) according to genomic position (x axis). Opposite signs are used for time-LWD and time-LWS on the one hand and hapFLK and FLK on the other hand, i.e. high test statistic values correspond to highly negative points for time-LWD and time-LWS and to highly positive points for hapFLK and FLK. All variants (AV set) were considered for these analyses. The bottom panel shows the position and name of genes located in the region. [file 12711_2023_789_MOESM4_ESM.xz › stat_profiles/all10_auto_chro2_1225288-1530903_scores.png]

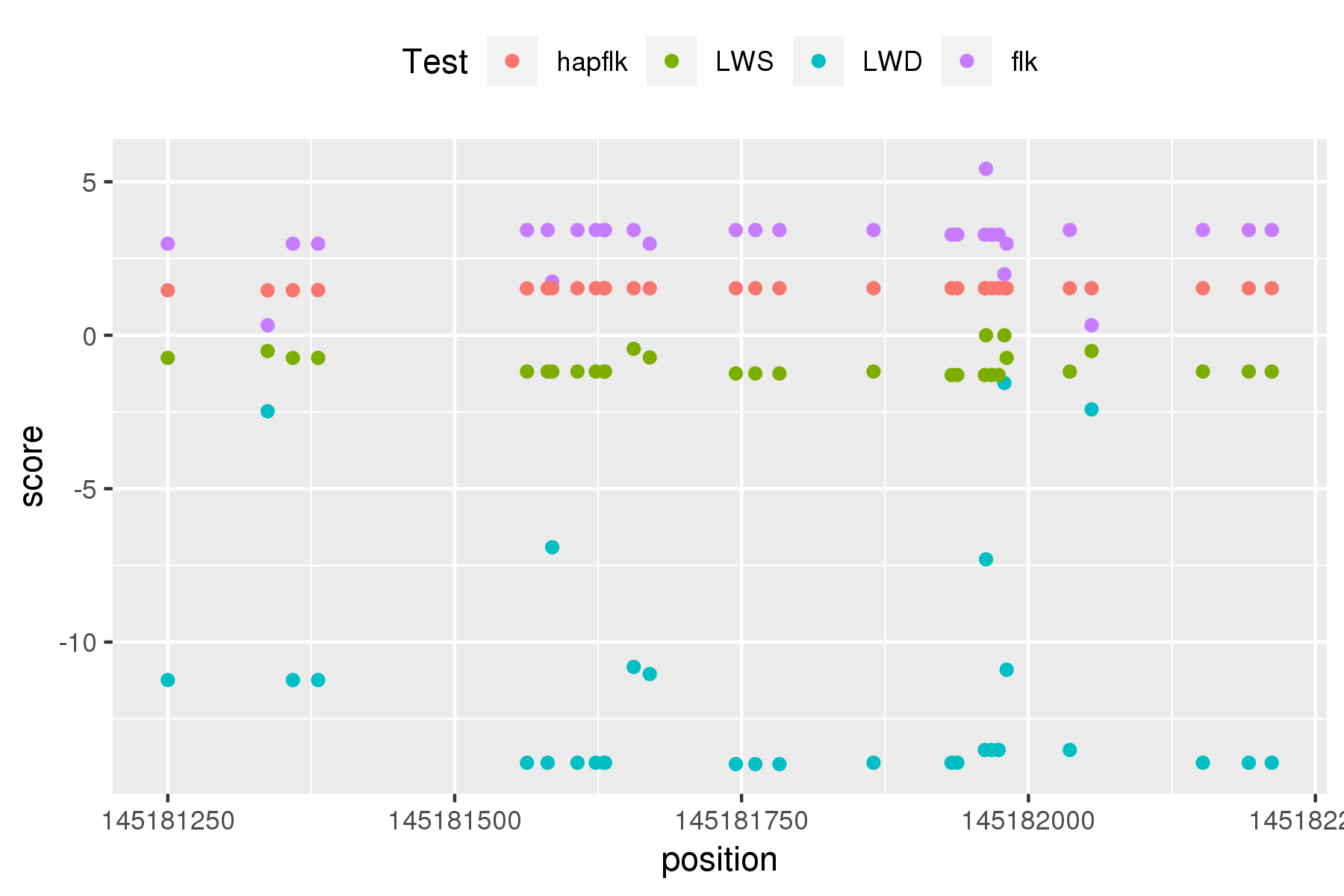

Supplement: Supplementary file 4 — Additional file 4. Summary plots of the scan for selection for all candidate regions of Table 1. Each file of this archive corresponds to a different candidate region, whose genomic position can be read in the file name. The top panel shows the results of the four tests for selection applied in the region. Values of the time-LWD (in blue), time-LWS (in green), hapFLK (in red) and FLK (in purple) tests are plotted (y axis) according to genomic position (x axis). Opposite signs are used for time-LWD and time-LWS on the one hand and hapFLK and FLK on the other hand, i.e. high test statistic values correspond to highly negative points for time-LWD and time-LWS and to highly positive points for hapFLK and FLK. All variants (AV set) were considered for these analyses. The bottom panel shows the position and name of genes located in the region. [file 12711_2023_789_MOESM4_ESM.xz › stat_profiles/all10_auto_chro2_145181250-145182212_scores.png]

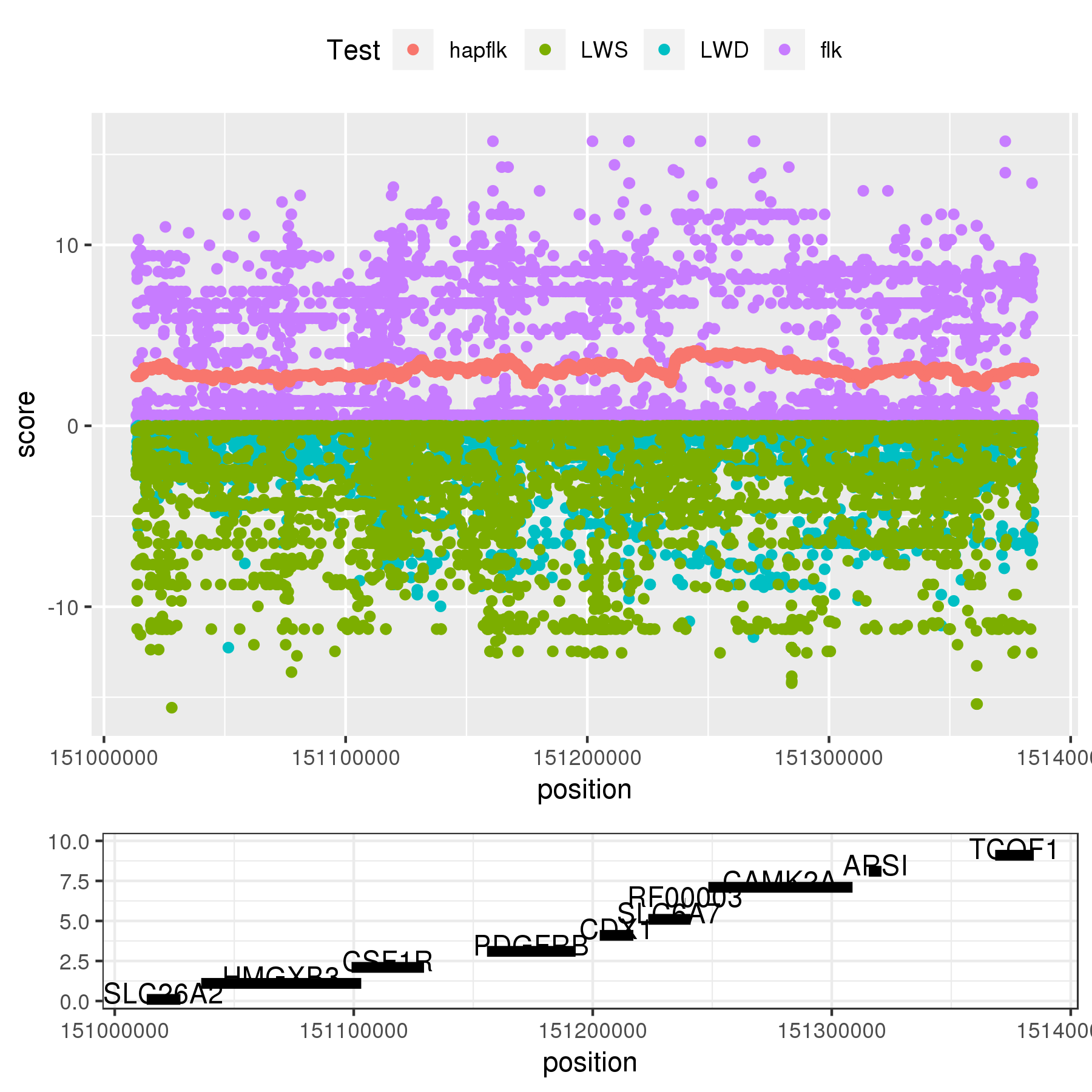

Supplement: Supplementary file 4 — Additional file 4. Summary plots of the scan for selection for all candidate regions of Table 1. Each file of this archive corresponds to a different candidate region, whose genomic position can be read in the file name. The top panel shows the results of the four tests for selection applied in the region. Values of the time-LWD (in blue), time-LWS (in green), hapFLK (in red) and FLK (in purple) tests are plotted (y axis) according to genomic position (x axis). Opposite signs are used for time-LWD and time-LWS on the one hand and hapFLK and FLK on the other hand, i.e. high test statistic values correspond to highly negative points for time-LWD and time-LWS and to highly positive points for hapFLK and FLK. All variants (AV set) were considered for these analyses. The bottom panel shows the position and name of genes located in the region. [file 12711_2023_789_MOESM4_ESM.xz › stat_profiles/all10_auto_chro2_151013393-151384536_scores.png]

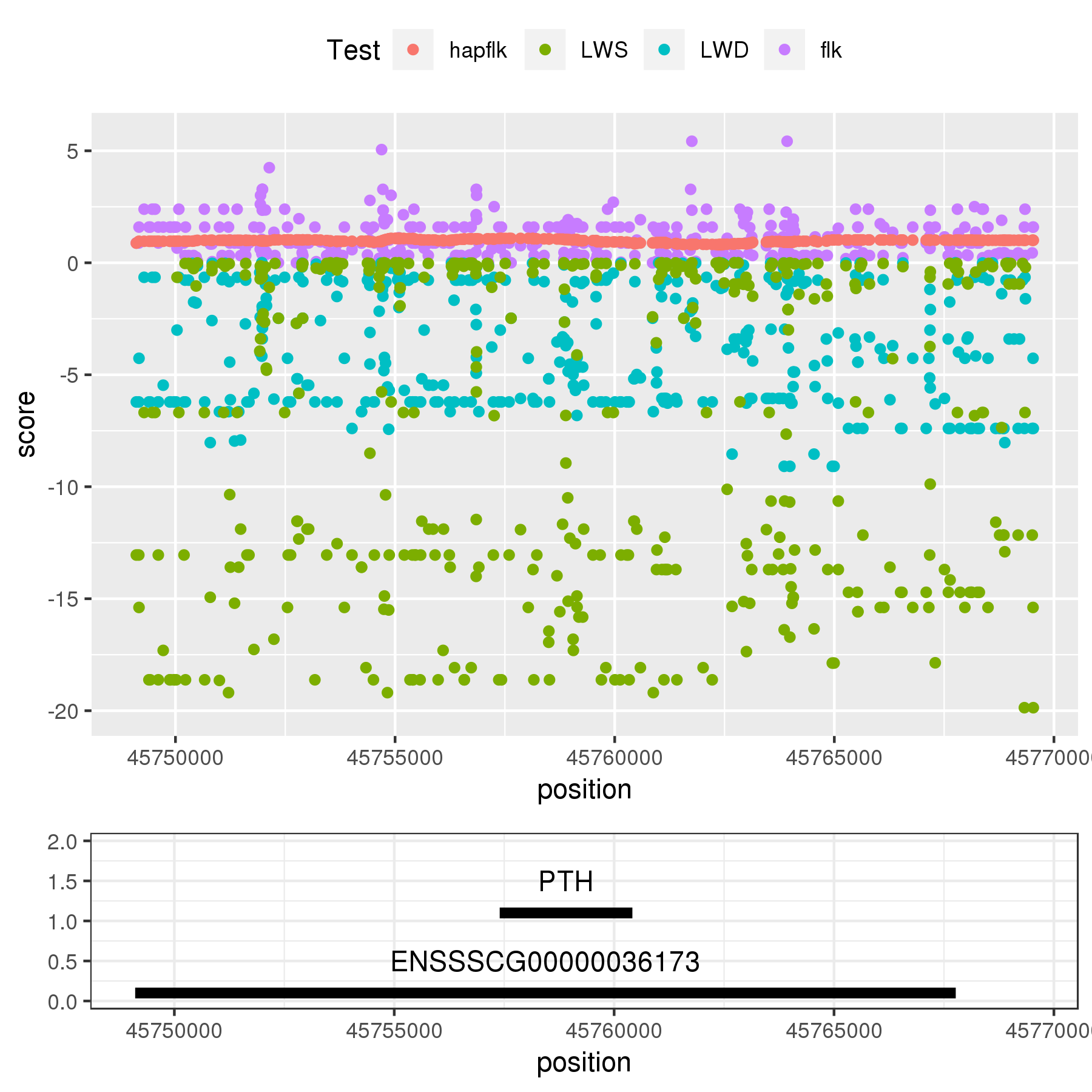

Supplement: Supplementary file 4 — Additional file 4. Summary plots of the scan for selection for all candidate regions of Table 1. Each file of this archive corresponds to a different candidate region, whose genomic position can be read in the file name. The top panel shows the results of the four tests for selection applied in the region. Values of the time-LWD (in blue), time-LWS (in green), hapFLK (in red) and FLK (in purple) tests are plotted (y axis) according to genomic position (x axis). Opposite signs are used for time-LWD and time-LWS on the one hand and hapFLK and FLK on the other hand, i.e. high test statistic values correspond to highly negative points for time-LWD and time-LWS and to highly positive points for hapFLK and FLK. All variants (AV set) were considered for these analyses. The bottom panel shows the position and name of genes located in the region. [file 12711_2023_789_MOESM4_ESM.xz › stat_profiles/all10_auto_chro2_45749109-45769530_scores.png]

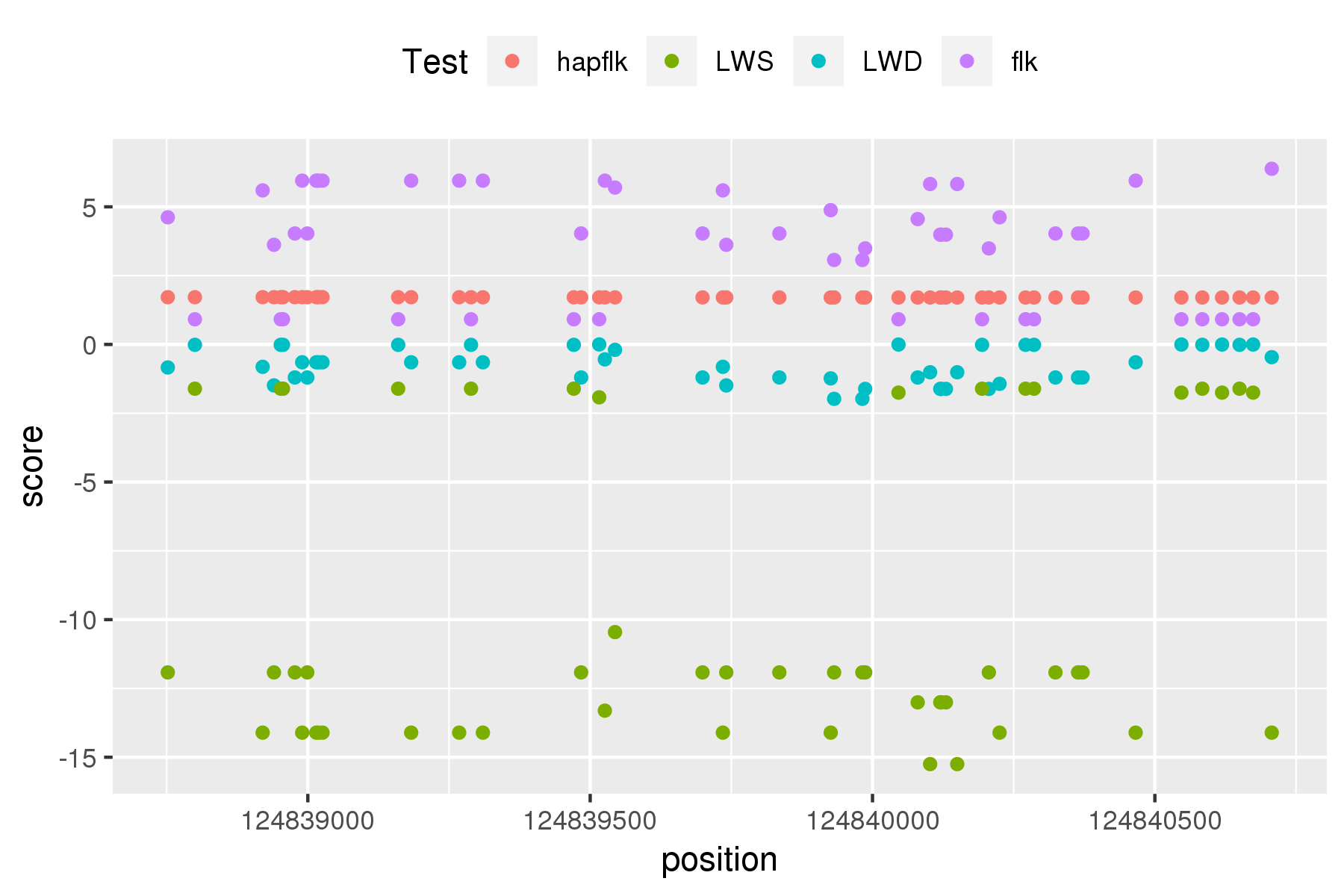

Supplement: Supplementary file 4 — Additional file 4. Summary plots of the scan for selection for all candidate regions of Table 1. Each file of this archive corresponds to a different candidate region, whose genomic position can be read in the file name. The top panel shows the results of the four tests for selection applied in the region. Values of the time-LWD (in blue), time-LWS (in green), hapFLK (in red) and FLK (in purple) tests are plotted (y axis) according to genomic position (x axis). Opposite signs are used for time-LWD and time-LWS on the one hand and hapFLK and FLK on the other hand, i.e. high test statistic values correspond to highly negative points for time-LWD and time-LWS and to highly positive points for hapFLK and FLK. All variants (AV set) were considered for these analyses. The bottom panel shows the position and name of genes located in the region. [file 12711_2023_789_MOESM4_ESM.xz › stat_profiles/all10_auto_chro3_124838752-124840707_scores.png]

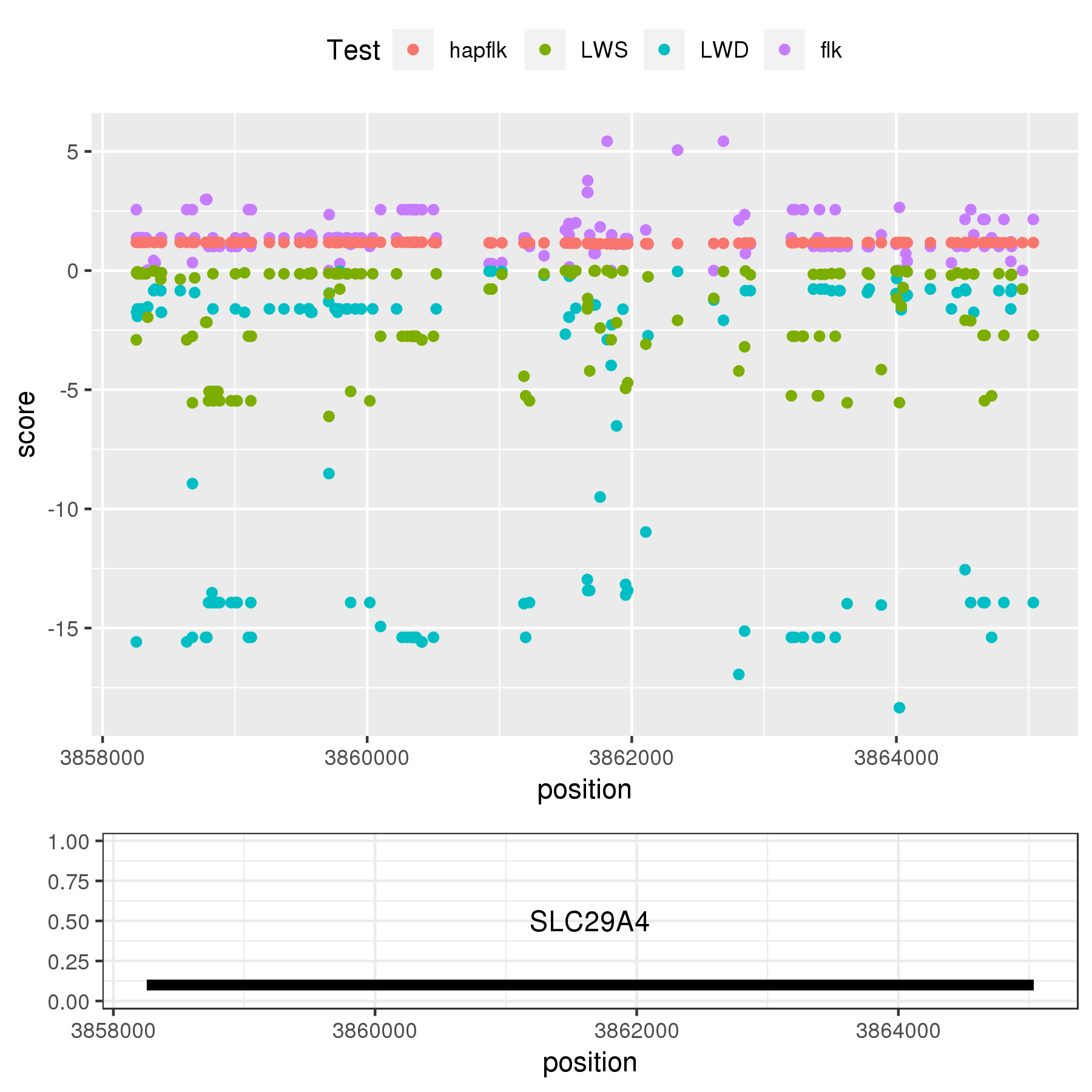

Supplement: Supplementary file 4 — Additional file 4. Summary plots of the scan for selection for all candidate regions of Table 1. Each file of this archive corresponds to a different candidate region, whose genomic position can be read in the file name. The top panel shows the results of the four tests for selection applied in the region. Values of the time-LWD (in blue), time-LWS (in green), hapFLK (in red) and FLK (in purple) tests are plotted (y axis) according to genomic position (x axis). Opposite signs are used for time-LWD and time-LWS on the one hand and hapFLK and FLK on the other hand, i.e. high test statistic values correspond to highly negative points for time-LWD and time-LWS and to highly positive points for hapFLK and FLK. All variants (AV set) were considered for these analyses. The bottom panel shows the position and name of genes located in the region. [file 12711_2023_789_MOESM4_ESM.xz › stat_profiles/all10_auto_chro3_3858255-3865035_scores.png]

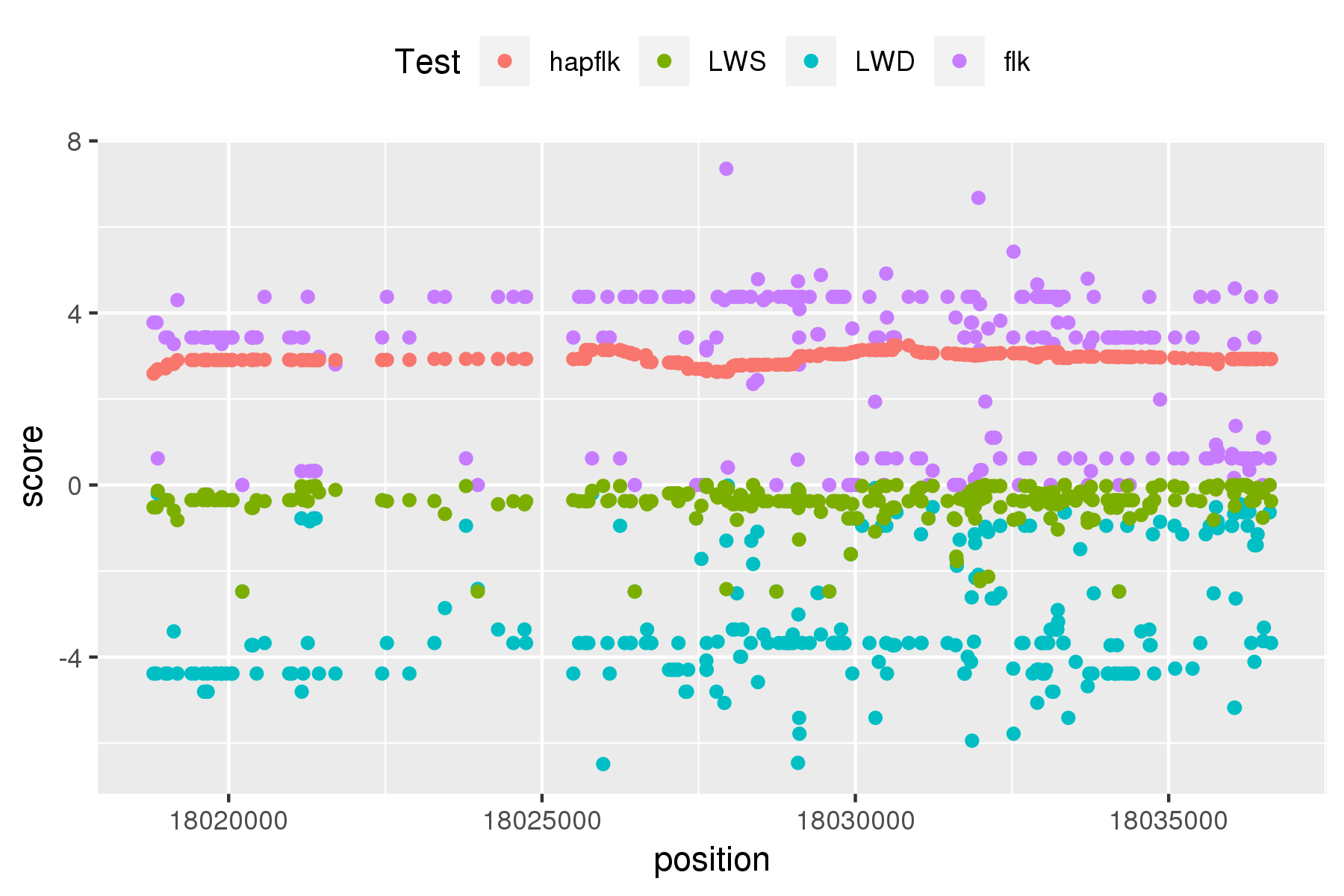

Supplement: Supplementary file 4 — Additional file 4. Summary plots of the scan for selection for all candidate regions of Table 1. Each file of this archive corresponds to a different candidate region, whose genomic position can be read in the file name. The top panel shows the results of the four tests for selection applied in the region. Values of the time-LWD (in blue), time-LWS (in green), hapFLK (in red) and FLK (in purple) tests are plotted (y axis) according to genomic position (x axis). Opposite signs are used for time-LWD and time-LWS on the one hand and hapFLK and FLK on the other hand, i.e. high test statistic values correspond to highly negative points for time-LWD and time-LWS and to highly positive points for hapFLK and FLK. All variants (AV set) were considered for these analyses. The bottom panel shows the position and name of genes located in the region. [file 12711_2023_789_MOESM4_ESM.xz › stat_profiles/all10_auto_chro4_18018801-18036634_scores.png]

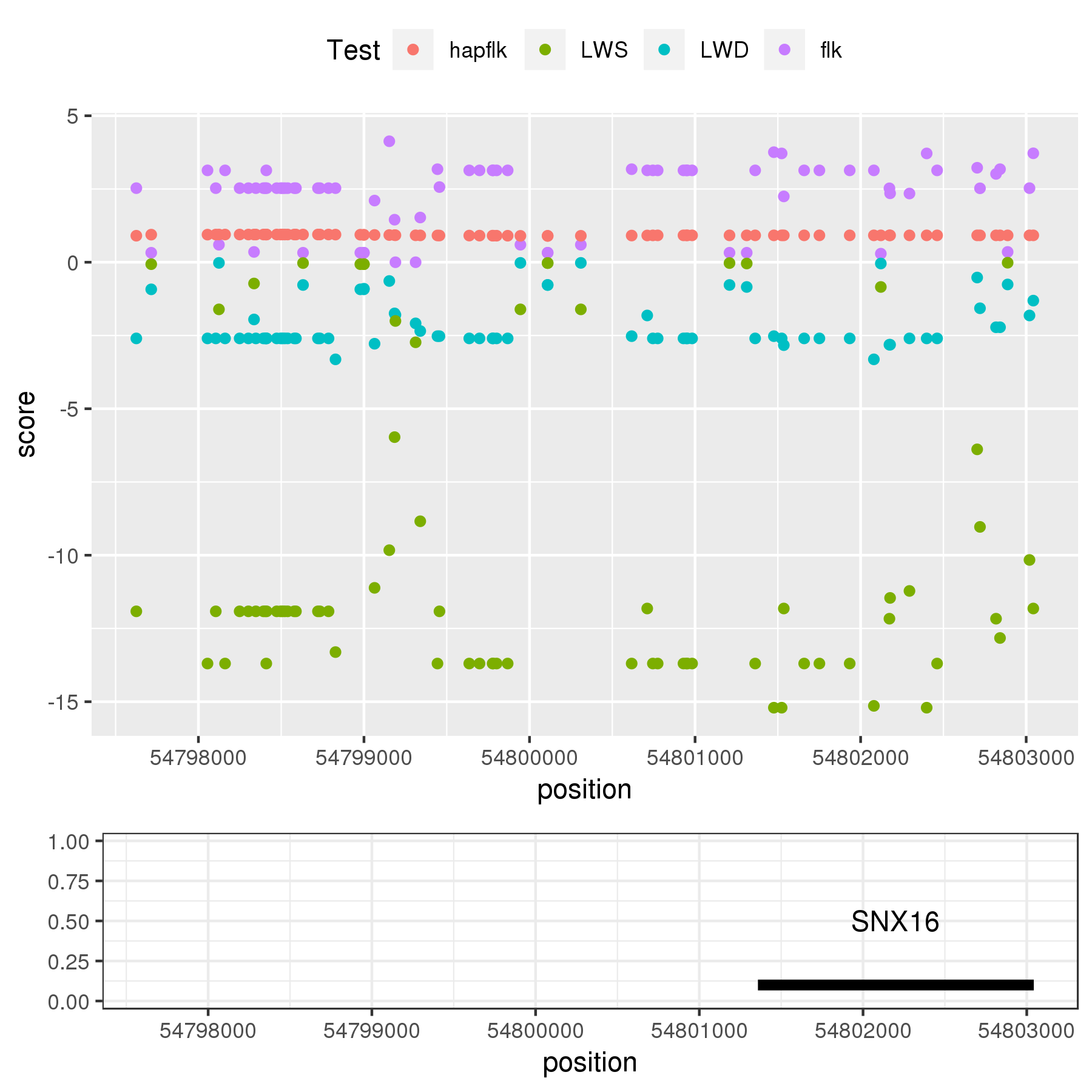

Supplement: Supplementary file 4 — Additional file 4. Summary plots of the scan for selection for all candidate regions of Table 1. Each file of this archive corresponds to a different candidate region, whose genomic position can be read in the file name. The top panel shows the results of the four tests for selection applied in the region. Values of the time-LWD (in blue), time-LWS (in green), hapFLK (in red) and FLK (in purple) tests are plotted (y axis) according to genomic position (x axis). Opposite signs are used for time-LWD and time-LWS on the one hand and hapFLK and FLK on the other hand, i.e. high test statistic values correspond to highly negative points for time-LWD and time-LWS and to highly positive points for hapFLK and FLK. All variants (AV set) were considered for these analyses. The bottom panel shows the position and name of genes located in the region. [file 12711_2023_789_MOESM4_ESM.xz › stat_profiles/all10_auto_chro4_54797625-54803043_scores.png]

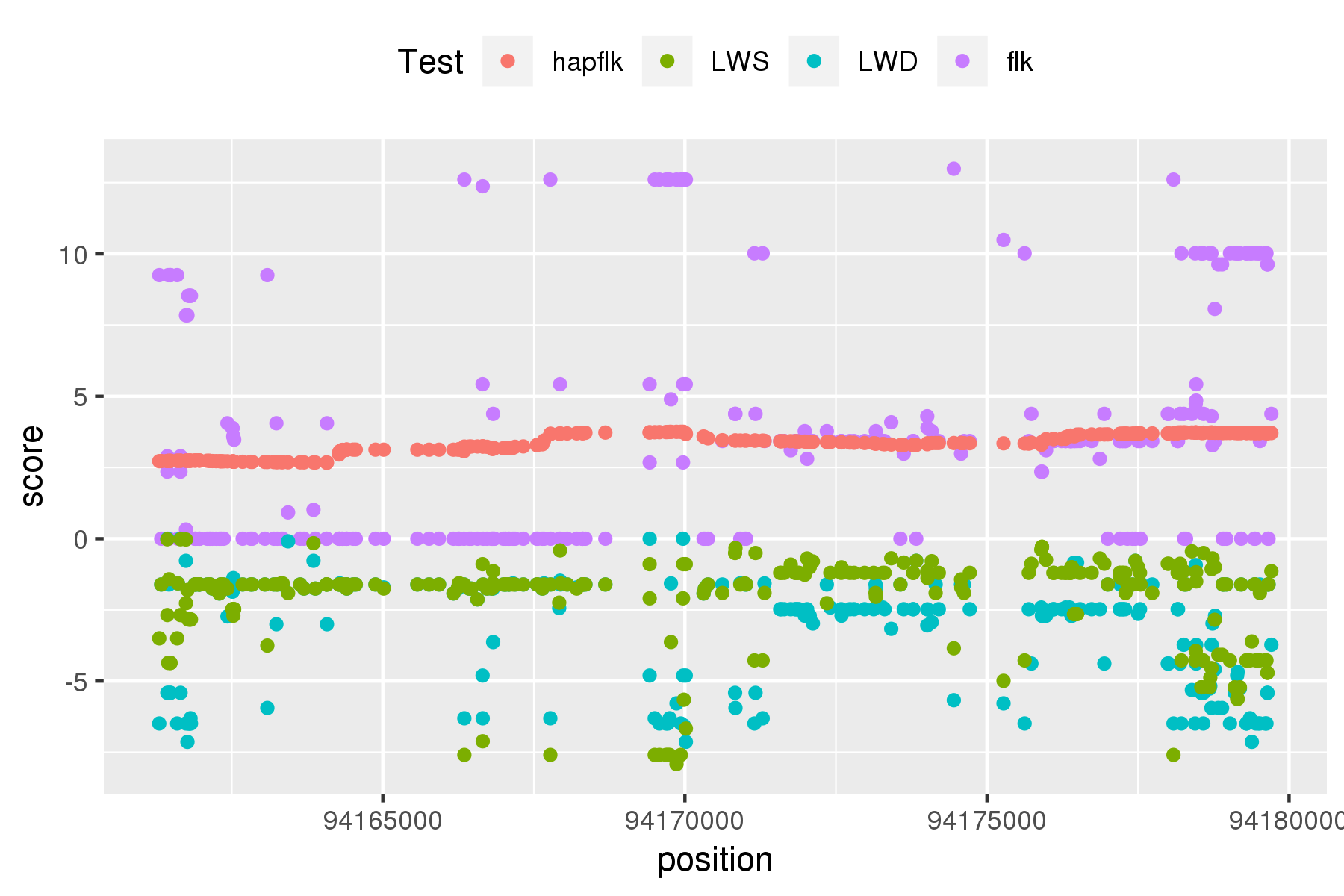

Supplement: Supplementary file 4 — Additional file 4. Summary plots of the scan for selection for all candidate regions of Table 1. Each file of this archive corresponds to a different candidate region, whose genomic position can be read in the file name. The top panel shows the results of the four tests for selection applied in the region. Values of the time-LWD (in blue), time-LWS (in green), hapFLK (in red) and FLK (in purple) tests are plotted (y axis) according to genomic position (x axis). Opposite signs are used for time-LWD and time-LWS on the one hand and hapFLK and FLK on the other hand, i.e. high test statistic values correspond to highly negative points for time-LWD and time-LWS and to highly positive points for hapFLK and FLK. All variants (AV set) were considered for these analyses. The bottom panel shows the position and name of genes located in the region. [file 12711_2023_789_MOESM4_ESM.xz › stat_profiles/all10_auto_chro5_94161299-94179707_scores.png]

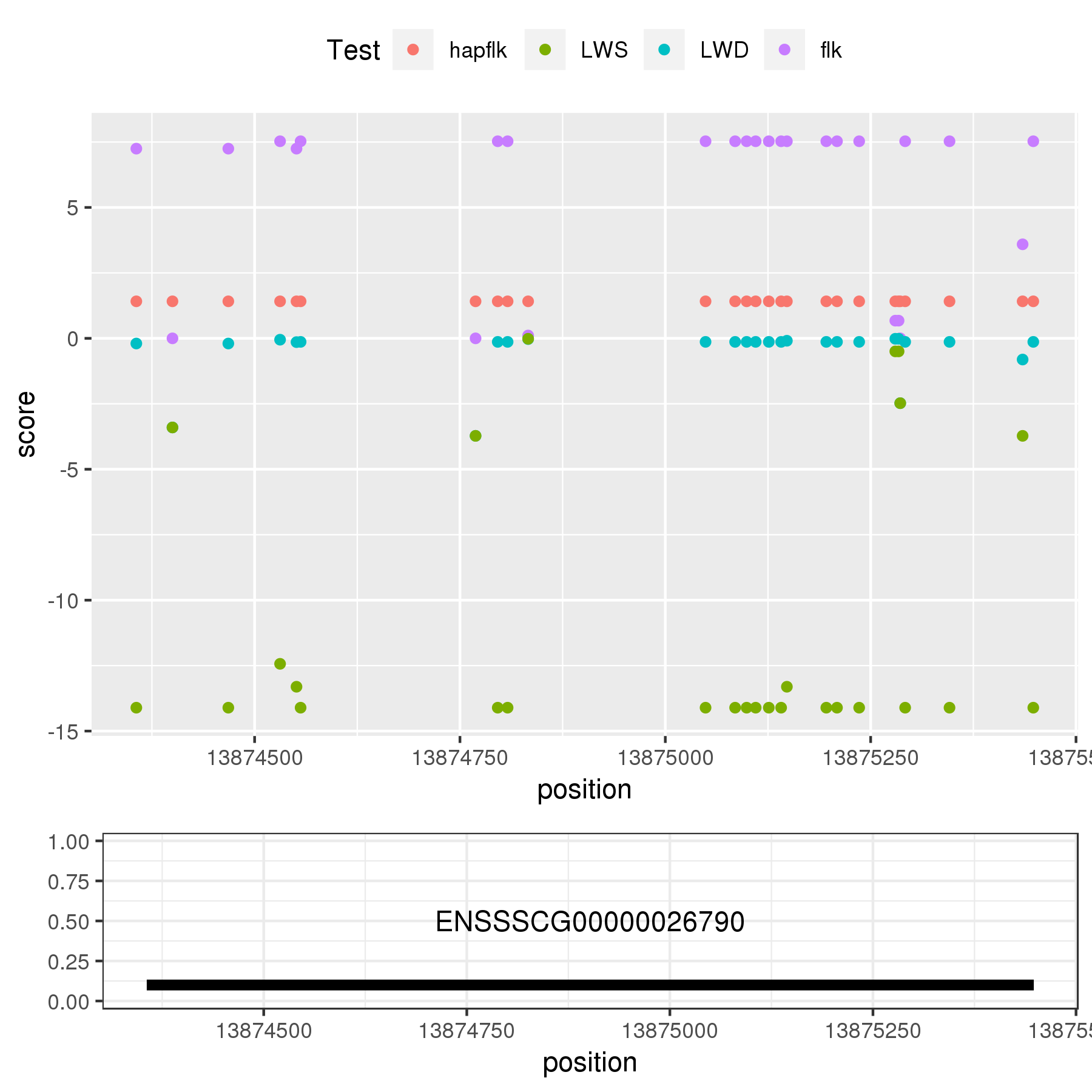

Supplement: Supplementary file 4 — Additional file 4. Summary plots of the scan for selection for all candidate regions of Table 1. Each file of this archive corresponds to a different candidate region, whose genomic position can be read in the file name. The top panel shows the results of the four tests for selection applied in the region. Values of the time-LWD (in blue), time-LWS (in green), hapFLK (in red) and FLK (in purple) tests are plotted (y axis) according to genomic position (x axis). Opposite signs are used for time-LWD and time-LWS on the one hand and hapFLK and FLK on the other hand, i.e. high test statistic values correspond to highly negative points for time-LWD and time-LWS and to highly positive points for hapFLK and FLK. All variants (AV set) were considered for these analyses. The bottom panel shows the position and name of genes located in the region. [file 12711_2023_789_MOESM4_ESM.xz › stat_profiles/all10_auto_chro6_13874356-13875448_scores.png]

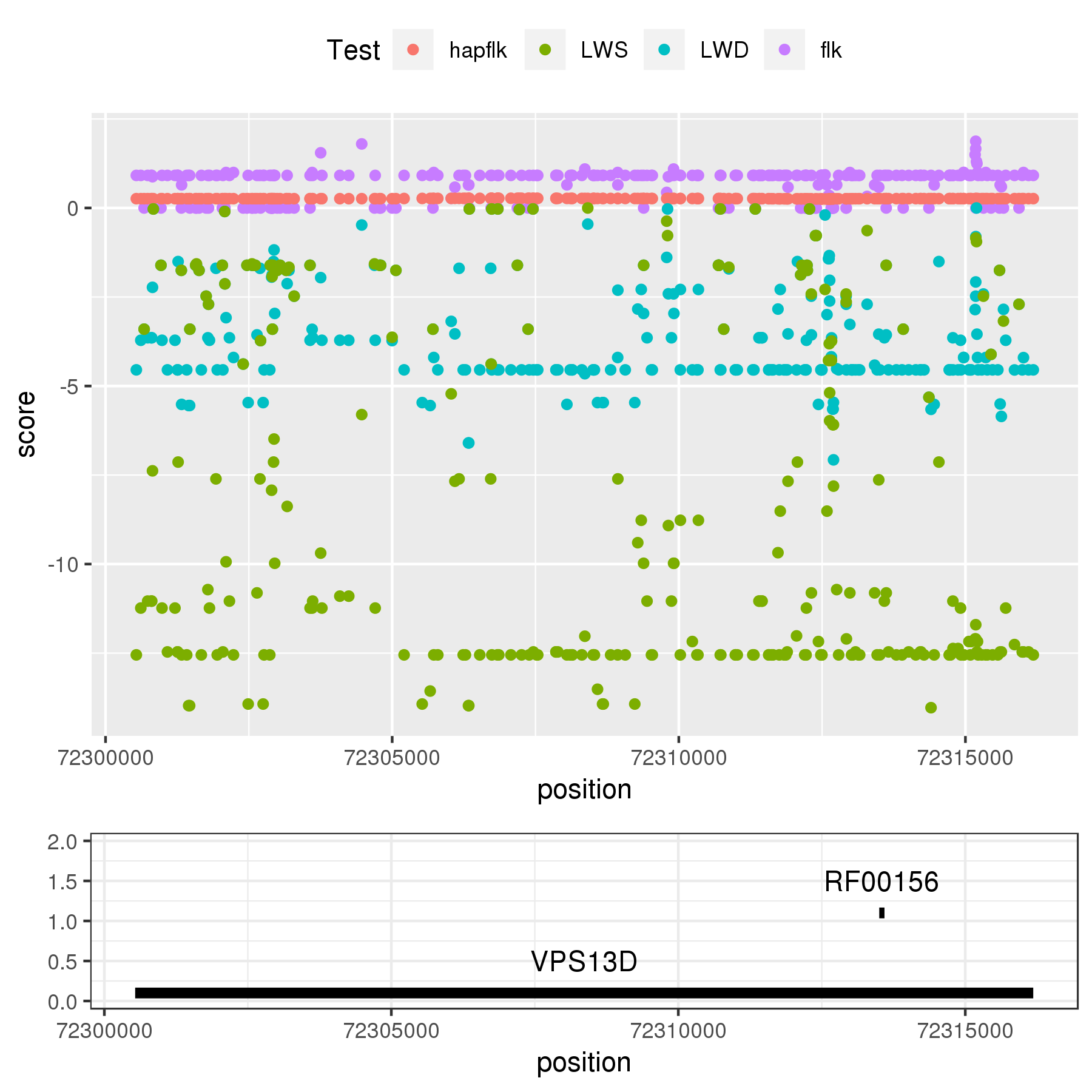

Supplement: Supplementary file 4 — Additional file 4. Summary plots of the scan for selection for all candidate regions of Table 1. Each file of this archive corresponds to a different candidate region, whose genomic position can be read in the file name. The top panel shows the results of the four tests for selection applied in the region. Values of the time-LWD (in blue), time-LWS (in green), hapFLK (in red) and FLK (in purple) tests are plotted (y axis) according to genomic position (x axis). Opposite signs are used for time-LWD and time-LWS on the one hand and hapFLK and FLK on the other hand, i.e. high test statistic values correspond to highly negative points for time-LWD and time-LWS and to highly positive points for hapFLK and FLK. All variants (AV set) were considered for these analyses. The bottom panel shows the position and name of genes located in the region. [file 12711_2023_789_MOESM4_ESM.xz › stat_profiles/all10_auto_chro6_72300537-72316184_scores.png]

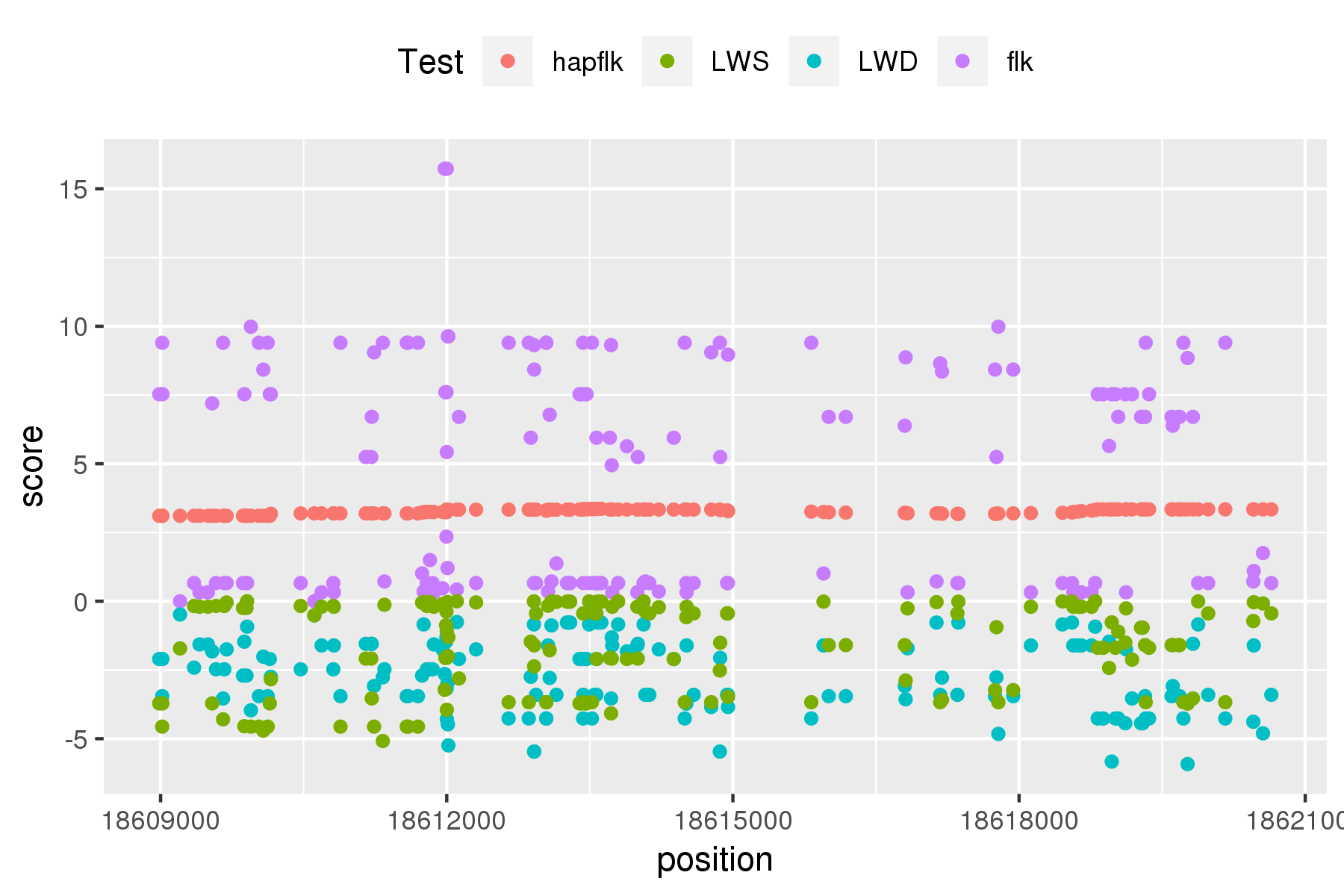

Supplement: Supplementary file 4 — Additional file 4. Summary plots of the scan for selection for all candidate regions of Table 1. Each file of this archive corresponds to a different candidate region, whose genomic position can be read in the file name. The top panel shows the results of the four tests for selection applied in the region. Values of the time-LWD (in blue), time-LWS (in green), hapFLK (in red) and FLK (in purple) tests are plotted (y axis) according to genomic position (x axis). Opposite signs are used for time-LWD and time-LWS on the one hand and hapFLK and FLK on the other hand, i.e. high test statistic values correspond to highly negative points for time-LWD and time-LWS and to highly positive points for hapFLK and FLK. All variants (AV set) were considered for these analyses. The bottom panel shows the position and name of genes located in the region. [file 12711_2023_789_MOESM4_ESM.xz › stat_profiles/all10_auto_chro7_18608986-18620645_scores.png]

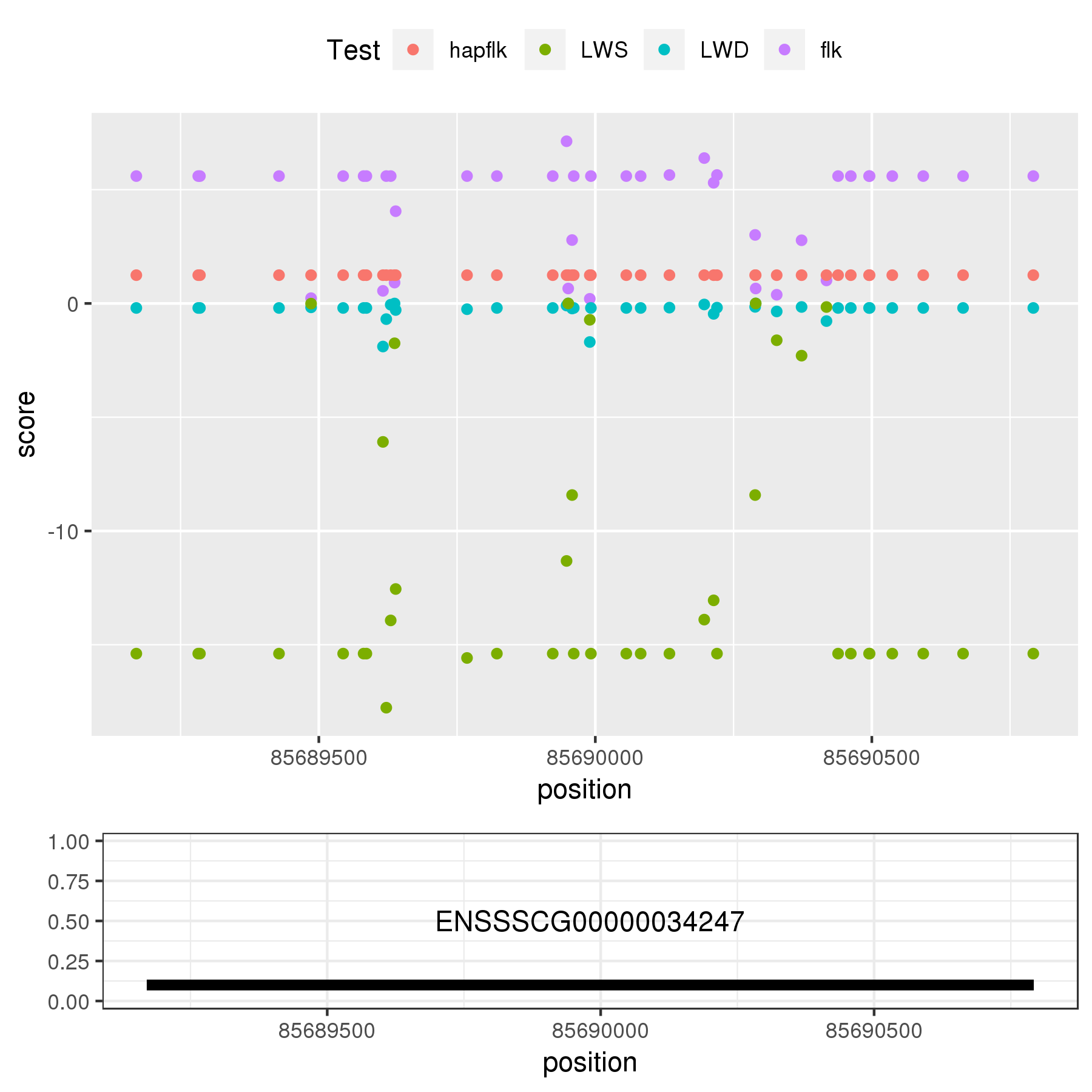

Supplement: Supplementary file 4 — Additional file 4. Summary plots of the scan for selection for all candidate regions of Table 1. Each file of this archive corresponds to a different candidate region, whose genomic position can be read in the file name. The top panel shows the results of the four tests for selection applied in the region. Values of the time-LWD (in blue), time-LWS (in green), hapFLK (in red) and FLK (in purple) tests are plotted (y axis) according to genomic position (x axis). Opposite signs are used for time-LWD and time-LWS on the one hand and hapFLK and FLK on the other hand, i.e. high test statistic values correspond to highly negative points for time-LWD and time-LWS and to highly positive points for hapFLK and FLK. All variants (AV set) were considered for these analyses. The bottom panel shows the position and name of genes located in the region. [file 12711_2023_789_MOESM4_ESM.xz › stat_profiles/all10_auto_chro7_85689170-85690792_scores.png]

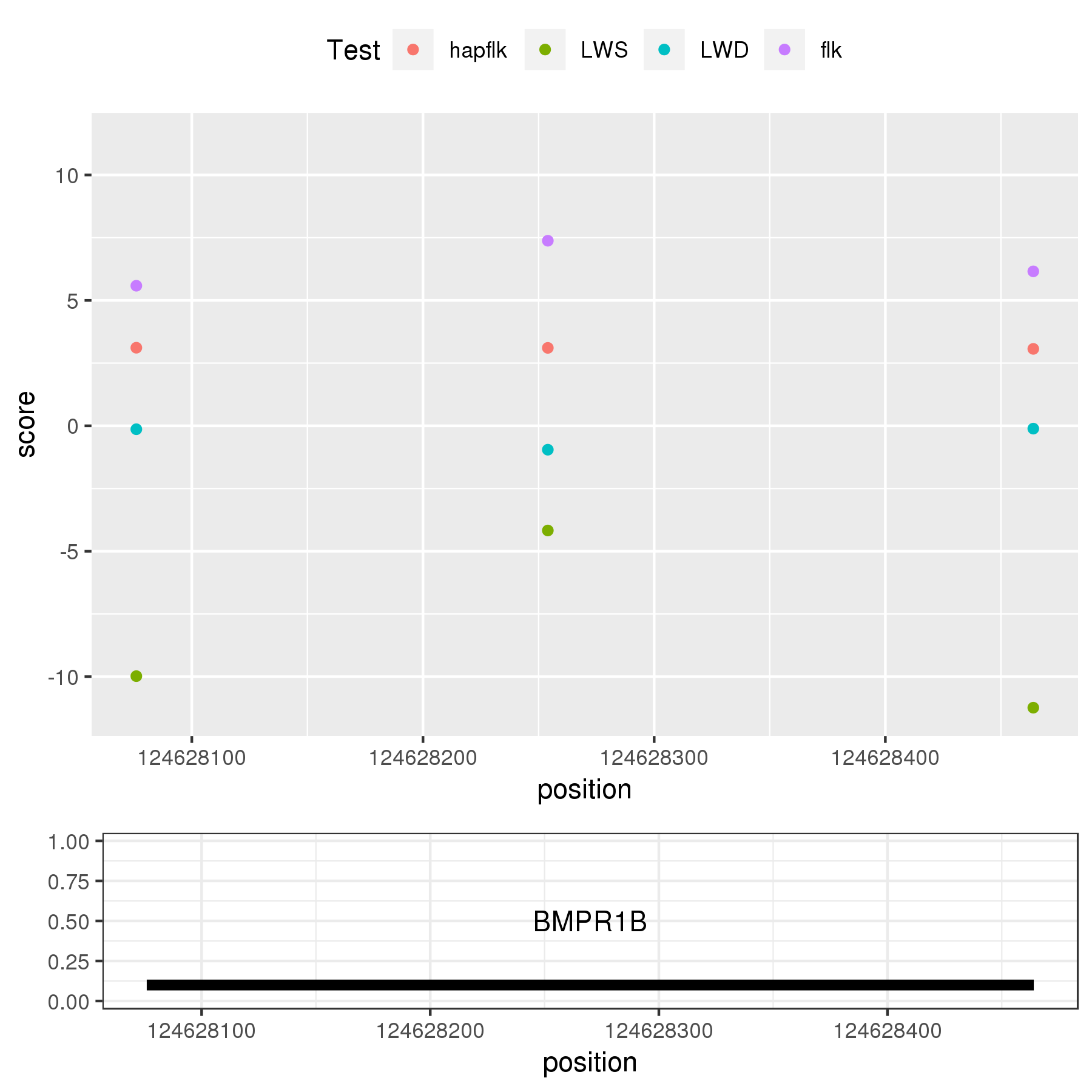

Supplement: Supplementary file 4 — Additional file 4. Summary plots of the scan for selection for all candidate regions of Table 1. Each file of this archive corresponds to a different candidate region, whose genomic position can be read in the file name. The top panel shows the results of the four tests for selection applied in the region. Values of the time-LWD (in blue), time-LWS (in green), hapFLK (in red) and FLK (in purple) tests are plotted (y axis) according to genomic position (x axis). Opposite signs are used for time-LWD and time-LWS on the one hand and hapFLK and FLK on the other hand, i.e. high test statistic values correspond to highly negative points for time-LWD and time-LWS and to highly positive points for hapFLK and FLK. All variants (AV set) were considered for these analyses. The bottom panel shows the position and name of genes located in the region. [file 12711_2023_789_MOESM4_ESM.xz › stat_profiles/all10_auto_chro8_124628076-124628464_scores.png]

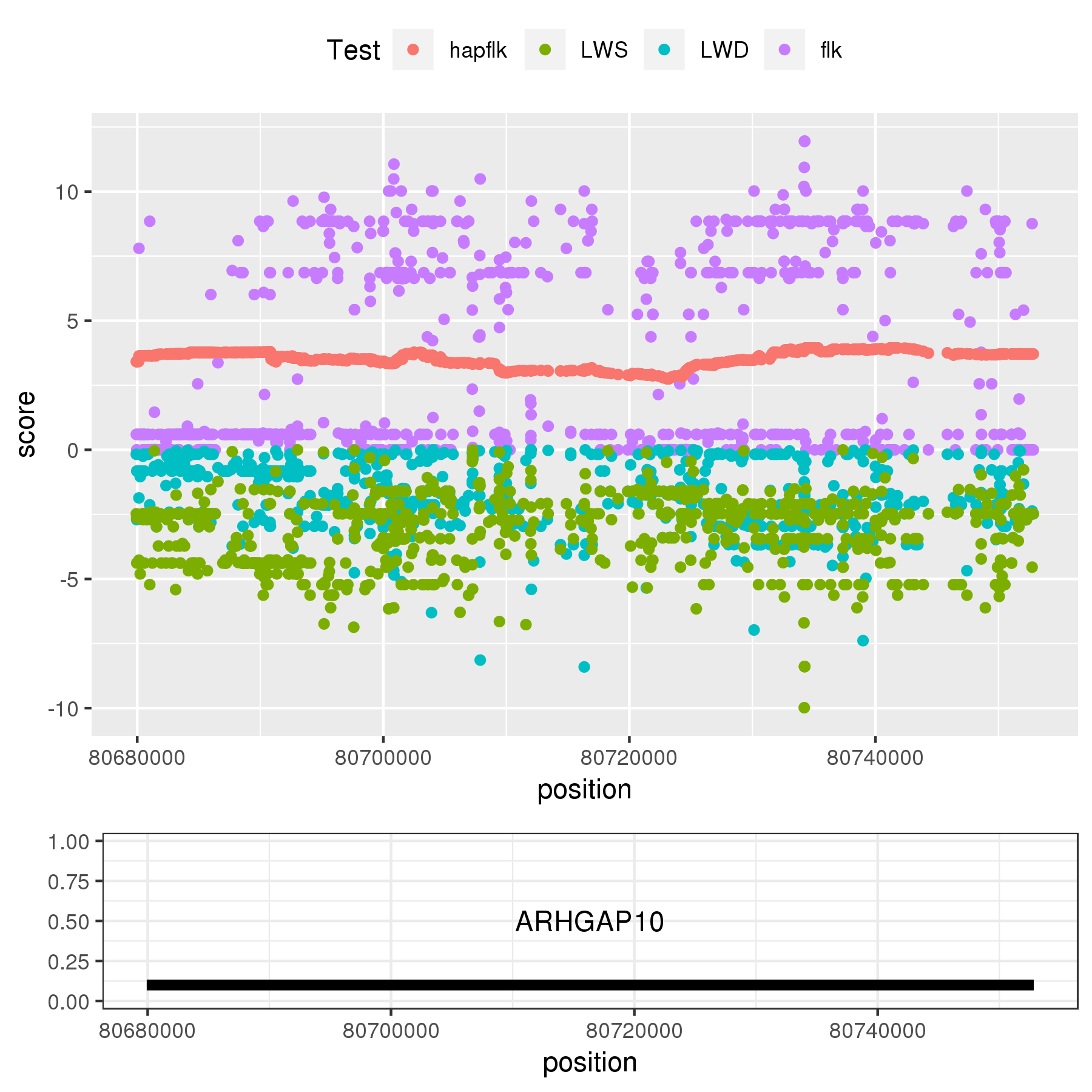

Supplement: Supplementary file 4 — Additional file 4. Summary plots of the scan for selection for all candidate regions of Table 1. Each file of this archive corresponds to a different candidate region, whose genomic position can be read in the file name. The top panel shows the results of the four tests for selection applied in the region. Values of the time-LWD (in blue), time-LWS (in green), hapFLK (in red) and FLK (in purple) tests are plotted (y axis) according to genomic position (x axis). Opposite signs are used for time-LWD and time-LWS on the one hand and hapFLK and FLK on the other hand, i.e. high test statistic values correspond to highly negative points for time-LWD and time-LWS and to highly positive points for hapFLK and FLK. All variants (AV set) were considered for these analyses. The bottom panel shows the position and name of genes located in the region. [file 12711_2023_789_MOESM4_ESM.xz › stat_profiles/all10_auto_chro8_80679925-80752831_scores.png]

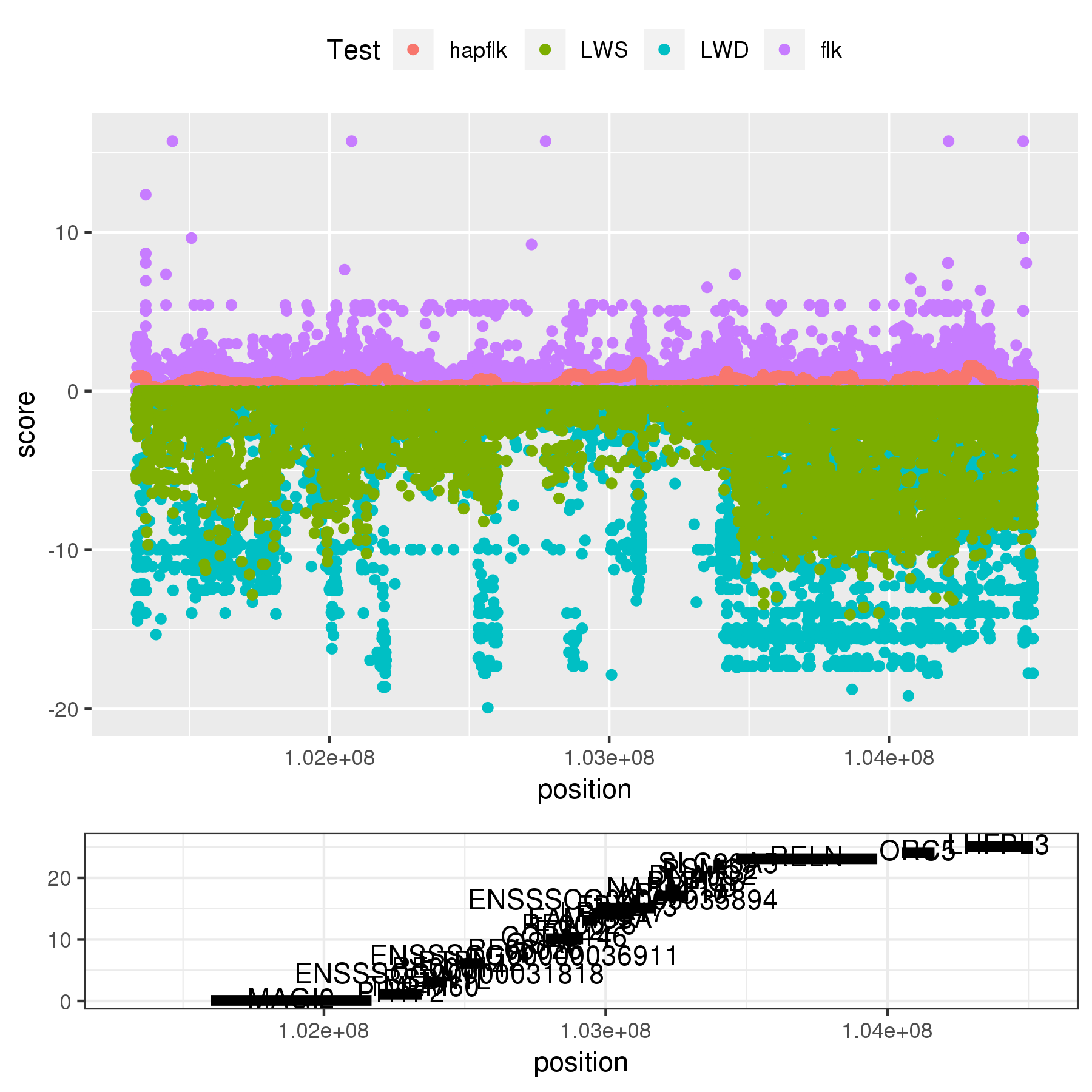

Supplement: Supplementary file 4 — Additional file 4. Summary plots of the scan for selection for all candidate regions of Table 1. Each file of this archive corresponds to a different candidate region, whose genomic position can be read in the file name. The top panel shows the results of the four tests for selection applied in the region. Values of the time-LWD (in blue), time-LWS (in green), hapFLK (in red) and FLK (in purple) tests are plotted (y axis) according to genomic position (x axis). Opposite signs are used for time-LWD and time-LWS on the one hand and hapFLK and FLK on the other hand, i.e. high test statistic values correspond to highly negative points for time-LWD and time-LWS and to highly positive points for hapFLK and FLK. All variants (AV set) were considered for these analyses. The bottom panel shows the position and name of genes located in the region. [file 12711_2023_789_MOESM4_ESM.xz › stat_profiles/all10_auto_chro9_101309610-104516170_scores.png]

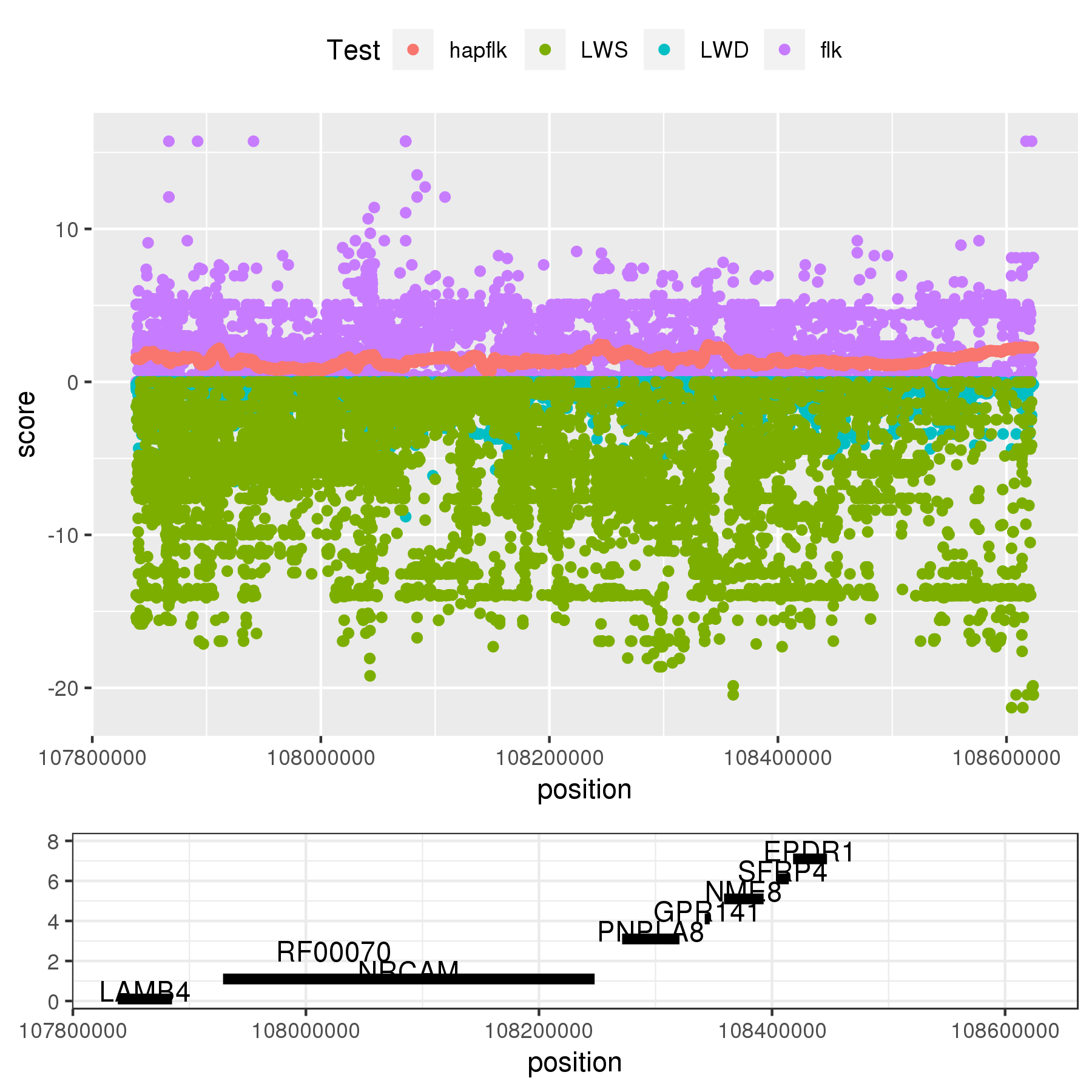

Supplement: Supplementary file 4 — Additional file 4. Summary plots of the scan for selection for all candidate regions of Table 1. Each file of this archive corresponds to a different candidate region, whose genomic position can be read in the file name. The top panel shows the results of the four tests for selection applied in the region. Values of the time-LWD (in blue), time-LWS (in green), hapFLK (in red) and FLK (in purple) tests are plotted (y axis) according to genomic position (x axis). Opposite signs are used for time-LWD and time-LWS on the one hand and hapFLK and FLK on the other hand, i.e. high test statistic values correspond to highly negative points for time-LWD and time-LWS and to highly positive points for hapFLK and FLK. All variants (AV set) were considered for these analyses. The bottom panel shows the position and name of genes located in the region. [file 12711_2023_789_MOESM4_ESM.xz › stat_profiles/all10_auto_chro9_107838551-108623449_scores.png]

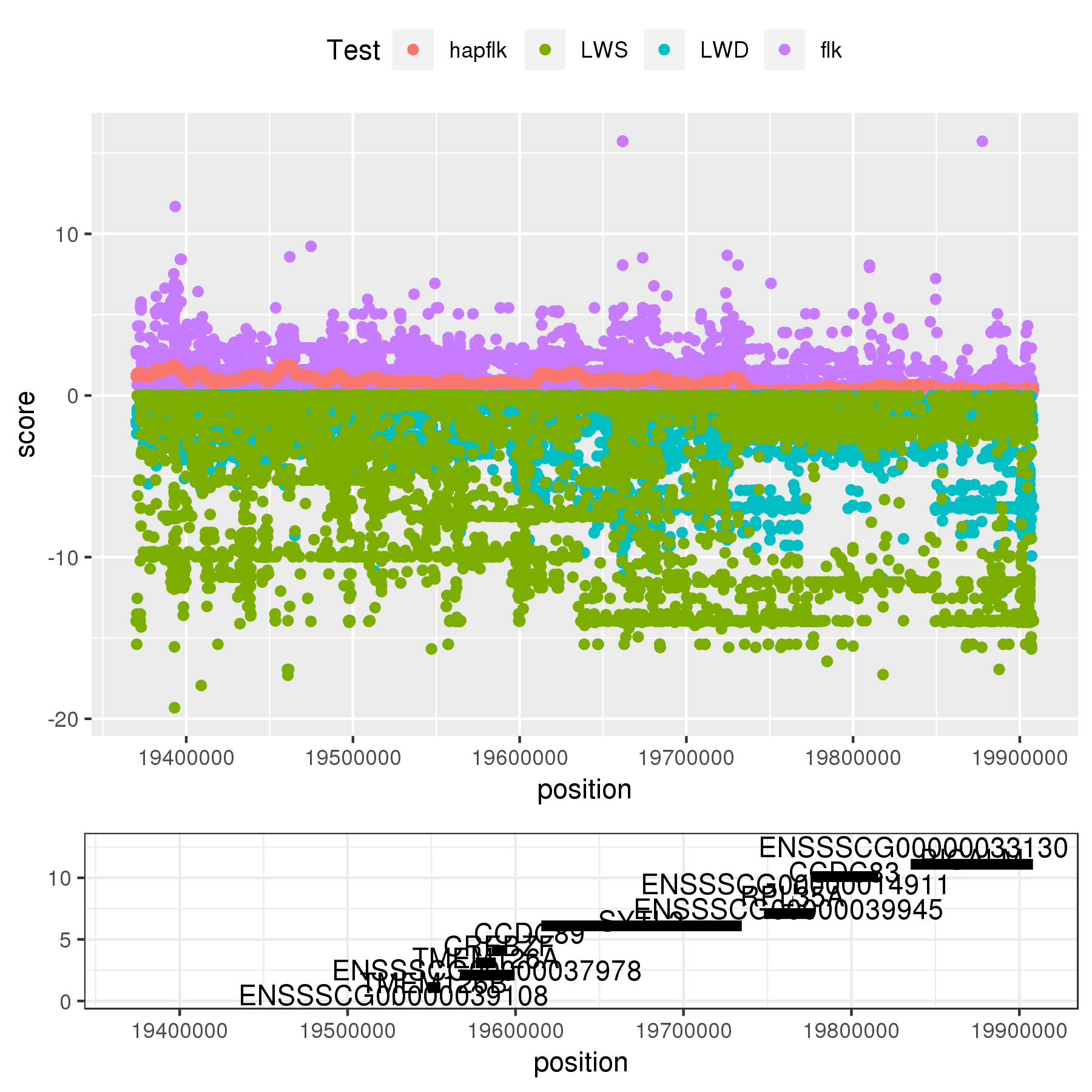

Supplement: Supplementary file 4 — Additional file 4. Summary plots of the scan for selection for all candidate regions of Table 1. Each file of this archive corresponds to a different candidate region, whose genomic position can be read in the file name. The top panel shows the results of the four tests for selection applied in the region. Values of the time-LWD (in blue), time-LWS (in green), hapFLK (in red) and FLK (in purple) tests are plotted (y axis) according to genomic position (x axis). Opposite signs are used for time-LWD and time-LWS on the one hand and hapFLK and FLK on the other hand, i.e. high test statistic values correspond to highly negative points for time-LWD and time-LWS and to highly positive points for hapFLK and FLK. All variants (AV set) were considered for these analyses. The bottom panel shows the position and name of genes located in the region. [file 12711_2023_789_MOESM4_ESM.xz › stat_profiles/all10_auto_chro9_19370051-19908097_scores.png]

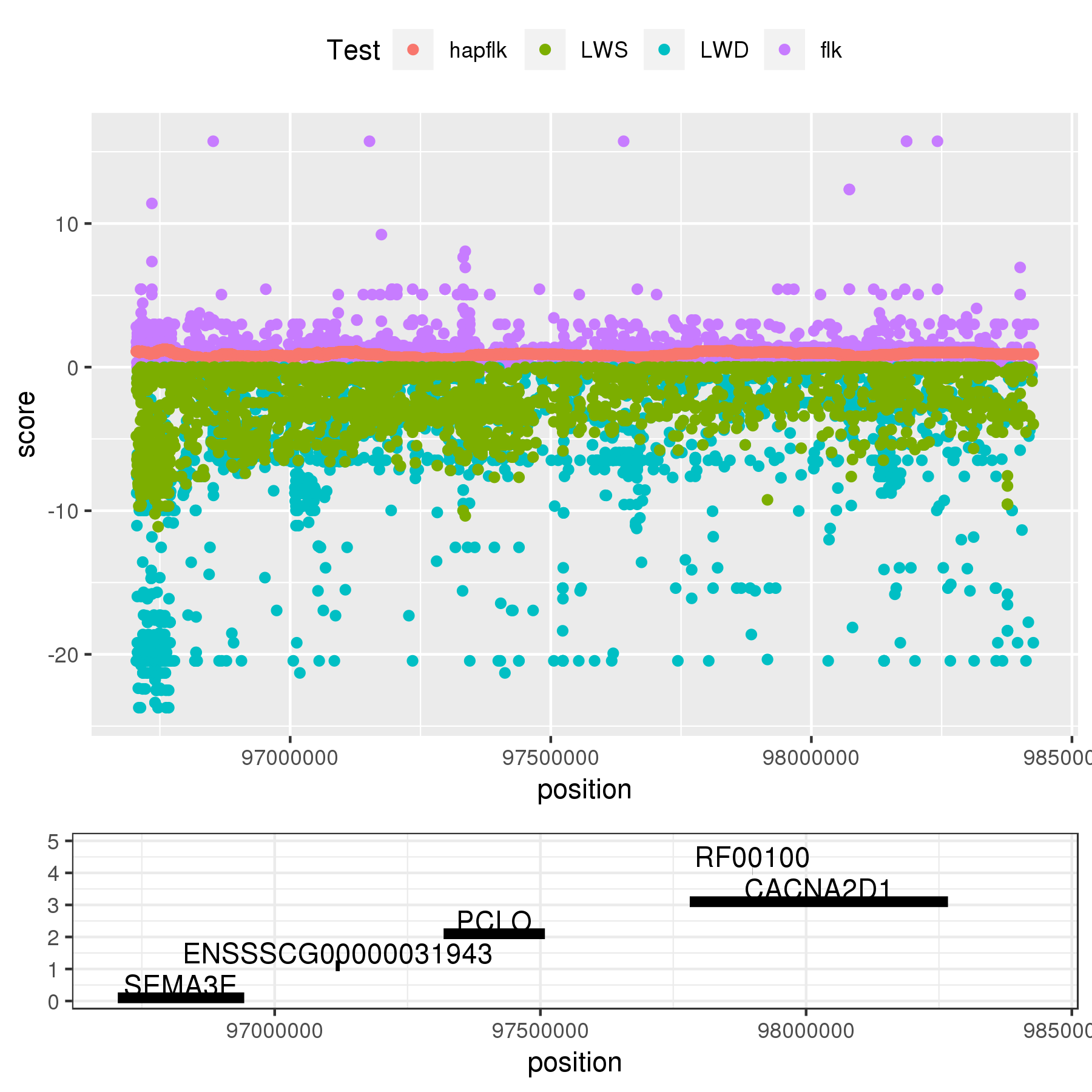

Supplement: Supplementary file 4 — Additional file 4. Summary plots of the scan for selection for all candidate regions of Table 1. Each file of this archive corresponds to a different candidate region, whose genomic position can be read in the file name. The top panel shows the results of the four tests for selection applied in the region. Values of the time-LWD (in blue), time-LWS (in green), hapFLK (in red) and FLK (in purple) tests are plotted (y axis) according to genomic position (x axis). Opposite signs are used for time-LWD and time-LWS on the one hand and hapFLK and FLK on the other hand, i.e. high test statistic values correspond to highly negative points for time-LWD and time-LWS and to highly positive points for hapFLK and FLK. All variants (AV set) were considered for these analyses. The bottom panel shows the position and name of genes located in the region. [file 12711_2023_789_MOESM4_ESM.xz › stat_profiles/all10_auto_chro9_96704786-98425852_scores.png]

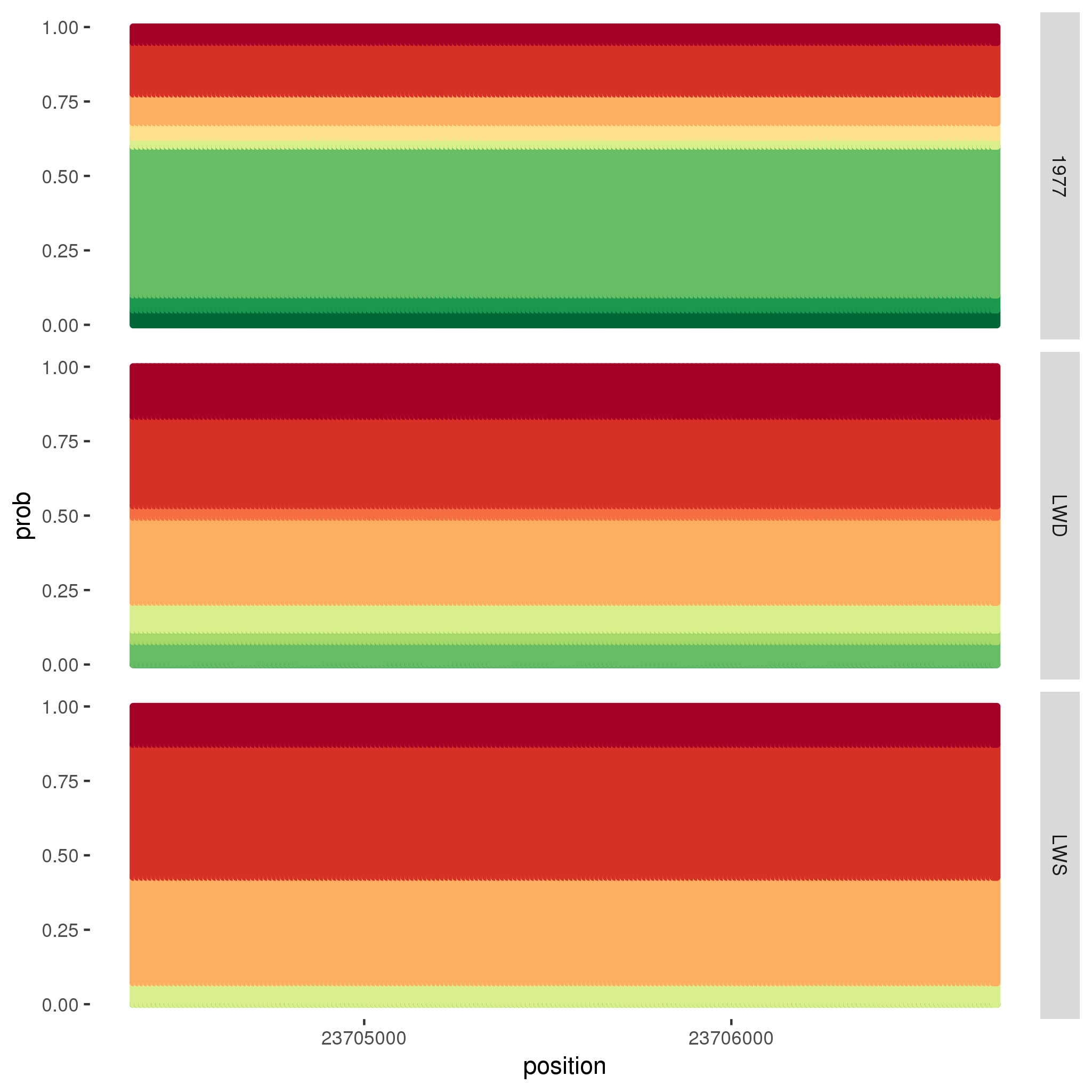

Supplement: Supplementary file 5 — Additional file 5. Haplotype frequency plots for all candidate regions of Table 1. Each file of this archive corresponds to a different candidate region, whose genomic position can be read in the file name. It shows the haplotype diversity in this region, estimated using hapFLK with 10 haplotype clusters (for one single EM run). For a given genomic position, each color corresponds to a different haplotype cluster and the height of the color band in a given population (represented by one of the panels) gives the frequency of the cluster in this population. All variants (AV set) were considered for these analyses. [file 12711_2023_789_MOESM5_ESM.xz › hapflk_plots/all10_auto_chro10_23704376-23706718.kfrq.fit_0.bz2-plot.png]

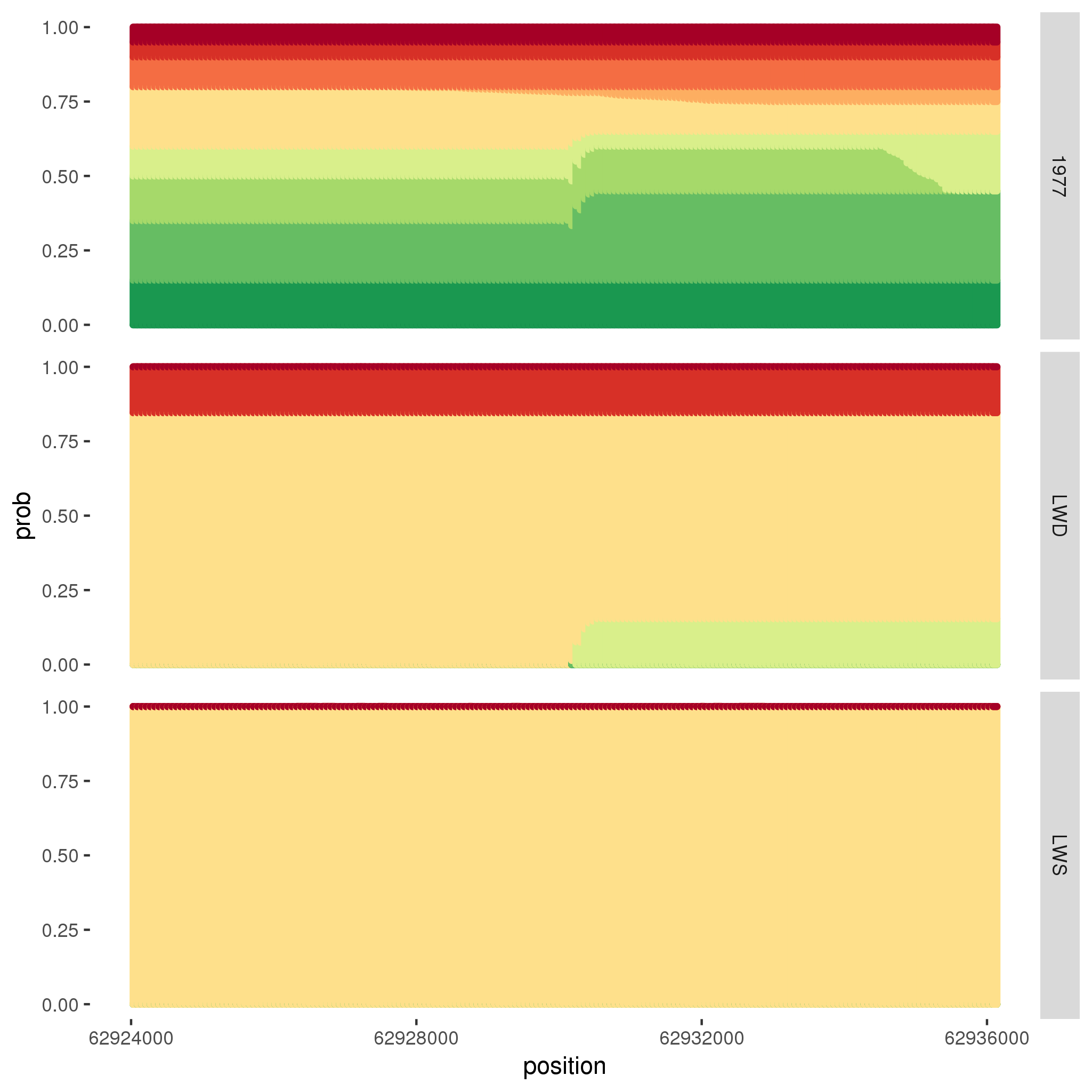

Supplement: Supplementary file 5 — Additional file 5. Haplotype frequency plots for all candidate regions of Table 1. Each file of this archive corresponds to a different candidate region, whose genomic position can be read in the file name. It shows the haplotype diversity in this region, estimated using hapFLK with 10 haplotype clusters (for one single EM run). For a given genomic position, each color corresponds to a different haplotype cluster and the height of the color band in a given population (represented by one of the panels) gives the frequency of the cluster in this population. All variants (AV set) were considered for these analyses. [file 12711_2023_789_MOESM5_ESM.xz › hapflk_plots/all10_auto_chro10_62924056-62936112.kfrq.fit_1.bz2-plot.png]

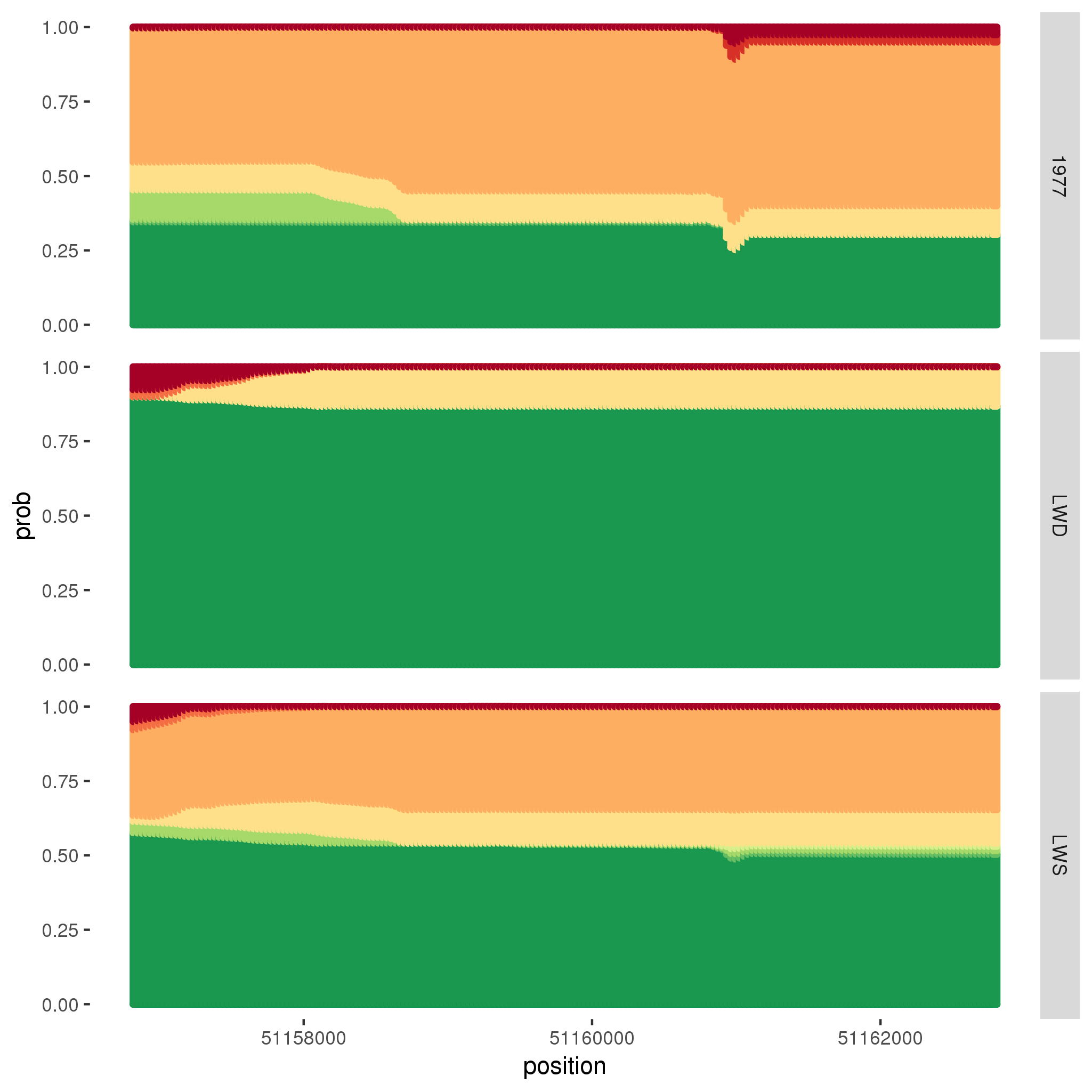

Supplement: Supplementary file 5 — Additional file 5. Haplotype frequency plots for all candidate regions of Table 1. Each file of this archive corresponds to a different candidate region, whose genomic position can be read in the file name. It shows the haplotype diversity in this region, estimated using hapFLK with 10 haplotype clusters (for one single EM run). For a given genomic position, each color corresponds to a different haplotype cluster and the height of the color band in a given population (represented by one of the panels) gives the frequency of the cluster in this population. All variants (AV set) were considered for these analyses. [file 12711_2023_789_MOESM5_ESM.xz › hapflk_plots/all10_auto_chro11_51156831-51162794.kfrq.fit_1.bz2-plot.png]

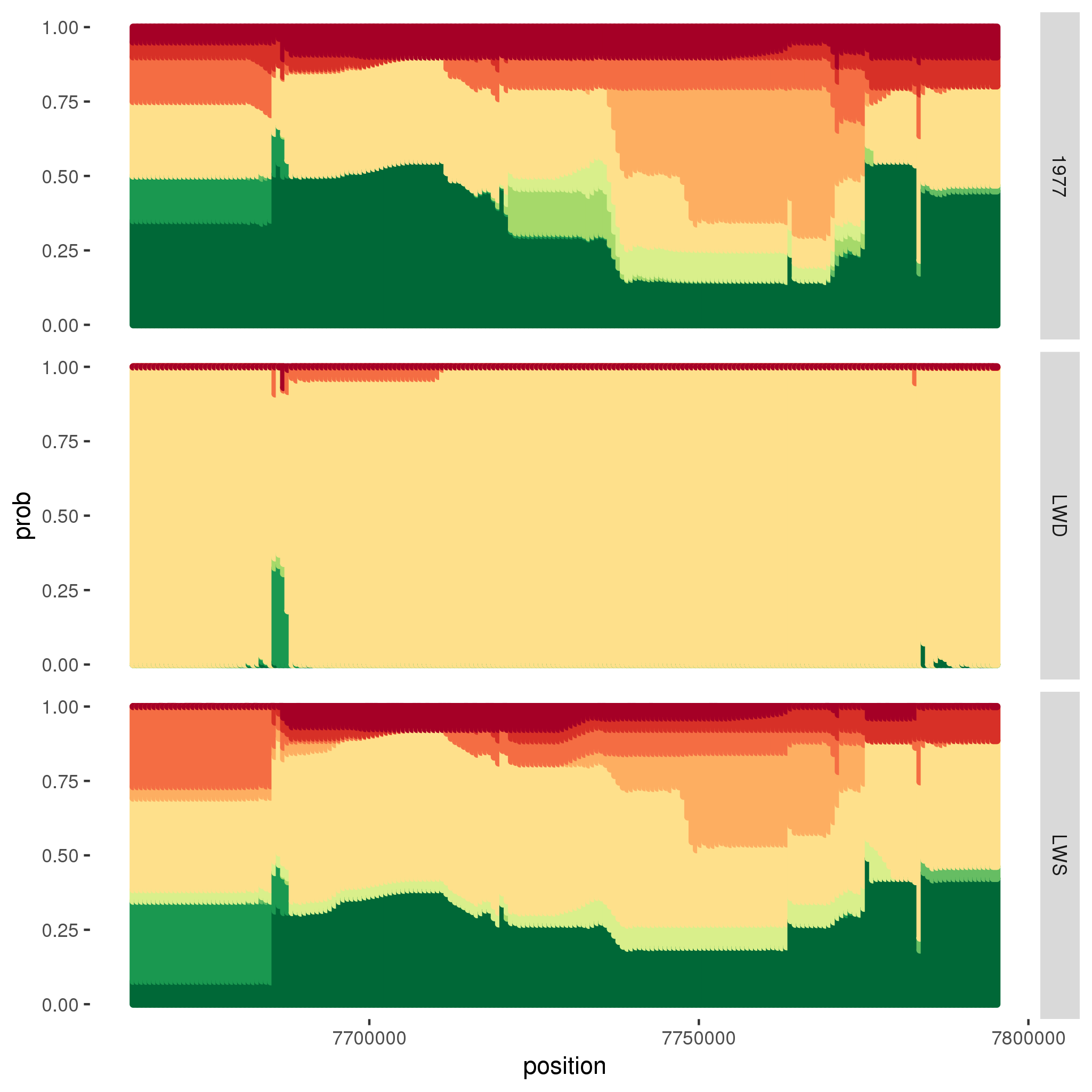

Supplement: Supplementary file 5 — Additional file 5. Haplotype frequency plots for all candidate regions of Table 1. Each file of this archive corresponds to a different candidate region, whose genomic position can be read in the file name. It shows the haplotype diversity in this region, estimated using hapFLK with 10 haplotype clusters (for one single EM run). For a given genomic position, each color corresponds to a different haplotype cluster and the height of the color band in a given population (represented by one of the panels) gives the frequency of the cluster in this population. All variants (AV set) were considered for these analyses. [file 12711_2023_789_MOESM5_ESM.xz › hapflk_plots/all10_auto_chro11_7664458-7794933.kfrq.fit_0.bz2-plot.png]

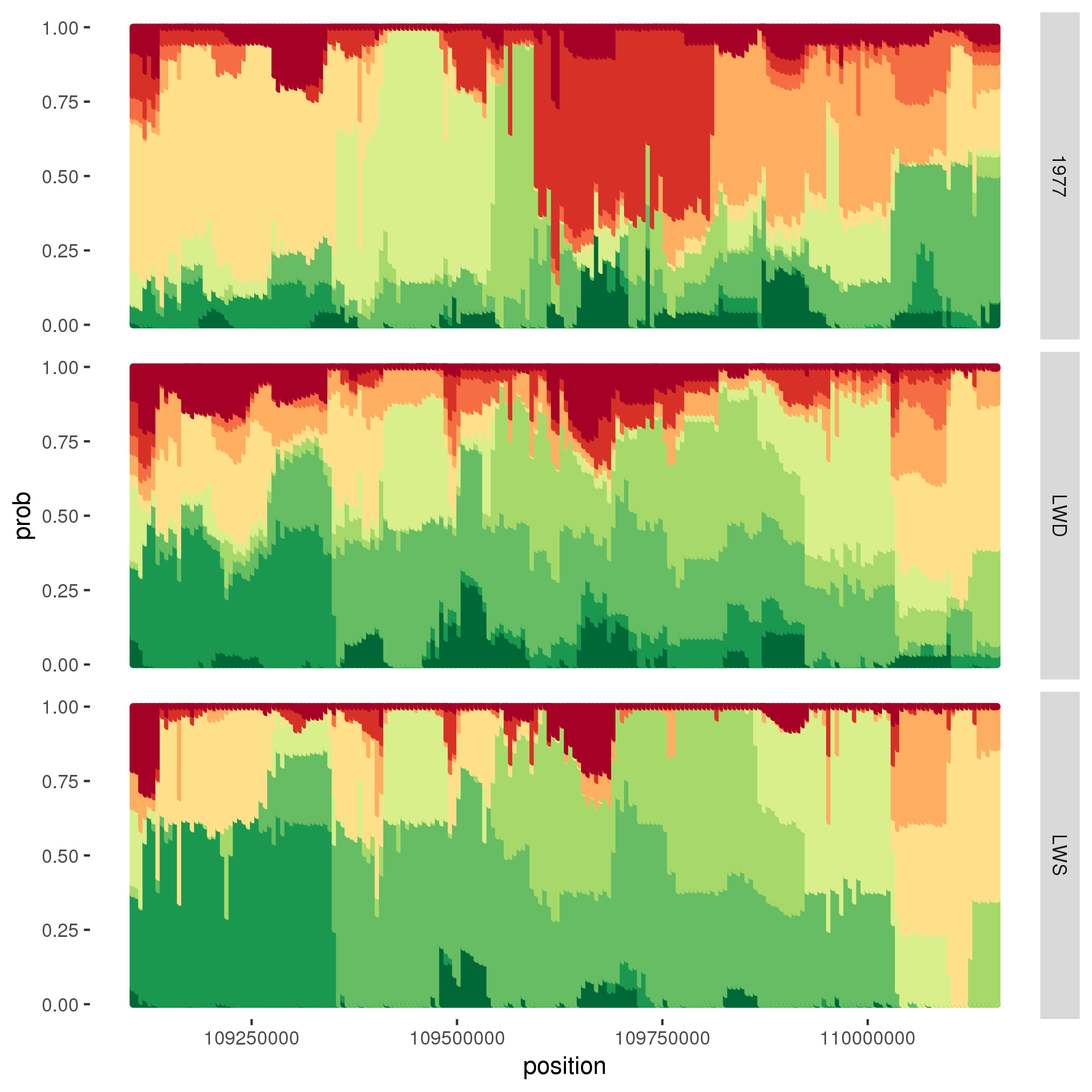

Supplement: Supplementary file 5 — Additional file 5. Haplotype frequency plots for all candidate regions of Table 1. Each file of this archive corresponds to a different candidate region, whose genomic position can be read in the file name. It shows the haplotype diversity in this region, estimated using hapFLK with 10 haplotype clusters (for one single EM run). For a given genomic position, each color corresponds to a different haplotype cluster and the height of the color band in a given population (represented by one of the panels) gives the frequency of the cluster in this population. All variants (AV set) were considered for these analyses. [file 12711_2023_789_MOESM5_ESM.xz › hapflk_plots/all10_auto_chro13_109108079-110154951.kfrq.fit_0.bz2-plot.png]

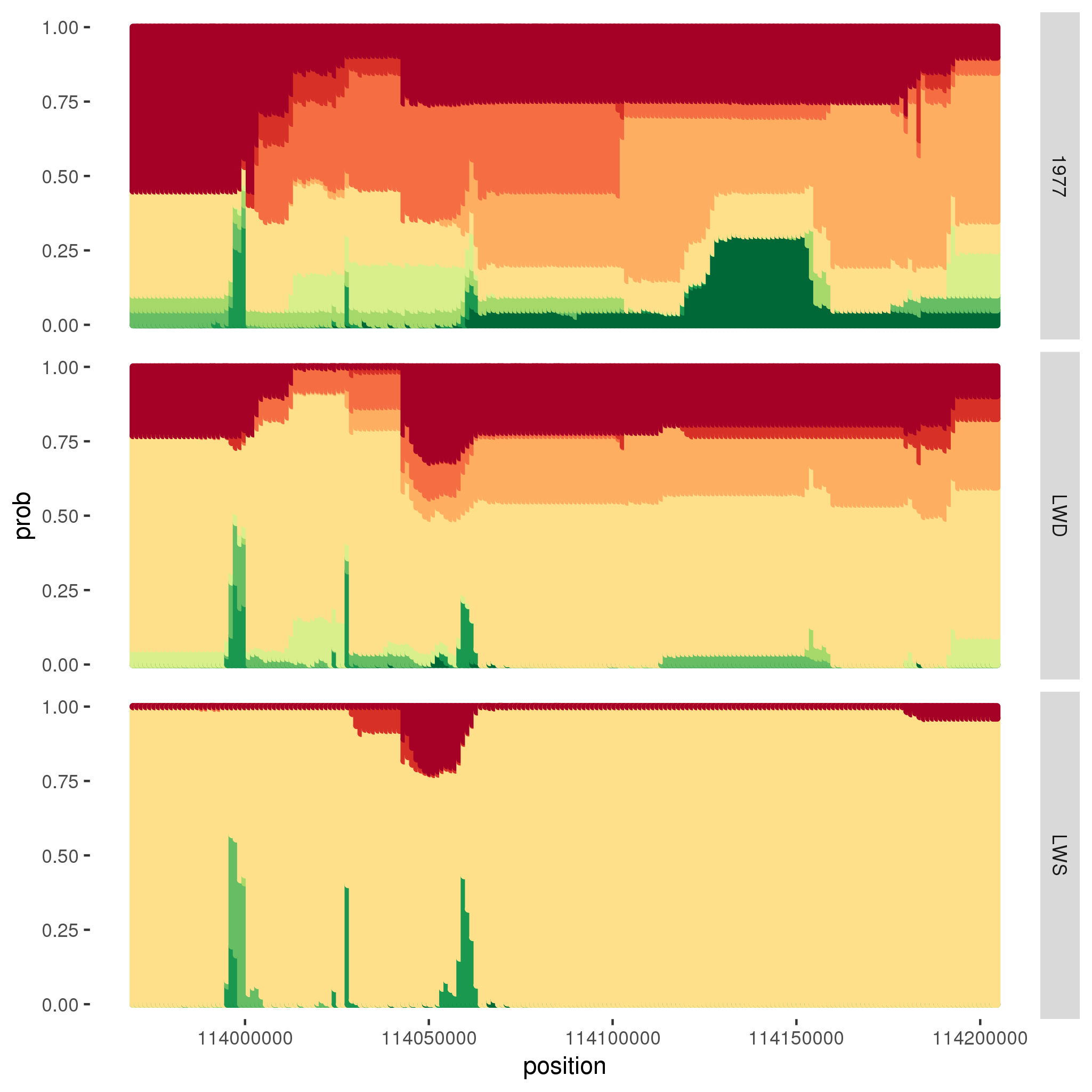

Supplement: Supplementary file 5 — Additional file 5. Haplotype frequency plots for all candidate regions of Table 1. Each file of this archive corresponds to a different candidate region, whose genomic position can be read in the file name. It shows the haplotype diversity in this region, estimated using hapFLK with 10 haplotype clusters (for one single EM run). For a given genomic position, each color corresponds to a different haplotype cluster and the height of the color band in a given population (represented by one of the panels) gives the frequency of the cluster in this population. All variants (AV set) were considered for these analyses. [file 12711_2023_789_MOESM5_ESM.xz › hapflk_plots/all10_auto_chro13_113970091-114204018.kfrq.fit_2.bz2-plot.png]

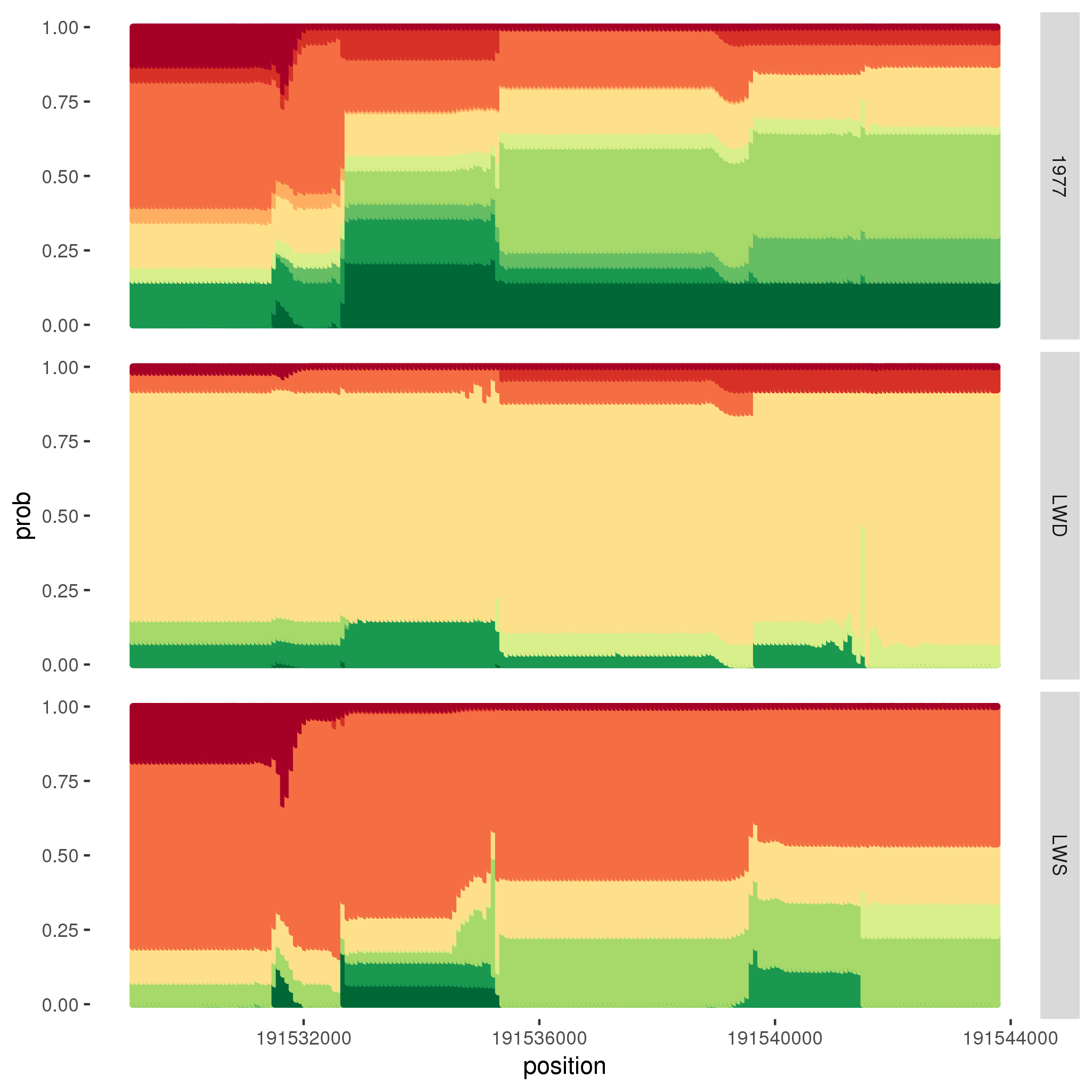

Supplement: Supplementary file 5 — Additional file 5. Haplotype frequency plots for all candidate regions of Table 1. Each file of this archive corresponds to a different candidate region, whose genomic position can be read in the file name. It shows the haplotype diversity in this region, estimated using hapFLK with 10 haplotype clusters (for one single EM run). For a given genomic position, each color corresponds to a different haplotype cluster and the height of the color band in a given population (represented by one of the panels) gives the frequency of the cluster in this population. All variants (AV set) were considered for these analyses. [file 12711_2023_789_MOESM5_ESM.xz › hapflk_plots/all10_auto_chro13_191529139-191543733.kfrq.fit_0.bz2-plot.png]

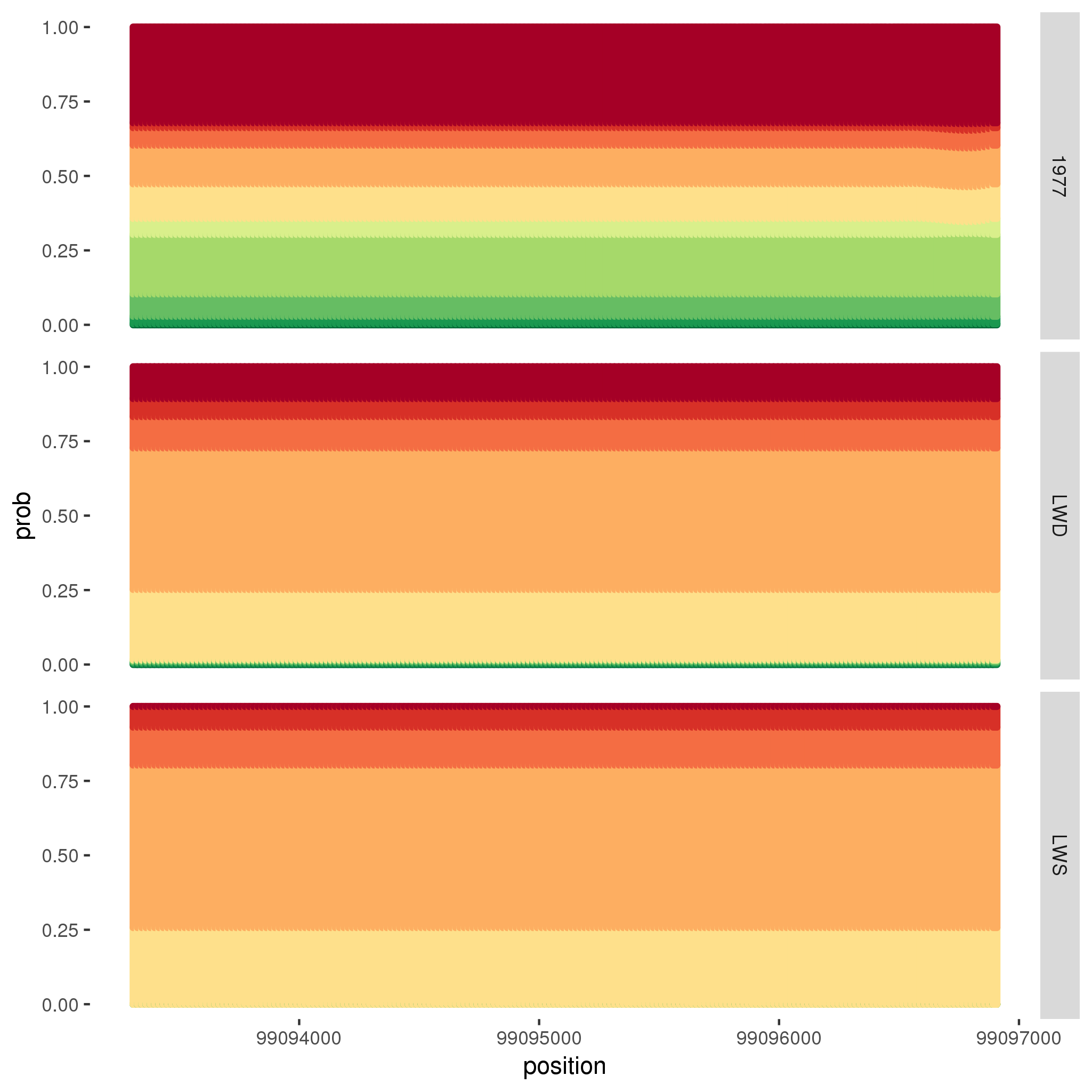

Supplement: Supplementary file 5 — Additional file 5. Haplotype frequency plots for all candidate regions of Table 1. Each file of this archive corresponds to a different candidate region, whose genomic position can be read in the file name. It shows the haplotype diversity in this region, estimated using hapFLK with 10 haplotype clusters (for one single EM run). For a given genomic position, each color corresponds to a different haplotype cluster and the height of the color band in a given population (represented by one of the panels) gives the frequency of the cluster in this population. All variants (AV set) were considered for these analyses. [file 12711_2023_789_MOESM5_ESM.xz › hapflk_plots/all10_auto_chro13_99093316-99096900.kfrq.fit_0.bz2-plot.png]

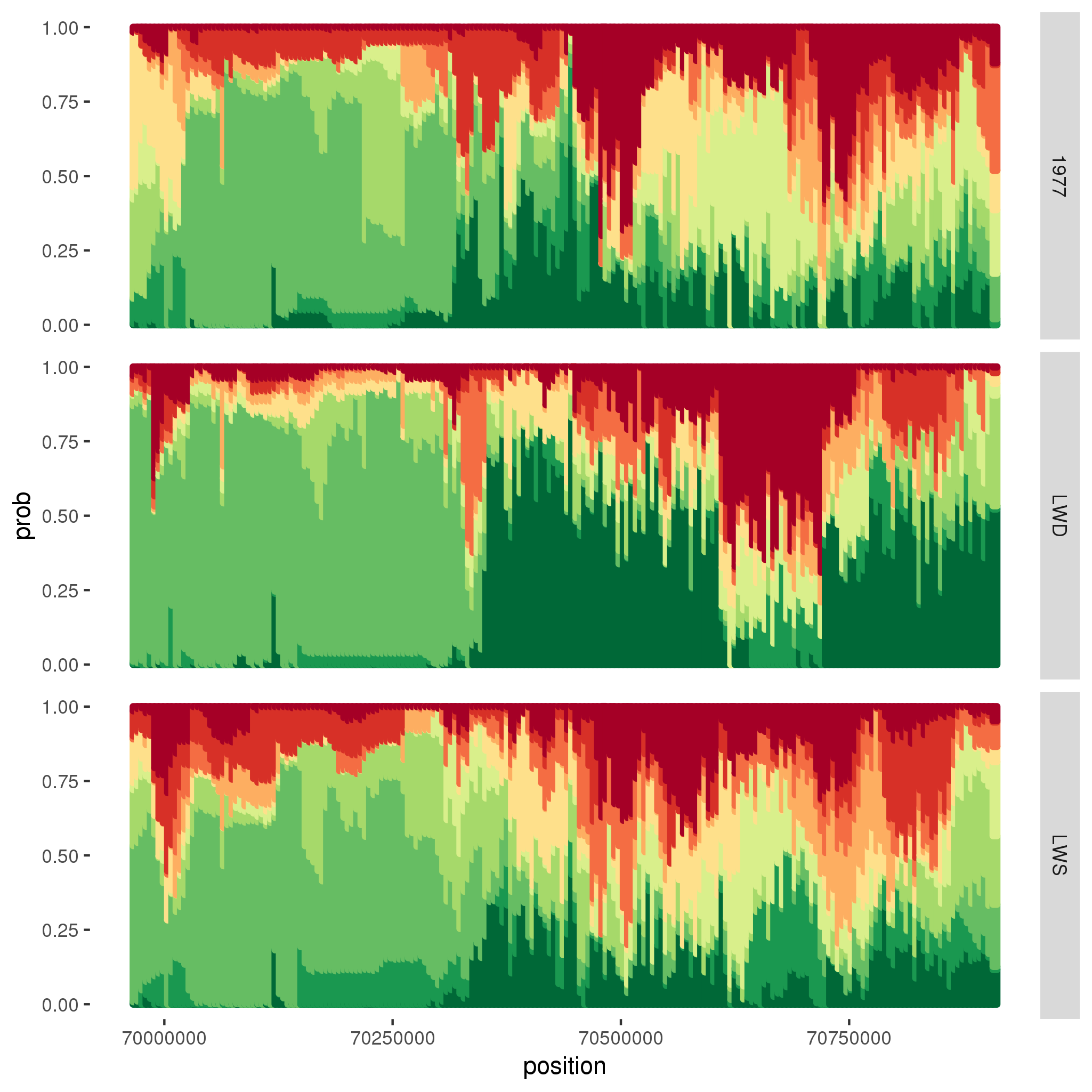

Supplement: Supplementary file 5 — Additional file 5. Haplotype frequency plots for all candidate regions of Table 1. Each file of this archive corresponds to a different candidate region, whose genomic position can be read in the file name. It shows the haplotype diversity in this region, estimated using hapFLK with 10 haplotype clusters (for one single EM run). For a given genomic position, each color corresponds to a different haplotype cluster and the height of the color band in a given population (represented by one of the panels) gives the frequency of the cluster in this population. All variants (AV set) were considered for these analyses. [file 12711_2023_789_MOESM5_ESM.xz › hapflk_plots/all10_auto_chro16_69967970-70909691.kfrq.fit_2.bz2-plot.png]

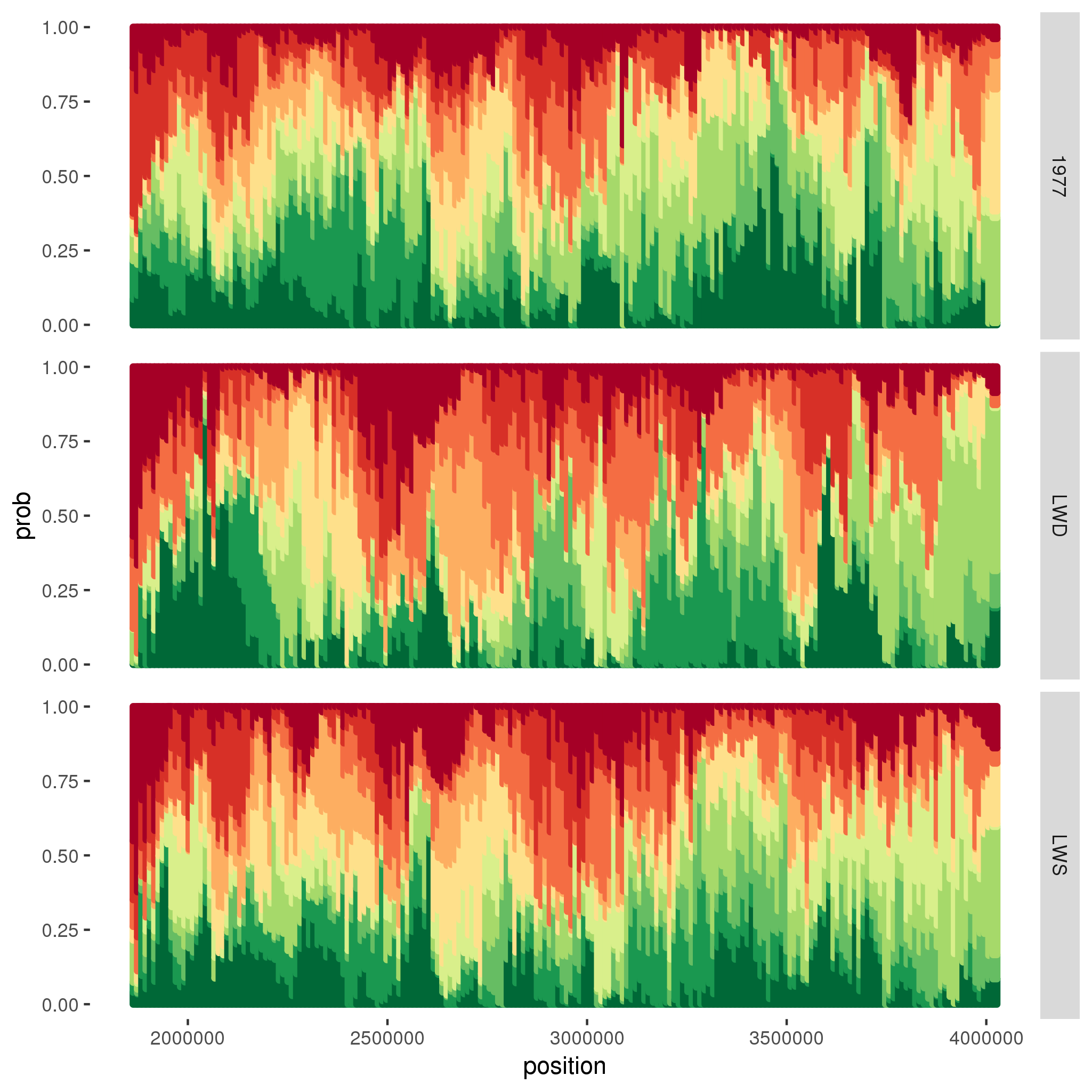

Supplement: Supplementary file 5 — Additional file 5. Haplotype frequency plots for all candidate regions of Table 1. Each file of this archive corresponds to a different candidate region, whose genomic position can be read in the file name. It shows the haplotype diversity in this region, estimated using hapFLK with 10 haplotype clusters (for one single EM run). For a given genomic position, each color corresponds to a different haplotype cluster and the height of the color band in a given population (represented by one of the panels) gives the frequency of the cluster in this population. All variants (AV set) were considered for these analyses. [file 12711_2023_789_MOESM5_ESM.xz › hapflk_plots/all10_auto_chro17_1867811-4021774.kfrq.fit_1.bz2-plot.png]

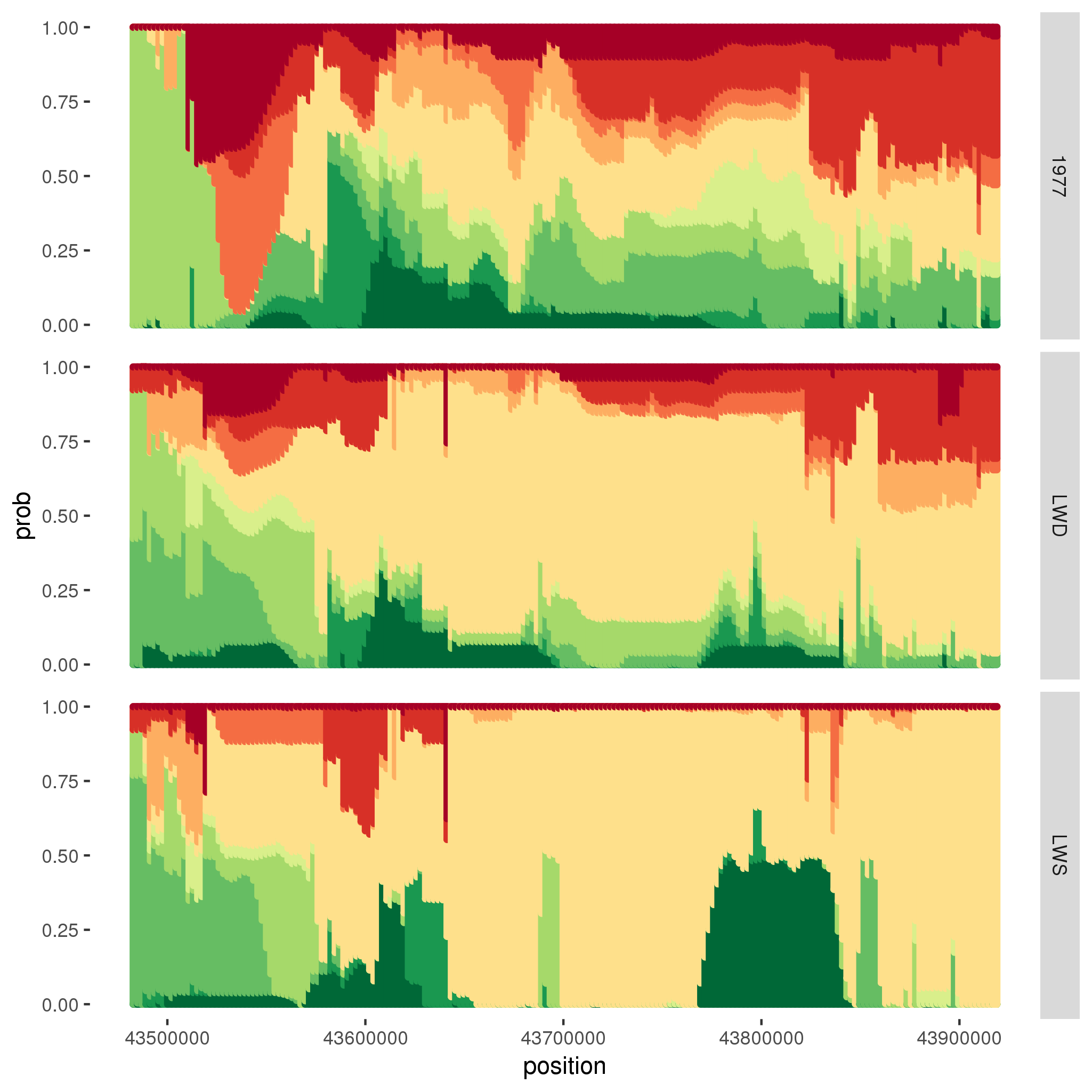

Supplement: Supplementary file 5 — Additional file 5. Haplotype frequency plots for all candidate regions of Table 1. Each file of this archive corresponds to a different candidate region, whose genomic position can be read in the file name. It shows the haplotype diversity in this region, estimated using hapFLK with 10 haplotype clusters (for one single EM run). For a given genomic position, each color corresponds to a different haplotype cluster and the height of the color band in a given population (represented by one of the panels) gives the frequency of the cluster in this population. All variants (AV set) were considered for these analyses. [file 12711_2023_789_MOESM5_ESM.xz › hapflk_plots/all10_auto_chro17_43483704-43917949.kfrq.fit_1.bz2-plot.png]

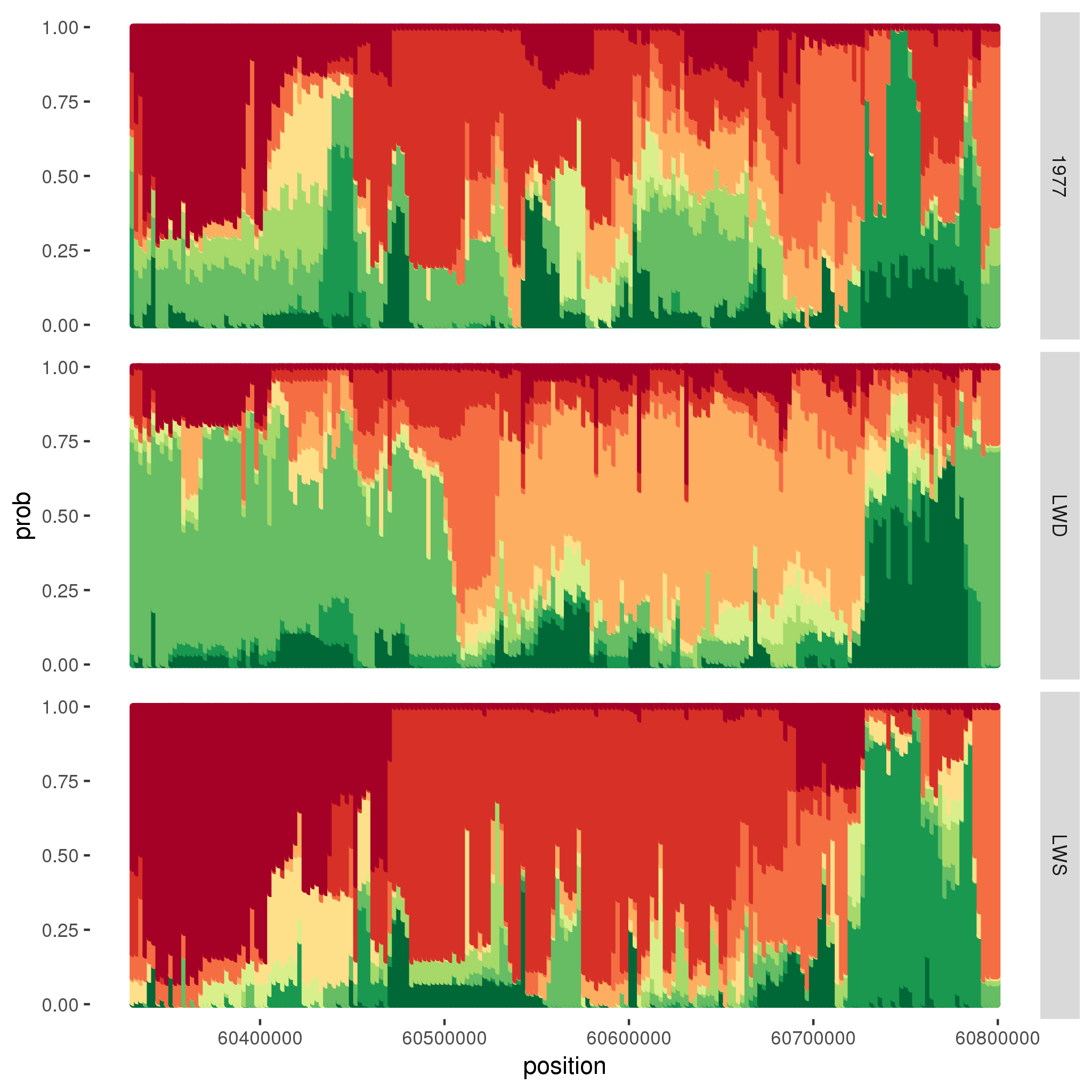

Supplement: Supplementary file 5 — Additional file 5. Haplotype frequency plots for all candidate regions of Table 1. Each file of this archive corresponds to a different candidate region, whose genomic position can be read in the file name. It shows the haplotype diversity in this region, estimated using hapFLK with 10 haplotype clusters (for one single EM run). For a given genomic position, each color corresponds to a different haplotype cluster and the height of the color band in a given population (represented by one of the panels) gives the frequency of the cluster in this population. All variants (AV set) were considered for these analyses. [file 12711_2023_789_MOESM5_ESM.xz › hapflk_plots/all10_auto_chro17_60332242-60798465.kfrq.fit_2.bz2-plot.png]

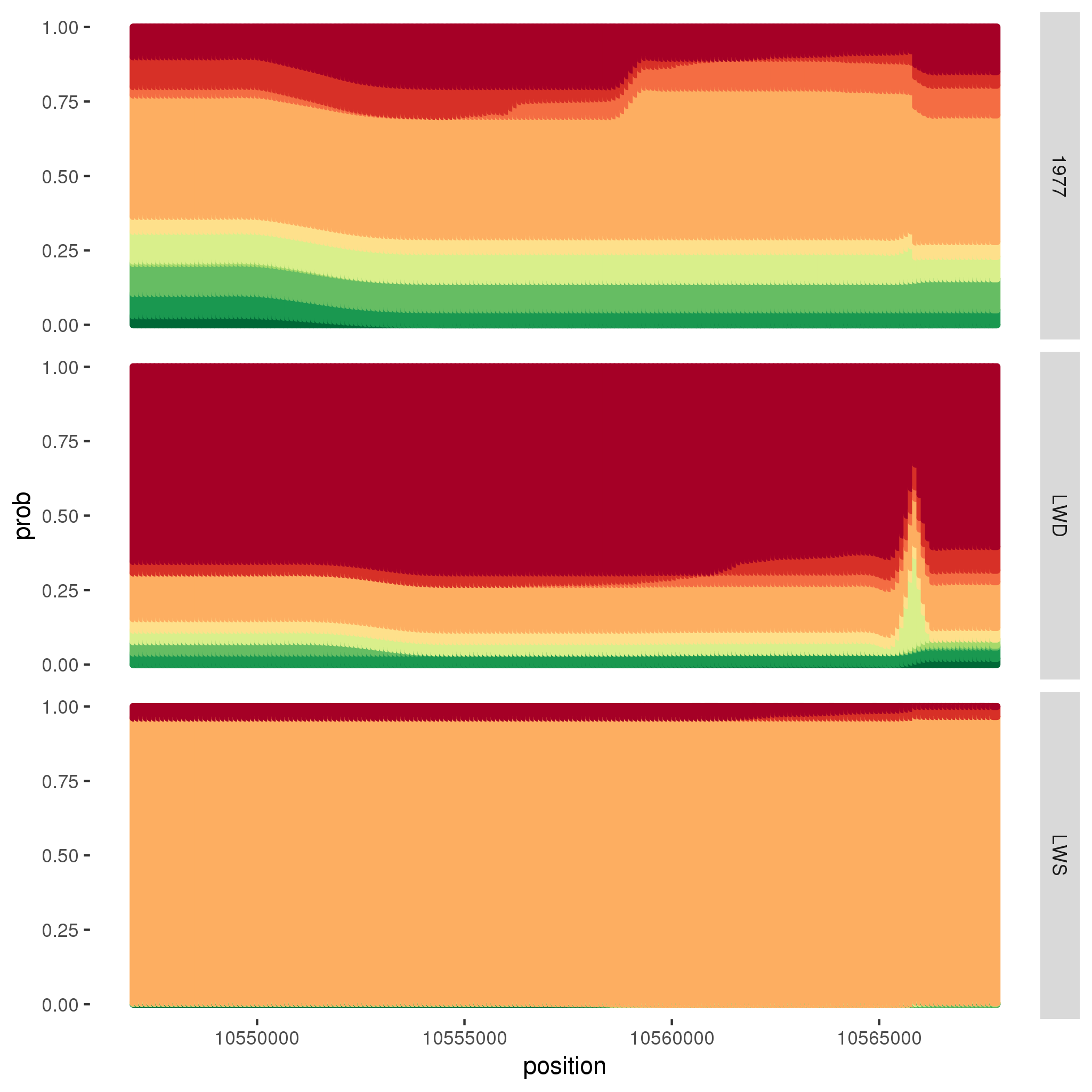

Supplement: Supplementary file 5 — Additional file 5. Haplotype frequency plots for all candidate regions of Table 1. Each file of this archive corresponds to a different candidate region, whose genomic position can be read in the file name. It shows the haplotype diversity in this region, estimated using hapFLK with 10 haplotype clusters (for one single EM run). For a given genomic position, each color corresponds to a different haplotype cluster and the height of the color band in a given population (represented by one of the panels) gives the frequency of the cluster in this population. All variants (AV set) were considered for these analyses. [file 12711_2023_789_MOESM5_ESM.xz › hapflk_plots/all10_auto_chro18_10547058-10567791.kfrq.fit_1.bz2-plot.png]

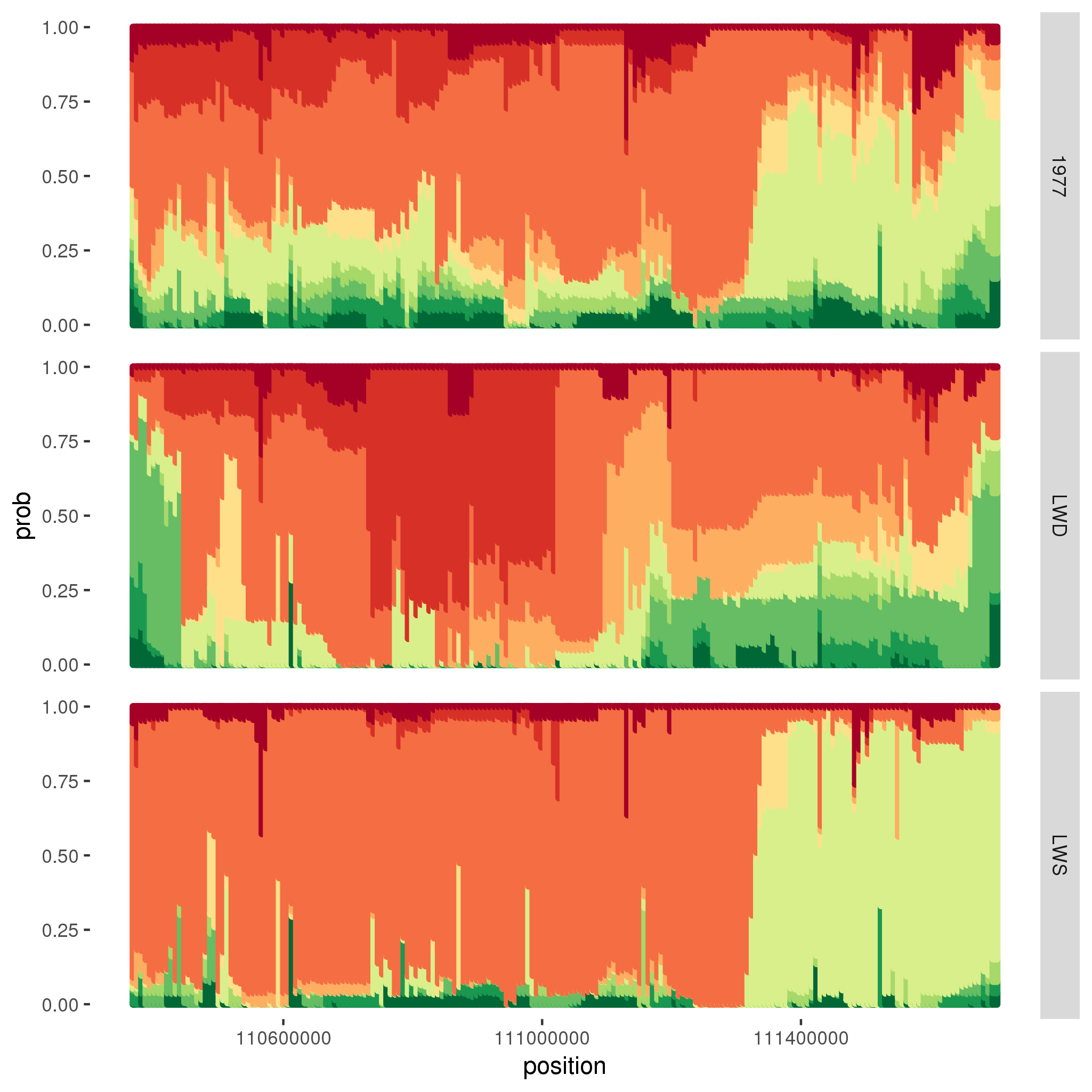

Supplement: Supplementary file 5 — Additional file 5. Haplotype frequency plots for all candidate regions of Table 1. Each file of this archive corresponds to a different candidate region, whose genomic position can be read in the file name. It shows the haplotype diversity in this region, estimated using hapFLK with 10 haplotype clusters (for one single EM run). For a given genomic position, each color corresponds to a different haplotype cluster and the height of the color band in a given population (represented by one of the panels) gives the frequency of the cluster in this population. All variants (AV set) were considered for these analyses. [file 12711_2023_789_MOESM5_ESM.xz › hapflk_plots/all10_auto_chro1_110370901-111699835.kfrq.fit_2.bz2-plot.png]

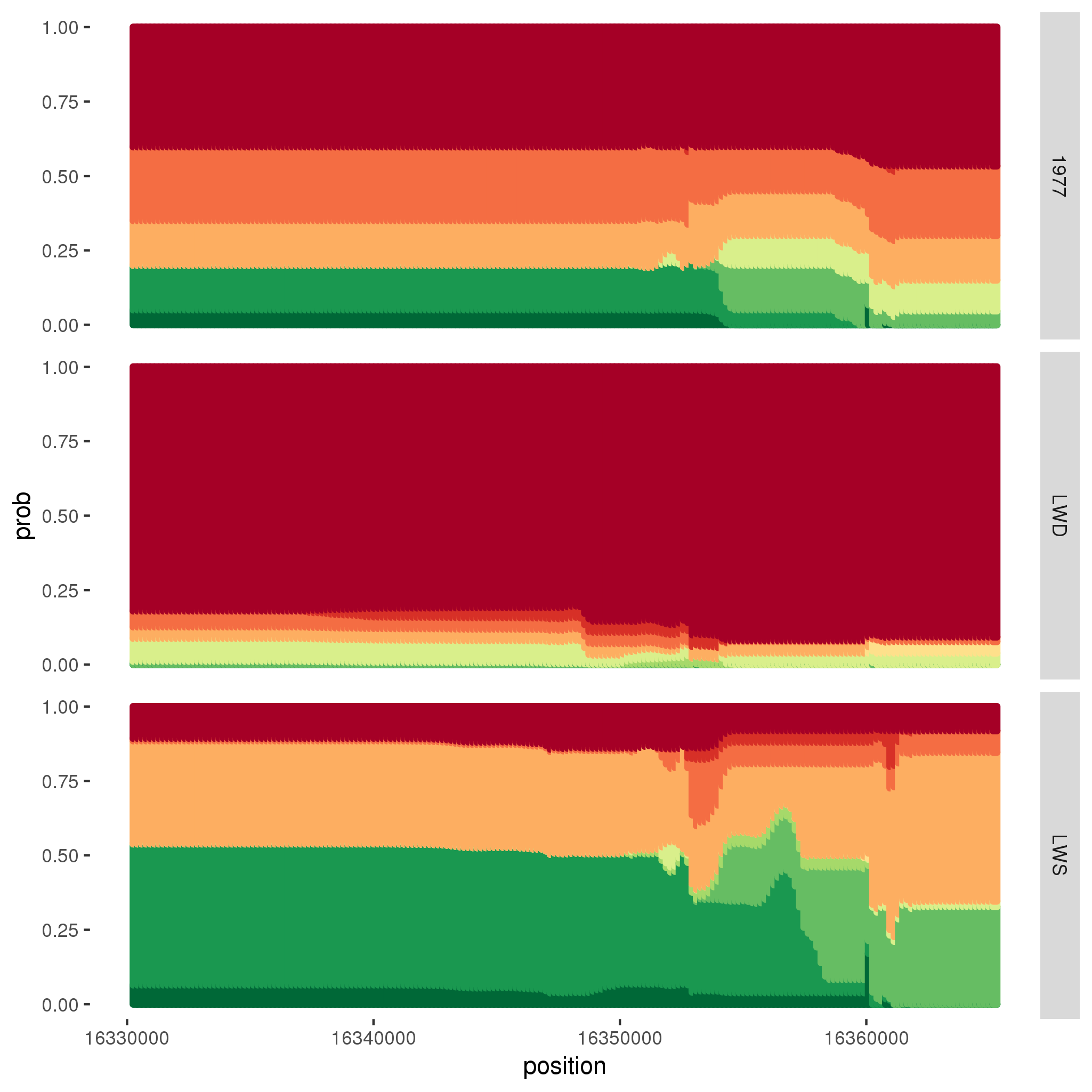

Supplement: Supplementary file 5 — Additional file 5. Haplotype frequency plots for all candidate regions of Table 1. Each file of this archive corresponds to a different candidate region, whose genomic position can be read in the file name. It shows the haplotype diversity in this region, estimated using hapFLK with 10 haplotype clusters (for one single EM run). For a given genomic position, each color corresponds to a different haplotype cluster and the height of the color band in a given population (represented by one of the panels) gives the frequency of the cluster in this population. All variants (AV set) were considered for these analyses. [file 12711_2023_789_MOESM5_ESM.xz › hapflk_plots/all10_auto_chro1_16330324-16365222.kfrq.fit_1.bz2-plot.png]

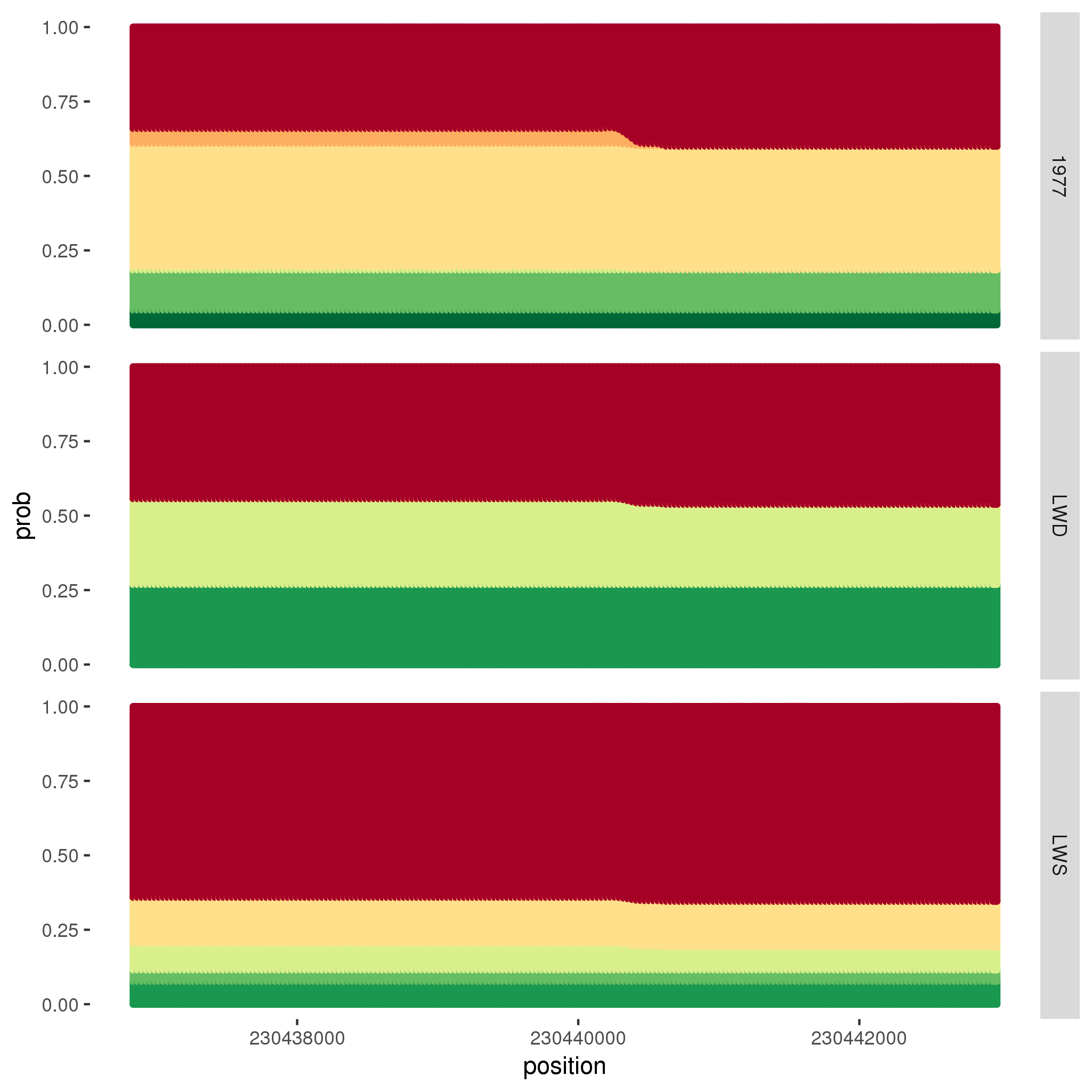

Supplement: Supplementary file 5 — Additional file 5. Haplotype frequency plots for all candidate regions of Table 1. Each file of this archive corresponds to a different candidate region, whose genomic position can be read in the file name. It shows the haplotype diversity in this region, estimated using hapFLK with 10 haplotype clusters (for one single EM run). For a given genomic position, each color corresponds to a different haplotype cluster and the height of the color band in a given population (represented by one of the panels) gives the frequency of the cluster in this population. All variants (AV set) were considered for these analyses. [file 12711_2023_789_MOESM5_ESM.xz › hapflk_plots/all10_auto_chro1_230436847-230442965.kfrq.fit_0.bz2-plot.png]

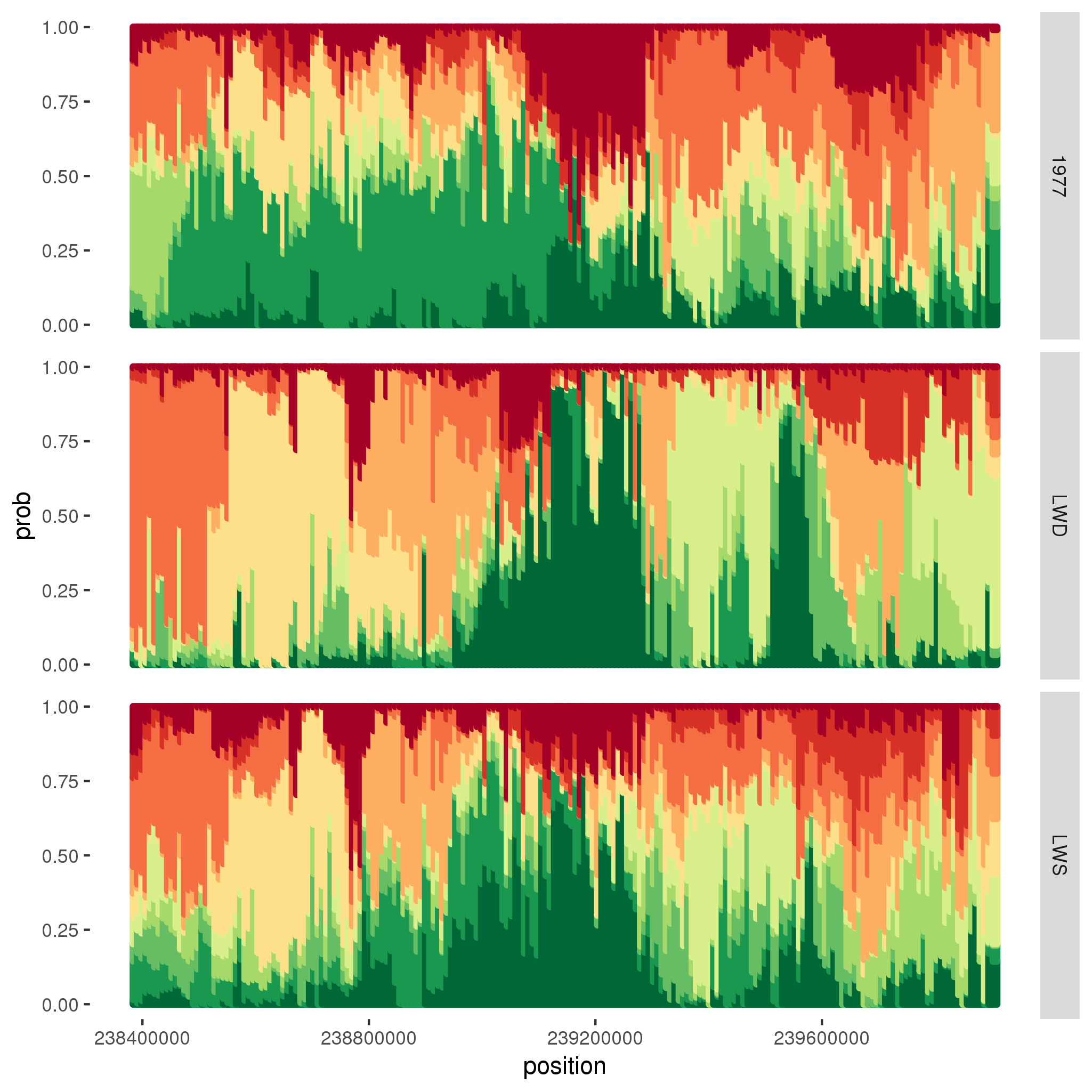

Supplement: Supplementary file 5 — Additional file 5. Haplotype frequency plots for all candidate regions of Table 1. Each file of this archive corresponds to a different candidate region, whose genomic position can be read in the file name. It shows the haplotype diversity in this region, estimated using hapFLK with 10 haplotype clusters (for one single EM run). For a given genomic position, each color corresponds to a different haplotype cluster and the height of the color band in a given population (represented by one of the panels) gives the frequency of the cluster in this population. All variants (AV set) were considered for these analyses. [file 12711_2023_789_MOESM5_ESM.xz › hapflk_plots/all10_auto_chro1_238387016-239905667.kfrq.fit_0.bz2-plot.png]

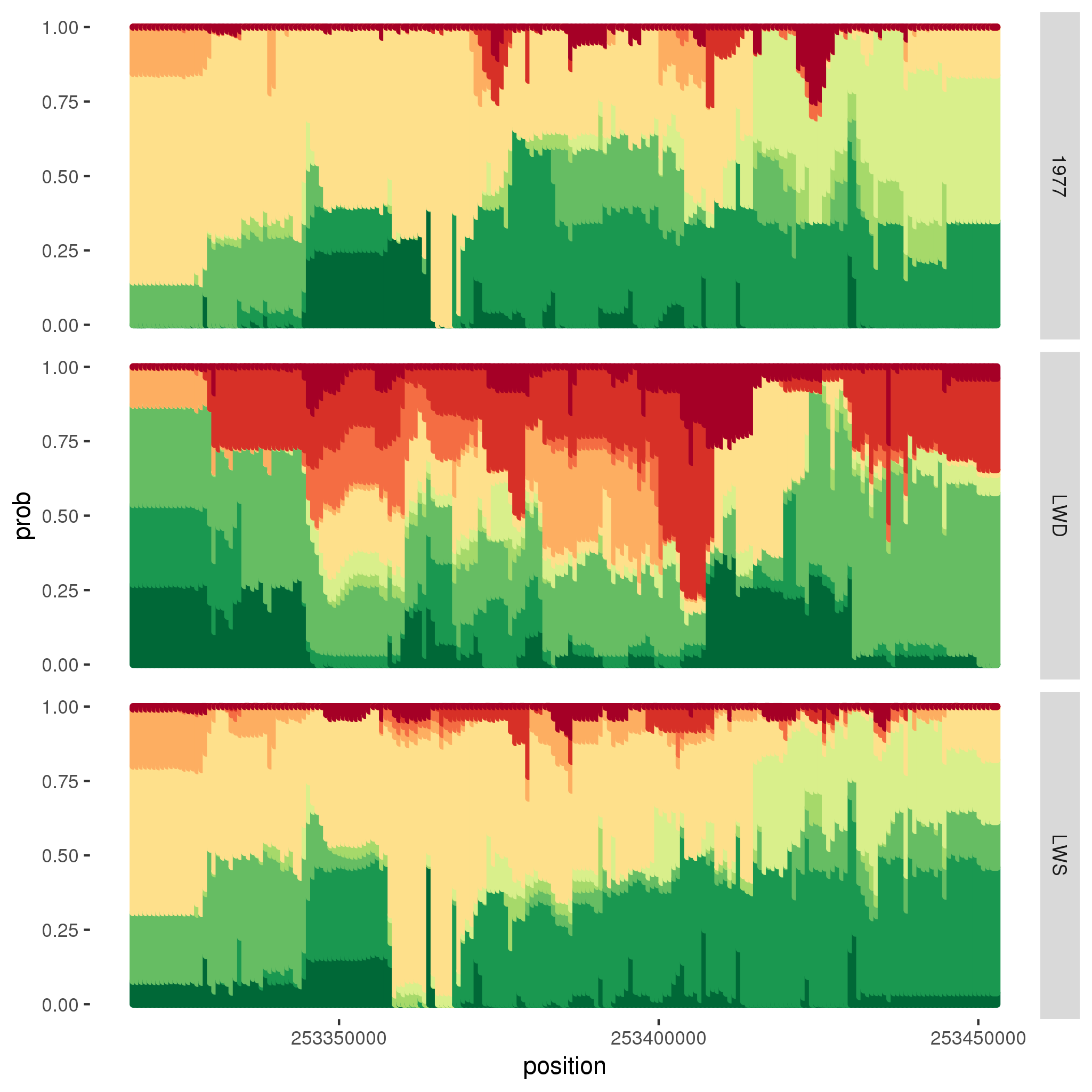

Supplement: Supplementary file 5 — Additional file 5. Haplotype frequency plots for all candidate regions of Table 1. Each file of this archive corresponds to a different candidate region, whose genomic position can be read in the file name. It shows the haplotype diversity in this region, estimated using hapFLK with 10 haplotype clusters (for one single EM run). For a given genomic position, each color corresponds to a different haplotype cluster and the height of the color band in a given population (represented by one of the panels) gives the frequency of the cluster in this population. All variants (AV set) were considered for these analyses. [file 12711_2023_789_MOESM5_ESM.xz › hapflk_plots/all10_auto_chro1_253318090-253452613.kfrq.fit_0.bz2-plot.png]

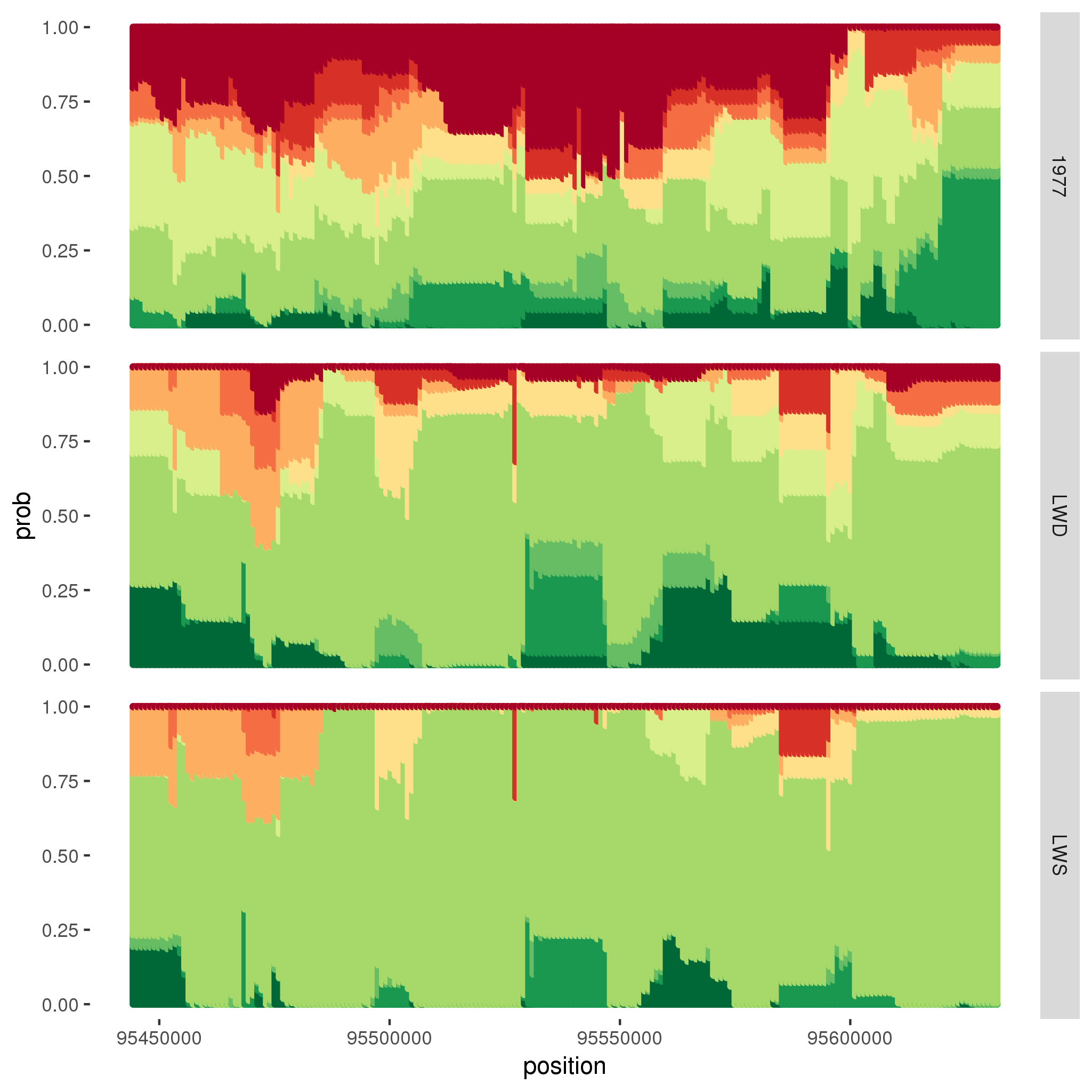

Supplement: Supplementary file 5 — Additional file 5. Haplotype frequency plots for all candidate regions of Table 1. Each file of this archive corresponds to a different candidate region, whose genomic position can be read in the file name. It shows the haplotype diversity in this region, estimated using hapFLK with 10 haplotype clusters (for one single EM run). For a given genomic position, each color corresponds to a different haplotype cluster and the height of the color band in a given population (represented by one of the panels) gives the frequency of the cluster in this population. All variants (AV set) were considered for these analyses. [file 12711_2023_789_MOESM5_ESM.xz › hapflk_plots/all10_auto_chro1_95444749-95631414.kfrq.fit_1.bz2-plot.png]

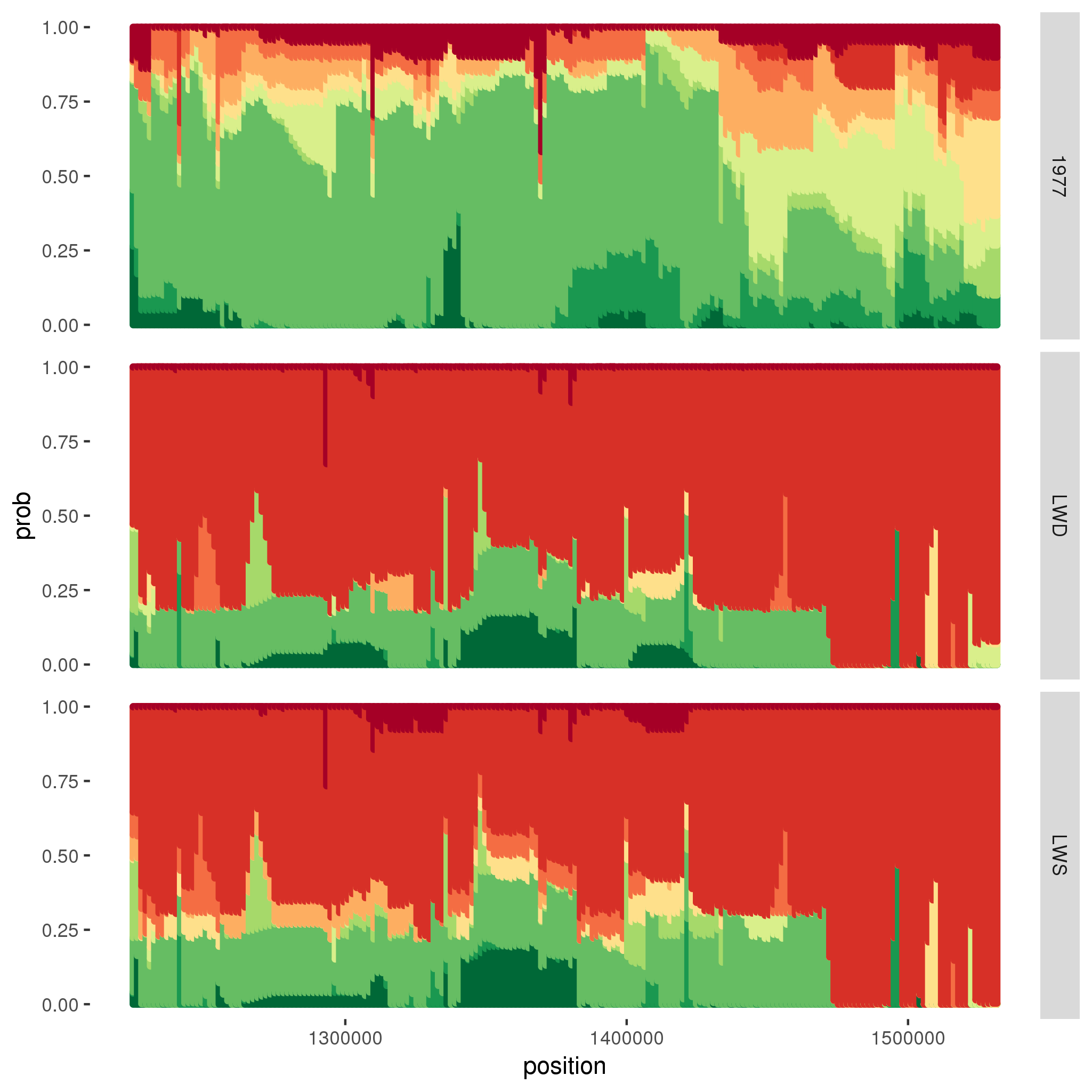

Supplement: Supplementary file 5 — Additional file 5. Haplotype frequency plots for all candidate regions of Table 1. Each file of this archive corresponds to a different candidate region, whose genomic position can be read in the file name. It shows the haplotype diversity in this region, estimated using hapFLK with 10 haplotype clusters (for one single EM run). For a given genomic position, each color corresponds to a different haplotype cluster and the height of the color band in a given population (represented by one of the panels) gives the frequency of the cluster in this population. All variants (AV set) were considered for these analyses. [file 12711_2023_789_MOESM5_ESM.xz › hapflk_plots/all10_auto_chro2_1225288-1530903.kfrq.fit_2.bz2-plot.png]

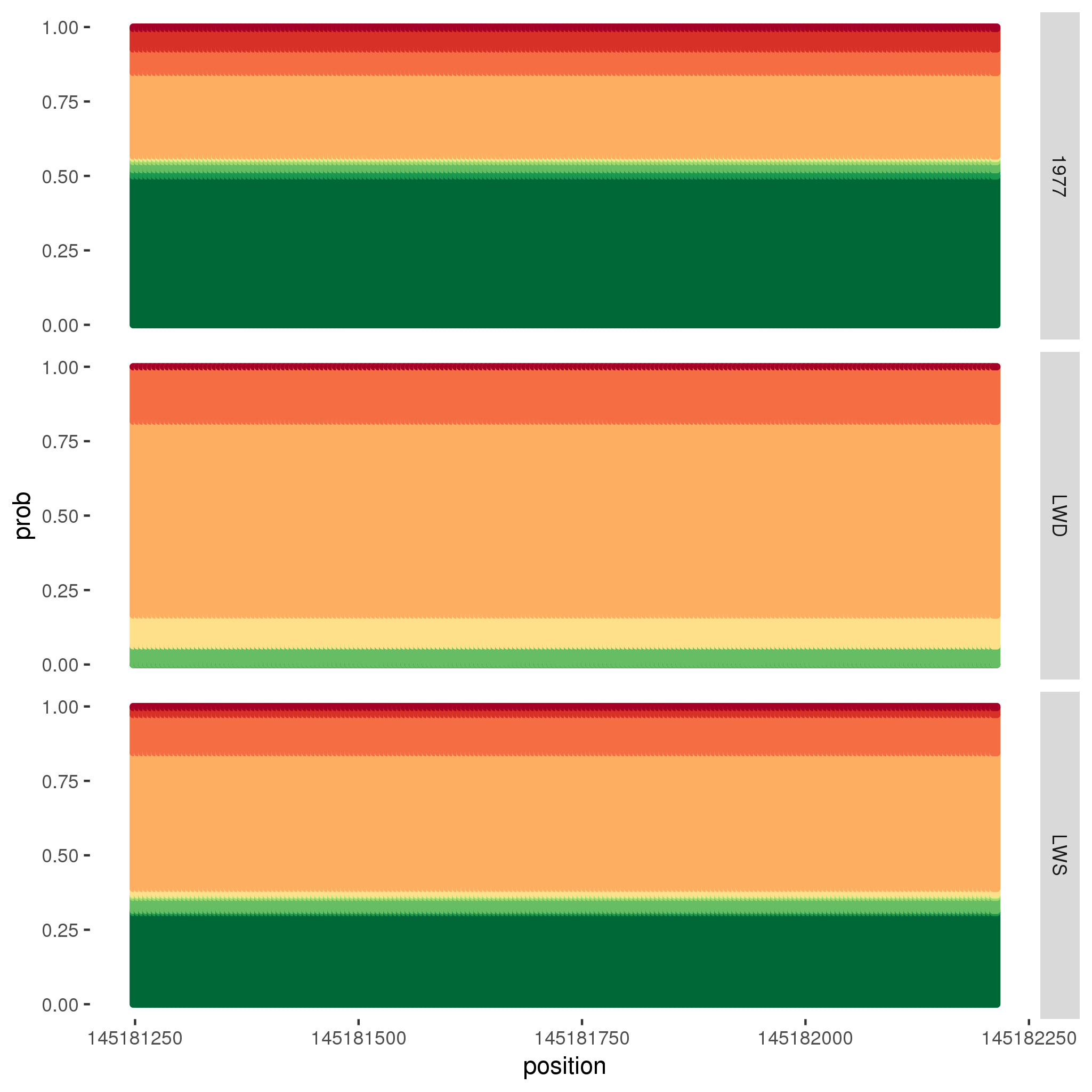

Supplement: Supplementary file 5 — Additional file 5. Haplotype frequency plots for all candidate regions of Table 1. Each file of this archive corresponds to a different candidate region, whose genomic position can be read in the file name. It shows the haplotype diversity in this region, estimated using hapFLK with 10 haplotype clusters (for one single EM run). For a given genomic position, each color corresponds to a different haplotype cluster and the height of the color band in a given population (represented by one of the panels) gives the frequency of the cluster in this population. All variants (AV set) were considered for these analyses. [file 12711_2023_789_MOESM5_ESM.xz › hapflk_plots/all10_auto_chro2_145181250-145182212.kfrq.fit_1.bz2-plot.png]

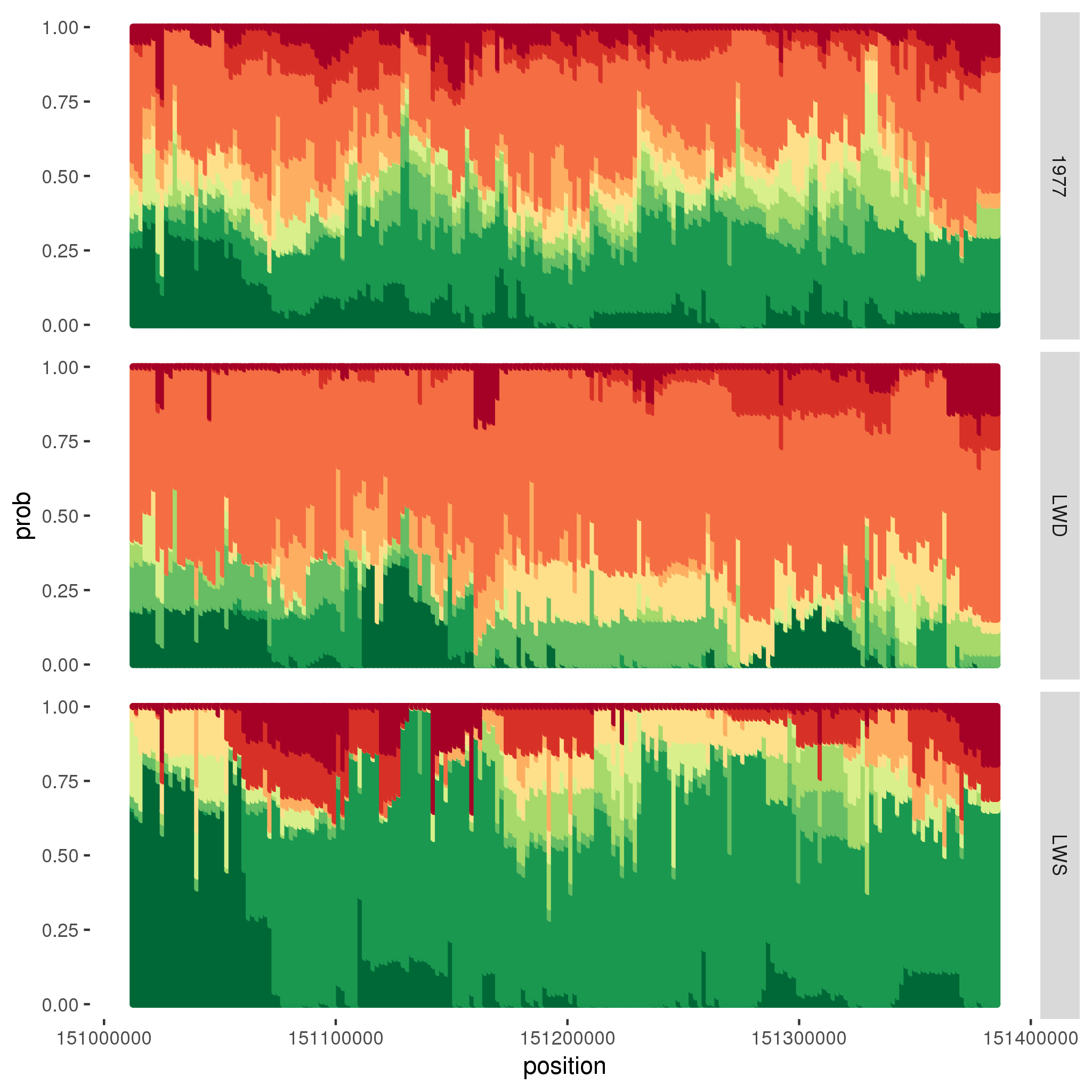

Supplement: Supplementary file 5 — Additional file 5. Haplotype frequency plots for all candidate regions of Table 1. Each file of this archive corresponds to a different candidate region, whose genomic position can be read in the file name. It shows the haplotype diversity in this region, estimated using hapFLK with 10 haplotype clusters (for one single EM run). For a given genomic position, each color corresponds to a different haplotype cluster and the height of the color band in a given population (represented by one of the panels) gives the frequency of the cluster in this population. All variants (AV set) were considered for these analyses. [file 12711_2023_789_MOESM5_ESM.xz › hapflk_plots/all10_auto_chro2_151013393-151384536.kfrq.fit_0.bz2-plot.png]

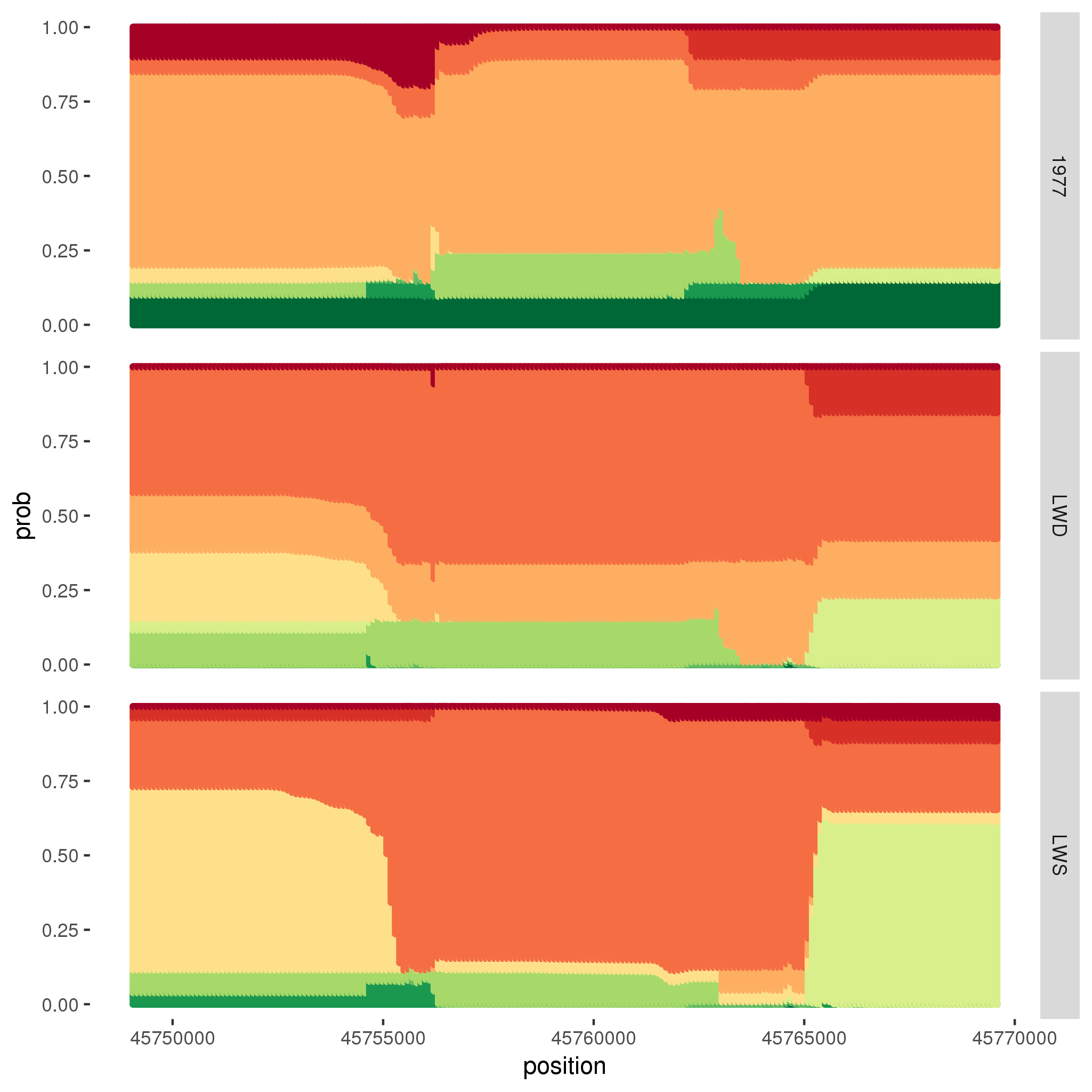

Supplement: Supplementary file 5 — Additional file 5. Haplotype frequency plots for all candidate regions of Table 1. Each file of this archive corresponds to a different candidate region, whose genomic position can be read in the file name. It shows the haplotype diversity in this region, estimated using hapFLK with 10 haplotype clusters (for one single EM run). For a given genomic position, each color corresponds to a different haplotype cluster and the height of the color band in a given population (represented by one of the panels) gives the frequency of the cluster in this population. All variants (AV set) were considered for these analyses. [file 12711_2023_789_MOESM5_ESM.xz › hapflk_plots/all10_auto_chro2_45749109-45769530.kfrq.fit_1.bz2-plot.png]

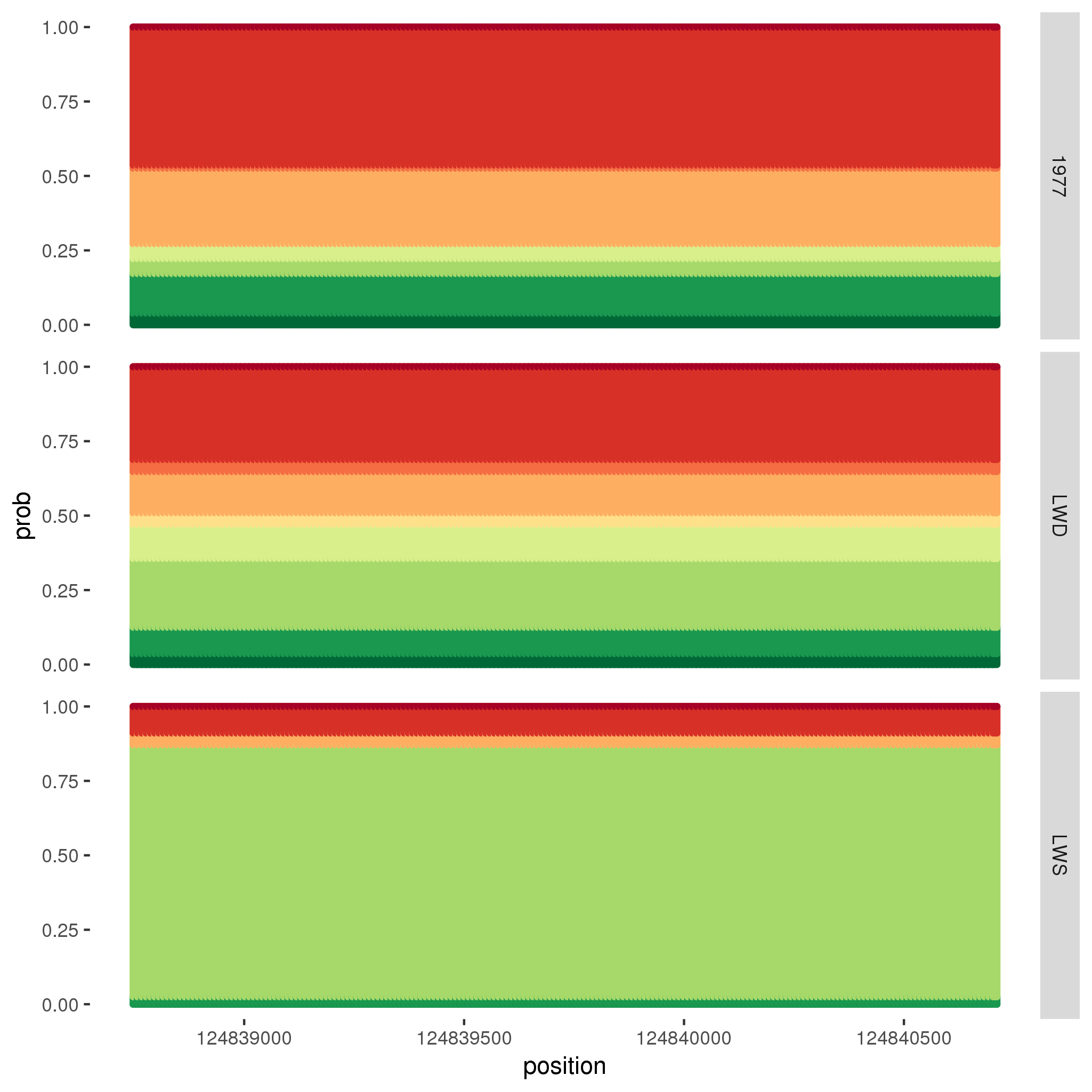

Supplement: Supplementary file 5 — Additional file 5. Haplotype frequency plots for all candidate regions of Table 1. Each file of this archive corresponds to a different candidate region, whose genomic position can be read in the file name. It shows the haplotype diversity in this region, estimated using hapFLK with 10 haplotype clusters (for one single EM run). For a given genomic position, each color corresponds to a different haplotype cluster and the height of the color band in a given population (represented by one of the panels) gives the frequency of the cluster in this population. All variants (AV set) were considered for these analyses. [file 12711_2023_789_MOESM5_ESM.xz › hapflk_plots/all10_auto_chro3_124838752-124840707.kfrq.fit_1.bz2-plot.png]

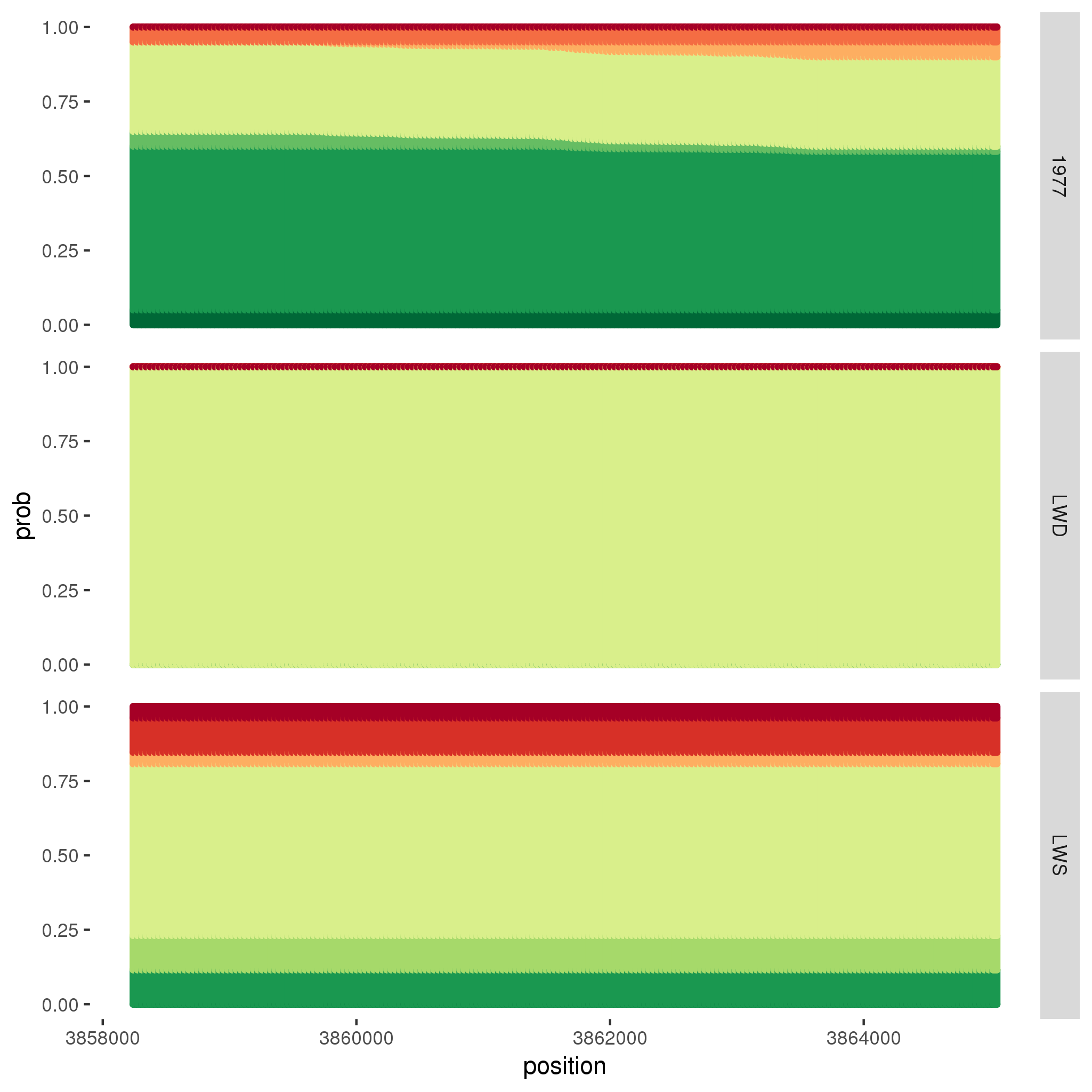

Supplement: Supplementary file 5 — Additional file 5. Haplotype frequency plots for all candidate regions of Table 1. Each file of this archive corresponds to a different candidate region, whose genomic position can be read in the file name. It shows the haplotype diversity in this region, estimated using hapFLK with 10 haplotype clusters (for one single EM run). For a given genomic position, each color corresponds to a different haplotype cluster and the height of the color band in a given population (represented by one of the panels) gives the frequency of the cluster in this population. All variants (AV set) were considered for these analyses. [file 12711_2023_789_MOESM5_ESM.xz › hapflk_plots/all10_auto_chro3_3858255-3865035.kfrq.fit_0.bz2-plot.png]

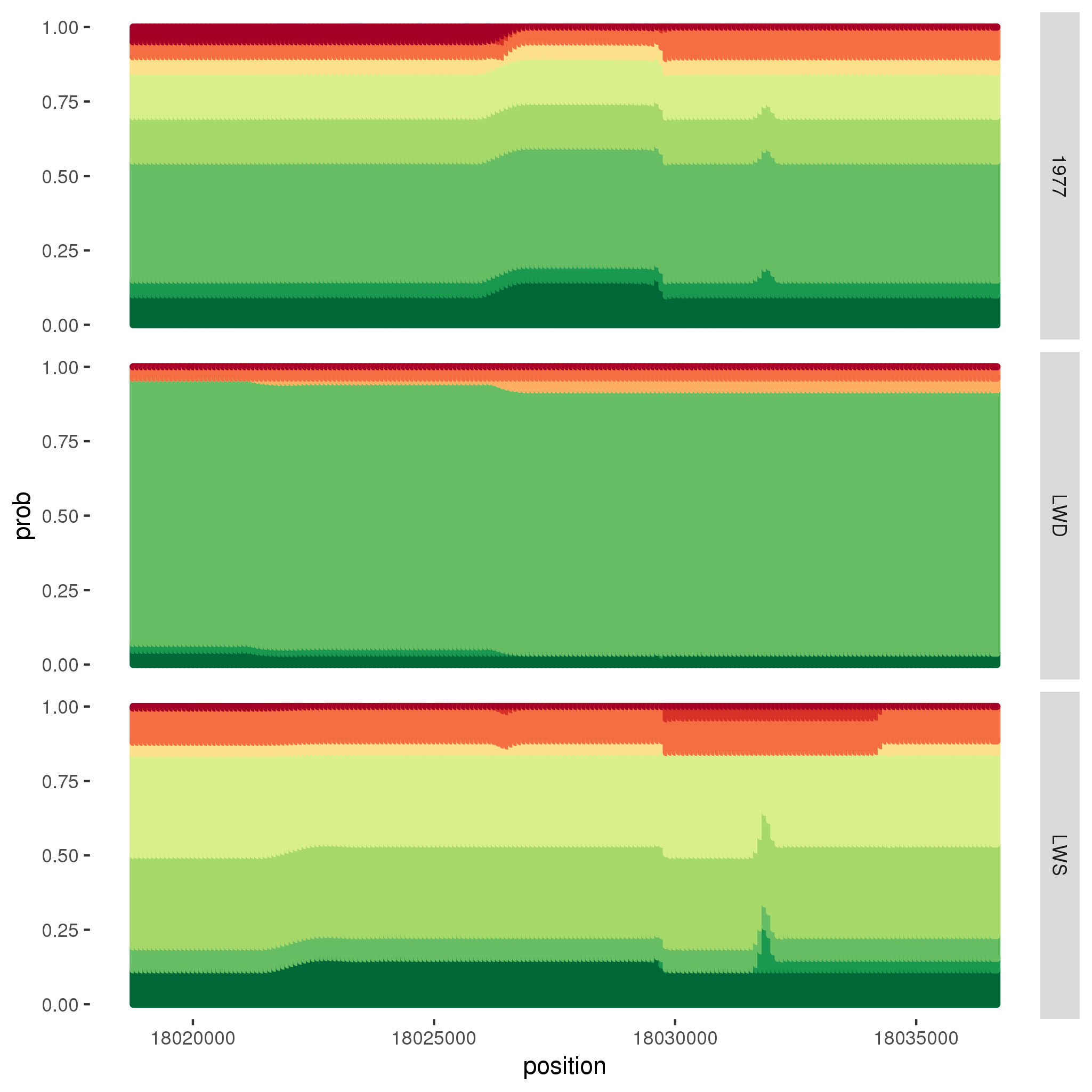

Supplement: Supplementary file 5 — Additional file 5. Haplotype frequency plots for all candidate regions of Table 1. Each file of this archive corresponds to a different candidate region, whose genomic position can be read in the file name. It shows the haplotype diversity in this region, estimated using hapFLK with 10 haplotype clusters (for one single EM run). For a given genomic position, each color corresponds to a different haplotype cluster and the height of the color band in a given population (represented by one of the panels) gives the frequency of the cluster in this population. All variants (AV set) were considered for these analyses. [file 12711_2023_789_MOESM5_ESM.xz › hapflk_plots/all10_auto_chro4_18018801-18036634.kfrq.fit_1.bz2-plot.png]

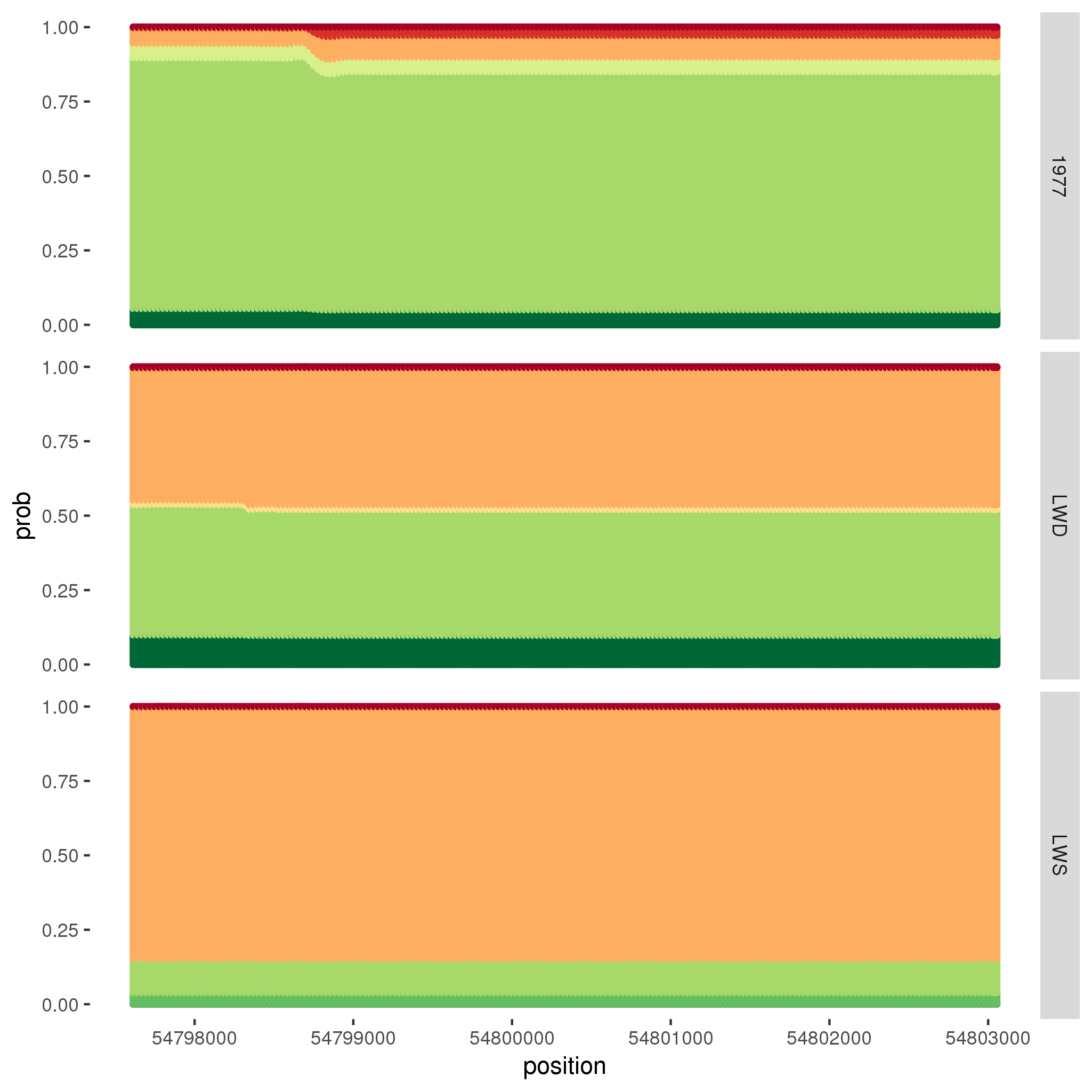

Supplement: Supplementary file 5 — Additional file 5. Haplotype frequency plots for all candidate regions of Table 1. Each file of this archive corresponds to a different candidate region, whose genomic position can be read in the file name. It shows the haplotype diversity in this region, estimated using hapFLK with 10 haplotype clusters (for one single EM run). For a given genomic position, each color corresponds to a different haplotype cluster and the height of the color band in a given population (represented by one of the panels) gives the frequency of the cluster in this population. All variants (AV set) were considered for these analyses. [file 12711_2023_789_MOESM5_ESM.xz › hapflk_plots/all10_auto_chro4_54797625-54803043.kfrq.fit_0.bz2-plot.png]

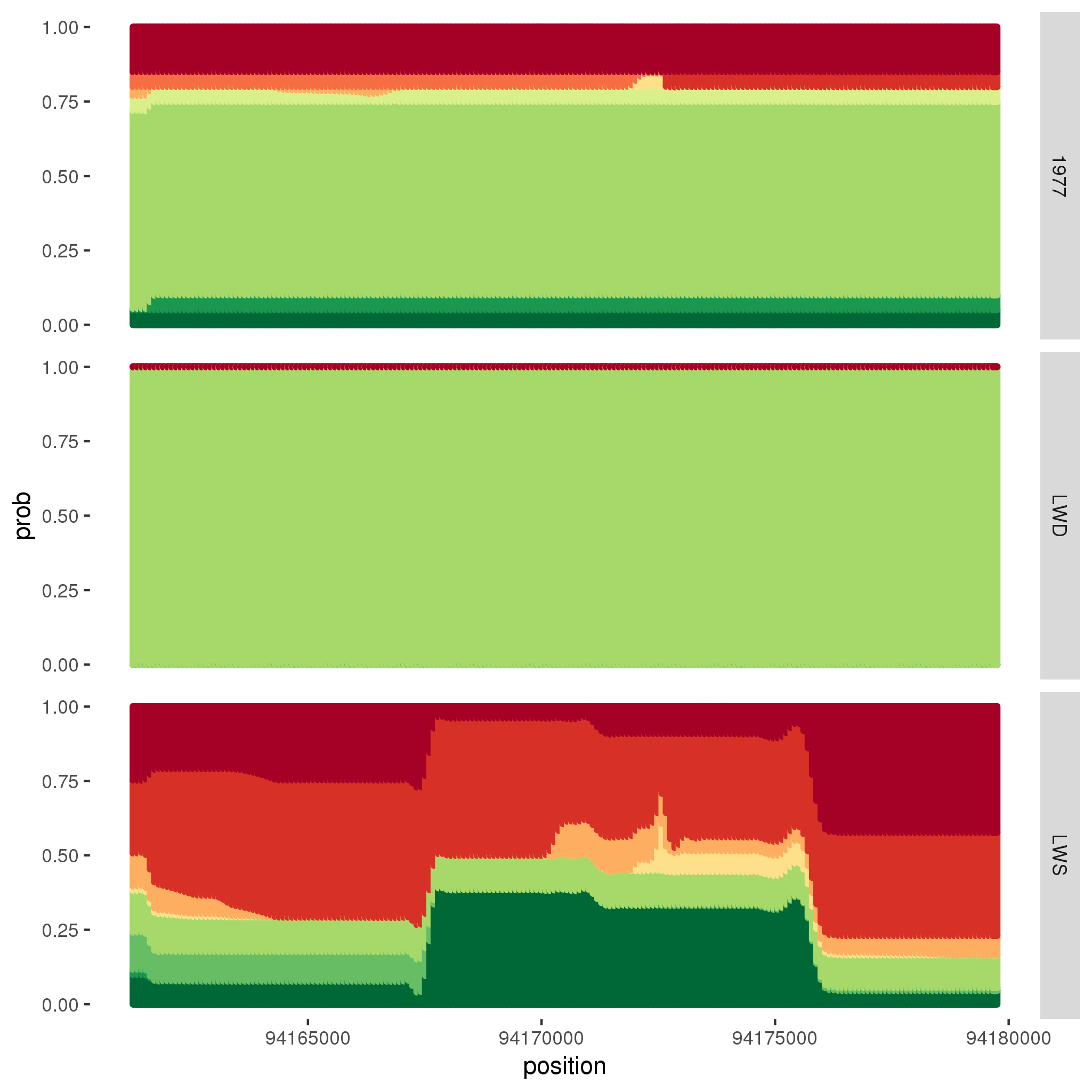

Supplement: Supplementary file 5 — Additional file 5. Haplotype frequency plots for all candidate regions of Table 1. Each file of this archive corresponds to a different candidate region, whose genomic position can be read in the file name. It shows the haplotype diversity in this region, estimated using hapFLK with 10 haplotype clusters (for one single EM run). For a given genomic position, each color corresponds to a different haplotype cluster and the height of the color band in a given population (represented by one of the panels) gives the frequency of the cluster in this population. All variants (AV set) were considered for these analyses. [file 12711_2023_789_MOESM5_ESM.xz › hapflk_plots/all10_auto_chro5_94161299-94179707.kfrq.fit_1.bz2-plot.png]

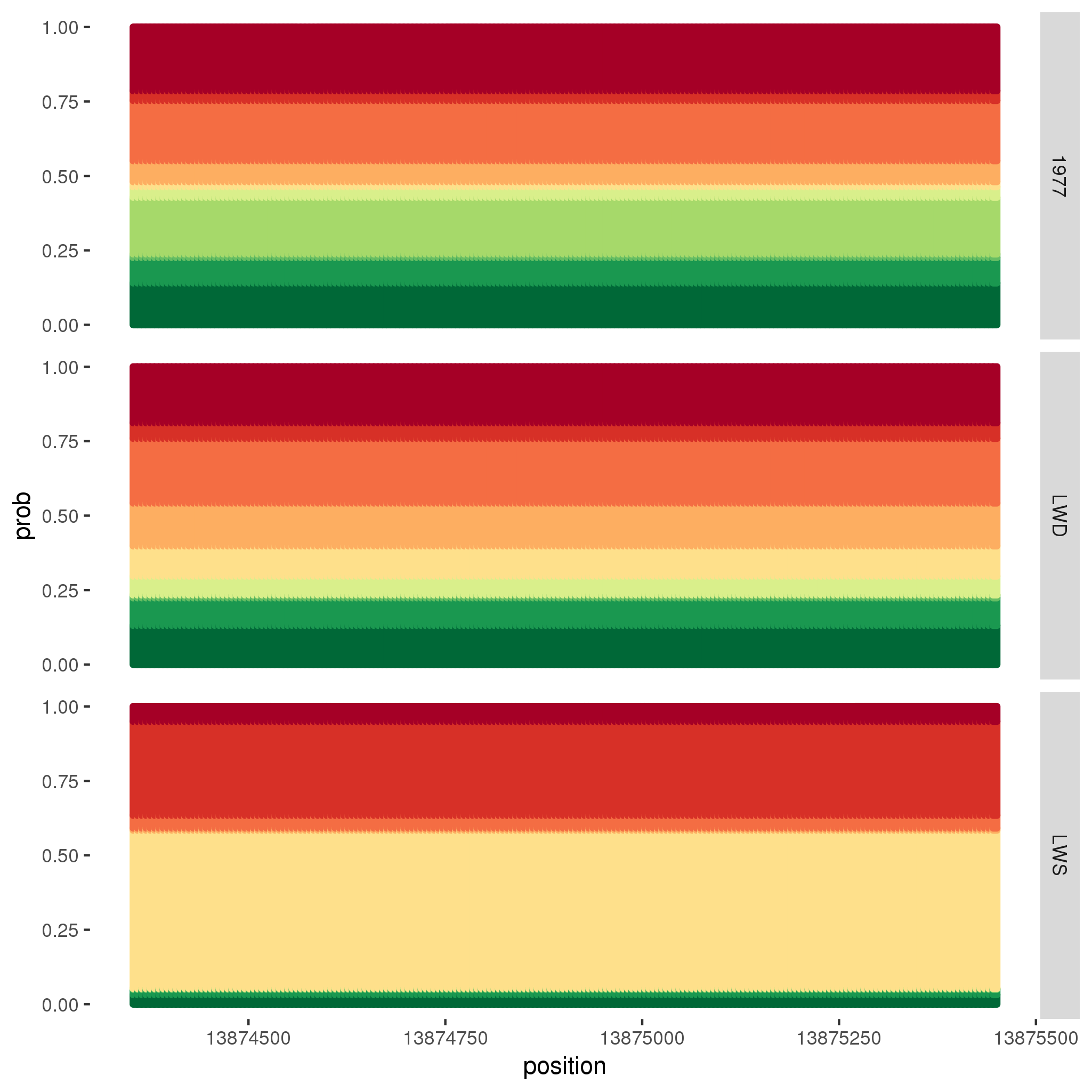

Supplement: Supplementary file 5 — Additional file 5. Haplotype frequency plots for all candidate regions of Table 1. Each file of this archive corresponds to a different candidate region, whose genomic position can be read in the file name. It shows the haplotype diversity in this region, estimated using hapFLK with 10 haplotype clusters (for one single EM run). For a given genomic position, each color corresponds to a different haplotype cluster and the height of the color band in a given population (represented by one of the panels) gives the frequency of the cluster in this population. All variants (AV set) were considered for these analyses. [file 12711_2023_789_MOESM5_ESM.xz › hapflk_plots/all10_auto_chro6_13874356-13875448.kfrq.fit_0.bz2-plot.png]

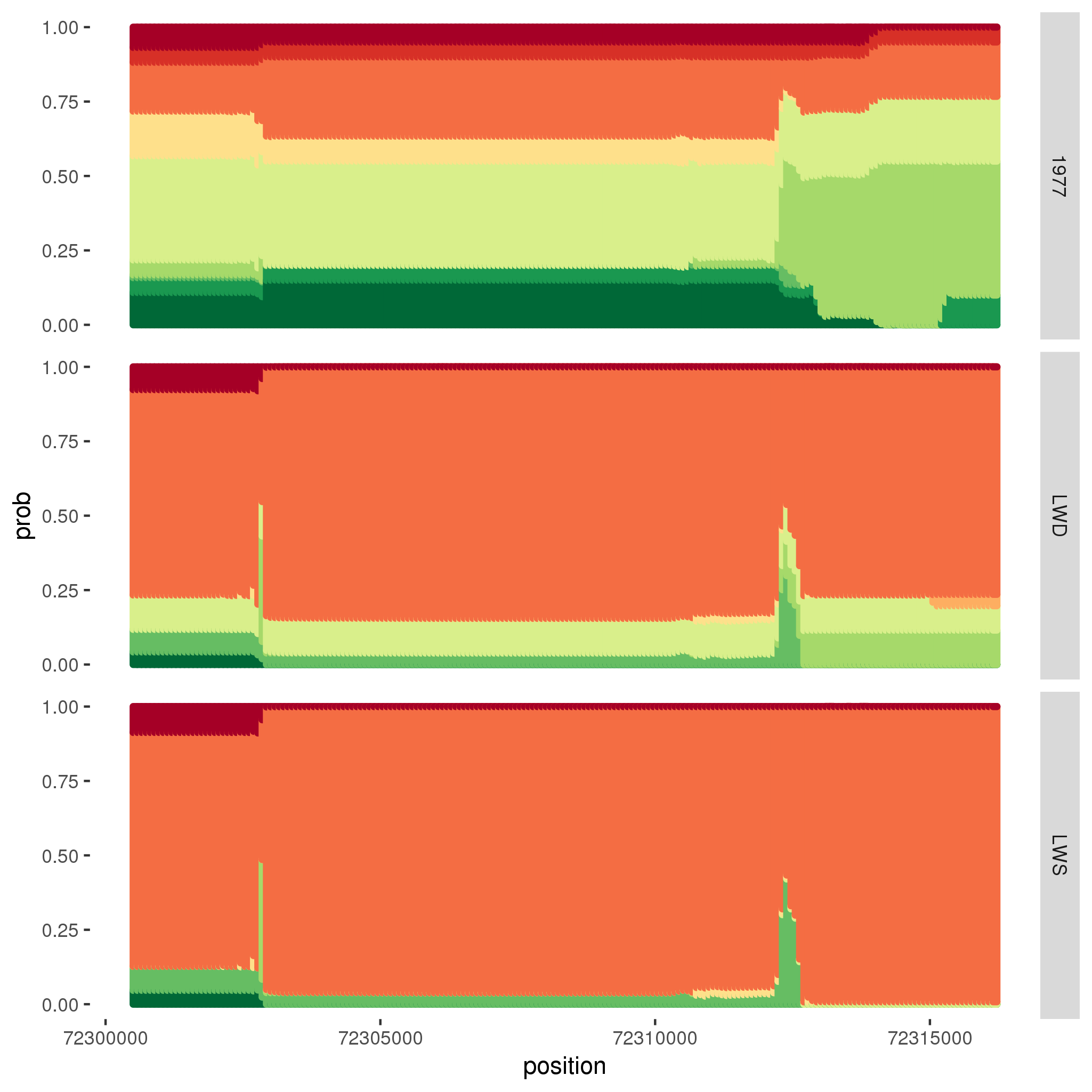

Supplement: Supplementary file 5 — Additional file 5. Haplotype frequency plots for all candidate regions of Table 1. Each file of this archive corresponds to a different candidate region, whose genomic position can be read in the file name. It shows the haplotype diversity in this region, estimated using hapFLK with 10 haplotype clusters (for one single EM run). For a given genomic position, each color corresponds to a different haplotype cluster and the height of the color band in a given population (represented by one of the panels) gives the frequency of the cluster in this population. All variants (AV set) were considered for these analyses. [file 12711_2023_789_MOESM5_ESM.xz › hapflk_plots/all10_auto_chro6_72300537-72316184.kfrq.fit_2.bz2-plot.png]

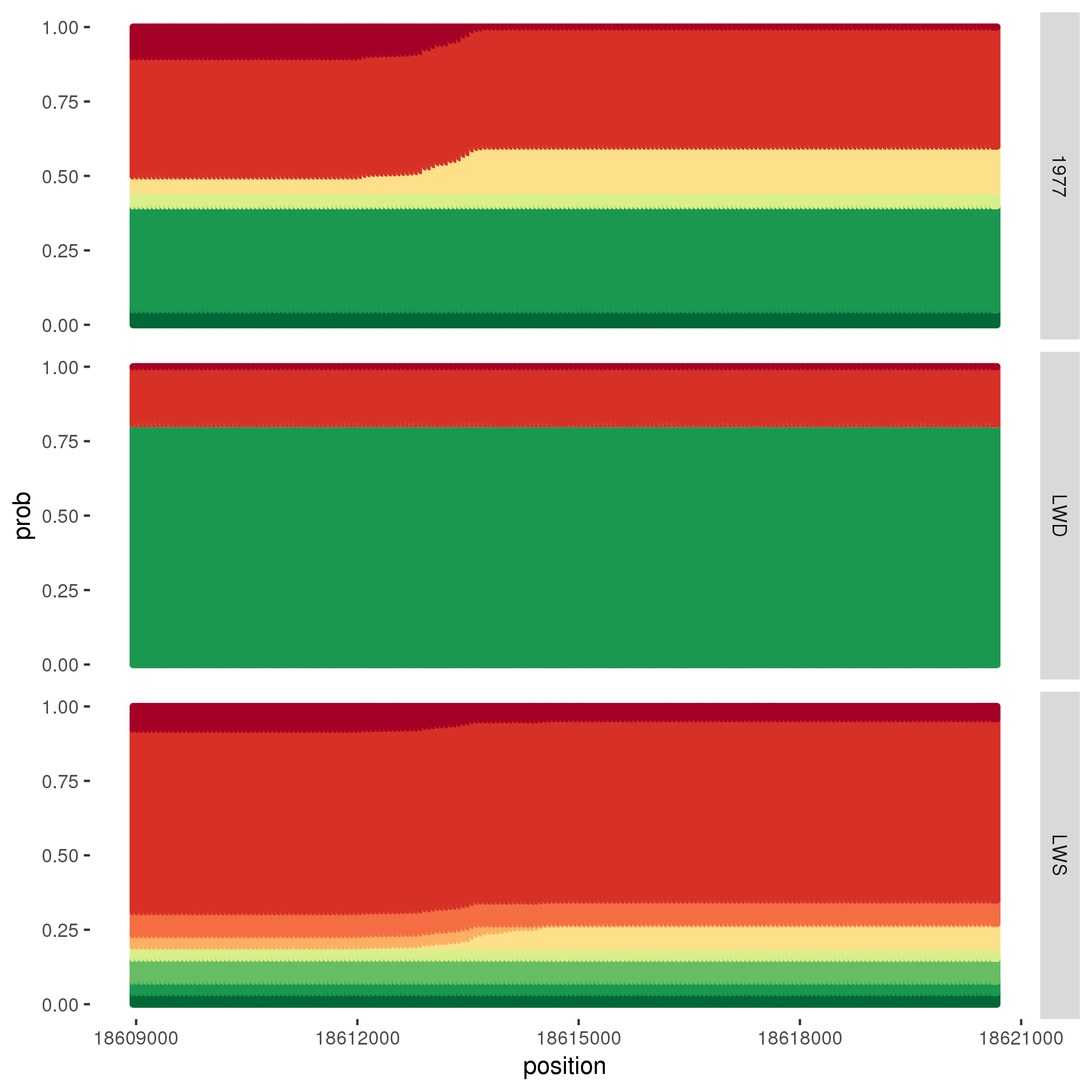

Supplement: Supplementary file 5 — Additional file 5. Haplotype frequency plots for all candidate regions of Table 1. Each file of this archive corresponds to a different candidate region, whose genomic position can be read in the file name. It shows the haplotype diversity in this region, estimated using hapFLK with 10 haplotype clusters (for one single EM run). For a given genomic position, each color corresponds to a different haplotype cluster and the height of the color band in a given population (represented by one of the panels) gives the frequency of the cluster in this population. All variants (AV set) were considered for these analyses. [file 12711_2023_789_MOESM5_ESM.xz › hapflk_plots/all10_auto_chro7_18608986-18620645.kfrq.fit_0.bz2-plot.png]

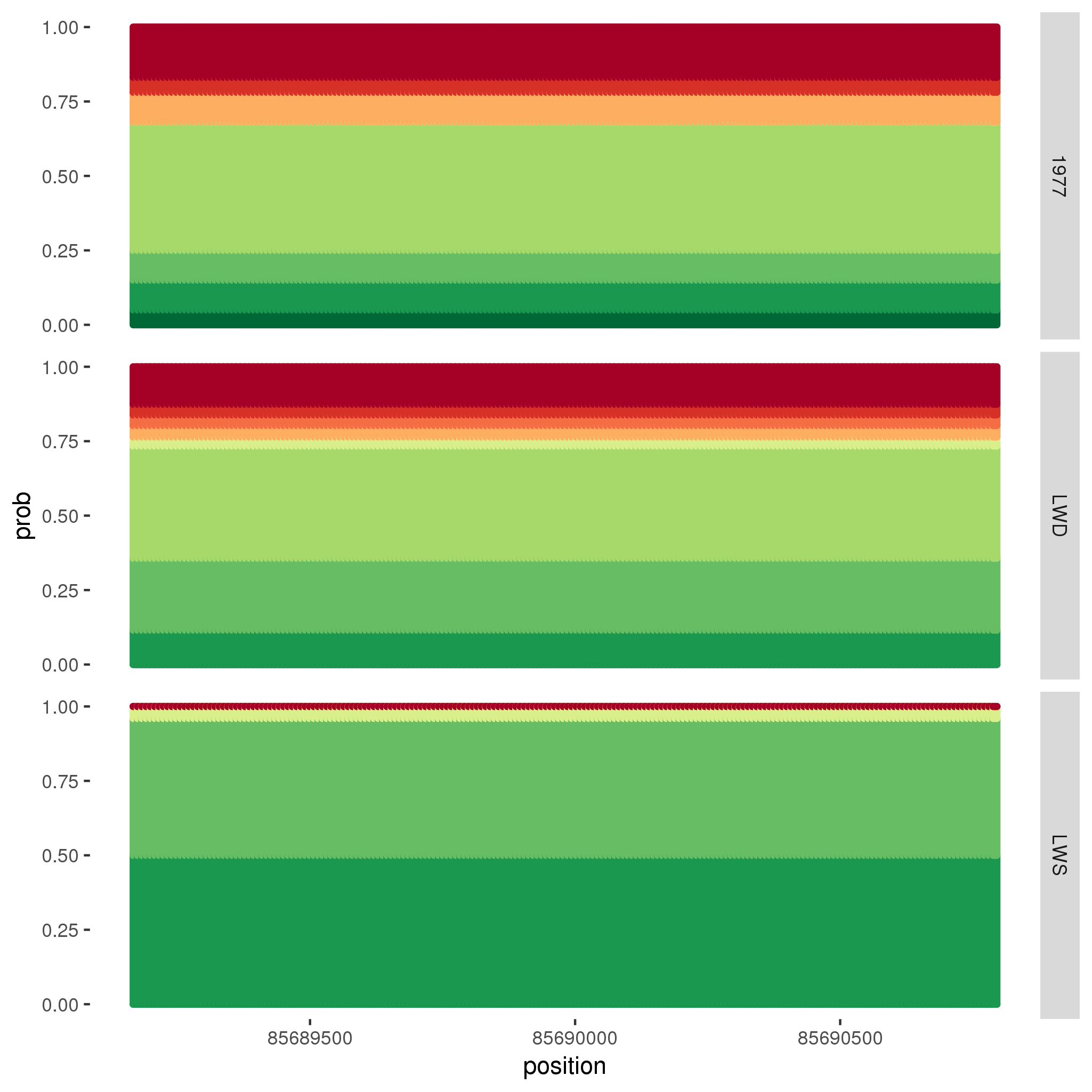

Supplement: Supplementary file 5 — Additional file 5. Haplotype frequency plots for all candidate regions of Table 1. Each file of this archive corresponds to a different candidate region, whose genomic position can be read in the file name. It shows the haplotype diversity in this region, estimated using hapFLK with 10 haplotype clusters (for one single EM run). For a given genomic position, each color corresponds to a different haplotype cluster and the height of the color band in a given population (represented by one of the panels) gives the frequency of the cluster in this population. All variants (AV set) were considered for these analyses. [file 12711_2023_789_MOESM5_ESM.xz › hapflk_plots/all10_auto_chro7_85689170-85690792.kfrq.fit_2.bz2-plot.png]

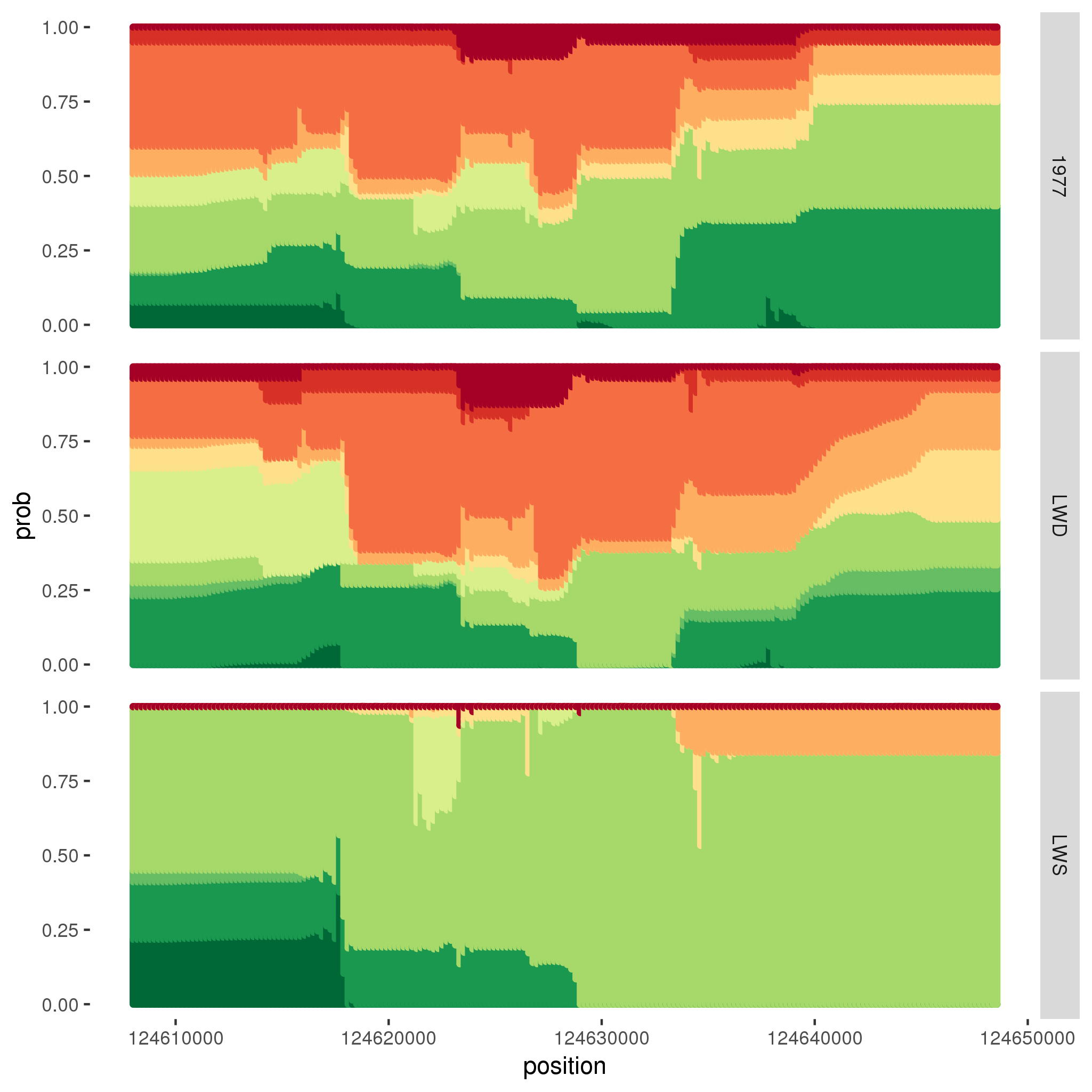

Supplement: Supplementary file 5 — Additional file 5. Haplotype frequency plots for all candidate regions of Table 1. Each file of this archive corresponds to a different candidate region, whose genomic position can be read in the file name. It shows the haplotype diversity in this region, estimated using hapFLK with 10 haplotype clusters (for one single EM run). For a given genomic position, each color corresponds to a different haplotype cluster and the height of the color band in a given population (represented by one of the panels) gives the frequency of the cluster in this population. All variants (AV set) were considered for these analyses. [file 12711_2023_789_MOESM5_ESM.xz › hapflk_plots/all10_auto_chro8_124628076-124628464.kfrq.fit_0.bz2-plot.png]

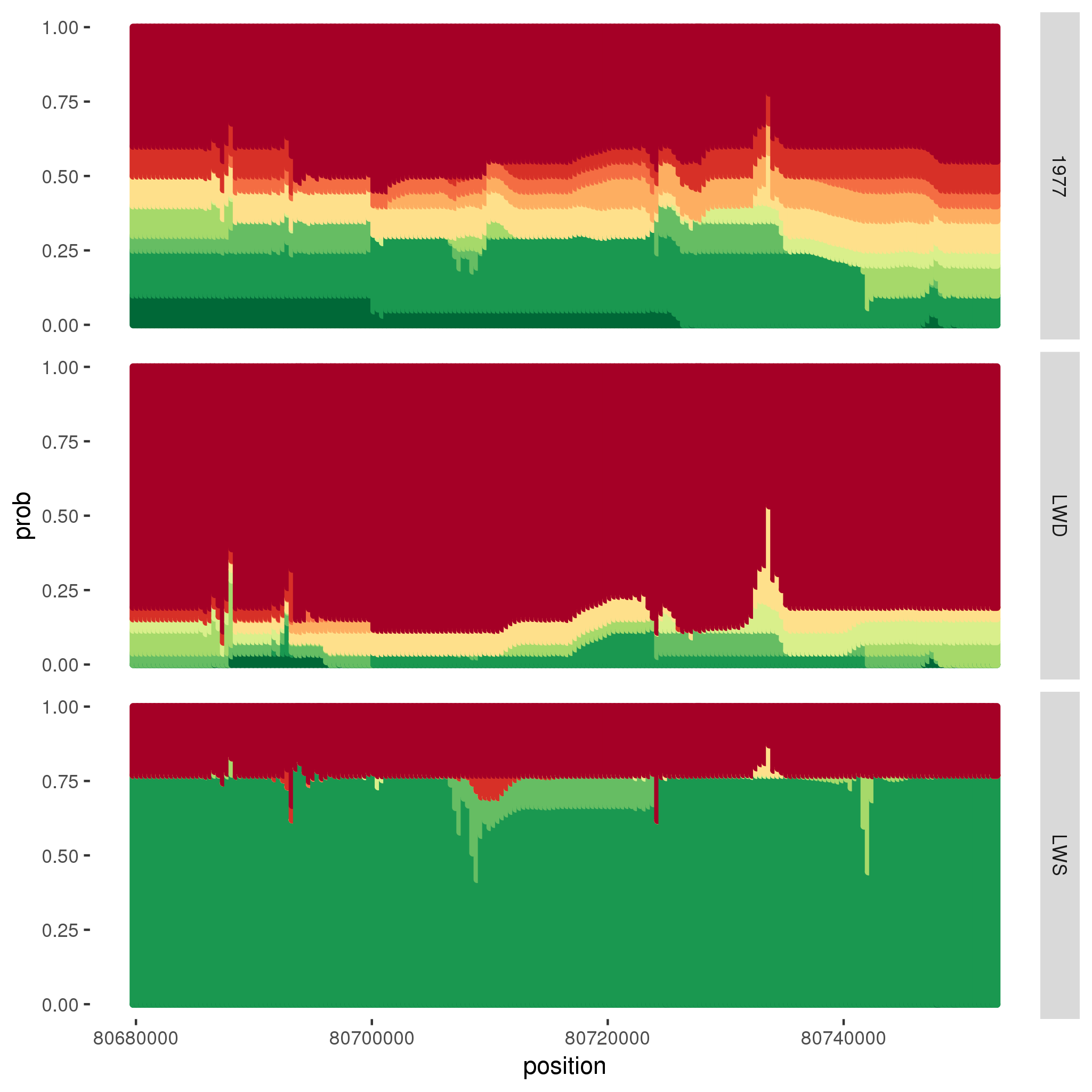

Supplement: Supplementary file 5 — Additional file 5. Haplotype frequency plots for all candidate regions of Table 1. Each file of this archive corresponds to a different candidate region, whose genomic position can be read in the file name. It shows the haplotype diversity in this region, estimated using hapFLK with 10 haplotype clusters (for one single EM run). For a given genomic position, each color corresponds to a different haplotype cluster and the height of the color band in a given population (represented by one of the panels) gives the frequency of the cluster in this population. All variants (AV set) were considered for these analyses. [file 12711_2023_789_MOESM5_ESM.xz › hapflk_plots/all10_auto_chro8_80679925-80752831.kfrq.fit_2.bz2-plot.png]

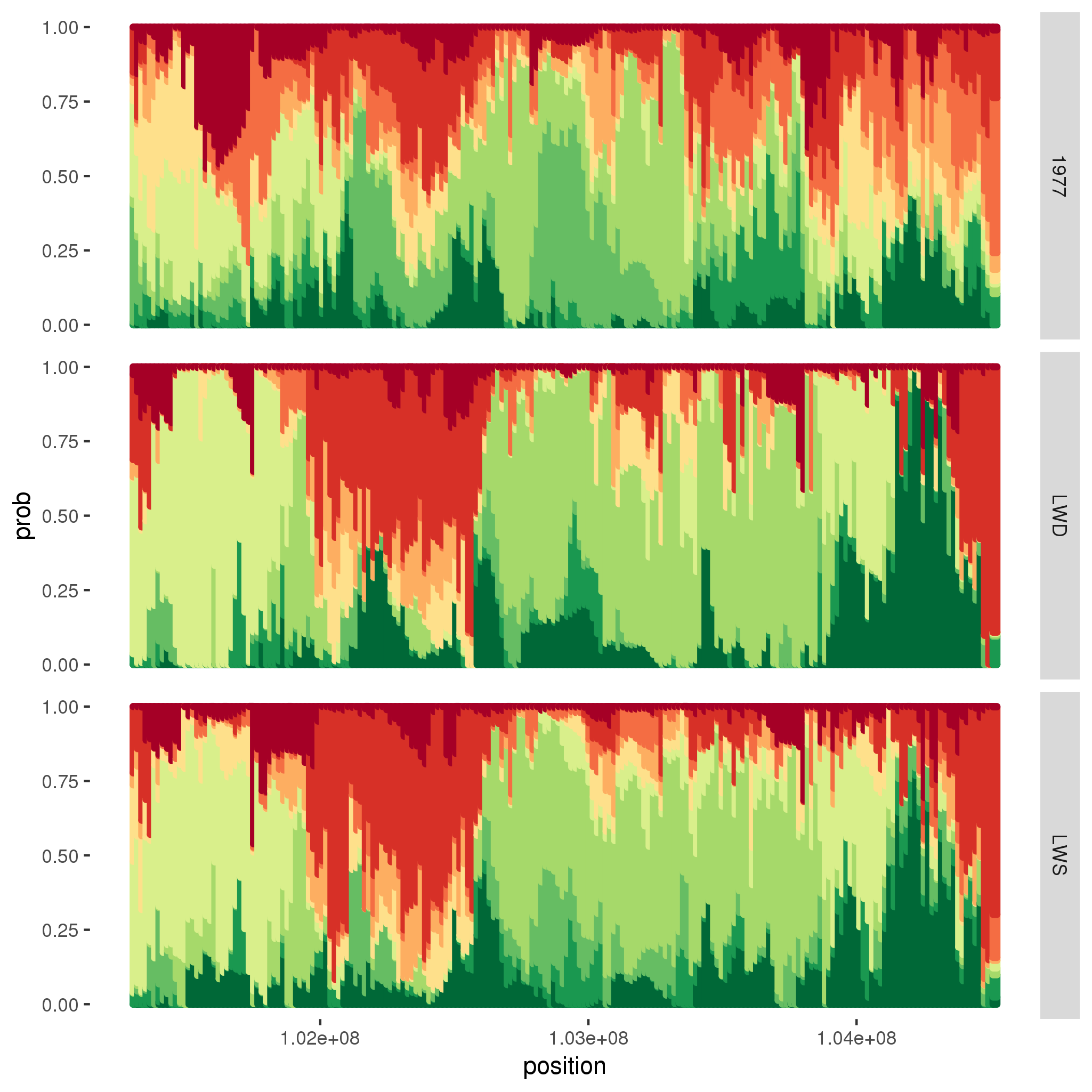

Supplement: Supplementary file 5 — Additional file 5. Haplotype frequency plots for all candidate regions of Table 1. Each file of this archive corresponds to a different candidate region, whose genomic position can be read in the file name. It shows the haplotype diversity in this region, estimated using hapFLK with 10 haplotype clusters (for one single EM run). For a given genomic position, each color corresponds to a different haplotype cluster and the height of the color band in a given population (represented by one of the panels) gives the frequency of the cluster in this population. All variants (AV set) were considered for these analyses. [file 12711_2023_789_MOESM5_ESM.xz › hapflk_plots/all10_auto_chro9_101309610-104516170.kfrq.fit_0.bz2-plot.png]

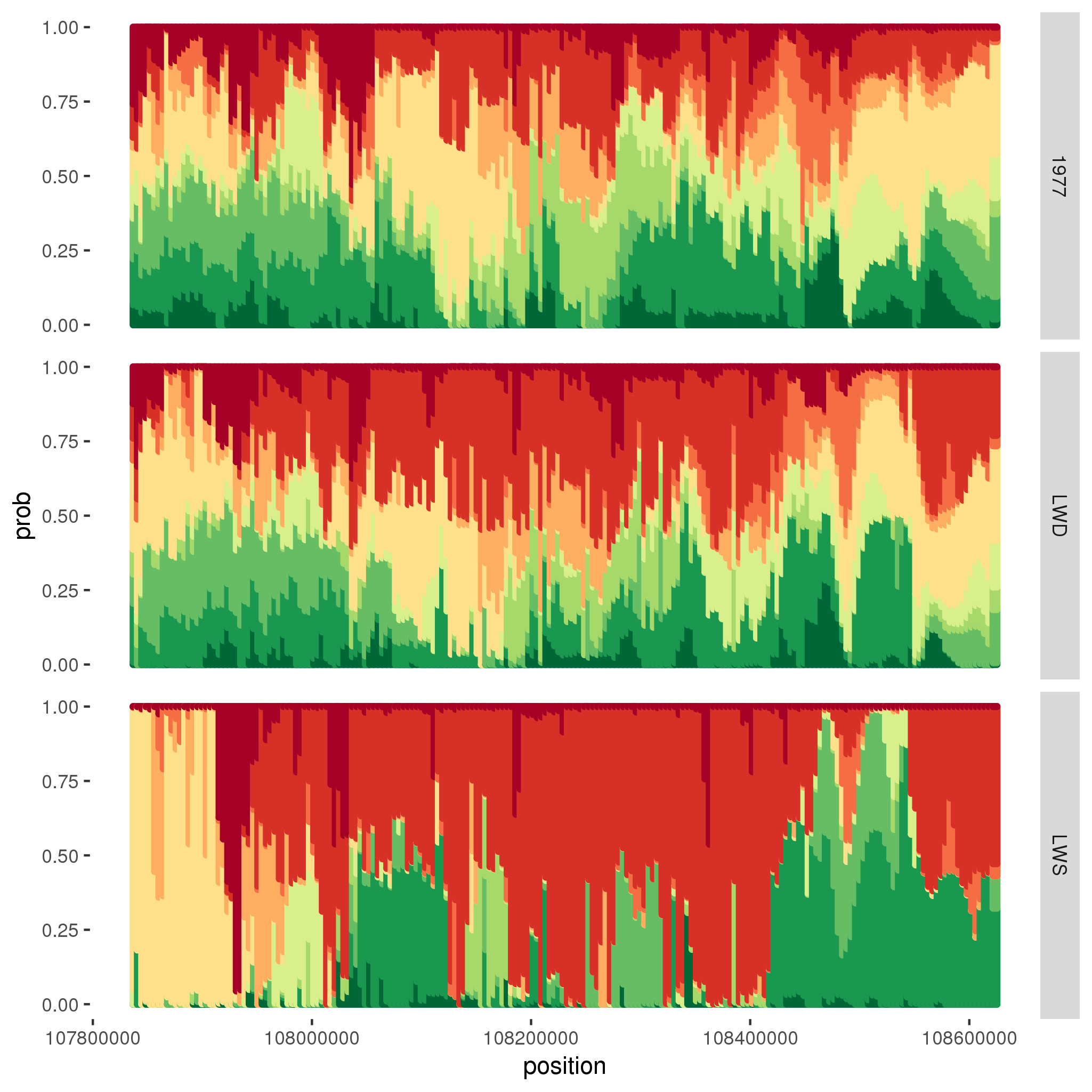

Supplement: Supplementary file 5 — Additional file 5. Haplotype frequency plots for all candidate regions of Table 1. Each file of this archive corresponds to a different candidate region, whose genomic position can be read in the file name. It shows the haplotype diversity in this region, estimated using hapFLK with 10 haplotype clusters (for one single EM run). For a given genomic position, each color corresponds to a different haplotype cluster and the height of the color band in a given population (represented by one of the panels) gives the frequency of the cluster in this population. All variants (AV set) were considered for these analyses. [file 12711_2023_789_MOESM5_ESM.xz › hapflk_plots/all10_auto_chro9_107838551-108623449.kfrq.fit_2.bz2-plot.png]

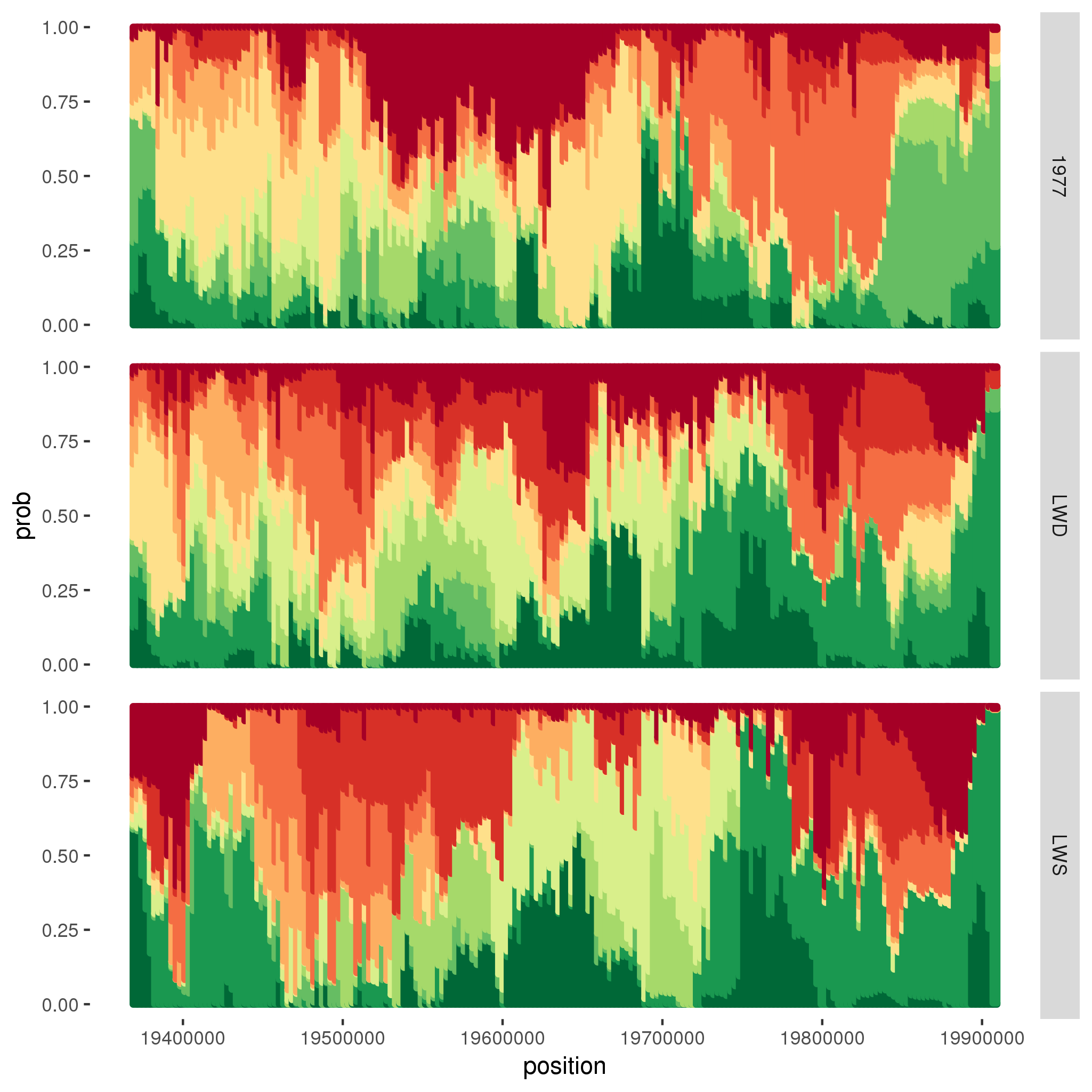

Supplement: Supplementary file 5 — Additional file 5. Haplotype frequency plots for all candidate regions of Table 1. Each file of this archive corresponds to a different candidate region, whose genomic position can be read in the file name. It shows the haplotype diversity in this region, estimated using hapFLK with 10 haplotype clusters (for one single EM run). For a given genomic position, each color corresponds to a different haplotype cluster and the height of the color band in a given population (represented by one of the panels) gives the frequency of the cluster in this population. All variants (AV set) were considered for these analyses. [file 12711_2023_789_MOESM5_ESM.xz › hapflk_plots/all10_auto_chro9_19370051-19908097.kfrq.fit_0.bz2-plot.png]

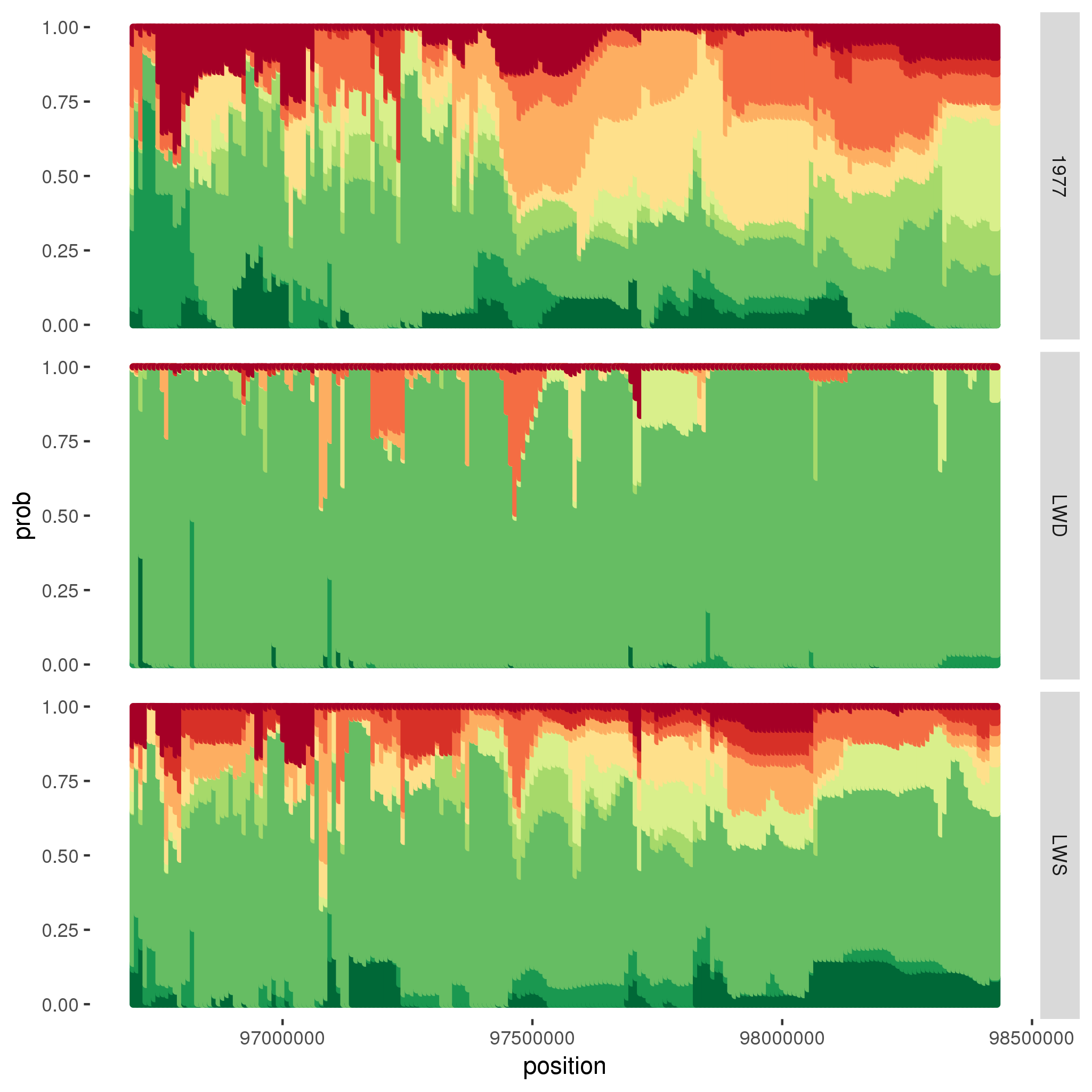

Supplement: Supplementary file 5 — Additional file 5. Haplotype frequency plots for all candidate regions of Table 1. Each file of this archive corresponds to a different candidate region, whose genomic position can be read in the file name. It shows the haplotype diversity in this region, estimated using hapFLK with 10 haplotype clusters (for one single EM run). For a given genomic position, each color corresponds to a different haplotype cluster and the height of the color band in a given population (represented by one of the panels) gives the frequency of the cluster in this population. All variants (AV set) were considered for these analyses. [file 12711_2023_789_MOESM5_ESM.xz › hapflk_plots/all10_auto_chro9_96704786-98425852.kfrq.fit_1.bz2-plot.png]

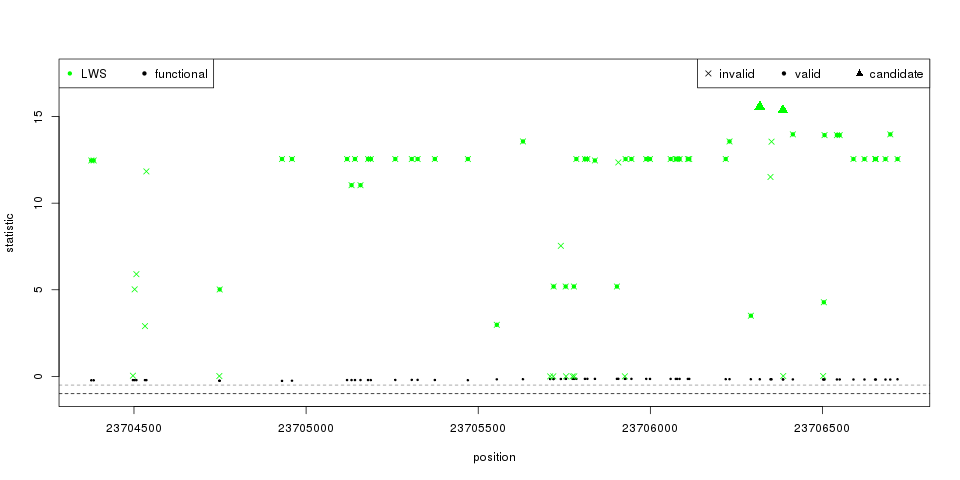

Supplement: Supplementary file 7 — Additional file 7. Candidate causal variants in each candidate region under selection. This archive contains three types of results. (i) Each file with suffix .annot corresponds to a different candidate region, whose genomic position can be read in the file name. It provides a list of candidate variants detected by the statistical approach in this region, together with their SnpEff annotation. (ii) The all strong.func file provides a list of candidate variants with a functional effect (according to SnpEff) selected by this approach for all regions, together with their annotation. The all weak.func file provides the same, but for variants that could only be detected by the functional approach. (iii) Each file with the suffix .png corresponds to a different candidate region, whose genomic position can be read in the file name. It provides a plot showing the values of the relevant statistic(s) in this region with additional details concerning the status of each variant: ‘invalid’ (i.e. with low call rate or allele frequency patterns inconsistent with the type of selection in the region), ‘valid’ (high call rate and consistent allele frequency patterns) or ‘candidate’ (those ‘valid’ variants with the highest value of the statistic, also listed in the .annot file). When functional candidate variants exist in a region, these are plotted in black, while all other variants are plotted in a color depending on the test represented. Finally, the rate of missing genotypes in sliding windows of 2 kb are represented by the black points on the negative part of the y axis, from 0 (no missing data) to − 1 (only missing data). Horizontal dashed lines at − 0.5 and − 1 help visualize the missing rates achieved at a given position. [file 12711_2023_789_MOESM7_ESM.xz › causal/all10_auto_chro10_23704376-23706718_strong.png]

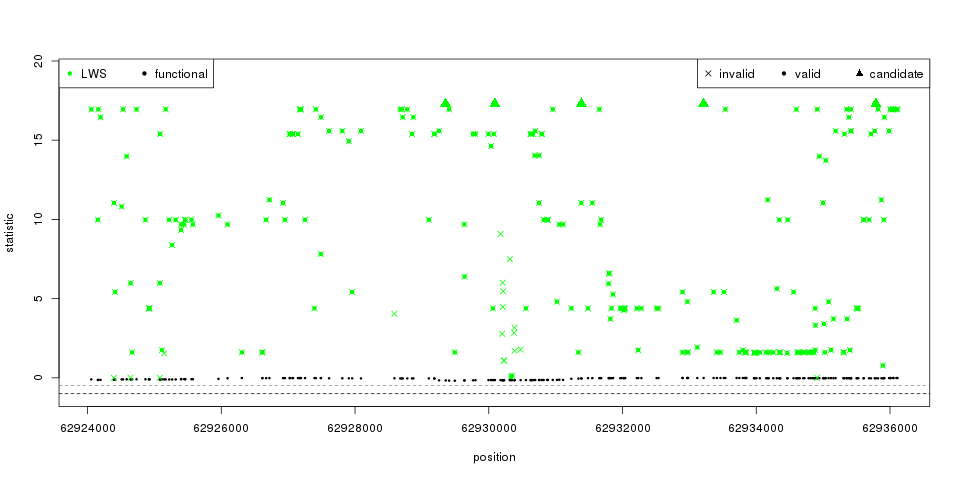

Supplement: Supplementary file 7 — Additional file 7. Candidate causal variants in each candidate region under selection. This archive contains three types of results. (i) Each file with suffix .annot corresponds to a different candidate region, whose genomic position can be read in the file name. It provides a list of candidate variants detected by the statistical approach in this region, together with their SnpEff annotation. (ii) The all strong.func file provides a list of candidate variants with a functional effect (according to SnpEff) selected by this approach for all regions, together with their annotation. The all weak.func file provides the same, but for variants that could only be detected by the functional approach. (iii) Each file with the suffix .png corresponds to a different candidate region, whose genomic position can be read in the file name. It provides a plot showing the values of the relevant statistic(s) in this region with additional details concerning the status of each variant: ‘invalid’ (i.e. with low call rate or allele frequency patterns inconsistent with the type of selection in the region), ‘valid’ (high call rate and consistent allele frequency patterns) or ‘candidate’ (those ‘valid’ variants with the highest value of the statistic, also listed in the .annot file). When functional candidate variants exist in a region, these are plotted in black, while all other variants are plotted in a color depending on the test represented. Finally, the rate of missing genotypes in sliding windows of 2 kb are represented by the black points on the negative part of the y axis, from 0 (no missing data) to − 1 (only missing data). Horizontal dashed lines at − 0.5 and − 1 help visualize the missing rates achieved at a given position. [file 12711_2023_789_MOESM7_ESM.xz › causal/all10_auto_chro10_62924056-62936112_strong.png]

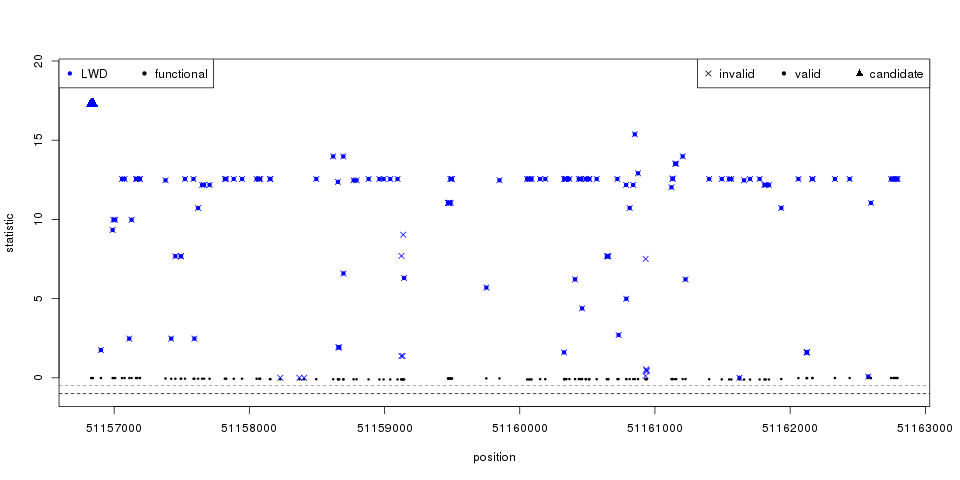

Supplement: Supplementary file 7 — Additional file 7. Candidate causal variants in each candidate region under selection. This archive contains three types of results. (i) Each file with suffix .annot corresponds to a different candidate region, whose genomic position can be read in the file name. It provides a list of candidate variants detected by the statistical approach in this region, together with their SnpEff annotation. (ii) The all strong.func file provides a list of candidate variants with a functional effect (according to SnpEff) selected by this approach for all regions, together with their annotation. The all weak.func file provides the same, but for variants that could only be detected by the functional approach. (iii) Each file with the suffix .png corresponds to a different candidate region, whose genomic position can be read in the file name. It provides a plot showing the values of the relevant statistic(s) in this region with additional details concerning the status of each variant: ‘invalid’ (i.e. with low call rate or allele frequency patterns inconsistent with the type of selection in the region), ‘valid’ (high call rate and consistent allele frequency patterns) or ‘candidate’ (those ‘valid’ variants with the highest value of the statistic, also listed in the .annot file). When functional candidate variants exist in a region, these are plotted in black, while all other variants are plotted in a color depending on the test represented. Finally, the rate of missing genotypes in sliding windows of 2 kb are represented by the black points on the negative part of the y axis, from 0 (no missing data) to − 1 (only missing data). Horizontal dashed lines at − 0.5 and − 1 help visualize the missing rates achieved at a given position. [file 12711_2023_789_MOESM7_ESM.xz › causal/all10_auto_chro11_51156831-51162794_strong.png]

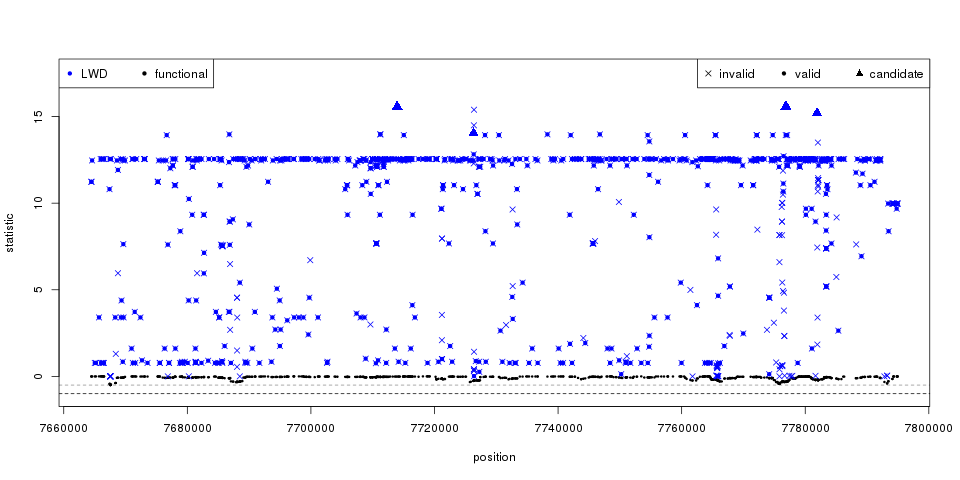

Supplement: Supplementary file 7 — Additional file 7. Candidate causal variants in each candidate region under selection. This archive contains three types of results. (i) Each file with suffix .annot corresponds to a different candidate region, whose genomic position can be read in the file name. It provides a list of candidate variants detected by the statistical approach in this region, together with their SnpEff annotation. (ii) The all strong.func file provides a list of candidate variants with a functional effect (according to SnpEff) selected by this approach for all regions, together with their annotation. The all weak.func file provides the same, but for variants that could only be detected by the functional approach. (iii) Each file with the suffix .png corresponds to a different candidate region, whose genomic position can be read in the file name. It provides a plot showing the values of the relevant statistic(s) in this region with additional details concerning the status of each variant: ‘invalid’ (i.e. with low call rate or allele frequency patterns inconsistent with the type of selection in the region), ‘valid’ (high call rate and consistent allele frequency patterns) or ‘candidate’ (those ‘valid’ variants with the highest value of the statistic, also listed in the .annot file). When functional candidate variants exist in a region, these are plotted in black, while all other variants are plotted in a color depending on the test represented. Finally, the rate of missing genotypes in sliding windows of 2 kb are represented by the black points on the negative part of the y axis, from 0 (no missing data) to − 1 (only missing data). Horizontal dashed lines at − 0.5 and − 1 help visualize the missing rates achieved at a given position. [file 12711_2023_789_MOESM7_ESM.xz › causal/all10_auto_chro11_7664458-7794933_strong.png]

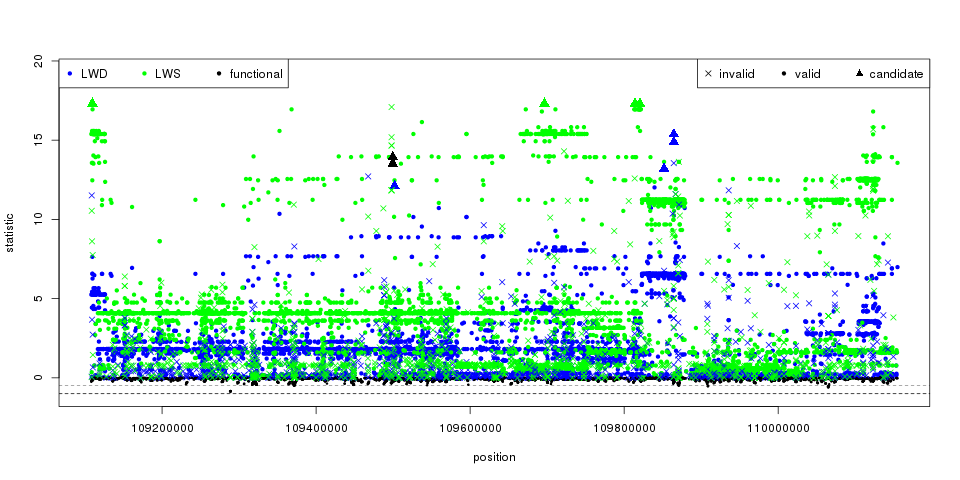

Supplement: Supplementary file 7 — Additional file 7. Candidate causal variants in each candidate region under selection. This archive contains three types of results. (i) Each file with suffix .annot corresponds to a different candidate region, whose genomic position can be read in the file name. It provides a list of candidate variants detected by the statistical approach in this region, together with their SnpEff annotation. (ii) The all strong.func file provides a list of candidate variants with a functional effect (according to SnpEff) selected by this approach for all regions, together with their annotation. The all weak.func file provides the same, but for variants that could only be detected by the functional approach. (iii) Each file with the suffix .png corresponds to a different candidate region, whose genomic position can be read in the file name. It provides a plot showing the values of the relevant statistic(s) in this region with additional details concerning the status of each variant: ‘invalid’ (i.e. with low call rate or allele frequency patterns inconsistent with the type of selection in the region), ‘valid’ (high call rate and consistent allele frequency patterns) or ‘candidate’ (those ‘valid’ variants with the highest value of the statistic, also listed in the .annot file). When functional candidate variants exist in a region, these are plotted in black, while all other variants are plotted in a color depending on the test represented. Finally, the rate of missing genotypes in sliding windows of 2 kb are represented by the black points on the negative part of the y axis, from 0 (no missing data) to − 1 (only missing data). Horizontal dashed lines at − 0.5 and − 1 help visualize the missing rates achieved at a given position. [file 12711_2023_789_MOESM7_ESM.xz › causal/all10_auto_chro13_109108079-110154951_strong.png]

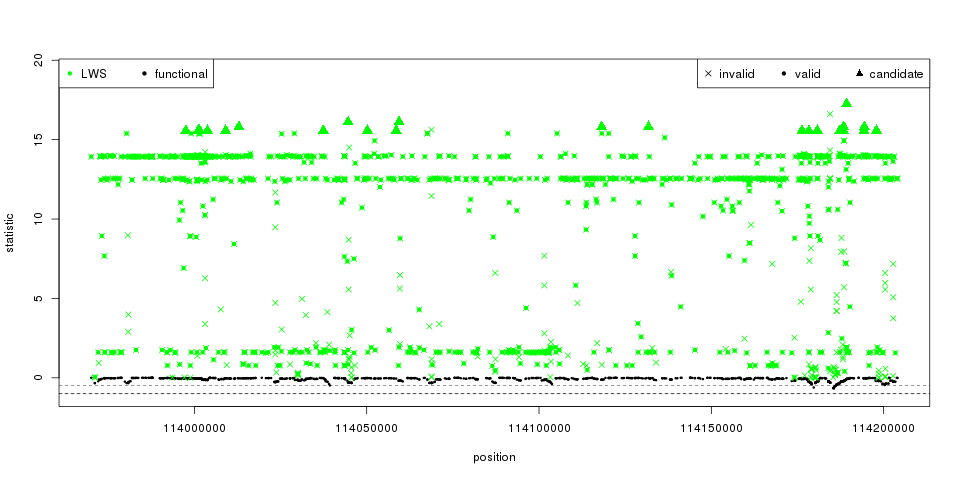

Supplement: Supplementary file 7 — Additional file 7. Candidate causal variants in each candidate region under selection. This archive contains three types of results. (i) Each file with suffix .annot corresponds to a different candidate region, whose genomic position can be read in the file name. It provides a list of candidate variants detected by the statistical approach in this region, together with their SnpEff annotation. (ii) The all strong.func file provides a list of candidate variants with a functional effect (according to SnpEff) selected by this approach for all regions, together with their annotation. The all weak.func file provides the same, but for variants that could only be detected by the functional approach. (iii) Each file with the suffix .png corresponds to a different candidate region, whose genomic position can be read in the file name. It provides a plot showing the values of the relevant statistic(s) in this region with additional details concerning the status of each variant: ‘invalid’ (i.e. with low call rate or allele frequency patterns inconsistent with the type of selection in the region), ‘valid’ (high call rate and consistent allele frequency patterns) or ‘candidate’ (those ‘valid’ variants with the highest value of the statistic, also listed in the .annot file). When functional candidate variants exist in a region, these are plotted in black, while all other variants are plotted in a color depending on the test represented. Finally, the rate of missing genotypes in sliding windows of 2 kb are represented by the black points on the negative part of the y axis, from 0 (no missing data) to − 1 (only missing data). Horizontal dashed lines at − 0.5 and − 1 help visualize the missing rates achieved at a given position. [file 12711_2023_789_MOESM7_ESM.xz › causal/all10_auto_chro13_113970091-114204018_strong.png]

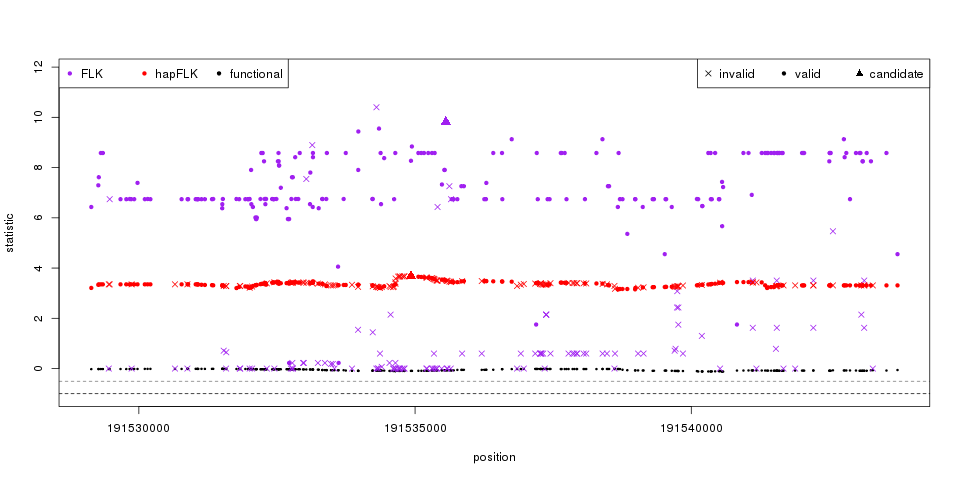

Supplement: Supplementary file 7 — Additional file 7. Candidate causal variants in each candidate region under selection. This archive contains three types of results. (i) Each file with suffix .annot corresponds to a different candidate region, whose genomic position can be read in the file name. It provides a list of candidate variants detected by the statistical approach in this region, together with their SnpEff annotation. (ii) The all strong.func file provides a list of candidate variants with a functional effect (according to SnpEff) selected by this approach for all regions, together with their annotation. The all weak.func file provides the same, but for variants that could only be detected by the functional approach. (iii) Each file with the suffix .png corresponds to a different candidate region, whose genomic position can be read in the file name. It provides a plot showing the values of the relevant statistic(s) in this region with additional details concerning the status of each variant: ‘invalid’ (i.e. with low call rate or allele frequency patterns inconsistent with the type of selection in the region), ‘valid’ (high call rate and consistent allele frequency patterns) or ‘candidate’ (those ‘valid’ variants with the highest value of the statistic, also listed in the .annot file). When functional candidate variants exist in a region, these are plotted in black, while all other variants are plotted in a color depending on the test represented. Finally, the rate of missing genotypes in sliding windows of 2 kb are represented by the black points on the negative part of the y axis, from 0 (no missing data) to − 1 (only missing data). Horizontal dashed lines at − 0.5 and − 1 help visualize the missing rates achieved at a given position. [file 12711_2023_789_MOESM7_ESM.xz › causal/all10_auto_chro13_191529139-191543733_strong.png]

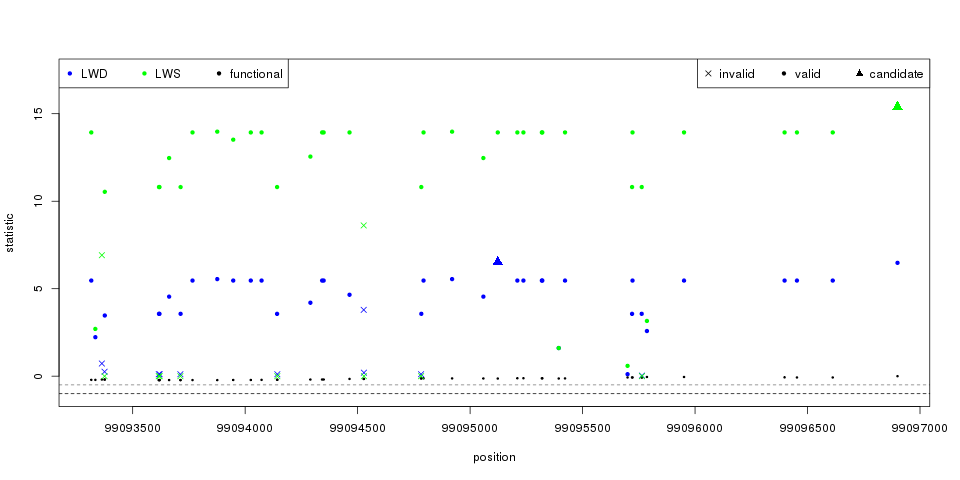

Supplement: Supplementary file 7 — Additional file 7. Candidate causal variants in each candidate region under selection. This archive contains three types of results. (i) Each file with suffix .annot corresponds to a different candidate region, whose genomic position can be read in the file name. It provides a list of candidate variants detected by the statistical approach in this region, together with their SnpEff annotation. (ii) The all strong.func file provides a list of candidate variants with a functional effect (according to SnpEff) selected by this approach for all regions, together with their annotation. The all weak.func file provides the same, but for variants that could only be detected by the functional approach. (iii) Each file with the suffix .png corresponds to a different candidate region, whose genomic position can be read in the file name. It provides a plot showing the values of the relevant statistic(s) in this region with additional details concerning the status of each variant: ‘invalid’ (i.e. with low call rate or allele frequency patterns inconsistent with the type of selection in the region), ‘valid’ (high call rate and consistent allele frequency patterns) or ‘candidate’ (those ‘valid’ variants with the highest value of the statistic, also listed in the .annot file). When functional candidate variants exist in a region, these are plotted in black, while all other variants are plotted in a color depending on the test represented. Finally, the rate of missing genotypes in sliding windows of 2 kb are represented by the black points on the negative part of the y axis, from 0 (no missing data) to − 1 (only missing data). Horizontal dashed lines at − 0.5 and − 1 help visualize the missing rates achieved at a given position. [file 12711_2023_789_MOESM7_ESM.xz › causal/all10_auto_chro13_99093316-99096900_strong.png]

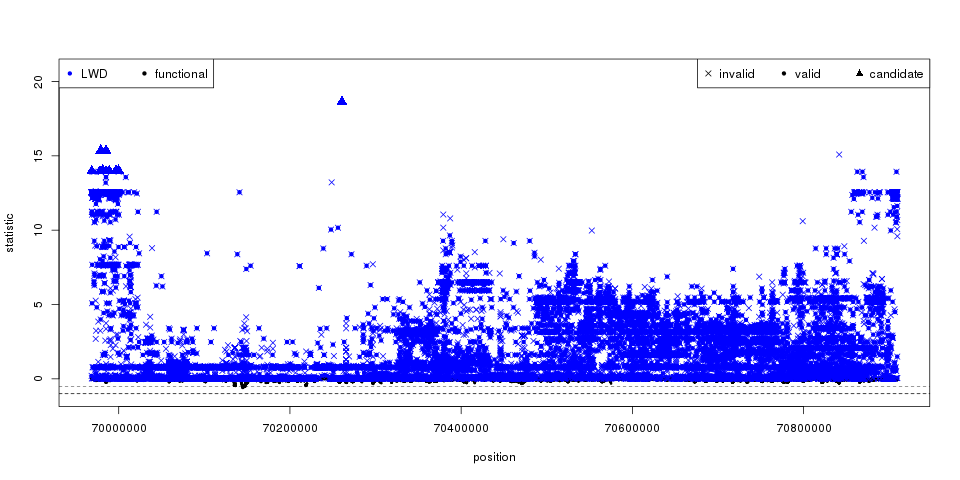

Supplement: Supplementary file 7 — Additional file 7. Candidate causal variants in each candidate region under selection. This archive contains three types of results. (i) Each file with suffix .annot corresponds to a different candidate region, whose genomic position can be read in the file name. It provides a list of candidate variants detected by the statistical approach in this region, together with their SnpEff annotation. (ii) The all strong.func file provides a list of candidate variants with a functional effect (according to SnpEff) selected by this approach for all regions, together with their annotation. The all weak.func file provides the same, but for variants that could only be detected by the functional approach. (iii) Each file with the suffix .png corresponds to a different candidate region, whose genomic position can be read in the file name. It provides a plot showing the values of the relevant statistic(s) in this region with additional details concerning the status of each variant: ‘invalid’ (i.e. with low call rate or allele frequency patterns inconsistent with the type of selection in the region), ‘valid’ (high call rate and consistent allele frequency patterns) or ‘candidate’ (those ‘valid’ variants with the highest value of the statistic, also listed in the .annot file). When functional candidate variants exist in a region, these are plotted in black, while all other variants are plotted in a color depending on the test represented. Finally, the rate of missing genotypes in sliding windows of 2 kb are represented by the black points on the negative part of the y axis, from 0 (no missing data) to − 1 (only missing data). Horizontal dashed lines at − 0.5 and − 1 help visualize the missing rates achieved at a given position. [file 12711_2023_789_MOESM7_ESM.xz › causal/all10_auto_chro16_69967970-70909691_strong.png]

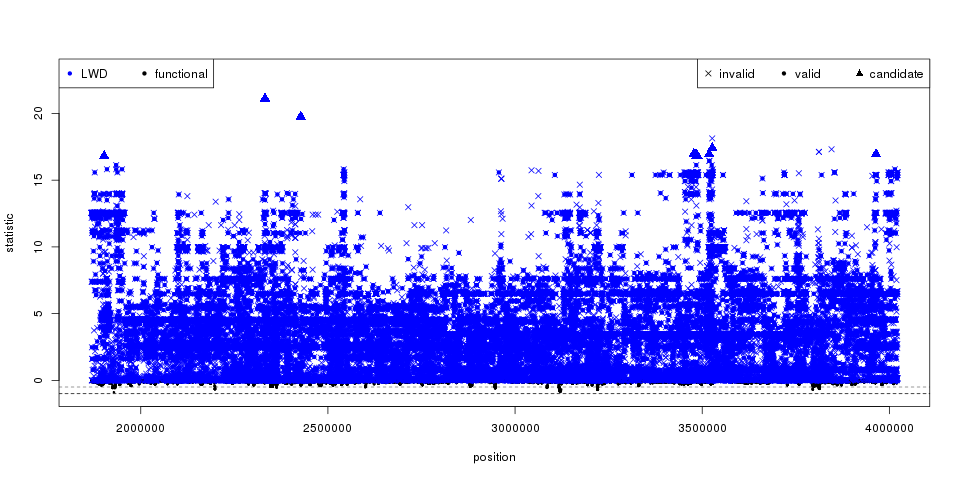

Supplement: Supplementary file 7 — Additional file 7. Candidate causal variants in each candidate region under selection. This archive contains three types of results. (i) Each file with suffix .annot corresponds to a different candidate region, whose genomic position can be read in the file name. It provides a list of candidate variants detected by the statistical approach in this region, together with their SnpEff annotation. (ii) The all strong.func file provides a list of candidate variants with a functional effect (according to SnpEff) selected by this approach for all regions, together with their annotation. The all weak.func file provides the same, but for variants that could only be detected by the functional approach. (iii) Each file with the suffix .png corresponds to a different candidate region, whose genomic position can be read in the file name. It provides a plot showing the values of the relevant statistic(s) in this region with additional details concerning the status of each variant: ‘invalid’ (i.e. with low call rate or allele frequency patterns inconsistent with the type of selection in the region), ‘valid’ (high call rate and consistent allele frequency patterns) or ‘candidate’ (those ‘valid’ variants with the highest value of the statistic, also listed in the .annot file). When functional candidate variants exist in a region, these are plotted in black, while all other variants are plotted in a color depending on the test represented. Finally, the rate of missing genotypes in sliding windows of 2 kb are represented by the black points on the negative part of the y axis, from 0 (no missing data) to − 1 (only missing data). Horizontal dashed lines at − 0.5 and − 1 help visualize the missing rates achieved at a given position. [file 12711_2023_789_MOESM7_ESM.xz › causal/all10_auto_chro17_1867811-4021774_strong.png]

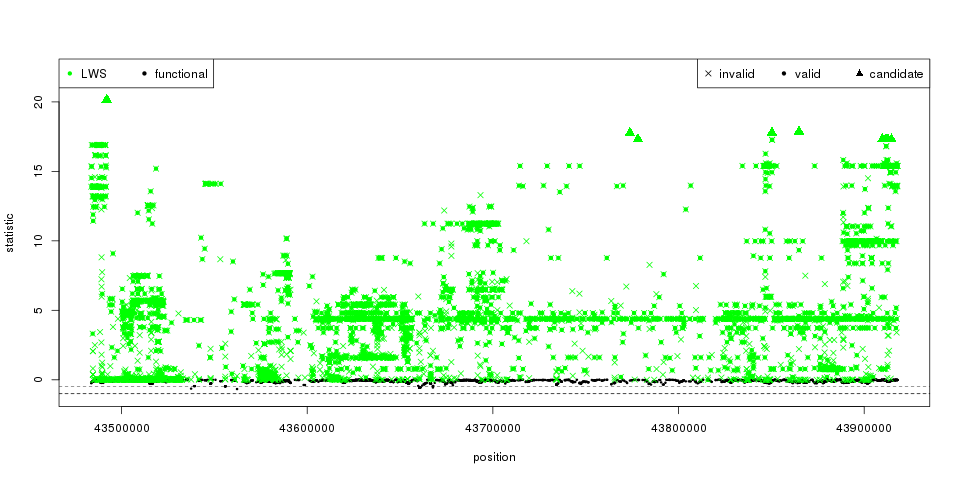

Supplement: Supplementary file 7 — Additional file 7. Candidate causal variants in each candidate region under selection. This archive contains three types of results. (i) Each file with suffix .annot corresponds to a different candidate region, whose genomic position can be read in the file name. It provides a list of candidate variants detected by the statistical approach in this region, together with their SnpEff annotation. (ii) The all strong.func file provides a list of candidate variants with a functional effect (according to SnpEff) selected by this approach for all regions, together with their annotation. The all weak.func file provides the same, but for variants that could only be detected by the functional approach. (iii) Each file with the suffix .png corresponds to a different candidate region, whose genomic position can be read in the file name. It provides a plot showing the values of the relevant statistic(s) in this region with additional details concerning the status of each variant: ‘invalid’ (i.e. with low call rate or allele frequency patterns inconsistent with the type of selection in the region), ‘valid’ (high call rate and consistent allele frequency patterns) or ‘candidate’ (those ‘valid’ variants with the highest value of the statistic, also listed in the .annot file). When functional candidate variants exist in a region, these are plotted in black, while all other variants are plotted in a color depending on the test represented. Finally, the rate of missing genotypes in sliding windows of 2 kb are represented by the black points on the negative part of the y axis, from 0 (no missing data) to − 1 (only missing data). Horizontal dashed lines at − 0.5 and − 1 help visualize the missing rates achieved at a given position. [file 12711_2023_789_MOESM7_ESM.xz › causal/all10_auto_chro17_43483704-43917949_strong.png]

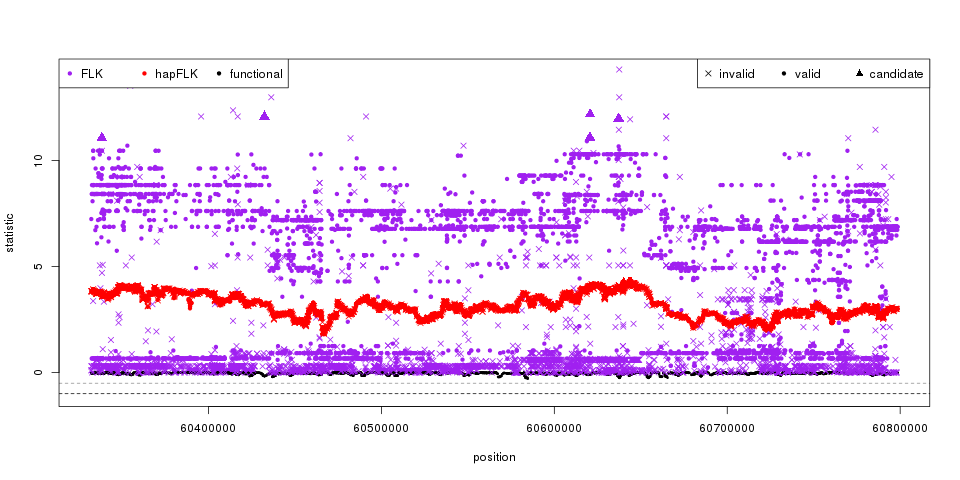

Supplement: Supplementary file 7 — Additional file 7. Candidate causal variants in each candidate region under selection. This archive contains three types of results. (i) Each file with suffix .annot corresponds to a different candidate region, whose genomic position can be read in the file name. It provides a list of candidate variants detected by the statistical approach in this region, together with their SnpEff annotation. (ii) The all strong.func file provides a list of candidate variants with a functional effect (according to SnpEff) selected by this approach for all regions, together with their annotation. The all weak.func file provides the same, but for variants that could only be detected by the functional approach. (iii) Each file with the suffix .png corresponds to a different candidate region, whose genomic position can be read in the file name. It provides a plot showing the values of the relevant statistic(s) in this region with additional details concerning the status of each variant: ‘invalid’ (i.e. with low call rate or allele frequency patterns inconsistent with the type of selection in the region), ‘valid’ (high call rate and consistent allele frequency patterns) or ‘candidate’ (those ‘valid’ variants with the highest value of the statistic, also listed in the .annot file). When functional candidate variants exist in a region, these are plotted in black, while all other variants are plotted in a color depending on the test represented. Finally, the rate of missing genotypes in sliding windows of 2 kb are represented by the black points on the negative part of the y axis, from 0 (no missing data) to − 1 (only missing data). Horizontal dashed lines at − 0.5 and − 1 help visualize the missing rates achieved at a given position. [file 12711_2023_789_MOESM7_ESM.xz › causal/all10_auto_chro17_60332242-60798465_strong.png]

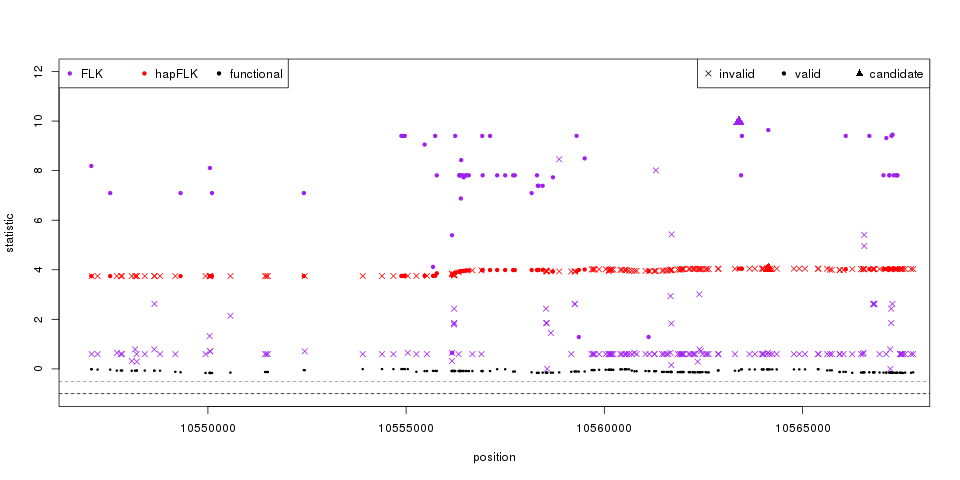

Supplement: Supplementary file 7 — Additional file 7. Candidate causal variants in each candidate region under selection. This archive contains three types of results. (i) Each file with suffix .annot corresponds to a different candidate region, whose genomic position can be read in the file name. It provides a list of candidate variants detected by the statistical approach in this region, together with their SnpEff annotation. (ii) The all strong.func file provides a list of candidate variants with a functional effect (according to SnpEff) selected by this approach for all regions, together with their annotation. The all weak.func file provides the same, but for variants that could only be detected by the functional approach. (iii) Each file with the suffix .png corresponds to a different candidate region, whose genomic position can be read in the file name. It provides a plot showing the values of the relevant statistic(s) in this region with additional details concerning the status of each variant: ‘invalid’ (i.e. with low call rate or allele frequency patterns inconsistent with the type of selection in the region), ‘valid’ (high call rate and consistent allele frequency patterns) or ‘candidate’ (those ‘valid’ variants with the highest value of the statistic, also listed in the .annot file). When functional candidate variants exist in a region, these are plotted in black, while all other variants are plotted in a color depending on the test represented. Finally, the rate of missing genotypes in sliding windows of 2 kb are represented by the black points on the negative part of the y axis, from 0 (no missing data) to − 1 (only missing data). Horizontal dashed lines at − 0.5 and − 1 help visualize the missing rates achieved at a given position. [file 12711_2023_789_MOESM7_ESM.xz › causal/all10_auto_chro18_10547058-10567791_strong.png]

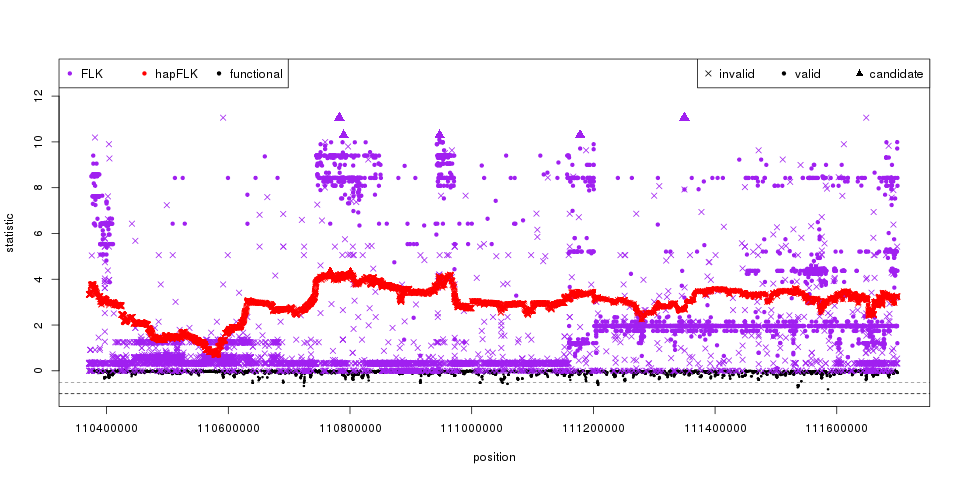

Supplement: Supplementary file 7 — Additional file 7. Candidate causal variants in each candidate region under selection. This archive contains three types of results. (i) Each file with suffix .annot corresponds to a different candidate region, whose genomic position can be read in the file name. It provides a list of candidate variants detected by the statistical approach in this region, together with their SnpEff annotation. (ii) The all strong.func file provides a list of candidate variants with a functional effect (according to SnpEff) selected by this approach for all regions, together with their annotation. The all weak.func file provides the same, but for variants that could only be detected by the functional approach. (iii) Each file with the suffix .png corresponds to a different candidate region, whose genomic position can be read in the file name. It provides a plot showing the values of the relevant statistic(s) in this region with additional details concerning the status of each variant: ‘invalid’ (i.e. with low call rate or allele frequency patterns inconsistent with the type of selection in the region), ‘valid’ (high call rate and consistent allele frequency patterns) or ‘candidate’ (those ‘valid’ variants with the highest value of the statistic, also listed in the .annot file). When functional candidate variants exist in a region, these are plotted in black, while all other variants are plotted in a color depending on the test represented. Finally, the rate of missing genotypes in sliding windows of 2 kb are represented by the black points on the negative part of the y axis, from 0 (no missing data) to − 1 (only missing data). Horizontal dashed lines at − 0.5 and − 1 help visualize the missing rates achieved at a given position. [file 12711_2023_789_MOESM7_ESM.xz › causal/all10_auto_chro1_110370901-111699835_strong.png]

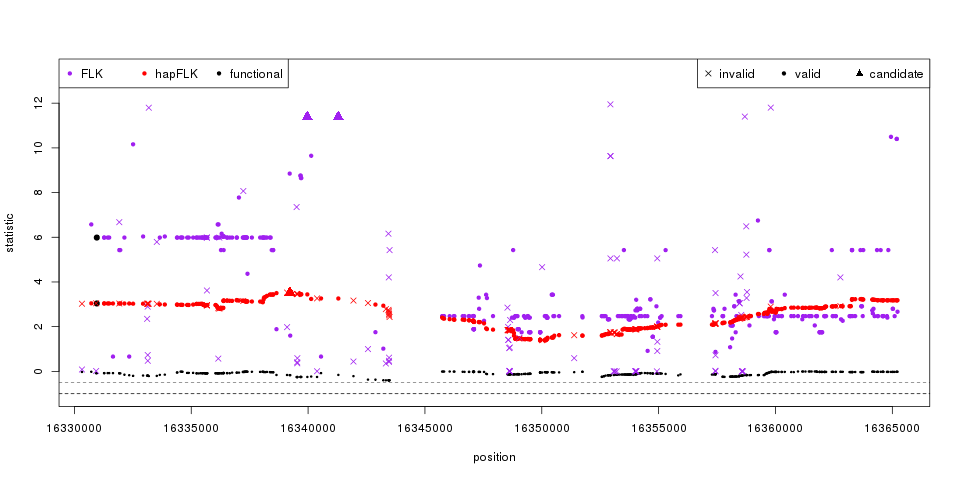

Supplement: Supplementary file 7 — Additional file 7. Candidate causal variants in each candidate region under selection. This archive contains three types of results. (i) Each file with suffix .annot corresponds to a different candidate region, whose genomic position can be read in the file name. It provides a list of candidate variants detected by the statistical approach in this region, together with their SnpEff annotation. (ii) The all strong.func file provides a list of candidate variants with a functional effect (according to SnpEff) selected by this approach for all regions, together with their annotation. The all weak.func file provides the same, but for variants that could only be detected by the functional approach. (iii) Each file with the suffix .png corresponds to a different candidate region, whose genomic position can be read in the file name. It provides a plot showing the values of the relevant statistic(s) in this region with additional details concerning the status of each variant: ‘invalid’ (i.e. with low call rate or allele frequency patterns inconsistent with the type of selection in the region), ‘valid’ (high call rate and consistent allele frequency patterns) or ‘candidate’ (those ‘valid’ variants with the highest value of the statistic, also listed in the .annot file). When functional candidate variants exist in a region, these are plotted in black, while all other variants are plotted in a color depending on the test represented. Finally, the rate of missing genotypes in sliding windows of 2 kb are represented by the black points on the negative part of the y axis, from 0 (no missing data) to − 1 (only missing data). Horizontal dashed lines at − 0.5 and − 1 help visualize the missing rates achieved at a given position. [file 12711_2023_789_MOESM7_ESM.xz › causal/all10_auto_chro1_16330324-16365222_strong.png]

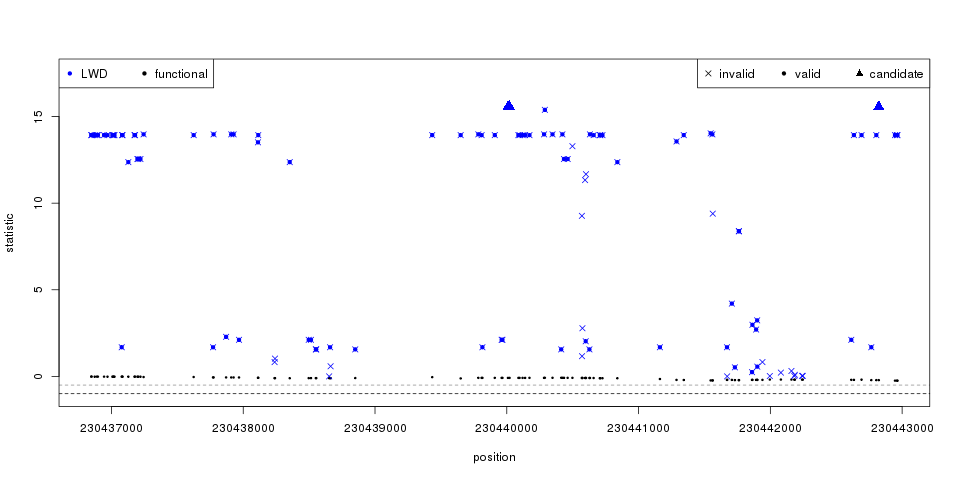

Supplement: Supplementary file 7 — Additional file 7. Candidate causal variants in each candidate region under selection. This archive contains three types of results. (i) Each file with suffix .annot corresponds to a different candidate region, whose genomic position can be read in the file name. It provides a list of candidate variants detected by the statistical approach in this region, together with their SnpEff annotation. (ii) The all strong.func file provides a list of candidate variants with a functional effect (according to SnpEff) selected by this approach for all regions, together with their annotation. The all weak.func file provides the same, but for variants that could only be detected by the functional approach. (iii) Each file with the suffix .png corresponds to a different candidate region, whose genomic position can be read in the file name. It provides a plot showing the values of the relevant statistic(s) in this region with additional details concerning the status of each variant: ‘invalid’ (i.e. with low call rate or allele frequency patterns inconsistent with the type of selection in the region), ‘valid’ (high call rate and consistent allele frequency patterns) or ‘candidate’ (those ‘valid’ variants with the highest value of the statistic, also listed in the .annot file). When functional candidate variants exist in a region, these are plotted in black, while all other variants are plotted in a color depending on the test represented. Finally, the rate of missing genotypes in sliding windows of 2 kb are represented by the black points on the negative part of the y axis, from 0 (no missing data) to − 1 (only missing data). Horizontal dashed lines at − 0.5 and − 1 help visualize the missing rates achieved at a given position. [file 12711_2023_789_MOESM7_ESM.xz › causal/all10_auto_chro1_230436847-230442965_strong.png]

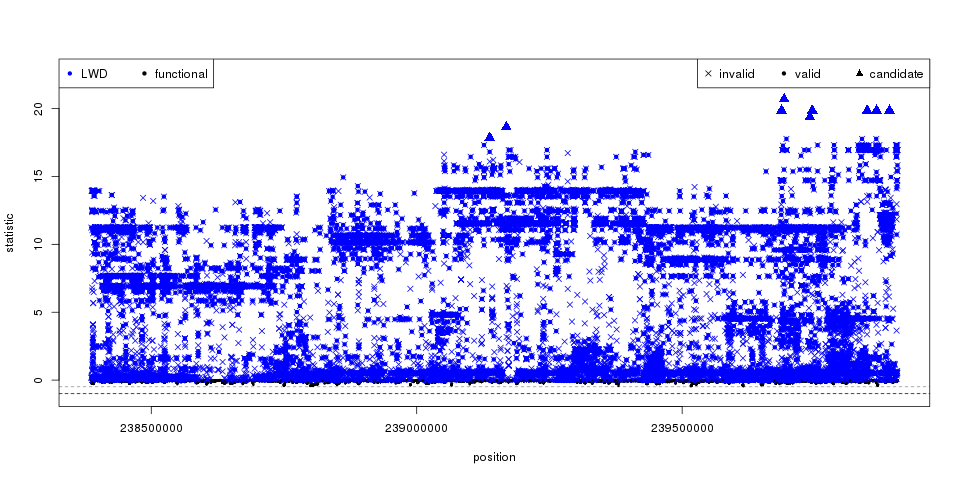

Supplement: Supplementary file 7 — Additional file 7. Candidate causal variants in each candidate region under selection. This archive contains three types of results. (i) Each file with suffix .annot corresponds to a different candidate region, whose genomic position can be read in the file name. It provides a list of candidate variants detected by the statistical approach in this region, together with their SnpEff annotation. (ii) The all strong.func file provides a list of candidate variants with a functional effect (according to SnpEff) selected by this approach for all regions, together with their annotation. The all weak.func file provides the same, but for variants that could only be detected by the functional approach. (iii) Each file with the suffix .png corresponds to a different candidate region, whose genomic position can be read in the file name. It provides a plot showing the values of the relevant statistic(s) in this region with additional details concerning the status of each variant: ‘invalid’ (i.e. with low call rate or allele frequency patterns inconsistent with the type of selection in the region), ‘valid’ (high call rate and consistent allele frequency patterns) or ‘candidate’ (those ‘valid’ variants with the highest value of the statistic, also listed in the .annot file). When functional candidate variants exist in a region, these are plotted in black, while all other variants are plotted in a color depending on the test represented. Finally, the rate of missing genotypes in sliding windows of 2 kb are represented by the black points on the negative part of the y axis, from 0 (no missing data) to − 1 (only missing data). Horizontal dashed lines at − 0.5 and − 1 help visualize the missing rates achieved at a given position. [file 12711_2023_789_MOESM7_ESM.xz › causal/all10_auto_chro1_238387016-239905667_strong.png]

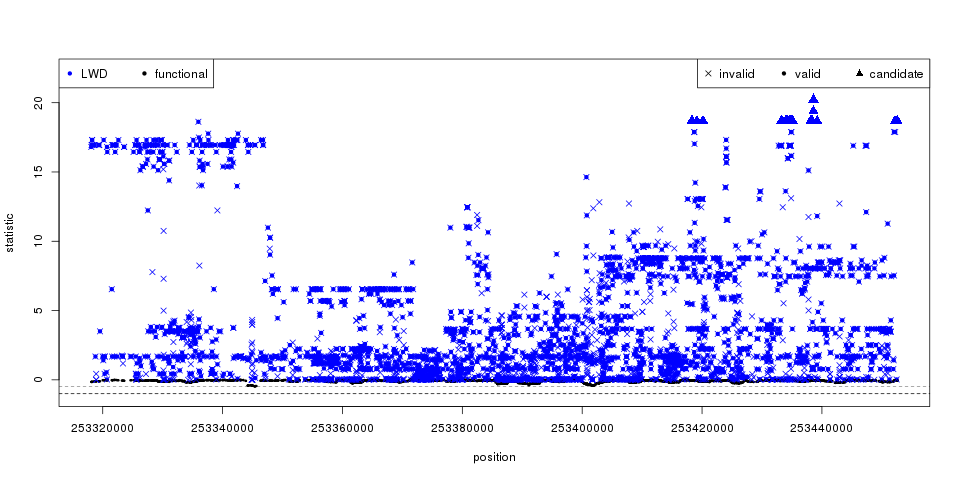

Supplement: Supplementary file 7 — Additional file 7. Candidate causal variants in each candidate region under selection. This archive contains three types of results. (i) Each file with suffix .annot corresponds to a different candidate region, whose genomic position can be read in the file name. It provides a list of candidate variants detected by the statistical approach in this region, together with their SnpEff annotation. (ii) The all strong.func file provides a list of candidate variants with a functional effect (according to SnpEff) selected by this approach for all regions, together with their annotation. The all weak.func file provides the same, but for variants that could only be detected by the functional approach. (iii) Each file with the suffix .png corresponds to a different candidate region, whose genomic position can be read in the file name. It provides a plot showing the values of the relevant statistic(s) in this region with additional details concerning the status of each variant: ‘invalid’ (i.e. with low call rate or allele frequency patterns inconsistent with the type of selection in the region), ‘valid’ (high call rate and consistent allele frequency patterns) or ‘candidate’ (those ‘valid’ variants with the highest value of the statistic, also listed in the .annot file). When functional candidate variants exist in a region, these are plotted in black, while all other variants are plotted in a color depending on the test represented. Finally, the rate of missing genotypes in sliding windows of 2 kb are represented by the black points on the negative part of the y axis, from 0 (no missing data) to − 1 (only missing data). Horizontal dashed lines at − 0.5 and − 1 help visualize the missing rates achieved at a given position. [file 12711_2023_789_MOESM7_ESM.xz › causal/all10_auto_chro1_253318090-253452613_strong.png]

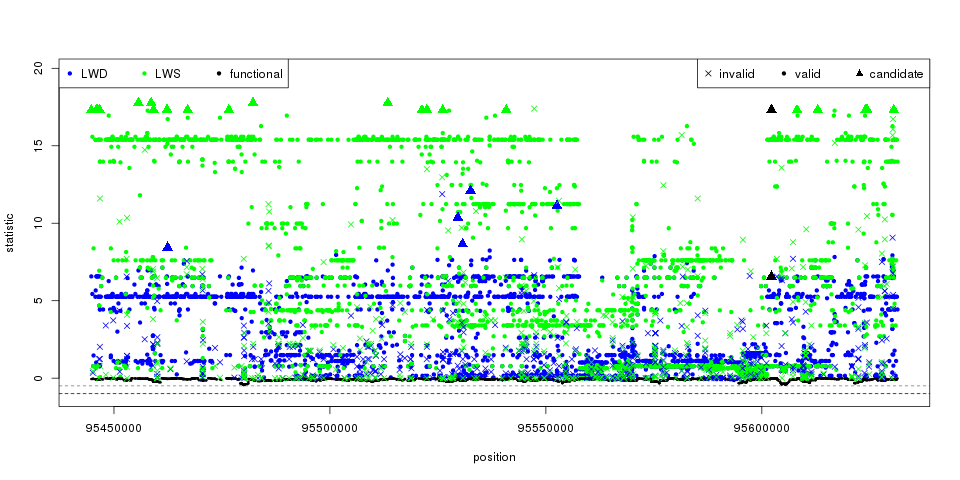

Supplement: Supplementary file 7 — Additional file 7. Candidate causal variants in each candidate region under selection. This archive contains three types of results. (i) Each file with suffix .annot corresponds to a different candidate region, whose genomic position can be read in the file name. It provides a list of candidate variants detected by the statistical approach in this region, together with their SnpEff annotation. (ii) The all strong.func file provides a list of candidate variants with a functional effect (according to SnpEff) selected by this approach for all regions, together with their annotation. The all weak.func file provides the same, but for variants that could only be detected by the functional approach. (iii) Each file with the suffix .png corresponds to a different candidate region, whose genomic position can be read in the file name. It provides a plot showing the values of the relevant statistic(s) in this region with additional details concerning the status of each variant: ‘invalid’ (i.e. with low call rate or allele frequency patterns inconsistent with the type of selection in the region), ‘valid’ (high call rate and consistent allele frequency patterns) or ‘candidate’ (those ‘valid’ variants with the highest value of the statistic, also listed in the .annot file). When functional candidate variants exist in a region, these are plotted in black, while all other variants are plotted in a color depending on the test represented. Finally, the rate of missing genotypes in sliding windows of 2 kb are represented by the black points on the negative part of the y axis, from 0 (no missing data) to − 1 (only missing data). Horizontal dashed lines at − 0.5 and − 1 help visualize the missing rates achieved at a given position. [file 12711_2023_789_MOESM7_ESM.xz › causal/all10_auto_chro1_95444749-95631414_strong.png]

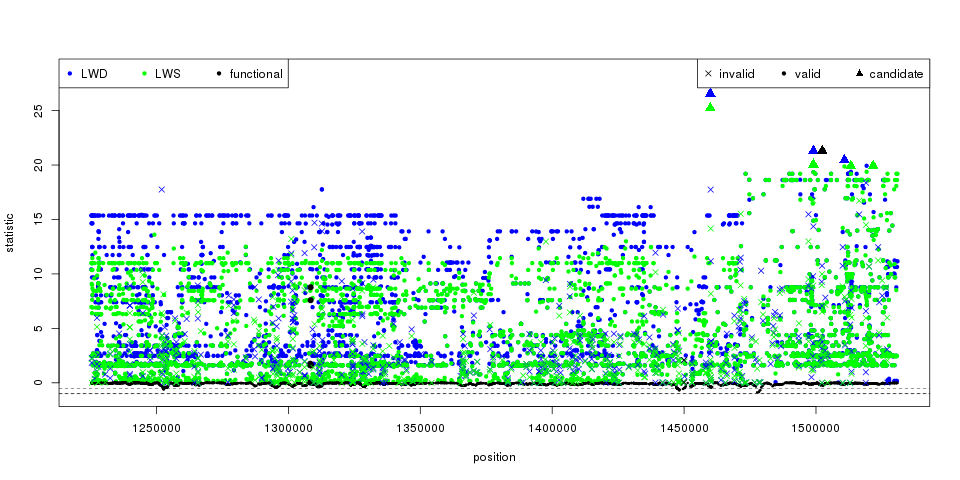

Supplement: Supplementary file 7 — Additional file 7. Candidate causal variants in each candidate region under selection. This archive contains three types of results. (i) Each file with suffix .annot corresponds to a different candidate region, whose genomic position can be read in the file name. It provides a list of candidate variants detected by the statistical approach in this region, together with their SnpEff annotation. (ii) The all strong.func file provides a list of candidate variants with a functional effect (according to SnpEff) selected by this approach for all regions, together with their annotation. The all weak.func file provides the same, but for variants that could only be detected by the functional approach. (iii) Each file with the suffix .png corresponds to a different candidate region, whose genomic position can be read in the file name. It provides a plot showing the values of the relevant statistic(s) in this region with additional details concerning the status of each variant: ‘invalid’ (i.e. with low call rate or allele frequency patterns inconsistent with the type of selection in the region), ‘valid’ (high call rate and consistent allele frequency patterns) or ‘candidate’ (those ‘valid’ variants with the highest value of the statistic, also listed in the .annot file). When functional candidate variants exist in a region, these are plotted in black, while all other variants are plotted in a color depending on the test represented. Finally, the rate of missing genotypes in sliding windows of 2 kb are represented by the black points on the negative part of the y axis, from 0 (no missing data) to − 1 (only missing data). Horizontal dashed lines at − 0.5 and − 1 help visualize the missing rates achieved at a given position. [file 12711_2023_789_MOESM7_ESM.xz › causal/all10_auto_chro2_1225288-1530903_strong.png]

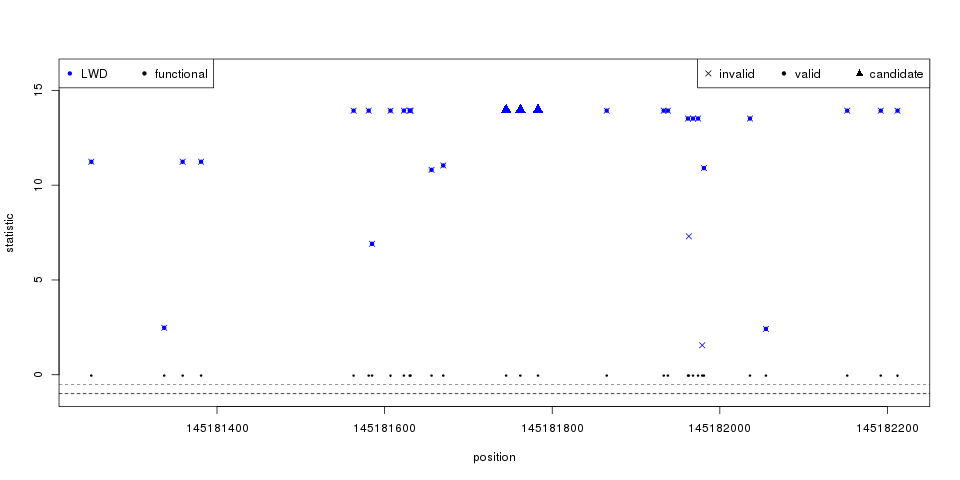

Supplement: Supplementary file 7 — Additional file 7. Candidate causal variants in each candidate region under selection. This archive contains three types of results. (i) Each file with suffix .annot corresponds to a different candidate region, whose genomic position can be read in the file name. It provides a list of candidate variants detected by the statistical approach in this region, together with their SnpEff annotation. (ii) The all strong.func file provides a list of candidate variants with a functional effect (according to SnpEff) selected by this approach for all regions, together with their annotation. The all weak.func file provides the same, but for variants that could only be detected by the functional approach. (iii) Each file with the suffix .png corresponds to a different candidate region, whose genomic position can be read in the file name. It provides a plot showing the values of the relevant statistic(s) in this region with additional details concerning the status of each variant: ‘invalid’ (i.e. with low call rate or allele frequency patterns inconsistent with the type of selection in the region), ‘valid’ (high call rate and consistent allele frequency patterns) or ‘candidate’ (those ‘valid’ variants with the highest value of the statistic, also listed in the .annot file). When functional candidate variants exist in a region, these are plotted in black, while all other variants are plotted in a color depending on the test represented. Finally, the rate of missing genotypes in sliding windows of 2 kb are represented by the black points on the negative part of the y axis, from 0 (no missing data) to − 1 (only missing data). Horizontal dashed lines at − 0.5 and − 1 help visualize the missing rates achieved at a given position. [file 12711_2023_789_MOESM7_ESM.xz › causal/all10_auto_chro2_145181250-145182212_strong.png]

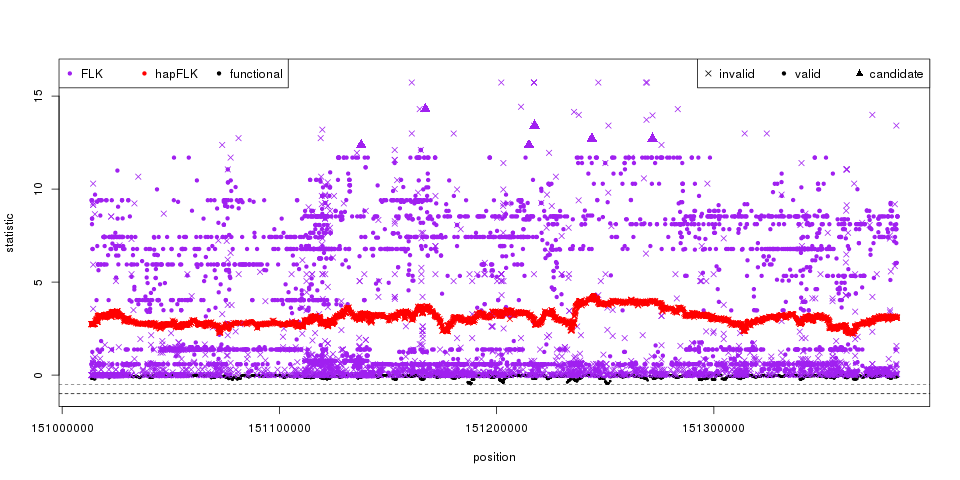

Supplement: Supplementary file 7 — Additional file 7. Candidate causal variants in each candidate region under selection. This archive contains three types of results. (i) Each file with suffix .annot corresponds to a different candidate region, whose genomic position can be read in the file name. It provides a list of candidate variants detected by the statistical approach in this region, together with their SnpEff annotation. (ii) The all strong.func file provides a list of candidate variants with a functional effect (according to SnpEff) selected by this approach for all regions, together with their annotation. The all weak.func file provides the same, but for variants that could only be detected by the functional approach. (iii) Each file with the suffix .png corresponds to a different candidate region, whose genomic position can be read in the file name. It provides a plot showing the values of the relevant statistic(s) in this region with additional details concerning the status of each variant: ‘invalid’ (i.e. with low call rate or allele frequency patterns inconsistent with the type of selection in the region), ‘valid’ (high call rate and consistent allele frequency patterns) or ‘candidate’ (those ‘valid’ variants with the highest value of the statistic, also listed in the .annot file). When functional candidate variants exist in a region, these are plotted in black, while all other variants are plotted in a color depending on the test represented. Finally, the rate of missing genotypes in sliding windows of 2 kb are represented by the black points on the negative part of the y axis, from 0 (no missing data) to − 1 (only missing data). Horizontal dashed lines at − 0.5 and − 1 help visualize the missing rates achieved at a given position. [file 12711_2023_789_MOESM7_ESM.xz › causal/all10_auto_chro2_151013393-151384536_strong.png]

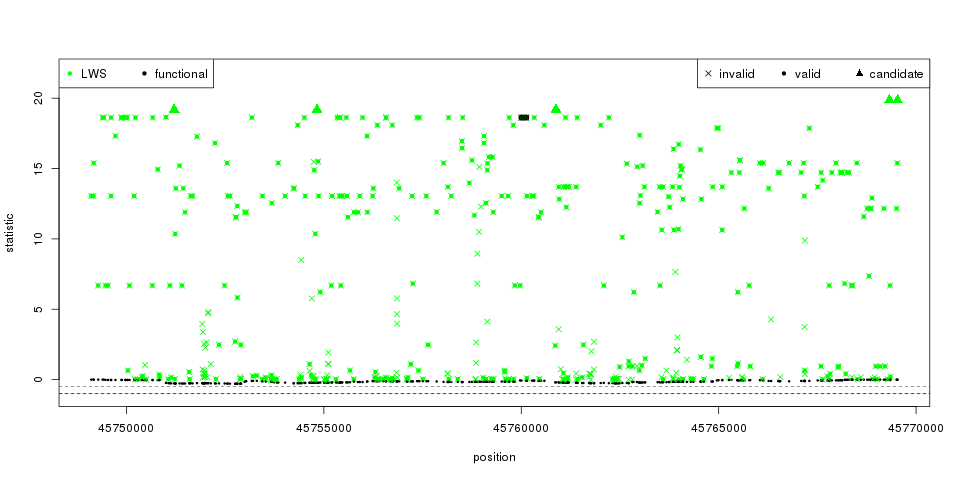

Supplement: Supplementary file 7 — Additional file 7. Candidate causal variants in each candidate region under selection. This archive contains three types of results. (i) Each file with suffix .annot corresponds to a different candidate region, whose genomic position can be read in the file name. It provides a list of candidate variants detected by the statistical approach in this region, together with their SnpEff annotation. (ii) The all strong.func file provides a list of candidate variants with a functional effect (according to SnpEff) selected by this approach for all regions, together with their annotation. The all weak.func file provides the same, but for variants that could only be detected by the functional approach. (iii) Each file with the suffix .png corresponds to a different candidate region, whose genomic position can be read in the file name. It provides a plot showing the values of the relevant statistic(s) in this region with additional details concerning the status of each variant: ‘invalid’ (i.e. with low call rate or allele frequency patterns inconsistent with the type of selection in the region), ‘valid’ (high call rate and consistent allele frequency patterns) or ‘candidate’ (those ‘valid’ variants with the highest value of the statistic, also listed in the .annot file). When functional candidate variants exist in a region, these are plotted in black, while all other variants are plotted in a color depending on the test represented. Finally, the rate of missing genotypes in sliding windows of 2 kb are represented by the black points on the negative part of the y axis, from 0 (no missing data) to − 1 (only missing data). Horizontal dashed lines at − 0.5 and − 1 help visualize the missing rates achieved at a given position. [file 12711_2023_789_MOESM7_ESM.xz › causal/all10_auto_chro2_45749109-45769530_strong.png]

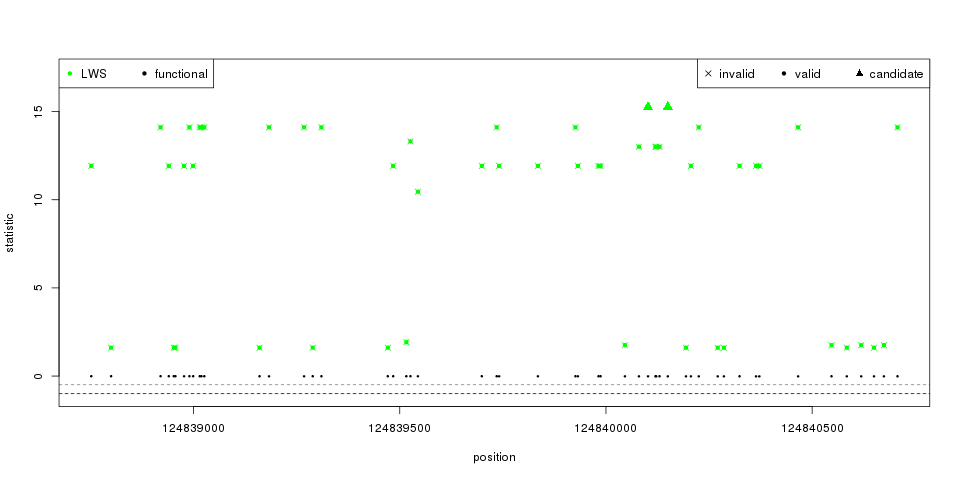

Supplement: Supplementary file 7 — Additional file 7. Candidate causal variants in each candidate region under selection. This archive contains three types of results. (i) Each file with suffix .annot corresponds to a different candidate region, whose genomic position can be read in the file name. It provides a list of candidate variants detected by the statistical approach in this region, together with their SnpEff annotation. (ii) The all strong.func file provides a list of candidate variants with a functional effect (according to SnpEff) selected by this approach for all regions, together with their annotation. The all weak.func file provides the same, but for variants that could only be detected by the functional approach. (iii) Each file with the suffix .png corresponds to a different candidate region, whose genomic position can be read in the file name. It provides a plot showing the values of the relevant statistic(s) in this region with additional details concerning the status of each variant: ‘invalid’ (i.e. with low call rate or allele frequency patterns inconsistent with the type of selection in the region), ‘valid’ (high call rate and consistent allele frequency patterns) or ‘candidate’ (those ‘valid’ variants with the highest value of the statistic, also listed in the .annot file). When functional candidate variants exist in a region, these are plotted in black, while all other variants are plotted in a color depending on the test represented. Finally, the rate of missing genotypes in sliding windows of 2 kb are represented by the black points on the negative part of the y axis, from 0 (no missing data) to − 1 (only missing data). Horizontal dashed lines at − 0.5 and − 1 help visualize the missing rates achieved at a given position. [file 12711_2023_789_MOESM7_ESM.xz › causal/all10_auto_chro3_124838752-124840707_strong.png]
